# Replacing salt with low‐sodium salt substitutes (LSSS) for cardiovascular health in adults, children and pregnant women

**DOI:** 10.1002/14651858.CD015207

**Published:** 2022-08-10

**Authors:** Amanda Brand, Marianne E Visser, Anel Schoonees, Celeste E Naude

**Affiliations:** Centre for Evidence-based Health Care, Division of Epidemiology and Biostatistics, Faculty of Medicine and Health SciencesStellenbosch UniversityCape TownSouth Africa

**Keywords:** Adult, Child, Female, Humans, Pregnancy, Hyperkalemia, Hypertension, Hypertension/drug therapy, Hypokalemia, Potassium, Potassium/therapeutic use, Pregnant Women, Randomized Controlled Trials as Topic, Sodium, Sodium Chloride, Sodium Chloride/therapeutic use, Sodium Chloride, Dietary, Sodium Chloride, Dietary/adverse effects, Stroke

## Abstract

**Background:**

Elevated blood pressure, or hypertension, is the leading cause of preventable deaths globally. Diets high in sodium (predominantly sodium chloride) and low in potassium contribute to elevated blood pressure. The WHO recommends decreasing mean population sodium intake through effective and safe strategies to reduce hypertension and its associated disease burden. Incorporating low‐sodium salt substitutes (LSSS) into population strategies has increasingly been recognised as a possible sodium reduction strategy, particularly in populations where a substantial proportion of overall sodium intake comes from discretionary salt. The LSSS contain lower concentrations of sodium through its displacement with potassium predominantly, or other minerals. Potassium‐containing LSSS can potentially simultaneously decrease sodium intake and increase potassium intake.  Benefits of LSSS include their potential blood pressure‐lowering effect and relatively low cost. However, there are concerns about potential adverse effects of LSSS, such as hyperkalaemia, particularly in people at risk, for example, those with chronic kidney disease (CKD) or taking medications that impair potassium excretion.

**Objectives:**

To assess the effects and safety of replacing salt with LSSS to reduce sodium intake on cardiovascular health in adults, pregnant women and children.

**Search methods:**

We searched MEDLINE (PubMed), Embase (Ovid), Cochrane Central Register of Controlled Trials (CENTRAL), Web of Science Core Collection (Clarivate Analytics), Cumulative Index to Nursing and Allied Health Literature (CINAHL, EBSCOhost), ClinicalTrials.gov and WHO International Clinical Trials Registry Platform (ICTRP) up to 18 August 2021, and screened reference lists of included trials and relevant systematic reviews. No language or publication restrictions were applied.

**Selection criteria:**

We included randomised controlled trials (RCTs) and prospective analytical cohort studies in participants of any age in the general population, from any setting in any country. This included participants with non‐communicable diseases and those taking medications that impair potassium excretion. Studies had to compare any type and method of implementation of LSSS with the use of regular salt, or no active intervention, at an individual, household or community level, for any duration.

**Data collection and analysis:**

Two review authors independently screened titles, abstracts and full‐text articles to determine eligibility; and extracted data, assessed risk of bias (RoB) using the Cochrane RoB tool, and assessed the certainty of the evidence using GRADE. We stratified analyses by adults, children (≤ 18 years) and pregnant women. Primary effectiveness outcomes were change in diastolic and systolic blood pressure (DBP and SBP), hypertension and blood pressure control; cardiovascular events and cardiovascular mortality were additionally assessed as primary effectiveness outcomes in adults. Primary safety outcomes were change in blood potassium, hyperkalaemia and hypokalaemia.

**Main results:**

We included 26 RCTs, 16 randomising individual participants and 10 randomising clusters (families, households or villages). A total of 34,961 adult participants and 92 children were randomised to either LSSS or regular salt, with the smallest trial including 10 and the largest including 20,995 participants. No studies in pregnant women were identified. Studies included only participants with hypertension (11/26), normal blood pressure (1/26), pre‐hypertension (1/26), or participants with and without hypertension (11/26). This was unknown in the remaining studies. The largest study included only participants with an elevated risk of stroke at baseline. Seven studies included adult participants possibly at risk of hyperkalaemia. All 26 trials specifically excluded participants in whom an increased potassium intake is known to be potentially harmful. The majority of trials were conducted in rural or suburban settings, with more than half (14/26) conducted in low‐ and middle‐income countries.

The proportion of sodium chloride replacement in the LSSS interventions varied from approximately 3% to 77%. The majority of trials (23/26) investigated LSSS where potassium‐containing salts were used to substitute sodium. In most trials, LSSS implementation was discretionary (22/26). Trial duration ranged from two months to nearly five years.

We assessed the overall risk of bias as high in six trials and unclear in 12 trials.

*LSSS compared to regular salt in adults:* LSSS compared to regular salt probably reduce DBP on average (mean difference (MD) ‐2.43 mmHg, 95% confidence interval (CI) ‐3.50 to ‐1.36; 20,830 participants, 19 RCTs, moderate‐certainty evidence) and SBP (MD ‐4.76 mmHg, 95% CI ‐6.01 to ‐3.50; 21,414 participants, 20 RCTs, moderate‐certainty evidence) slightly.

On average, LSSS probably reduce non‐fatal stroke (absolute effect (AE) 20 fewer/100,000 person‐years, 95% CI ‐40 to 2; 21,250 participants, 3 RCTs, moderate‐certainty evidence), non‐fatal acute coronary syndrome (AE 150 fewer/100,000 person‐years, 95% CI ‐250 to ‐30; 20,995 participants, 1 RCT, moderate‐certainty evidence) and cardiovascular mortality (AE 180 fewer/100,000 person‐years, 95% CI ‐310 to 0; 23,200 participants, 3 RCTs, moderate‐certainty evidence) slightly, and probably increase blood potassium slightly (MD 0.12 mmol/L, 95% CI 0.07 to 0.18; 784 participants, 6 RCTs, moderate‐certainty evidence), compared to regular salt.

LSSS may result in little to no difference, on average, in hypertension (AE 17 fewer/1000, 95% CI ‐58 to 17; 2566 participants, 1 RCT, low‐certainty evidence) and hyperkalaemia (AE 4 more/100,000, 95% CI ‐47 to 121; 22,849 participants, 5 RCTs, moderate‐certainty evidence) compared to regular salt. The evidence is very uncertain about the effects of LSSS on blood pressure control, various cardiovascular events, stroke mortality, hypokalaemia, and other adverse events (very‐low certainty evidence).

*LSSS compared to regular salt in children:* The evidence is very uncertain about the effects of LSSS on DBP and SBP in children. We found no evidence about the effects of LSSS on hypertension, blood pressure control, blood potassium, hyperkalaemia and hypokalaemia in children.

**Authors' conclusions:**

When compared to regular salt, LSSS probably reduce blood pressure, non‐fatal cardiovascular events and cardiovascular mortality slightly in adults. However, LSSS also probably increase blood potassium slightly in adults. These small effects may be important when LSSS interventions are implemented at the population level. Evidence is limited for adults without elevated blood pressure, and there is a lack of evidence in pregnant women and people in whom an increased potassium intake is known to be potentially harmful, limiting conclusions on the safety of LSSS in the general population. We also cannot draw firm conclusions about effects of non‐discretionary LSSS implementations. The evidence is very uncertain about the effects of LSSS on blood pressure in children.

## Plain language summary

**Does using low‐sodium salt substitutes (LSSS) instead of regular salt reduce blood pressure and heart disease risks, and is it safe?**

**Key messages**

• In adults, using LSSS instead of regular salt in food probably lowers blood pressure slightly. Adults using LSSS instead of regular salt probably have a slightly lower risk of non‐fatal heart conditions, such as stroke or a sudden reduced blood flow to the heart, and death from heart disease.

• Using LSSS instead of regular salt probably also slightly increases the level of blood potassium (a mineral that keeps your heart beating at the right pace) in adults. This could be harmful for people who cannot effectively regulate the potassium in their bodies. Other evidence on safety is very limited.

• We are not certain about effects of using LSSS instead of regular salt on blood pressure in children, or whether using LSSS is safe in children.

• This evidence may not directly apply to people known to be at risk of high blood potassium, such as people with kidney problems or on certain medications.

**What are low‐sodium salt substitutes (LSSS)?**

LSSS are products with less sodium than regular salt. Amounts of sodium in LSSS are lowered by replacing some of the sodium with potassium or other minerals. LSSS may help lower risks of using regular salt, since eating lots of sodium and not enough potassium contributes to high blood pressure. Globally, high blood pressure is the largest cause of preventable deaths, mainly because it causes stroke, acute coronary syndrome (ACS; where less blood flows to the heart), and kidney problems.

However, LSSS also has potential health risks. Using LSSS may lead to higher than normal blood potassium (hyperkalaemia), which causes problems with the heartbeat speed and rhythm, or can cause the heart to stop. These risks are greater in certain people, for example, those whose kidneys do not work properly to remove potassium.

**What did we want to find out?**

We wanted to find out what the effects of using LSSS instead of regular salt are on blood pressure as well as on events (stroke and ACS) and heart disease death. We also wanted to know if using LSSS instead of regular salt is safe, both in the general population and in people who are known to be at risk of high blood potassium levels.

We wanted to find this out for adults, children and pregnant women.

**What did we do?** We searched five electronic databases and trial registries for studies that compared using LSSS with using regular salt. We compared and summarised the results of the studies and rated our confidence in the combined evidence, based on factors such as study methods and sizes.

**What did we find?**

We found 26 trials* involving 34,961 adults and 92 children. No studies in pregnant women were found. Most trials were undertaken in rural or suburban areas, with more than half done in low‐ and middle‐income countries. Most trials included some people with high blood pressure (22); the largest included only people with a high risk of stroke. Seven trials were done in people at possible risk of high blood potassium. All trials excluded people where high potassium intake is known to be harmful, such as people with kidney problems or on certain medications. Nearly all trials (23) examined LSSS types where some sodium was replaced with potassium. The amount of sodium replaced in the various LSSS used in the trials ranged from very small (3%) to large (77%).

*Trials are types of studies in which participants are assigned randomly to two or more treatment groups. This is the best way to ensure similar groups of participants.

**Main results**

In adults, LSSS probably lowers blood pressure (diastolic and systolic) slightly when compared to regular salt. Using LSSS also probably lowers risk of non‐fatal stroke, non‐fatal ACS and heart disease death slightly when compared to regular salt.

However, using LSSS instead of regular salt probably also slightly increases the level of potassium in the blood.

Compared to regular salt, LSSS may result in little to no difference in high blood pressure and hyperkalaemia.

We could not draw any conclusions about effects of LSSS on blood pressure control, various heart disease events, death caused by stroke, lower than normal blood potassium (hypokalaemia), and other adverse events.

We could not draw any conclusions about the effects or safety of using LSSS instead of regular salt in children.

**What are the limitations of the evidence? **

We are moderately confident in the evidence. Our confidence was lowered mainly because of concerns about how some trials were conducted, and whether the results apply to the general population. We are not sure about the effects and safety of LSSS in children, pregnant women, people known to have a risk of high blood potassium, or those who do not have high blood pressure. We are also unsure about the effects of LSSS when used in foods not prepared at home. Further research may change these results.

**How up to date is this evidence?**

The evidence is up‐to‐date to August 2021.

## Summary of findings

**Summary of findings 1:** Summary of findings table ‐ LSSS intervention compared to regular salt in adults (≥ 18 years) in the general population

**LSSS intervention compared to regular salt in adults (≥ 18 years) in the general population**						
**Patient or population:** adults (≥ 18 years) in the general population **Setting:** any setting in any country **Intervention:** LSSS intervention **Comparison:** regular salt						
**Outcomes**	Anticipated absolute effects^*^ (95% CI)		**Relative effect (95% CI)**	**№ of participants (studies)**	**Certainty of the evidence (GRADE)**	**Comments**
	**Risk with regular salt**	**Risk with LSSS intervention**				
Change in DBP (mmHg) follow‐up: range 56 days to 3 years	The mean change in DBP (mmHg) was **‐0.74** mmHg	MD **2.43 mmHg lower** (3.5 lower to 1.36 lower)	‐	20830 (19 RCTs)	⊕⊕⊕⊝ Moderate^a^	LSSS interventions probably reduce DBP (mmHg) slightly.
Change in SBP (mmHg) follow‐up: range 56 days to 3 years	The mean change in SBP (mmHg) was **‐1.32** mmHg	MD **4.76 mmHg lower** (6.01 lower to 3.5 lower)	‐	21414 (20 RCTs)	⊕⊕⊕⊝ Moderate^b^	LSSS interventions probably reduce SBP (mmHg) slightly.
Hypertension follow‐up: 18 months	580 per 1000	**563 per 1000** (522 to 598)	**RR 0.97** (0.90 to 1.03)	2566 (1 RCT)	⊕⊕⊝⊝ Low^c,^^d^	LSSS interventions may result in little to no difference in hypertension.
Blood pressure control follow‐up: range 8 weeks to 3 months	128 per 1000	**271 per 1000** (169 to 436)	**RR 2.12** (1.32 to 3.41)	253 (2 RCTs)	⊕⊝⊝⊝ Very low^e,^^f,^^g^	The evidence is very uncertain about the effect of LSSS interventions on blood pressure control.
Cardiovascular events: various follow‐up: range ≤ 3 to > 3‐12 months	1623 per 100,000	**1980 per 100,000** (795 to 4933)	**RR 1.22** (0.49 to 3.04)	982 (5 RCTs)	⊕⊝⊝⊝ Very low^h,^^i^	The evidence is very uncertain about the effect of LSSS interventions on various other cardiovascular events.
Cardiovascular events: non‐fatal stroke follow‐up: range ≤ 3 to > 12 months	198 per 100,000	**178 per 100,000** (158 to 200)	**RR 0.90** (0.80 to 1.01)	21250 (3 RCTs)	⊕⊕⊕⊝ Moderate^j^	LSSS interventions probably reduce non‐fatal stroke events slightly.
Cardiovascular events: non‐fatal acute coronary syndrome (events per 100,000 person‐years) follow‐up: mean 4.75 years	512 per 100,000	**358 per 100,000** (266 to 481)	**Rate ratio 0.70** (0.52 to 0.94)	20995 (1 RCT)	⊕⊕⊕⊝ Moderate^j^	LSSS interventions probably reduce non‐fatal acute coronary syndrome events slightly.
Cardiovascular mortality (events per 100,000 person‐years) follow‐up: range mean 2.6 to 13 years	786 per 100,000	**605 per 100,000** (472 to 786)	**Rate ratio 0.77** (0.60 to 1.00)	23200 (3 RCTs)	⊕⊕⊕⊝ Moderate^j^	LSSS interventions probably reduce cardiovascular mortality slightly.
Stroke mortality (events per 100,000 person‐years) follow‐up: range mean 4.75 to 13 years	405 per 100,000	**259 per 100,000** (134 to 506)	**Rate ratio 0.64** (0.33 to 1.25)	21423 (2 RCTs)	⊕⊝⊝⊝ Very low^j,^^k^	The evidence is very uncertain about the effect of LSSS interventions on stroke mortality.
Change in blood potassium (mmol/L) follow‐up: range 56 days to 1.5 years	The mean change in blood potassium (mmol/L) was **0.01** mmol/L	MD **0.12 mmol/L higher** (0.07 higher to 0.18 higher)	‐	784 (6 RCTs)	⊕⊕⊕⊝ Moderate^l^	LSSS interventions probably increase blood potassium (mmol/L) slightly.
Hyperkalaemia follow‐up: range 3 months to mean 4.75 years	88 per 100,000	**91 per 100,000** (40 to 209)	**RR 1.04** (0.46 to 2.38)	22849 (5 RCTs)	⊕⊕⊕⊝ Moderate^m^	LSSS interventions likely result in little to no difference in hyperkalaemia.
Hypokalaemia follow‐up: 12 weeks	One small trial in younger, hypertensive participants receiving potassium supplementation due to the use of potassium‐depleting diuretics reported no hypokalaemia events in the intervention (n = 12) or control (n = 10) group.			22 (1 RCT)	⊕⊝⊝⊝ Very low^c,^^n^	The evidence is very uncertain about the effect of LSSS interventions on hypokalaemia.
Adverse events: other follow‐up: range ≤ 3 to > 12 months	Eight trials reported other adverse events, with a total of 25/1094 (2.3%) and 14/1015 (1.4%) diverse adverse events reported across studies in the intervention and control groups, respectively (not pooled).			2109 (8 RCTs)	⊕⊝⊝⊝ Very low^l,^^o,^^p^	The evidence is very uncertain about the effect of LSSS interventions on other adverse events.
*The risk in the intervention group (and its 95% confidence interval) is based on the assumed risk in the comparison group and the **relative effect** of the intervention (and its 95% CI). **CI:** confidence interval; **MD:** mean difference; **RR:** risk ratio						
**GRADE Working Group grades of evidence** **High certainty:** we are very confident that the true effect lies close to that of the estimate of the effect. **Moderate certainty:** we are moderately confident in the effect estimate: the true effect is likely to be close to the estimate of the effect, but there is a possibility that it is substantially different. **Low certainty:** our confidence in the effect estimate is limited: the true effect may be substantially different from the estimate of the effect. **Very low certainty:** we have very little confidence in the effect estimate: the true effect is likely to be substantially different from the estimate of effect.						
See interactive version of this table: https://gdt.gradepro.org/presentations/#/isof/isof_question_revman_web_431386681611398744.						

^a^ Serious inconsistency: Substantial heterogeneity (I^2 = 88%), not explained by subgroup analyses (study duration, ethnicity, BP status, type of LSSS, baseline Na excretion) or meta‐regression (type of LSSS, baseline Na excretion, overall risk of bias) ^b^ Serious inconsistency: Substantial heterogeneity (I^2 = 78%), not explained by subgroup analyses (study duration, ethnicity, BP status, type of LSSS, baseline Na excretion) or meta‐regression (type of LSSS, baseline Na excretion, overall risk of bias) ^c^ Serious risk of bias: All information is from a study at unclear overall risk of bias ^d^ Serious imprecision: 95%CI is consistent with the possibility of important benefit and unimportant harm (minimally important threshold: 5000 per 100,000) ^e^ Serious risk of bias: The majority of information is from a study at unclear overall risk of bias ^f^ Serious indirectness: majority of information is from a study using an LSSS with 97% NaCl (table salt) ^g^ Serious imprecision: 95% CI was consistent with the possibility of unimportant and important benefit (minimally important threshold 5000 per 100,000) ^h^ Serious indirectness: Pooled effect is driven by a large study in high‐risk individuals (participants selected based on high risk of future vascular disease) that is less likely to be directly applicable to the general population ^i^ Very serious imprecision: Using the OIS approach, the ratio of the upper to the lower boundary of the 95% CI is more than 3 (RR); 18 events in total ^j^ Serious indirectness: Pooled effect is driven by a large secondary prevention trial (73% of participants with previous stroke) that is less likely to be directly applicable to the general population, and that reported limited data on safety outcomes ^k^ Very serious imprecision: The ratio of the upper to the lower boundary of the 95% CI is more than 3 ^l^ Serious risk of bias: The majority of information is from studies at high or unclear overall risk of bias ^m^ Serious risk of bias: The majority of information is from a study at high overall risk of bias ^n^ Very serious indirectness: All information is from a study including younger hypertensive participants with a different rationale for administering LSSS (potassium supplementation due to the use of potassium‐depleting diuretics) ^o^ Serious inconsistency: Other adverse event outcomes were too diverse to pool ^p^ Serious imprecision: 39 events in total, OIS not met (not rare events)

**Summary of findings 2:** Summary of findings table ‐ LSSS intervention compared to regular salt in children (2 to < 18 years) in the general population

**LSSS intervention compared to regular salt in children (2 to < 18 years) in the general population**						
**Patient or population:** children (2 to < 18 years) in the general population **Setting:** any setting in any country **Intervention:** LSSS intervention **Comparison:** regular salt						
**Outcomes**	Anticipated absolute effects^*^ (95% CI)		**Relative effect (95% CI)**	**№ of participants (studies)**	**Certainty of the evidence (GRADE)**	**Comments**
	**Risk with regular salt**	**Risk with LSSS intervention**				
Change in DBP (mmHg) follow‐up: 4 months	The mean change in DBP (mmHg) was **‐5.87** mmHg	MD **1.28 mmHg higher** (1.56 lower to 4.12 higher)	‐	92 (1 RCT)	⊕⊝⊝⊝ Very low^a,^^b,^^c^	The evidence is very uncertain about the effect of LSSS interventions on change in DBP (mmHg).
Change in SBP (mmHg) follow‐up: 4 months	The mean change in SBP (mmHg) was **‐6.05** mmHg	MD **0.12 mmHg higher** (4.41 lower to 4.64 higher)	‐	92 (1 RCT)	⊕⊝⊝⊝ Very low^a,^^b,^^c^	The evidence is very uncertain about the effect of LSSS intervention on change in SBP (mmHg).
Hypertension ‐ not measured	‐	‐	‐	‐	‐	No studies in children reported on this outcome.
Blood pressure control ‐ not measured	‐	‐	‐	‐	‐	No studies in children reported on this outcome.
Change in blood potassium (mmol/L) ‐ not measured	‐	‐	‐	‐	‐	No studies in children reported on this outcome.
Hyperkalaemia ‐ not measured	‐	‐	‐	‐	‐	No studies in children reported on this outcome.
Hypokalaemia ‐ not measured	‐	‐	‐	‐	‐	No studies in children reported on this outcome.
*The risk in the intervention group (and its 95% confidence interval) is based on the assumed risk in the comparison group and the **relative effect** of the intervention (and its 95% CI). **CI:** confidence interval; **MD:** mean difference						
**GRADE Working Group grades of evidence** **High certainty:** we are very confident that the true effect lies close to that of the estimate of the effect. **Moderate certainty:** we are moderately confident in the effect estimate: the true effect is likely to be close to the estimate of the effect, but there is a possibility that it is substantially different. **Low certainty:** our confidence in the effect estimate is limited: the true effect may be substantially different from the estimate of the effect. **Very low certainty:** we have very little confidence in the effect estimate: the true effect is likely to be substantially different from the estimate of effect.						
See interactive version of this table: https://gdt.gradepro.org/presentations/#/isof/isof_question_revman_web_431387925381133163.						

^a^ Serious risk of bias: All information is from a study at unclear overall risk of bias ^b^ Serious indirectness: LSSS was delivered in bread only (non‐discretionary) for 4 months ^c^ Serious imprecision: Wide 95% CI including both reductions and increases in blood pressure

## Background

### Description of the condition

High blood pressure is the leading cause of preventable deaths worldwide, contributing to more than 10 million deaths and 211 million disability‐adjusted life years annually, mainly due to acute coronary syndrome (formerly called ischaemic heart disease) and stroke (Forouzanfar 2017).

Hypertension is typically defined by a diastolic blood pressure (DBP) ≥ 90 mmHg and a systolic blood pressure (SBP) ≥ 140 mmHg, although recent guidelines define stage 1 hypertension as a SBP ranging from 130 to 139 mmHg and a DBP ranging from 80 to 89 mmHg to reflect current blood‐pressure lowering targets (Arnett 2019).  After 50 years of age, SBP increases disproportionately to DBP in many individuals due to factors such as reduced arterial stiffness (Franklin 2011; Lee 2010), with an elevated SBP being a prominent risk factor for cardiovascular events in older people (Staessen 2000). In 2015, an estimated 874 million adults had a SBP of 140 mmHg or higher (Forouzanfar 2017).

High sodium together with insufficient potassium intake contribute to hypertension, thereby increasing the risk of cardiovascular disease and stroke. Current global estimates of sodium intake are 3950 mg (172 mmol) per person per day (Powles 2013), which equates to nearly ten grams of salt (sodium chloride) per person per day. For sodium, current World Health Organization (WHO) guidelines strongly recommend reducing intake in adults to < 2 g/day sodium (equating to about 5 g salt per day) and a downward adjusted intake in children (WHO 2012a). Global estimates of potassium intake for all ages, education levels, residences and sexes in 2018 are 2.3 grams per person per day (Global Dietary Database 2022), which equates to an intake of 59 mmol per person per day. For potassium, WHO conditionally recommends an intake in adults of at least 90 mmol/day (3150 mg/day) and a downward adjusted intake in children (WHO 2012b).  Although antihypertensive drug therapy is an effective method for controlling blood pressure, poor adherence to antihypertensive therapy substantially increases the near‐ and long‐term risk of stroke among patients with hypertension (Herttua 2013), and access to health care such as blood‐pressure lowering medication is not universally available.

Hypertension is also a major contributor to the development and progression of chronic kidney disease (CKD). Adequate blood pressure control has been shown to be effective in slowing the progression of CKD to end stage renal disease (ESRD). In addition, adequate treatment of diabetes and cardiovascular risk factors such as dyslipidemia, are also linked to lower rates of progression to ESRD, and associated with significant reductions in cardiovascular morbidity and mortality (Couser 2011).

### Description of the intervention

The WHO target of a 30% relative reduction in mean population salt/sodium intake by 2025 requires effective and safe strategies to reduce population intake. One of several existing salt reduction strategies is using salt products with lower concentrations of sodium ‐ usually replaced by potassium or other minerals, or both. These low‐sodium salt substitutes (LSSS) vary widely in their formulations and are available in high‐income as well as low‐ and middle‐income countries. In many LSSS, a proportion of sodium chloride (NaCl) is replaced with potassium chloride (KCl), which shares many properties with NaCl but also has unwanted relatively offensive side tastes (bitter, acrid, and metallic). A recent narrative review (Cepanec 2017) described the many various formulations of KCl‐based LSSS, which include the use of numerous taste‐improving agents (TIAs) and formulation concepts. Authors concluded that “within the great number of various compositions of KCl‐based salt substitutes, presumably the most effective ones are based on well‐balanced mixtures of KCl and NaCl, maintaining a sodium reduction range from −25% to −50% (relative to NaCl), which always include certain percentages of one or more TIAs. A typical formulation of a KCl‐based salt substitute with 50% in sodium reduction is 50% NaCl + 30‐45% KCl + 5‐20% taste‐improving agents.” Incorporating salt substitutes into population strategies to reduce sodium intake has increasingly been recognised by health authorities and public health organisations (Greer 2020), especially in countries where the majority of sodium intake comes from the discretionary use of salt by households.

### How the intervention might work

The dose‐response relationship between reduced dietary sodium and blood pressure change was examined in a recent systematic review (133 studies with 12,197 participants). Authors showed that in diverse populations, lower sodium intakes resulted in blood pressure reductions, with greater reductions in sodium intake producing greater reductions in BP (Huang 2020). Additionally, older and non‐white populations (for SBP), as well as those with higher baseline blood pressure (for SBP and DBP) achieved greater blood pressure reductions from the same amount of sodium reduction (Huang 2020). Reductions in blood pressure, such as a reduction of 5% in SBP, translate to important reductions (10%) in the risk of major cardiovascular events (e.g. fatal or non‐fatal stroke or myocardial infarction), as demonstrated by a recent meta‐analysis of individual participant‐level data from 48 trials of blood‐pressure lowering medication (Rahimi 2021). Observational studies have demonstrated that stroke risk is inversely associated with dietary potassium intake (Vinceti 2016). In addition, data from randomised clinical trials have shown that potassium supplements have a blood‐pressure lowering effect in people with hypertension, particularly those with a high sodium intake (Filippini 2017). As described in the Description of the condition section, global estimates of potassium intake are lower than what is currently recommended by the WHO. The low dietary intake of potassium, in addition to high dietary sodium intake, contributes to hypertension. Therefore, interventions or strategies promoting the use of a potassium‐enriched LSSS could aid in reducing sodium intake, while concurrently increasing potassium intake, at the population level. 

Reduction in sodium intakes ‐ either through reduction of dietary salt intake, salt substitution, or a combination of these ‐ may also be a practical choice for patients with hypertension who are resistant to antihypertensive medications or who experience side effects from medications. It may also play an important role as an adjunctive therapy in the management of hypertensive individuals by potentially lowering the doses of antihypertensive medication required. In cases where the behavioural changes required to reduce dietary salt intake are very difficult or unfeasible, salt substitutes may offer convenience and practicality. Therefore, salt substitution as a cost‐effective strategy could result in reductions in health‐care costs associated with non‐communicable diseases at a population level. However, it should be noted that if foods with high levels of non‐discretionary sodium chloride are regularly consumed, the discretionary use of LSSS may not result in a sufficient reduction in sodium intake to be beneficial. 

LSSS may offer a potential solution for the food industry to develop lower sodium food products without compromising on taste or safety, particularly in countries where non‐discretionary sodium intake contributes significantly to the overall population intake of sodium. However, because KCl costs more than NaCl, significant consumer demand, industry‐targeted subsidies or taxes on high sodium content foods will likely be required before the food industry will absorb the costs of product reformulation. Therefore, the application of LSSS strategies at population level to reduce sodium consumption is dependent on several factors, including its main uses within a population, as well as its effects on food taste and cost (Greer 2020).

The greatest risk with potassium‐based LSSS is the potential for adverse effects resulting from hyperkalaemia, particularly the increased risk of arrhythmias and sudden cardiac death. The risk of adverse events is greater at higher levels of serum potassium. There is no absolute threshold at which these adverse events occur, however a serum potassium level of ≥ 6.0 mmol/L is commonly considered to be a clinically significant threshold above which the most serious manifestations of hyperkalaemia occur (Ahee 2000; Hollander‐Rodriguez 2006). Multiple factors influence the occurrence of these adverse events, such as the underlying cause of hyperkalaemia and the rate at which serum potassium increases. High intakes of dietary potassium have not been linked to adverse effects in healthy adults and children with normal kidney function. However, the effects of high dietary potassium intakes on the risk of adverse effects are a key concern among people with impaired potassium excretion, such as those with chronic kidney disease or taking medications that impair potassium excretion (Greer 2020; Kovesdy 2018).

A reduction of dietary sodium intake through the population‐level implementation of LSSS may also result in hyponatraemia in people with impaired renal function (Sahay 2014), including older people and people treated with thiazide diuretics (Upadhyay 2009).

### Why it is important to do this review

If the best available evidence on replacing salt with LSSS shows adequate effectiveness and safety for important outcomes, it could be recommended as a population‐level intervention for reducing cardiovascular disease risk. However, concerns exist about potential adverse effects of LSSS, such as hyperkalaemia, particularly in those at risk, such as people with chronic kidney disease or on medications that impair potassium excretion. 

The WHO is currently developing a guideline on the use of LSSS in adults and children. This review was commissioned by the WHO Nutrition Guidance Expert Advisory Group (NUGAG) Subgroup on Diet and Health in order to inform and contribute to the development of a WHO recommendation on the use of LSSS for this guideline. The results of this review, including Grading of Recommendations, Assessment, Development and Evaluations (GRADE) assessments, were discussed and reviewed by the WHO NUGAG Subgroup on Diet and Health as part of their guideline development process.

## Objectives

To assess the effects and safety of replacing salt with LSSS to reduce sodium intake on cardiovascular health in adults, pregnant women and children.

## Methods

### Criteria for considering studies for this review

#### Types of studies

The Populations, Intervention, Comparison, and Outcomes (PICO) were agreed by the WHO NUGAG Subgroup on Diet and Health, who ranked the outcomes and also agreed on subgroups and study designs to be included. As per these agreements and our prospective registration on the international prospective register of systematic reviews (PROSPERO 2020 CRD42020180162; available at https://www.crd.york.ac.uk/prospero/display_record.php?RecordID=180162), we included individually randomised controlled trials (RCTs) and cluster‐randomised controlled trials (cluster‐RCTs) with true randomisation methods, regardless of the unit of allocation. We also planned to include prospective analytical cohort studies, where LSSS intake/exposure was assessed at baseline and related to any of the prespecified outcomes at a later time point using empirical data. We excluded RCTs with a cross‐over trial design if data for the first phase per group were unavailable, due to the possible period and carry‐over effects that would arise with the eligible dietary interventions/exposures and outcomes not being easily reversible, as required for a valid cross‐over design (Younge 2015). We additionally excluded cluster‐RCTs with fewer than two intervention and two control clusters.

#### Types of participants

 We included studies in the general population, from any setting in any country, including participants with the following condition(s) and/or risk factors: hypertension, cardiovascular disease (CVD), diabetes mellitus, renal impairment and those taking medications that impair potassium excretion. 

In accordance with the WHO NUGAG PICO conceptualisations and agreements,  the following three comparisons were planned if data allowed:

1. LSSS versus regular salt or no active intervention in adults (aged 18 years and older)
2. LSSS versus regular salt or no active intervention in children aged 2 to < 18 years
3. LSSS versus regular salt or no active intervention in pregnant women

#### Types of interventions

We included studies that assessed the health effects associated with the use of LSSS at an individual, household or community level. LSSS interventions/exposures of any type or duration were included, provided they aimed to replace the dietary intake of any amount of sodium with another mineral or compound. Studies investigating either discretionary (i.e. salt on table or added during cooking) or non‐discretionary use of LSSS (i.e. included during food manufacturing), or both, were included. 

Eligible comparators/controls included the use of regular salt (NaCl) or no active intervention to reduce salt intake. Studies where the control group received only basic information on sodium reduction at baseline, were included. Studies with multi‐component interventions were included if effects of LSSS could be isolated from the multifactorial design.  

We excluded studies with multi‐component interventions if the additional intervention components were not aimed primarily at promoting LSSS use by participants or communities, but were instead focussed more broadly on reducing sodium intake (e.g. changing lifestyle and dietary behaviour of which LSSS use is only one component) or aimed at improving health in general (e.g. counselling for exercise or smoking cessation), such that LSSS effects could not be isolated.

#### Types of outcome measures

We did not exclude studies on the basis of outcomes measured. However, we did exclude studies measuring only sensory or organoleptic outcomes (e.g. taste of or preference for LSSS). 

##### Primary outcomes

Table 3, Table 4 and Table 5 detail the prespecified primary outcomes for each comparison, with outcome ranking by the WHO NUGAG Subgroup on Diet and Health indicated as follows: critical^c^, important^i^ and not important ^ni^.  The following primary outcomes were regarded as safety outcomes related to the intake of LSSS with potassium: change in blood potassium, hyperkalaemia and hypokalaemia.

**1:** Primary and secondary outcomes for the comparison in adults

Primary clinical outcomes*	Primary laboratory outcomes*	Secondary clinical outcomes*	Secondary laboratory outcomes*
**Change in diastolic blood pressure (DBP, mmHg)^c^**	**Change in blood potassium (mmol/L)^c^**	All‐cause mortality^i^	Renal function (e.g. serum creatinine, albuminuria, urinary albumin‐to‐creatinine ratio (uACR), glomerular filtration rate (GFR))^i^
**Change in systolic blood pressure (SBP, mmHg)^c^**	**Hyperkalaemia (e.g. number of adults with serum potassium concentration > 5.5 mmol/L, or as reported by study authors)^c^**	Adverse events (other), excluding those that overlap with other outcomes, such as electrolyte disturbances and cardiac arrhythmias (e.g. nausea, vomiting)^i^	Hyponatraemia (e.g. number of adults with serum sodium concentration < 135 mmol/L, or as reported by study authors)^i^
**Hypertension (e.g. number of adults with SBP > 140 mmHg or DBP > 85 mmHg, or as reported by study authors)^c^**	**Hypokalaemia (e.g. number of adults with serum potassium concentration < 3.5 mmol/L, or as reported by study authors^c^**	Antihypertensive medication use^i^	Change in fasting blood glucose (mmol/L)^i^
**Blood pressure control (e.g. number of adults achieving blood pressure threshold or blood pressure under "control", or as prespecified by study authors)^c^**		Diabetes mellitus diagnosis (as reported by study authors)^i^	Change in blood triglycerides (mmol/L)^i^
**Cardiovascular events (as reported by study authors, such as stroke, myocardial infarction, dysrhythmia)^c^ **		Change in body mass index (BMI) (kg/m^2^)^i^	Change in total blood cholesterol (mmol/L)^i^
**Cardiovascular mortality^c^**			Change in 24‐hour urinary sodium excretion (mmol/24‐hours)**
			Change in 24‐hour urinary potassium excretion (mmol/24‐hours)**

Outcome ranking by WHO NUGAG ‐ Subgroup on Diet and Health: **critical^c^**, important^i^ and not important^ni^ * Outcomes measured at longest follow‐up ** Additional outcomes added by WHO NUGAG during the guideline development process; measured using 24‐h urine samples only (spot samples excluded) Abbreviations: BMI: body mass index DBP: diastolic blood pressure GFR: glomerular filtration rate NUGAG: Nutrition Guidance Expert Advisory Group SBP: systolic blood pressure uACR: urine albumin‐to‐creatinine ratio

**2:** Primary and secondary outcomes for the comparison in pregnant women

Primary clinical outcomes*	Primary laboratory outcomes*	Secondary clinical outcomes in women*	Secondary clinical outcomes in newborns*	Secondary laboratory outcomes*
**Pre‐eclampsia (e.g. number of women meeting the following diagnostic criteria: SBP > 140 mmHg or DBP > 90 mmHg after 20 weeks of pregnancy, with proteinuria and/or other maternal organ dysfunction such as renal, liver neurological or haematological abnormalities, or uteroplacental dysfunction, or as reported by study authors)^c^**	**Change in blood potassium (mmol/L)^c^**	All‐cause mortality^i^	Pre‐term infant (i.e. number of infants born < 37 weeks gestation)^i^	Renal function (e.g. serum creatinine, albuminuria, urinary albumin‐to‐creatinine ratio (uACR), glomerular filtration rate (GFR))^i^
**Eclampsia (e.g. number of women with pre‐eclampsia who present with convulsions, or as reported by study authors)^c^**	**Hyperkalaemia (e.g. number of women with serum potassium concentration > 5.5 mmol/L, or as reported by study authors)^c^**	Cardiovascular mortality^i^	Intra‐uterine growth restriction (IUGR) (e.g. number of small‐for‐gestational age (SGA) infants, defined as those with a birthweight < 2 SD below the reference standard or < 10^th^ percentile, or as reported by study authors)^i^	Change in fasting blood glucose (mmol/L)^i^
**Change in diastolic blood pressure (DBP, mmHg)^c^**	**Hypokalaemia (e.g. number of women with serum potassium concentration < 3.5 mmol/L, or as reported by study authors)^c^**	Adverse events (other), excluding those that overlap with other outcomes, such as electrolyte disturbances and cardiac arrhythmias (e.g. nausea, vomiting)^i^	Birthweight (g)^i^	Change in blood triglycerides (mmol/L)^i^
**Change in systolic blood pressure (SBP, mmHg)^c^**		Antihypertensive medication use^i^		Change in total blood cholesterol (mmol/L)^i^
**Hypertension (e.g. number of women with SBP > 140 mmHg or DBP > 85 mmHg, or as reported by study authors)^c^**		Gestational diabetes diagnosis^i^ (e.g. number of women meeting one of the following diagnostic criteria: fasting plasma glucose 5.1–6.9 mmol/L; 1‐hour plasma glucose 10.0 mmol/L, or 2‐hour plasma glucose 8.5–11.0 mmol/L following a 75 g oral glucose load, or as reported by study authors)		Change in 24‐hour urinary sodium excretion (mmol/24‐hours)**
**Blood pressure control (e.g. number of women achieving blood pressure threshold or blood pressure under "control", or as prespecified by study authors)^c^**		Diabetes mellitus diagnosis (as reported by study authors)^i^		Change in 24‐hour urinary potassium excretion (mmol/24‐hours)**
Cardiovascular events (as reported by study authors, such as stroke^i^, myocardial infarction, **dysrhythmia^c^)**				

Outcome ranking by WHO NUGAG ‐ Subgroup on Diet and Health: **critical^c^**, important^i^ and not important^ni^ * Outcomes measured at longest follow‐up ** Additional outcomes added by WHO NUGAG during the guideline development process; measured using 24‐h urine samples only (spot samples excluded) Abbreviations: DBP: diastolic blood pressure GFR: glomerular filtration rate IUGR: intra‐uterine growth restriction NUGAG: Nutrition Guidance Expert Advisory Group SBP: systolic blood pressure SD: standard deviation SGA: small‐for‐gestational age uACR: urine albumin‐to‐creatinine ratio

**3:** Primary and secondary outcomes for the comparison in children

Primary clinical outcomes*	Primary laboratory outcomes*	Secondary clinical outcomes*	Secondary laboratory outcomes*
**Change in diastolic blood pressure (DBP, mmHg)^c^**	**Change in blood potassium (mmol/L)^c^**	Growth changes (e.g. z‐scores for height‐ or length‐for‐age (HAZ or LAZ), weight‐for‐height (WHZ), weight‐for age (WAZ), BMI‐for‐age)^i^	Renal function (e.g. serum creatinine, albuminuria, urinary albumin‐to‐creatinine ratio (uACR), glomerular filtration rate (GFR))^i^
**Change in systolic blood pressure (SBP, mmHg)^c^**	**Hyperkalaemia (e.g. number of children with serum potassium concentration > 5.5 mmol/L, or as reported by study authors)^c^**	Adverse events (other), excluding those that overlap with other outcomes, such as electrolyte disturbances and cardiac arrhythmias (e.g. nausea, vomiting)^i^	Bone health (e.g. serum alkaline phosphatase (ALP) in mmol/L)^i^
**Hypertension (e.g. as average systolic BP (SBP) and/or diastolic BP (DBP) that is ≥ 95th percentile for gender, age, and height on ≥ 3 occasions, or as reported by study authors)^c^**	**Hypokalaemia (e.g. number of children with serum potassium concentration < 3.5 mmol/L, or as reported by study authors)^c^**	Cardiovascular events (as reported by study author, such as stroke, myocardial infarction, dysrhythmia)^i^	Hyponatraemia (e.g. number of children with serum sodium concentration < 135 mmol/L, or as reported by study authors)^i^
**Blood pressure control (e.g. number of children achieving blood pressure threshold or blood pressure under "control", or as prespecified by study authors)^c^**		Antihypertensive medication use^i^	Changes in fasting blood glucose (mmol/L)^i^
		All‐cause mortality^i^	Changes in blood triglycerides (mmol/L)^i^
		Cardiovascular mortality^i^	Changes in total blood cholesterol (mmol/L)^i^
		Bone densitometry measures (e.g. bone mineral density changes)^i^	Change in 24‐hour urinary sodium excretion (mmol/24‐hours)**
			Change in 24‐hour urinary potassium excretion (mmol/24‐hours)**

Outcome ranking by WHO NUGAG ‐ Subgroup on Diet and Health: **critical^c^**, important^i^ and not important^ni^ * Outcomes measured at longest follow‐up ** Additional outcomes added by WHO NUGAG during the guideline development process; measured using 24‐h urine samples only (spot samples excluded) Abbreviations: ALP: alkaline phosphatase BMI: body mass index BP: blood pressure DBP: diastolic blood pressure GFR: glomerular filtration rate HAZ: height‐for‐age z‐score LAZ: length‐for‐age z‐score NUGAG: Nutrition Guidance Expert Advisory Group SBP: systolic blood pressure uACR: urine albumin‐to‐creatinine ratio WAZ: weight‐for‐age z‐score WHZ: weight‐for‐height z‐score

##### Secondary outcomes

Table 3, Table 4 and Table 5 detail the prespecified secondary outcomes for each comparison, with outcome ranking by the WHO NUGAG Subgroup on Diet and Health indicated as follows: critical^c^, important^i^ and not important ^ni^.  The following secondary outcomes were regarded as safety outcomes related to the intake of LSSS with potassium: adverse events, renal function and hyponatraemia.

### Search methods for identification of studies

The search strategy was developed, peer‐reviewed and implemented by Cochrane information specialists in consultation with the review team. We used a comprehensive search strategy aiming to identify all eligible studies regardless of language, publication type or publication status. Publication date restrictions were not imposed, except for conference abstracts identified through Embase which covered only those published in the past two years. With this, we specifically aimed to find recent proceedings of studies that may not yet have been published as full articles at the time of the search. We used filters for trials (Lefebvre 2022), cohort studies (Li 2019) and adverse effects (Golder 2006; Golder 2012) to inform our search strategy.

#### Electronic searches

We aimed to identify RCTs and prospective analytical cohort studies through systematic searches of the following bibliographic databases: 

- MEDLINE (PubMed, from 1946 to 18 August 2021)
- Embase (Ovid, from 1947 to 18 August 2021)
- Cochrane Central Register of Controlled Trials (CENTRAL), in the Cochrane Library (Issue 8 of 12, 2021)
- Web of Science Core Collection with Indexes SCI‐Expanded, SSCi, CPCI‐S (Clarivate Analytics, from 1970 to 18 August 2021)
- Cumulative Index to Nursing and Allied Health Literature (CINAHL) (EBSCOhost, from 1937 to 18 August 2021)

We also conducted a search of ClinicalTrials.gov (www.ClinicalTrials.gov) and the WHO International Clinical Trials Registry Platform (ICTRP) for ongoing and unpublished trials (https://trialsearch.who.int/). The date of the last searches here was also 18 August 2021. Search strategies per database/registry searched are detailed in Appendix 1. 

#### Searching other resources

To identify any additional eligible records, two reviewers also screened the reference lists of three recent systematic reviews evaluating the effects of LSSS use (Hernandez 2019; Jafarnejad 2020; Jin 2020), as well as the bibliographies of all studies included in this review.

### Data collection and analysis

#### Selection of studies

After de‐duplication of search records, titles and abstracts were screened independently by two reviewers using Covidence (Covidence). Full‐text articles for all records identified as potentially eligible for inclusion were then screened by two reviewers independently to determine final eligibility. Records where we could not obtain the full text or more details of the study in order to determine eligibility, were classified as ‘Studies awaiting classification’.  We resolved any disagreements between reviewers at any stage of the eligibility assessment process through discussion and consultation with a third reviewer, where necessary.

#### Data extraction and management

Two reviewers independently extracted data onto forms designed and piloted for the review, and we resolved any disagreements during the data extraction and management process through discussion and consultation with a third reviewer, where necessary. Where necessary, translations of records in non‐English were obtained. We extracted data on the following:

- Study details, including author details, conflict of interest declaration, funding source, setting
- Methods, including design, aim, dates, limitations as reported by authors, sample size calculation, participants, including eligibility criteria; method of recruitment; number of clusters per trial arm and how authors accounted for the effect of clustering; participant flow details such as number assessed for eligibility, number randomised; baseline characteristic such as demographic and lifestyle characteristics, health status and intake of sodium and potassium; and any differences in these characteristics by trial arm
- Interventions/exposures, including description, delivery/use, addition of fortificants, duration, co‐interventions, integrity of delivery
- Comparators,* *including description, delivery, duration, co‐interventions, integrity of delivery
- Outcomes,* *including numeric data relevant to all primary and secondary outcomes according to the following time point ranges, when available: baseline to 3 months, > 3 to 12 months and > 12 months, except for cardiovascular events, all‐cause mortality, cardiovascular mortality and adverse events, for which data were extracted for the duration of the study. When outcome data were reported at more than one point, we extracted data from the latest point available. For studies that did not use the International System of Units (SI) to report outcomes, we converted values to SI units, where possible. For trials, we extracted change data (change in the outcome from baseline to outcome assessment) with relevant data on variance for intervention and control groups (along with numbers of participants at the time point). Where change data were not available, we extracted end‐values at the time point, along with the variance and numbers of participants for each group, or mean differences (MDs) and measures of variance per group. Where outcome data were only reported per subgroup of the total sample of study participants (e.g. participants with hypertension and participants with normal blood pressure), we extracted these data and calculated the combined mean and standard deviation (SD) for the total sample according to the guidance by (Higgins 2020a), where possible. We preferentially extracted and used supine over standing blood pressure measurements, 24‐hour measurements over measurements done at a single time point, and ambulatory measurements over those conducted in a clinic setting. For cohort studies, we planned to extract the most adjusted odds ratio, risk ratio, mean change or mean end values per group, when comparing the most exposed group of participants with the least exposed group, and the most adjusted regression outputs when LSSS intake was assessed at baseline and related to an outcome measure later.

#### Assessment of risk of bias in included studies

We assessed the risk of bias in RCTs and cluster‐RCTs using the Cochrane tool for assessment of risk of bias (Higgins 2017). Two reviewers conducted these assessments independently for each included study.  We resolved disagreements by discussion or through consultation with a third reviewer. We assessed the risk of bias for RCTs according to the following domains:

- Random sequence generation (selection bias)
- Allocation concealment (selection bias)
- Blinding of participants and personnel (performance bias)
- Blinding of outcome assessment (detection bias)
- Incomplete outcome data (attrition bias)
- Selective outcome reporting (reporting bias)
- Other bias

We also assessed the risk of bias for cluster‐RCTs according to the following domains (Higgins 2017):

- Recruitment bias (selection bias)
- Comparability with RCTs
- Baseline imbalance (selection bias)
- Loss of clusters (attrition bias)
- Incorrect analysis

For cohort studies, we planned to use the following domains to assess risk of bias (Naude 2018):

- Were adequate outcome data available?
- Was there matching of less‐exposed and more‐exposed participants for prognostic factors associated with outcome, or were relevant statistical adjustments done?
- Did the exposures between groups differ in components other than only LSSS exposure?
- Could we be confident in the assessment of outcomes?
- Could we be confident in the assessment of exposure?
- Could we be confident in the assessment of presence or absence of prognostic factors?
- Was selection of less‐exposed and more‐exposed groups from the same population?

##### Overall risk of bias assessment

As this review addressed mainly objective outcomes (e.g. blood pressure measurements, laboratory‐determined electrolyte values), we did not regard blinding to be of key importance for informing judgements on overall bias. Consequently, we judged overall risk of bias for each included study using two key domains for RCTs and four key domains for cluster‐RCTs, as follows:

- RCTs: allocation concealment (selection bias) and incomplete outcome data (attrition bias), and
- Cluster‐RCTs: baseline imbalance (selection bias), recruitment bias (selection bias), incomplete outcome data (attrition bias) and loss of clusters (attrition bias).

We assessed the overall risk of bias of each included study as follows:

- low risk (low risk of bias for all key domains);
- high risk (high risk of bias for one or more key domains); or
- unclear risk (unclear risk of bias for one or more key domains).

For cohort studies, we planned to consider domains relevant to confounding to inform judgements of the overall risk of bias.

#### Measures of treatment effect

For dichotomous outcomes, we presented proportions; for two‐group comparisons where numbers of events and participants were provided, we presented results as risk ratios (RRs) with 95% confidence intervals (CIs). Where event rates were reported per person‐years followed in separate groups, we calculated incidence rate ratios (IRRs) with 95% CIs to enable meta‐analysis of these studies with studies reporting rate ratios for the same outcomes. Rate ratios were calculated by dividing the rate in the intervention group by the rate in the control group. The 95% confidence interval (95% CI) of these rate ratios were calculated by taking the antilogarithm of the natural log of the rate ratio (log(IRR)), plus or minus 1.96 times the standard error of the log(IRR) (Boston University School of Public Health 2018). Briefly, the standard error was calculated as the square root of the sum of the inverse of events in the intervention and control group.

Where hazard ratios (HRs) were reported for incident hypertension in the stepped‐wedge trial (Bernabe‐Ortiz 2014), we presented these results with 95% CIs. Due to the different way of analysing these data and the unique design of this trial, these measures were not combined with other data reporting on hypertension.

For continuous outcomes, we used the mean difference (MD) with 95% CIs if outcomes were measured in the same way between trials. Where continuous data were reported using different units across included studies, we planned to calculate and present the standardised mean difference (SMD).

#### Unit of analysis issues

##### Studies with more than two intervention groups

For the single study with more than two intervention groups (Pan 2017), we combined event outcome data reported separately for both intervention groups (LSSS < 50% KCl and LSSS ≥ 50% KCl) in our meta‐analyses. These intervention groups were combined using the methods set out in the Cochrane Handbook (Higgins 2020a). Another study randomised participants to receive LSSS or continue with their usual practice, after which intervention participants were again randomised to receive LSSS with or without price subsidy (Li 2016). As the LSSS intervention was the same in both these arms, we only extracted and used data for the overall LSSS group (both with and without subsidy) and the usual practice group.

##### Cluster‐RCTs

Four included cluster‐RCTs did not report sufficient information on adjustment for clustering in the statistical analysis or results section of either the full text (Hu 2018; Li 2014; Zhou 2013), or conference abstract (Zhang 2015).  We calculated the effective sample sizes for these trials by calculating the design effect (DE), which is 1 + (c ‐ 1) x ICC, where c is the average cluster size.  Our calculations were based on an estimated intra‐cluster correlation coefficient (ICC) of 0.04, reported by a study conducted in similar trial settings in Northern China (Neal 2017). For continuous data (e.g. DBP, SBP), we adjusted for the sample size only; while for dichotomous outcomes (e.g. cardiovascular events) we divided both the sample size and the number of people who experienced the event by the design effect. Where cluster‐RCTs reporting rates did not account for the effect of clustering in its analyses, we adjusted for clustering by inflating the standard errors by multiplying the standard error of the log(IRR) by the square root of the DE (Higgins 2011). All the estimates from cluster‐RCTs were combined with those from RCTs that had individual group assignment in our meta‐analyses (Higgins 2020b).

#### Dealing with missing data

We contacted study authors to request any missing or unreported data, such as group means, SDs, details of attrition, or details of the type of analysis conducted (e.g. intention‐to‐treat).

In cases where there were missing data due to attrition, we used the data available to conduct available case (modified intention‐to‐treat) meta‐analyses. We assessed the extent and impact of missing data and attrition for each included study during the Risk of bias assessment.

#### Assessment of heterogeneity

For each meta‐analysis, we examined the forest plots visually to determine whether heterogeneity of the size and direction of treatment effect was present between studies. We used the I² statistic, Tau², and the Chi² test to estimate the level of heterogeneity among the studies in each analysis. We defined substantial heterogeneity as Tau² > 0, and either I² > 50% or a low P value (< 0.10) in the Chi² test. Where substantial heterogeneity was found, we noted this in the text and explored it by conducting prespecified subgroup analyses to account for potential sources of clinical heterogeneity (see section: Subgroup analysis and investigation of heterogeneity). We also considered other potential sources of heterogeneity, for example, differences in the nature of the interventions delivered. In addition, we explored methodological sources of heterogeneity by examining studies with different levels of risk of bias in a sensitivity analysis (see section: Sensitivity analysis). We used caution in the interpretation of results with high levels of unexplained heterogeneity. We did not perform a meta‐analysis if the I² statistic was 90% or higher (considerable heterogeneity) (Deeks 2020).

#### Assessment of reporting biases

Where more than 10 included studies addressed a primary outcome, we used funnel plots to assess the possibility of small‐study effects and, in the case of asymmetry, intended to consider various explanations such as publication bias, poor study design and the effect of study size (Sterne 2017).

#### Data synthesis

All syntheses were conducted using Review Manager Web 2021 (RevMan Web 2021). We used a random‐effects meta‐analysis to combine data across more than one study, as we anticipated that there may be natural heterogeneity between studies, attributable to the different study settings, intervention strategies, or both. If a study only reported an MD and variance per group for an outcome, we first calculated MDs and 95% CI for the other studies reporting on that outcome, and then combined MDs and 95% CIs from all studies in a meta‐analysis using generic inverse variance (GIV). If a study reported rate ratios or events per person‐years, from which rate ratios could be calculated (see section: Measures of treatment effect), we also combined these rate ratios and 95% CIs in a meta‐analysis using GIV. Where studies reported event outcomes as rate ratios and risk ratios, these were combined in a meta‐analysis using GIV by using rate ratios as approximations for risk ratios.

We sought to only generate pooled estimates where data from separate studies were similar enough to be combined (see section: Assessment of heterogeneity). Data not suitable for pooling (defined as considerable heterogeneity, I^2^ ≥ 90%) in meta‐analyses were presented in forest plots without the pooled estimate, or in tables, as appropriate. Data from peer‐reviewed publications and conference abstracts were eligible for inclusion in meta‐analysis. Data from conference abstracts were identified in forest plots using footnotes. If needed, we also planned to conduct a narrative synthesis, by adopting a systematic approach to presentation, guided by the reporting guideline, Synthesis Without Meta‐analysis (SWiM) in systematic reviews (Campbell 2020).

#### Subgroup analysis and investigation of heterogeneity

We performed subgroup analyses where data allowed using a test for interaction (i.e. heterogeneity across subgroups rather than across studies), calculating summary effect sizes for each subgroup in a univariate analysis for prespecified subgroups provided by WHO NUGAG, as follows.

##### All comparisons:

- Study duration: short‐term (≤ 3 months) versus medium‐term (> 3 to 12 months) versus long‐term (> 12 months)
- Gender: male versus female versus mixed versus unknown
- Ethnicity: African versus Asian versus European versus mixed versus conducted in one setting (e.g. Europe), but ethnicity unspecified
- Blood pressure status (as defined by study authors): hypertensive versus normotensive versus hypotensive versus mixed versus unknown
- Baseline potassium intake: lower (urinary 24‐hour [24‐h] potassium excretion < 59 mmol [2.3 g] per day) versus higher (urinary 24‐h potassium excretion ≥ 59 mmol [2.3 g] per day) versus unknown or not reported as 24‐h excretion; based on global potassium intake estimates (Global Dietary Database 2022)
- Baseline sodium intake: lower (urinary 24‐h sodium excretion < 172 mmol [3.95 g sodium or 9.88 g sodium chloride] per day) versus higher (urinary 24‐h sodium excretion ≥ 172 mmol [3.95 g sodium or 9.88 g sodium chloride] per day) versus unknown or not reported as 24‐h excretion; based on global sodium intake estimates (Powles 2013)
- Iodine status (as defined by study authors): within normal ranges versus insufficient or deficient versus mixed versus unknown
- Type of LSSS: based on proportion of potassium chloride: ≥ 30% KCl versus < 30% KCl versus unknown versus non‐potassium containing LSSS (based on the description of a 'typical' formulation of potassium‐based salt substitutes with a 50% sodium chloride reduction in Cepanec 2017)
- LSSS implementation: discretionary only (through added LSSS in cooking and at table) versus non‐discretionary only (through consumption of manufactured products) versus discretionary and non‐discretionary
- Salt as fortification vehicle: using salt as fortification vehicle versus not or unknown

##### All comparisons (only safety outcomes):

- Possible risk of hyperkalaemia versus not at risk or unclear risk of hyperkalaemia (according to the criteria and assessment in Table 6), regardless of heterogeneity, only in the primary analyses of the following safety outcomes: change in blood potassium, hyperkalaemia, hypokalaemia and adverse events. The WHO NUGAG made the decision to limit this subgrouping to the safety outcomes since there are no clinical justifications to expect differences in the effects of LSSS on the effectiveness outcomes in populations possibly at risk of hyperkalaemia.

**4:** Summary of criteria and assessments applied to classify the hyperkalaemia risk of participants in the included studies

**Study**	**Renal function reported at baseline (e.g. serum creatinine concentration, GFR) **	**Renal impairment at screening as exclusion criterion (e.g. or serum creatinine concentration, GFR)**	**Blood Pressure status reported at baseline**	**Use of antihypertensive medication reported at baseline**	**Use of antihypertensive medication at screening as exclusion criterion**	**Use of potassium‐sparing medications at screening as exclusion criterion**	**Assessment of hyperkalaemia risk**
Allaert 2013	NR	NR	Hypertensive, % (n/N): 100 (40/40)	No medication	Yes	No	Not at risk
Allaert 2017	NR	Kidney failure or disease (not defined)	Pre‐hypertensive, % (n/N): 100 (22/22); 100 (19/19)	No medication	Yes	No	Not at risk
Arzilli 1986	NR	NR	Hypertensive, % (n/N); 100 (10/10)	NR	NR	NR	Unclear risk ^a^
Bernabe‐Ortiz 2014	NR	A history of terminal or severe chronic kidney disease (receiving any form of dialysis)	(Hypertensive, % (n/N): 18.3 (428/2342) [village A 17.1 (91/534); village B 20.5 (90/449); village C 18.2 (59/329); village D 13.6 (56/414); village E 24.8 (79/328); village F 16.9 (53/322)]	NR	No	Yes	Not at risk
Chang 2006	NR	Serum creatinine ≥ 3.5 mg/dL (>= 309 µmol/L)	Hypertensive, % (n/N): 40.2 (309/768); 40.4 (490/1213)	NR	No	No	Possibly at risk
CSSS Collaborative Group 2007	Serum creatinine in µmol/L, mean (SD): 74.0 (20.0); 74.5 (19.1)	Abnormal serum creatinine concentrations	Hypertensive, % (n/N): 57 (173/306); 57 (172/302)	Any antihypertensive medication, % (n): 61 (185/306); diuretic, % (n): 6 (19/306); ACE inhibitor or ARB, % (n): 10 (31/306); beta‐blocker, % (n): 6 (17/306); calcium antagonist, % (n): 23 (70/306)	No	Yes	Not at risk
Geleijnse 1994	NR	Serum creatinine > 200 µmol/L	Hypertensive, % (n/N): 100 (49/49); 100 (51/51)	No medication	Yes	No	Possibly at risk
Gilleran 1996	NR	Hypertensive nephropathy (persistent proteinuria or serum creatinine > 130 µmol/L)	Hypertensive, % (n/N): 100 (20/20); 100 (20/20)	Stopped one month prior	Yes	No	Not at risk
Hu 2018 (hypertensive participants)	NR	Serum creatinine > 177 µmol/L	Hypertensive, % (n/N):100 (110/110); 100 (110/110)	Antihypertensive medication, % (n/N): 71.8 (79/110); 77.3 (85/110)	No	Yes	Possibly at risk ^b^
Hu 2018 (family members)	NR	Serum creatinine > 177 µmol/L	Family members: Hypertensive, % (n/N): 31.6 (59/187); 24.2 (45/186)	No medication	No	Yes	Possibly at risk ^b^
Kawasaki 1998	NR	NR	Hypertensive, % (n/N): 47.6 (10/21); 40.0 (4/20)	Beta‐blockers, calcium channel blockers or both, n/N: 19.0 (4/21); 20.0 (4/20)	No	No	Not at risk
Li 2014	NR	Serious kidney diseases (not defined)	NR	NR	No	Yes	Not at risk
Li 2016 ^c^	NR	Microalbuminuria, % (n/N) 6.6 (64/969); 9.2 (84/916); macroalbuminuria, % (n/N): 0.01 (5/975); 0.011 (10/928).	Hypertensive, %: 56.5 (731/1294); 58 (738/1272)	Antihypertensive medication use, % (n/N): 19 (246/1294); 21 (267/1272)	NR	NR	Unclear risk
Mu 2003	NR	NR	Hypertensive, % (n/N): 100 (110/110); 100 (110/110)	NR	No	No	Unclear risk ^a^
Mu 2009 (participants with hypertension and family members)	NR	Serum creatinine above normal range	Hypertensive, % (n/N): 100 (101/101); 100 (114/114)	NR	No	Yes	Not at risk
Neal 2021	NR	Serious kidney disease (not defined)	Hypertensive (uncontrolled), % (n/N): 59.4 (6240/10505) , 59.2 (6211/10491)	Any antihypertension medication use ^g^, % (n/N): 79.9 (8393/10505), 78.7 (8256/10491); ACE inhibitor or ARB, % (n/N): 23.1 (2427/10505), 23.0 (2413/10491)	No	Yes	Possibly at risk
Omvik 1995	NR	Serum creatinine above normal range	Hypertensive, % (n/N): 100 (20/20); 100 (20/20)	No medication	No	No	Not at risk
Pan 2017	NR	GFR < 60ml/min	Hypertensive, %(n/N): 56.7 (55/97); 68.4 (62/95)	NR	No	Yes	Not at risk
Pereira 2005	Serum creatinine in µmol/L, mean (SD): 81.35 (8.84); 80.46 (8.84)	Kidney disease (not defined)	Hypertensive, % (n/N): 100 (28/28)	Chlorthalidone 25 mg, % (n/N): 68 (15/22); on hydrochlorothiazide 25 mg, % (n/N): 32 (7/22)	Use of other antihypertensives other than those specified.	Yes	Not at risk
Sarkkinen 2011	NR	Abnormal kidney function (not defined)	Hypertensive, % (n/N): 100 (25/25); 100 (25/25)	No medication	Yes	Yes (NSAIDs, cyclosporine, tacrolimus)	Not at risk
Suppa 1988	NR	Serum creatinine ≥ 1.5 mg/dL (133 µmol/L)	Hypertensive % (n/N): 100 (163/163); 100 (159/159)	Beta‐blocker monotherapy (metoprolol), % (n/N): 100 (163/163); 100 (159/159)	No	No	Not at risk
Toft 2020	NR	NR	Normotensive	No medication	Yes	No	Not at risk
Yu 2021	NR	History of acute or chronic kidney disease (CKD) ^g^	Hypertensive, % (n/N) 100 (252/252); 100 (250/250)	Antihypertensive medication use, % (n/N): 97.2 (245/252); 94.4 (236/250); ACE inhibitors or ARB, % (n/N): 27.8 (70/252); 32.0 (80/250)	No	Yes	Possibly at risk
Zhang 2015	Serum creatinine in µmol/L, mean (SD mean (SD): 69 (20.4); 69 (18.7)	NR	NR	NR	NR	NR	Unclear risk ^d^
Zhao 2014	NR	History of kidney disease	Hypertensive, % (n/N): 100 (141/141); 100 (141/141)	Antihypertensive use in the past month, % (n/N): 47.0 (61/141); 50.7 (71/141); average number of antihypertensive medicines taken, mean (SD): 0.4 (0.5); 0.5 (0.5)	No	No	Possibly at risk ^e^
Zhou 2009 (hypertensive participants)	Serum creatinine, in µmol/L, mean (SD): 78.5 (18.5); 76.8 (19.0)	Impaired renal function (not defined)	Hypertensive, % (n/N): 100 (62/62); 100 (64/64)	Antihypertensive medication use, % (n/N): 53.2 (33/62); 54.7 (35/64)	No	Yes	Not at risk
Zhou 2009 (normotensive participants)	Serum creatinine, in µmol/L, mean (SD): 75.6 (21.2); 77.5 (18.9)	Impaired renal function (not defined)	Normotensive, % (n/N): 100 (57/57); 100 (65/65)	No medication	No	Yes	Not at risk
Zhou 2013	NR	Significant renal impairment (not defined)	History of hypertension, % (n/N): 75 (169/224); 74 (176/238)	Captopril, nifedipine or compound reserpine, % (n/N): 41.07 (92/224); 40 (94/238)	No	Yes	Possibly at risk ^f^

Abbreviations:  ACE: angiotensin‐converting enzyme ARB: angiotensin receptor blocker CKD: chronic kidney disease GFR: glomerular filtration rate NR: not reported NSAID: non‐steroidal anti‐inflammatory drug SD: standard deviation  ^a ^ Medication use and renal function unclear ^b^ Even though participants on potassium‐sparing medications were excluded, serum creatinine cut‐off used could still indicate sub‐optimal kidney function and possible risk of hyperkalaemia ^c^ Baseline data not collected. Values reflect data collected during the endline survey ^d^ Medication use not reported ^e^ Some of participants judged to be at risk due to antihypertensive medication use ^f^ Use of captopril ^g^ From Levey 2013

##### Additionally, for adults:

- Age: adults younger than 65 years versus 65 years and older versus mixed ages versus unknown ages
- Body mass index (BMI): underweight (< 18.5 kg/m^2^) versus normal weight (18.5 to 24.9 kg/m^2^) versus overweight (25 to 29.9 kg/m^2^) versus obese (≥ 30 kg/m^2^) for non‐Asian adults, or underweight (< 18.5 kg/m^2^) versus normal weight (18.5 to 22.9 kg/m^2^) versus overweight (23 to 24.9 kg/m^2^) versus obese (≥ 25 kg/m^2^) for Asian adults (WHO 2000)

##### Additionally, for children:

- Age at start of study: 2 to 5 years versus 6 to 12 years versus 13 to 18 years versus mixed versus unknown

We also planned the following additional subgroup analyses, but available data did not allow these:

- Term of pregnancy at start of study: first trimester versus second trimester versus third trimester versus mixed versus unknown (in comparison of pregnant women);
- Conditions and risk factors: renal impairment versus other NCDs versus medication use that impair potassium excretion versus mixed versus unknown (in comparison of pregnant women).

#### Sensitivity analysis

We conducted sensitivity analyses for primary outcomes if we had three or more studies per meta‐analysis, assessing the impact of:

- Risk of bias: removing studies with a high risk of overall bias (see section: Assessment of risk of bias in included studies);
- Study design: removing cluster‐RCTs

#### Summary of findings and assessment of the certainty of the evidence

We used the GRADE approach to judge the certainty of the evidence as it relates to the studies contributing data to the meta‐analyses for the main outcomes, using GRADEprofiler (GRADEpro) software (GRADEpro GDT). The GRADE approach assesses certainty as high, moderate, low, or very low according to five criteria, namely, risk of bias, inconsistency of results, indirectness, imprecision and publication bias (Schünemann 2020). 

For the following outcomes, we presented assessments in a GRADE Evidence Profile for Comparisons 1 and 3 (i.e. per type of population included in this review): DBP, SBP, hypertension, blood pressure control, cardiovascular events, cardiovascular mortality, blood potassium, hyperkalaemia, hypokalaemia and adverse events (other). We could not compile GRADE Evidence Profiles for the comparison in pregnant women as no eligible studies reported on outcomes in pregnant women. The effects of interventions on the outcomes included in the GRADE Evidence Profiles were interpreted according to magnitude of effect and certainty of the evidence, using GRADE guidance on informative statements to combine size and certainty of an effect (Santesso 2020).

We used the approaches described below for each domain to guide our ratings and included explanations as footnotes in the GRADE Evidence Profiles.

##### Risk of bias

We considered downgrading if the majority (> 50%) of the weighted outcome data in a meta‐analysis were from studies at a high or unclear overall risk of bias.

##### Inconsistency

We considered downgrading due to either unexplained considerable (defined as I² ≥ 90%) or substantial heterogeneity (defined as I² between 50% to < 90%). We explored heterogeneity using prespecified subgroup analysis, and examined sensitivity analyses of study design and quality (overall risk of bias).

##### Indirectness

We considered downgrading based on population characteristics such as age, ethnicity, blood pressure status; or due to intervention characteristics (e.g. purpose of intervention, LSSS type), comparator, direct comparison and outcome.

##### Imprecision

###### Number of events or participants

We considered downgrading based on an insufficient number of events (i.e. 300 events) for dichotomous outcomes or sample size not meeting the optimal information size (OIS) (i.e. 400 people providing outcome measures) for continuous outcomes.

###### Minimally contextualised approach to GRADE ratings

In line with recent GRADE guidance (Hultcrantz 2017; Zeng 2021), we selected a minimally contextualised approach that required us to specify thresholds for minimally important differences for key outcomes. The upper and lower limits of the 95% CIs were assessed in the same way to determine if they included the possibility of a small, trivial or no effect and an important benefit or harm (Zeng 2021).

Applying this approach to rating the certainty of evidence using GRADE in relation to thresholds (other than no effect) ideally requires the use of absolute numbers (Zeng 2021). To further support decision‐making by the WHO NUGAG Subgroup on Diet and Health about a population‐level intervention, we generated estimated population impacts for effect estimates and variation (95% CIs) for key clinical effectiveness outcomes when LSSS use was compared to regular salt use (Verbeek 2021). This was applied to the following key clinical effectiveness outcomes in adults: change in DBP, change in SBP, cardiovascular events: non‐fatal stroke, cardiovascular events: non‐fatal acute coronary syndrome, cardiovascular mortality and stroke mortality. We used a simplified model to estimate absolute numbers from relative cardiovascular measures, as well as the absolute numbers of stroke deaths prevented or caused by changes in blood pressure, as a surrogate outcome (Verbeek 2021). More detail on this simplified modelling approach can be found in Appendix 2. 

## Results

### Description of studies

For detailed information, see Characteristics of included studies; Characteristics of excluded studies; Characteristics of ongoing studies; Characteristics of studies awaiting classification.

#### Results of the search

The study selection flowchart is available in Figure 1. We screened the titles and abstracts of 6511 de‐duplicated records identified through searching electronic databases, as well as 14 records identified through handsearching of three relevant systematic reviews. We assessed the full texts of 161 records against our eligibility criteria, of which four were in Chinese and one in Portuguese; we obtained language translation assistance for assessment of these. We included 26 studies reported in 74 full‐text records (Included studies), of which one did not provide data that could be used in the quantitative syntheses (meta‐analyses) (Arzilli 1986). Eight studies were identified as ongoing. We placed three studies under awaiting classification because we were unable to obtain further study details or data from the study authors in order to assess their eligibility for inclusion. We excluded a total of 75 full‐text records, of which 42 were duplicates (Excluded studies).

**1:**
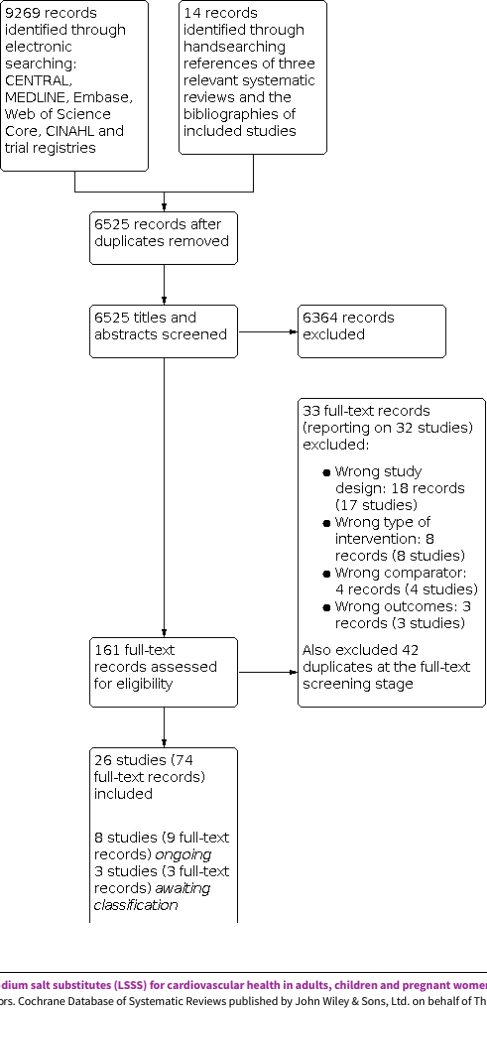
PRISMA flow diagram

#### Included studies

##### Study designs

We included 26 eligible RCTs and did not identify any eligible prospective analytical cohort studies. The details of the included studies are summarised in Characteristics of included studies. Of the included trials, 16 were individually randomised trials (Allaert 2013; Allaert 2017; Arzilli 1986; CSSS Collaborative Group 2007; Geleijnse 1994; Gilleran 1996; Kawasaki 1998; Mu 2003; Omvik 1995; Pan 2017; Pereira 2005; Sarkkinen 2011; Suppa 1988; Yu 2021; Zhao 2014; Zhou 2009) and 10 were cluster‐RCTs (Bernabe‐Ortiz 2014; Chang 2006; Hu 2018; Li 2014; Li 2016; Mu 2009; Neal 2021; Toft 2020; Zhang 2015; Zhou 2013), including one stepped‐wedge cluster‐RCT (Bernabe‐Ortiz 2014). One RCT reported a cross‐over design (Allaert 2013) for which we only used first‐phase data. Twenty‐five of the eligible trials were published in peer‐reviewed journals; one cluster‐RCT was published in three separate conference abstracts and one trial as an abstract in a journal supplement. Full‐text publications of these could not be sourced after numerous attempts to contact authors.

##### Sample sizes and follow‐up

Nine of the 16 RCTs randomised ≤ 100 participants while seven were larger, randomising > 100 participants up to a maximum of 608 participants (CSSS Collaborative Group 2007; Mu 2003; Pan 2017; Suppa 1988; Yu 2021; Zhao 2014; Zhou 2009). Fewer than half of these RCTs (n = 7) reported sample size calculations, based on expected changes of between 3 and 10 mmHg in SBP (Allaert 2013; Allaert 2017; CSSS Collaborative Group 2007; Geleijnse 1994; Yu 2021; Zhao 2014; Zhou 2009); of these, two trials additionally based sample size calculations on expected changes of between 1.7 and 4 mmHg in DBP (CSSS Collaborative Group 2007; Geleijnse 1994).

Of the cluster‐RCTs, five randomised families or households (Hu 2018; Li 2014; Mu 2009; Toft 2020; Zhou 2013), ranging between 89 and 325 households, including 309 to 659 individual participants. Three cluster‐RCTs randomised villages; one conducted in Peru (N = 6 clusters; 2376 participants; Bernabe‐Ortiz 2014) and two conducted in China (N = 120 clusters; 2566 participants; Li 2016 and N = 600; 20,995 participants; Neal 2021). The cluster‐RCT by Zhang 2015 randomised nursing homes (N = 30); another randomised kitchens within a retirement home (N = 5; 2764 participants) (Chang 2006). Of the ten cluster‐RCTs, only four reported appropriate sample size calculations (including an ICC) based on expected changes in blood pressure, 24‐h sodium excretion, relative reduction in stroke and sodium intake reduction (Bernabe‐Ortiz 2014; Li 2016; Neal 2021; Toft 2020, respectively); two cluster‐RCTs (Chang 2006; Mu 2009) did not report a sample size calculation but did adjust for the effect of clustering in their analyses. One cluster‐RCT (Hu 2018) reported a sample size calculation based on an expected change in SBP, but did not report incorporating an ICC in this calculation. 

Eleven (Allaert 2013; Allaert 2017; Arzilli 1986; CSSS Collaborative Group 2007; Gilleran 1996; Kawasaki 1998; Mu 2003; Omvik 1995; Sarkkinen 2011; Suppa 1988; Zhou 2009) of the 16 RCTs included a run‐in period, ranging between five days and six weeks. Ten RCTs tested an active LSSS intervention for a period of up to three months (Allaert 2013; Allaert 2017; Arzilli 1986; Geleijnse 1994; Kawasaki 1998; Pereira 2005; Sarkkinen 2011; Suppa 1988; Yu 2021; Zhao 2014), and five for between three and 12 months (CSSS Collaborative Group 2007; Gilleran 1996; Omvik 1995; Pan 2017; Zhou 2009). One RCT implemented a LSSS intervention for longer than 12 months (Mu 2003). For the 10 cluster‐RCTs, the duration of the LSSS intervention was two months in one trial (Li 2014), four months in another (Toft 2020), and ranged between one and five years in the other eight cluster‐RCTs. 

##### Settings

Four of the 16 RCTs were conducted in northern China or Tibet; most were done in rural or suburban households (CSSS Collaborative Group 2007; Mu 2003; Zhao 2014; Zhou 2009). The remaining individually randomised trials from Brazil (Pereira 2005), France (Allaert 2013; Allaert 2017), Finland (Sarkkinen 2011), India (Yu 2021); Italy (Suppa 1988), Japan (Kawasaki 1998), Netherlands (Geleijnse 1994), Norway (Omvik 1995), Taiwan (Pan 2017) and the UK (Gilleran 1996) were conducted at household level, except for one trial in a European hospital setting (Arzilli 1986). Seven cluster‐RCTs were conducted in northern China or Tibet; most were done in rural or suburban households or communities (Hu 2018; Li 2014; Li 2016; Mu 2009; Neal 2021; Zhou 2013), with one cluster‐RCT having been conducted in nursing homes (Zhang 2015). One included cluster‐RCT from Taiwan (Chang 2006) was also conducted in a nursing home setting. In Peru, one stepped‐wedge cluster‐RCT was conducted in rural villages and households (Bernabe‐Ortiz 2014); a cluster‐RCT in Southwestern Denmark was conducted in families (Toft 2020).  

##### Participants

We did not find any eligible studies in pregnant women. Trial participants were adults with a mean age ranging from 20 to 75.21 years and children with a mean age ranging from 8.4 to 9.5 years. Most of the included trials (15/26) were conducted in populations living in Asian countries. Eleven studies specifically included only participants with hypertension (Allaert 2013; Arzilli 1986; Geleijnse 1994; Gilleran 1996; Mu 2003; Omvik 1995; Pereira 2005; Sarkkinen 2011; Suppa 1988; Yu 2021; Zhao 2014), 11 included participants with and without hypertension (Bernabe‐Ortiz 2014; Chang 2006; CSSS Collaborative Group 2007; Hu 2018; Kawasaki 1998; Li 2014; Mu 2009; Neal 2021; Pan 2017; Zhou 2009; Zhou 2013), one each included only participants with normal blood pressure (Toft 2020) and participants who were pre‐hypertensive (Allaert 2017), and blood pressure status at baseline in the remaining studies were unknown (Li 2016; Zhang 2015). The largest trial (Neal 2021) included participants with an elevated risk of stroke and approximately 70% of participants in intervention and control groups had a history of stroke at baseline. Fifteen studies reported outcome data separately in participants with hypertension (Allaert 2013; Arzilli 1986; Geleijnse 1994; Gilleran 1996; Hu 2018; Kawasaki 1998; Mu 2003; Mu 2009; Omvik 1995; Pereira 2005; Sarkkinen 2011; Suppa 1988; Yu 2021; Zhao 2014; Zhou 2009). Twelve of these studies specifically included only participants with hypertension. Three of these studies included participants with hypertension and their family members (Hu 2018; Mu 2009; Zhou 2009) and one study included clinically healthy middle‐aged and elderly volunteers (Kawasaki 1998); these studies additionally reported outcome data in participants with normal blood pressure separately.

One cluster‐RCT compared LSSS to regular salt in 92 children (numbers analysed) (Toft 2020) by randomising families to LSSS or regular salt in bread. Seven studies included participants possibly at risk of hyperkalaemia (Chang 2006; Geleijnse 1994; Hu 2018; Neal 2021; Yu 2021; Zhao 2014; Zhou 2013), four studies included participants at unclear risk of hyperkalaemia (Arzilli 1986; Li 2016; Mu 2003; Zhang 2015) and the remaining trials included participants considered not to be at risk of hyperkalaemia. The criteria and assessments applied to classify the hyperkalaemia risk of participants per included study are summarised in Table 6. All 26 included trials excluded participants in whom an increased intake of potassium is known to be potentially harmful, for example, people with chronic kidney disease, type 1 or 2 diabetes mellitus, impaired renal function, or those using potassium‐sparing medications. 

##### Interventions

In 23 of the 26 studies, combinations of potassium and/or magnesium and/or calcium salts were used as sodium substitutes in the LSSS interventions, with two studies assessing a LSSS intervention consisting of NaCl combined with 3% chitosan (Allaert 2013; Allaert 2017) and the remaining study assessing a LSSS intervention ‘naturally low in sodium’ in bread (Toft 2020). Product characteristics of the latter, obtained through author correspondence (Toft 2020), showed that the compound contained trace amounts of potassium (approximately 0.1 to 0.2%). Two RCTs (Arzilli 1986; Mu 2003) and one cluster‐RCT (Mu 2009) assessed LSSS with an unknown KCl content. Four cluster‐RCTs (Chang 2006; Li 2014; Neal 2021; Zhang 2015) and six RCTs (Geleijnse 1994; Gilleran 1996; Pan 2017; Pereira 2005; Yu 2021; Zhou 2009) assessed the effects of a LSSS intervention containing ≥ 30% KCl, while the remainder of the trials used LSSS interventions containing < 30% KCl. One RCT included two LSSS intervention arms, both including ≥ 30% KCl (Pan 2017).

Most trials (22/26) administered the LSSS intervention as a discretionary intervention (at the individual, household, institution or salt supply chain level). Of these, most trials replaced the supply of regular salt with LSSS within each household, to be used at the table and during food preparation (Bernabe‐Ortiz 2014; CSSS Collaborative Group 2007; Gilleran 1996; Hu 2018; Li 2014; Li 2016; Mu 2003; Mu 2009; Neal 2021; Omvik 1995; Pan 2017; Pereira 2005; Yu 2021; Zhao 2014; Zhou 2009; Zhou 2013). Two trials were conducted in nursing homes where LSSS was used during food preparation in the intervention kitchens of one trial (Chang 2006), while the specific implementation of the LSSS was unclear in the other trial (Zhang 2015). LSSS was administered as ‘added salt’ in four trials (Allaert 2013; Allaert 2017; Arzilli 1986; Suppa 1988). 

A cluster‐RCT from Peru replaced the supply of regular salt in each village with LSSS in the salt supply chain, including households, food vendors, bakeries, community kitchens and restaurants. A social marketing/education strategy promoting LSSS in each village was aimed at women who were responsible for household food preparation (Bernabe‐Ortiz 2014). Another cluster‐RCT from northern China provided LSSS via the local food supply chain. LSSS was available for purchase at local village shops at either a subsidised price (same as regular salt) in half of the intervention villages, or at a regular price (approximately double that of regular salt). A community‐based health education programme to promote the use of LSSS was implemented via public announcement systems, bulletin boards, and specially developed promotional materials (Li 2016).

Three RCTs incorporated LSSS into prepared test foods, such as processed main dishes, bread, cheese, luncheon meats, soups or smoked sausage (Geleijnse 1994; Sarkkinen 2011), or seasonings containing LSSS, such as miso and soy sauce (Kawasaki 1998). These trials also provided trial participants with LSSS as salt for household food preparation and for use as table salt. A fourth cluster‐RCT used bread as the exclusive method of LSSS implementation by incrementally replacing normal salt with LSSS in bread over a period of five to six weeks, with participants followed up for four months in total (Toft 2020).

Trial participants in four studies were instructed not to change their dietary habits during the study period (Allaert 2017; Geleijnse 1994; Hu 2018; Kawasaki 1998), whereas participants from two trials were advised to either reduce their salt intake (Omvik 1995), or avoid salt‐rich foods (Sarkkinen 2011). Two trials reported co‐interventions such as lifestyle advice about eating less fat and sugar and doing more physical exercise (Allaert 2013), or a hypocaloric diet with increased physical exercise (Pereira 2005).

##### Outcome measures

Outcomes were regarded as clinical effectiveness outcomes, or safety outcomes related to the intake of LSSS with potassium (as guided by the WHO NUGAG Subgroup on Diet and Health).

Clinical effectiveness outcomes for comparisons in both adults and children were change in DBP, change in SBP, hypertension, blood pressure control, cardiovascular events, cardiovascular mortality, all‐cause mortality, antihypertensive medication use, change in fasting blood glucose, change in blood triglycerides, change in total blood cholesterol, and change in 24‐h urinary sodium and potassium excretion. In addition, clinical effectiveness outcomes in adults only were diabetes mellitus diagnosis and change in BMI; and in children only were growth changes, bone densitometry and bone health.

Safety outcomes for comparisons in both adults and children were change in blood potassium, hyperkalaemia, hypokalaemia, adverse events, renal function and hyponatraemia.

###### Primary outcomes

Only one RCT (Pan 2017) and one cluster‐RCT (Chang 2006) did not report on changes in DBP and SBP. However, we were also unable to use relevant outcome data from four trials, due to blood pressure data being reported in a figure (CSSS Collaborative Group 2007 (DBP only); Kawasaki 1998 (DBP and SBP)) or study authors providing insufficient information on the number of participants per treatment group (Arzilli 1986; Mu 2009). Three RCTs reported two types of DBP and SBP outcome data, i.e. ambulatory and clinic BP measurements (Allaert 2017; Omvik 1995; Pereira 2005). In order to minimise potential heterogeneity between studies, we included only the clinic BP measurements from these trials in our meta‐analyses.

Two cluster‐RCTs reported on the outcome hypertension; defined as SBP ≥ 140 mmHg, DBP ≥ 90 mmHg, or the use of blood‐pressure lowering therapy in the last two weeks (Li 2016) or SBP ≥ 140 mmHg, DBP ≥ 90 mmHg, a self‐reported physician diagnosis or current treatment for hypertension (Bernabe‐Ortiz 2014). Two RCTs reported on blood pressure control, defined as achieving SBP ≤ 140 mmHg and DBP ≤ 90 mmHg in both trials (Allaert 2013; Zhao 2014).

For the outcome cardiovascular events, we extracted data related to events such as stroke (Gilleran 1996; Hu 2018; Neal 2021; Pan 2017; Zhao 2014), myocardial infarction (Hu 2018) or acute coronary syndrome (Neal 2021), coronary heart disease or heart failure (Li 2016), hypotension (Li 2016), angina (Allaert 2017; Omvik 1995), bradycardia (Suppa 1988), as well as composite outcomes such as cardiovascular events (CSSS Collaborative Group 2007; Zhou 2009) or cardiovascular symptoms (Sarkkinen 2011). Three trials reported insufficient data on the number of events per group (Li 2016; Suppa 1988) as described in Table 7. Cardiovascular mortality was reported by one RCT (Zhao 2014) and three cluster‐RCTs (Chang 2006; Neal 2021; Zhou 2013); stroke mortality was reported by two cluster‐RCTs (Neal 2021; Zhou 2013).

**5:** Trials without usable data for outcomes in Summary of Findings tables in Comparison 1

**Outcomes included in Summary of Findings tables**	**Included studies that are believed to have measured the outcome, but did not report it in a usable format**
Change in DBP (mmHg)	Arzilli 1986: only between‐group P values reported CSSS Collaborative Group 2007: reported in a figure Kawasaki 1998: reported mean change in intervention group, but not control group Mu 2009: reported only mean change with no SDs or participant numbers
Change in SBP (mmHg)	Arzilli 1986: only between‐group P values reported Kawasaki 1998: reported mean change in intervention group, but not control group Mu 2009: reported only mean change with no SDs or participant numbers
Hypertension	None
Blood pressure control	None
Cardiovascular events: various	Li 2016: reported non‐significant difference between groups (not exact P value), mean difference not reported Suppa 1988: numbers of events per group not reported
Cardiovascular events: non‐fatal stroke	None
Cardiovascular events: non‐fatal ACS	None
Cardiovascular mortality	None
Stroke mortality	None
Change in blood potassium (mmol/L)	Sarkkinen 2011: reported change and significance of change in control group only
Hyperkalaemia	Li 2016: reported non‐significant difference between groups (not exact P value), mean difference not reported
Hypokalaemia	None
Adverse events: other	Suppa 1988: numbers of events per group not reported

Abbreviations:  ACS: acute coronary syndrome DBP: diastolic blood pressure SBP: systolic blood pressure SD: standard deviation

Six trials reported on hyperkalaemia events; two trials (CSSS Collaborative Group 2007; Mu 2009) did not explicitly define criteria for hyperkalaemia, and one assessed self‐reported hyperkalaemia without defined criteria (Li 2016). Yu 2021 and Zhang 2015 defined the outcome as a serum potassium level more than 6.5 mmol/L and 5.5 mmol/L, respectively. Neal 2021 reported on definite, probable, possible and unlikely hyperkalaemia ‐ only data on definite and probable events, defined as elevated serum potassium > 5.5 mmol/L and typical electrocardiogram (ECG) changes documented in medical notes, were extracted; events that were possible (self‐reported serum potassium > 5.5 mmol/L or ECG changes but no supporting documentation to verify) and unlikely (clinical history and documentation suggest minimal indication for the diagnosis) were excluded from our review. Insufficient information was available from one trial (Li 2016) as described in Table 7. Only one RCT reported on hypokalaemia events (Pereira 2005), though criteria for the condition were not explicitly defined.

Seven trials reported changes in serum (Allaert 2017; Geleijnse 1994; Kawasaki 1998; Pereira 2005; Zhang 2015) or plasma concentrations of potassium (Omvik 1995; Sarkkinen 2011) between intervention and control groups. Data could not be extracted from one study (Sarkkinen 2011), as described in Table 7. 

###### Secondary outcomes

Four RCTs (CSSS Collaborative Group 2007; Pan 2017; Zhao 2014; Zhou 2013), and three cluster‐RCTs (Chang 2006; Neal 2021; Zhang 2015) reported on all‐cause mortality.  

Three trials reported on the occurrence of serious adverse events (not defined) during the study period (Bernabe‐Ortiz 2014; CSSS Collaborative Group 2007; Pan 2017). Other adverse event outcomes extracted included gastrointestinal symptoms (stomach ache or abdominal distension) (Sarkkinen 2011; Zhao 2014), hypercalcemia and renal calculi (Mu 2009), appendicitis, nephritis, nephrosis (Hu 2018), influenza (Allaert 2017), respiratory symptoms (Sarkkinen 2011) and dorsalgia (Allaert 2017). Suppa 1988 included self‐reported adverse events including asthenia, bradycardia, drowsiness, insomnia, decreased libido and depression, but did not report the number of events per group. None of the included trials reported adverse events such as nausea or vomiting.

Five trials reported on changes in antihypertensive medication use (Bernabe‐Ortiz 2014; Hu 2018; Li 2016; Zhao 2014; Zhou 2013). Four trials reported the number of participants using antihypertensive medications: two trials reported on this outcome in participants with hypertension (Hu 2018 included participants with hypertension and their family members, but only reported on the former; Zhao 2014 included only participants with hypertension) and one trial each in participants with unknown hypertensive status (Li 2016) and participants using hypotensive medication at baseline (Zhou 2013). Bernabe‐Ortiz 2014 assessed participant‐reported changes in medication use for hypertension and type 2 diabetes mellitus combined.

Three trials reported changes in serum creatinine (Omvik 1995; Pereira 2005; Zhang 2015). One cluster‐RCT (Li 2016) reported the mean urinary albumin‐to‐creatinine ratios of participants in the intervention and control groups, as well as the proportion of participants with albuminuria (including micro‐ and macro‐albuminuria) in both groups.

Four studies reported changes in BMI (Li 2016; Pereira 2005; Sarkkinen 2011; Toft 2020), while two reported changes in fasting blood glucose concentrations (Toft 2020; Zhou 2013). Five studies reported changes in blood triglycerides (Gilleran 1996; Kawasaki 1998; Pereira 2005; Toft 2020; Zhou 2009) and total blood cholesterol (Geleijnse 1994; Gilleran 1996; Kawasaki 1998; Toft 2020; Zhou 2009), respectively.

A total of 12 studies collected 24‐h urine samples and reported on 24‐h urinary sodium excretion (Bernabe‐Ortiz 2014; Geleijnse 1994; Gilleran 1996; Kawasaki 1998; Li 2016; Neal 2021; Omvik 1995; Sarkkinen 2011; Suppa 1988; Toft 2020; Yu 2021; Zhou 2009); the same studies reported on 24‐h urinary potassium excretion from 24‐h urine samples. 

None of the included studies reported on the outcomes of diabetes mellitus diagnosis or hyponatremia.

##### Funding sources and conflicts of interest

Nine of the 26 studies did not disclose their funding source(s). The remaining studies were funded as follows:

- nine public/non‐commercial funding only, including government bodies and research institutions (Bernabe‐Ortiz 2014; CSSS Collaborative Group 2007; Li 2014; Li 2016; Mu 2009; Pan 2017; Toft 2020; Zhao 2014; Zhou 2013);
- four public/non‐commercial funding plus LSSS provided for the trial by LSSS manufacturer (Geleijnse 1994; Neal 2021; Omvik 1995; Yu 2021);
- two commercial funding by LSSS manufacturers plus LSSS provided for the trial (Chang 2006; Sarkkinen 2011);
- one commercial funding for the study from food industry research fund plus LSSS provided for the trial by LSSS manufacturer (Hu 2018); and
- one LSSS provided for the trial by the LSSS manufacturer (Kawasaki 1998).

The authors of 13 included studies did not report on potential conflicts of interest (COI), whereas those from 13 studies did. Of these 13 studies, authors of eleven declared that they had no potential COI (Bernabe‐Ortiz 2014; Chang 2006; CSSS Collaborative Group 2007; Mu 2009; Pan 2017; Pereira 2005; Toft 2020; Yu 2021; Zhao 2014; Zhou 2009; Zhou 2013), whereas the author of one study declared a potential conflict of interest as the chair of the Australian Division of World Action on Salt and Health (Li 2016), and some members of the author team of a large cluster‐RCT declared potential conflicts of interest, while the remaining members declared no conflict (Neal 2021).

#### Excluded studies

We contacted nine corresponding authors for further information to assist with study inclusion. We excluded 32 studies (33 full‐text records) due to the following reasons:** **

Wrong study design (single‐arm trial): 5 Wrong study design (commentary/letter): 3 Wrong study design (case report/study): 2 Wrong study design (case series): 2 Wrong study design (non‐randomised trial): 2 Wrong study design (quasi‐randomised trial): 2 Wrong study design (cross‐over with first phase data not available): 1 Wrong type of intervention (multifactorial): 4 Wrong type of intervention (dietary): 2 Wrong type of intervention (LSSS administered as supplement): 1 Wrong type of intervention (salt restriction education): 1 Wrong comparator: 4 Wrong outcome (sensory/organoleptic): 2 Wrong outcome (sodium concentration of homemade food): 1

The Characteristics of excluded studies section illustrates these 32 studies with reasons for exclusion. We also excluded 42 duplicates at the full‐text screening stage. The remaining references where we could not reach the authors or information provided was not sufficient to make a clear judgement (n = 3) were included as Studies awaiting classification. The eight ongoing studies are detailed in Characteristics of ongoing studies.

### Risk of bias in included studies

The Characteristics of included studies provides details of the judgements for each risk of bias domain per study. Figure 2 presents a summary of the risk of bias judgements for each included study and Figure 3 the summary of the judgements per risk of bias domain.

**2:**
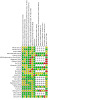
Summary of the risk of bias judgements for each included study

**3:**
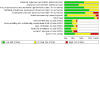
Summary of the judgements per risk of bias domain

#### Allocation

##### Random sequence generation

Fifteen trials described adequate methods of random sequence generation and were at low risk of selection bias (Bernabe‐Ortiz 2014; Chang 2006; CSSS Collaborative Group 2007; Geleijnse 1994; Hu 2018; Li 2014; Li 2016; Mu 2009; Neal 2021; Pan 2017; Toft 2020; Yu 2021; Zhao 2014; Zhou 2009; Zhou 2013). Eleven trials did not report how the random sequence had been generated and were at unclear risk of selection bias. 

##### Allocation concealment

Eleven trials described methods of allocation concealment judged to be at low risk of selection bias (Allaert 2017; Bernabe‐Ortiz 2014; CSSS Collaborative Group 2007; Hu 2018; Li 2016; Neal 2021; Pan 2017; Toft 2020; Yu 2021; Zhao 2014; Zhou 2009). The remaining fifteen did not report sufficient information on allocation concealment, and were at unclear risk of selection bias.

#### Blinding

##### Blinding of participants and personnel (performance bias)

Performance bias was assessed as unlikely in fifteen trials, while eleven trials had an unclear risk of bias, mainly due to insufficient information on blinding of either participants or personnel (Allaert 2013; Arzilli 1986; Bernabe‐Ortiz 2014; Chang 2006; Kawasaki 1998; Li 2014; Li 2016; Mu 2003; Mu 2009; Suppa 1988; Zhang 2015). 

##### Blinding of outcome assessment (detection bias)

Nineteen trials were assessed to have a low risk of detection bias, while seven had an unclear risk mainly due to insufficient information on blinding of outcome assessors (Li 2016; Mu 2003; Mu 2009; Pereira 2005; Suppa 1988; Zhang 2015; Zhou 2013). Some of these trials reported the measurement of blood pressure with non‐automatic devices, increasing the likelihood of detection bias (Mu 2003; Pereira 2005; Suppa 1988).

#### Incomplete outcome data

Four trials were at a high risk of bias due to high overall or differential attrition (≥ 10%) (Geleijnse 1994; Gilleran 1996; Hu 2018; Mu 2003). Fourteen studies were at low risk because they reported low overall or differential attrition (Allaert 2013; Allaert 2017; Chang 2006; CSSS Collaborative Group 2007; Kawasaki 1998; Li 2014; Neal 2021; Omvik 1995; Sarkkinen 2011; Suppa 1988; Toft 2020; Yu 2021; Zhou 2009; Zhou 2013). Two studies reported high attrition; however intention‐to‐treat analyses (ITT) were conducted using multiple imputation in one study (Zhao 2014) and the last‐observation‐carried‐forward method in the other study (Pan 2017); therefore, these were judged as being at low risk. High attrition was reported at some time points in the stepped‐wedge cluster‐RCT; however, it was unclear whether any data were imputed and therefore this study had an unclear risk (Bernabe‐Ortiz 2014). Five additional studies were at unclear risk of bias since insufficient information on attrition was provided.

#### Selective reporting

Two trials were assessed as being at high risk of bias.  Substudies of both trials reported outcomes not prespecified in the study protocol (CSSS Collaborative Group 2007; Li 2016). Two trials at low risk of bias reported outcomes prespecified in the study protocol; the remaining studies had an unclear risk due to inadequate reporting of all prespecified outcomes, or the unavailability of the study protocol.

#### Other potential sources of bias

One trial was assessed as being high risk for misclassification bias due to limited information for adjudication of clinical outcome events, as well as self‐reported potential hyperkalaemia events (Neal 2021). Two trials were assessed as being at unclear risk. In the stepped‐wedge trial, a reduction in blood pressure was observed in some clusters (villages) before the intervention (Bernabe‐Ortiz 2014) while, in another cluster‐RCT, there was a considerable risk of contamination in the intervention group due to the unlimited availability of condiments and spices such as soy sauce and monosodium glutamate (Chang 2006). No potential sources of bias were identified in the remaining studies.

##### Additional domains assessed for cluster‐RCTs

###### Recruitment bias

One trial had a high risk for recruitment bias since a number of participants were recruited after the clusters (kitchens) were randomised (Chang 2006).  Six trials reported recruitment of participants before randomisation of clusters and were therefore considered at low risk (Bernabe‐Ortiz 2014; Hu 2018; Li 2014; Li 2016; Neal 2021; Toft 2020). Three trials did not provide sufficient information regarding the timing of recruitment.

###### Comparability with individually randomised trials (RCTs)

Six trials at low risk of bias reported effect estimates comparable to those reported by similar RCTs (Bernabe‐Ortiz 2014; Chang 2006; Hu 2018; Li 2014; Zhang 2015; Zhou 2013), whereas it was not possible to compare the effect estimates in four trials.

###### Baseline imbalance

Three trials reported no differences in baseline characteristics of the participants in the intervention and control groups (Hu 2018; Li 2014; Neal 2021), while two adjusted for baseline differences and were therefore also considered to be at low risk (Bernabe‐Ortiz 2014; Zhou 2013). Five studies that reported insufficient information regarding baseline characteristics were at unclear risk of bias.

###### Loss of clusters

One trial reported an analysis without outcome data from almost one‐third of their included clusters, thus was considered at high risk of attrition bias (Zhang 2015). Seven trials had a low risk (Bernabe‐Ortiz 2014; Chang 2006 ; Hu 2018; Li 2014; Li 2016; Neal 2021; Toft 2020) while two did not provide sufficient information on the number of clusters (families) lost to follow‐up (Mu 2009), or the reasons for loss to follow‐up (Zhou 2013).

###### Incorrect analysis

Three trials did not report adjustment for clustering in their analysis, thus were considered at high risk of bias (Hu 2018; Li 2014; Zhou 2013). Six trials were at low risk; five reported statistical adjustment for clustering (Chang 2006; Li 2016; Mu 2009; Neal 2021; Toft 2020) and one also accounted for time trends in their analysis (Bernabe‐Ortiz 2014). One trial that reported insufficient information regarding statistical adjustment for clusters was considered at unclear risk.

##### Overall risk of bias

Three RCTs and three cluster‐RCTs had a high overall risk of bias due to attrition (Geleijnse 1994; Gilleran 1996; Hu 2018; Mu 2003; Zhang 2015) or recruitment bias (Chang 2006). Six RCTs had a low overall risk (Allaert 2017; CSSS Collaborative Group 2007; Pan 2017; Yu 2021; Zhao 2014; Zhou 2009) and two cluster‐RCTs (Li 2014; Neal 2021) had a low overall risk, while the remaining seven RCTs and five cluster‐RCTs had an unclear risk mainly due to uncertainty about selection, attrition and recruitment bias (Allaert 2013; Arzilli 1986; Bernabe‐Ortiz 2014; Kawasaki 1998; Li 2016; Mu 2009; Omvik 1995; Pereira 2005; Sarkkinen 2011; Suppa 1988; Toft 2020; Zhou 2013).

### Effects of interventions

See: Table 1; Table 2

See: Table 1; Table 2.

#### Comparison 1. Low‐sodium salt substitutes versus regular salt or no active intervention in adults

Table 1 presents the effects of LSSS compared to regular salt or no active intervention in adult participants on changes in DBP and SBP; as well as the number of participants per group with hypertension, blood pressure control, cardiovascular events (various events and non‐fatal stroke); the rate ratio of participants in the intervention group with non‐fatal acute coronary syndrome (ACS), cardiovascular mortality and stroke mortality; changes in blood potassium; and the number of participants per group with hyperkalaemia, hypokalaemia and other adverse events.

A total of 26 RCTs, 16 randomising individual participants and 10 randomising clusters, reporting on 34,961 adult participants, were included in this comparison. Key details about studies in this comparison, including study design, setting and overall risk of bias; characteristics of the intervention, comparator, population and outcomes; method of synthesis, and time points of measurement are included in the Overview of Synthesis and Included Studies (OSIS) table (Table 8).

**6:** Comparison 1: Overview of Synthesis and Included Studies (OSIS)

**Study name (year)**	**Study design** (individual vs stepped‐wedge vs cluster‐randomised [and unit of randomisation, if applicable])	**Country of conduct**	**Overall risk of bias** (arranged low to high)	**Key details of intervention** (% KCl, LSSS (implementation [discretionary, non‐discretionary, both], quantity, provided or purchased, co‐interventions [education, advice])	**Key details of the comparator** (implementation [discretionary, non‐discretionary, both], quantity, provided or household supply, co‐interventions [education, advice])	**Population** (No. of participants randomised [intervention/control], age, gender, hypertensive status, antihypertensive medication use, BMI)	**Outcome domains with available data** (synthesis method/metric)	**Specific outcomes measure**	**Time point of measurement**
Allaert 2017	Individually randomised	France	Low	No KCl content in LSSS (97% NaCl and 3% chitosan) Discretionary use to a maximum of 3 g per day 300 g provided Participants not to change their dietary, physical activity, smoking habits during study period	Regular salt Discretionary use to a maximum of 3 g per day 300 g provided Participants not to change their dietary, physical activity, smoking habits during study period	22/19 51 (16) years Male, %: 51.2 Pre‐hypertensive None NR	Change in blood pressure Cardiovascular events Change in blood potassium Adverse events	1. Change in DBP 2. Change in SBP 3. Various other cardiovascular events 4. Change in blood potassium 5. Various other adverse events	1. 56 days 2. 56 days 3. ≤ 3 months 4. 56 days 5. ≤ 3 months
CSSS Collaborative Group 2007	Individually randomised	China	Low	25% KCl LSSS (with 65% NaCl and 10% MgSO_4_) Discretionary use Up to 3 kg per month provided for household use Co‐interventions NR	Regular salt Discretionary use Up to 3 kg per month provided for household use Co‐interventions NR	306/302 59 (10)/61 (9.7) years Female, %: 54/58 Hypertensive, %: 57/57 Use of any antihypertensive medication, %*:* 61/61 26 (3.6)/25(3.9) kg/m^2^	Change in blood pressure Cardiovascular events Hyperkalaemia Mortality Adverse events	1. Change in DBP 2. Change in SBP 3. Various other cardiovascular events 4. Hyperkalaemia 5. All‐cause mortality 6. Various other adverse events	1. See Table 7 2. 12 months 3. > 3 to 12 months 4. 12 months 5. > 3 to 12 months 6. > 3 to 12 months
Li 2014	Cluster‐randomised (households)	China	Low	30% KCl LSSS (KCl 30.0 ± 10.0 %; NaCl 70.0 ± 10.0%;) Discretionary use 350 g provided (frequency NR) Co‐interventions NR	Regular salt Discretionary use Own household supply Co‐interventions NR	253/263 59.3 (11.7)/59.2 (8.7) years Female, %: 50.9/48.7 NR NR NR	Change in blood pressure	1. Change in DBP 2. Change in SBP	1. 2 months 2. 2 months
Neal 2021	Cluster‐randomised (villages)	China	Low	30 ± 10% KCl (with 70 ± 10% NaCl ) Discretionary use at average intake of 20 g per person per day Provided regular supply of LSSS to households (up to 20 kg per year for a household with 3 members) Co‐interventions NR	Regular salt Discretionary use Own household supply Advice about reducing salt intake given at study commencement.	10504/10491 65.2 (8.5)/ 65.5 (8.5) years Female, %: 49.7 Hypertensive, %: 59.4/59.2 Any antihypertensive medication, %: 79.9/78.7 24.8 (3.6)/24.9 (3.7) kg/m^2^	Change in blood pressure Cardiovascular events Mortality Hyperkalaemia 24‐h urinary excretion	1. Change in DBP 2. Change in SBP 3. Cardiovascular events: non‐fatal stroke 4. Cardiovascular events: non‐fatal acute coronary syndrome 5. Cardiovascular mortality 6. Stroke mortality 7. All‐cause mortality 8. Hyperkalaemia 9. 24‐h urinary sodium excretion 10. 24‐h urinary potassium excretion	1. 60 months 2. 60 months 3. 4.75 years mean follow‐up 4. 4.75 years mean follow‐up 5. 4.75 years mean follow‐up 6. 4.75 years mean follow‐up 7. 4.75 years mean follow‐up 8. 4.75 years mean follow‐up 9. 60 months 10. 60 months
Pan 2017	Individually randomised	Taiwan	Low	Two intervention arms: 50% KCl LSSS (with NaCl 50%) or 42.85% KCl LSSS, (with 42.85% NaCl, 14.3% MgSO4) Discretionary use at approx. 6.5 g per person day 1 kg provided at study entry and 3 months, for household use Co‐interventions NR	Regular salt Discretionary use at approx. 6.5 g per person per day 1 kg provided at study entry and 3 months, for household use Co‐interventions NR	97/95/99 64.4 (9.8)/64.7 (9.9)/ 64.8 (10.3) years Female, %: 42.3/34.7/32.3 Hypertensive, %: 56.7/68.4/50.5 NR NR	Cardiovascular events Mortality Adverse events	1. Cardiovascular events: non‐fatal stroke 2. All‐cause mortality 3. Various other adverse events	1. > 3 to 12 months 2. > 3 to 12 months 3. > 3 to 12 months
Yu 2021	Individually randomised	India	Low	30% KCl (with 70% NaCl) Discretionary use of 20 g per person per day. Provided up to 5 kg every 3 months for household use Co‐interventions NR	Regular salt Discretionary use of 20 g per person per day Provided up to 5 kg every 3 months for household use Co‐interventions NR	252/252 61.5 (11.1)/ 61.7 (12.9) years Female, %: 58.3/59.2 Hypertensive Any hypertensive medication use, %: 97.2/94.4 23.1 (4.7)/23.6 (4.2) kg/m^2^	Change in blood pressure Hyperkalaemia Adverse events 24‐h urinary excretion	1. Change in DBP 2. Change in SBP 3. Hyperkalaemia 4. Various other adverse events 5. 24‐h urinary sodium excretion 6. 24‐h urinary potassium excretion	1. 3 months 2. 3 months 3. 3 months 4. ≤ 3 months 5. 3 months 6. 3 months
Zhao 2014	Individually randomised	Tibet	Low	25% KCl LSSS (with 65% NaCl and 10% MgSO4) Discretionary use Provided in sufficient amounts for household use Patients with pre‐existing antihypertensive medications not alter their prior regimen	Regular salt Discretionary use Provided in sufficient amounts for household use Patients with pre‐existing antihypertensive medications not alter their prior regimen	141/141 62.8 (11.1)/ 63.5 (11.3) years Female, %: 60.3/57.4 Hypertensive Antihypertensive use in the past month, %: 47.0/50.7 23.7 (3.1)/23.6 (3.4) kg/m^2^	Change in blood pressure Blood pressure control Adverse events	1. Change in DBP 2. Change in SBP 3. Blood pressure control 4. Antihypertensive medication use 5. Various other adverse events	1. 3 months 2. 3 months 3. 3 months 4. 3 months 5. ≤ 3 months
Zhou 2009	Individually randomised	China	Low	30% KCl LSSS (with 65% NaCl, calcium, folic acid) Discretionary use 3 kg per month provided for household use Co‐interventions NR	Regular salt Discretionary use 3 kg per month provided for household use Co‐interventions NR	62/64 67.5 (5.2)/ 65.7 (6.3) years Female, %: 56.5/57.8 Hypertensive Any antihypertensive medication use, %: 53.2/54.7 25.2 (3.5)/24.9 (3.7) kg/m^2^ 57/65 68.1 (8.3)/65.4 (4.5) years Female, %: 50.9/55.4 Normotensive N/A 23.9 (3.2)/23.7 (3.3) kg/m^2^	Change in blood pressure Cardiovascular events Change in blood glucose Change in blood lipids 24‐h urinary excretion	1. Change in DBP 2. Change in SBP 3. Various other cardiovascular events 4. Change in fasting blood glucose 5. Change in blood triglycerides 6. Change in total blood cholesterol 7. 24‐h urinary sodium excretion 8. 24‐h urinary potassium excretion	1. 6 months 2. 6 months 3 > 3 to 12 months 4. 6 months 5. 6 months 6. 6 months 7. 6 months 8. 6 months
Allaert 2013	Individually randomised	France	Unclear	No KCl content in LSSS (97% NaCl and 3% chitosan) Discretionary use to a maximum of 3 g per day Quantity provided NR Lifestyle advice (eating less fat and sugar, avoidance of liquorice) and physical exercise	Sea salt Discretionary use to a maximum of 3 g per day Quantity provided NR Lifestyle advice (eating less fat and sugar, avoidance of liquorice) and physical exercise	21/19 59.1 (11.6)/ 58.0 (12.7) years Female, %: 61.9/57.9 Hypertensive None 25.1 (3.8)/27.7 (5.8) kg/m^2^	Change in blood pressure Blood pressure control	1. Change in DBP 2. Change in SBP 3. Blood pressure control	1. 8 weeks 2. 8 weeks 3. 8 weeks
Arzilli 1986	Individually randomised	Italy	Unclear	Unknown KCl LSSS Discretionary use 2 g twice daily provided Hospital diet containing 20 mmol Na per day provided Co‐interventions NR	Regular salt Discretionary use 2 g twice daily provided Hospital diet containing 20 mmol Na per day provided Co‐interventions NR	10/10 28 to 53 years Female, %: 40 Hypertensive NR NR	Change in blood pressure	1. Change in DBP 2. Change in SBP	1. See Table 7 2. See Table 7
Bernabe‐Ortiz 2014	Stepped‐wedge (villages)	Peru	Unclear	25% KCl LSSS (with 75% NaCl) Discretionary use Provision of LSSS via salt supply chain in each village. Social marketing strategy promoting LSSS use	Regular salt Discretionary use Normal salt supply chain	2376 (total) 43.3 (17.2) years Female, %: 50.4 Hypertensive, %: 18.3 NR 27.2 (4.6) kg/m^2^	Change in blood pressure Blood pressure control 24‐h urinary excretion	1. Change in DBP 2. Change in SBP 3. Hypertension 4. 24‐h urinary sodium excretion 5. 24‐h urinary potassium excretion	1. 30 months 2. 30 months 3. 30 months 4. 30 months 5. 30 months
Kawasaki 1998	Individually randomised	Japan	Unclear	10.1% K LSSS (with 22.9 % Na; 1.2% Mg) Discretionary and non‐discretionary use (soy sauce, miso) Participants to refrain from dining out and were not to change their lifestyle during the study period.	Regular salt Discretionary and non‐discretionary use (regular soy sauce, miso) Participants to refrain from dining out and were not to change their lifestyle during the study period.	21/20 65.9 (7.4)/65.8 (7.6) years Female, %: 47.6/ 50 Hypertensive, %: 47.6/40.0 Antihypertensive medication use, %: 19.0/20.0 22.9 (2.7)/23.3 (2.3) kg/m^2^	Change in blood pressure Change in blood potassium Change in blood lipids 24‐h urinary excretion	1. Change in DBP 2. Change in SBP 3. Change in blood potassium 4. Change in blood triglycerides 5. Change in total blood cholesterol 6. 24‐h urinary sodium excretion 7. 24‐h urinary potassium excretion	1. See Table 7 2. See Table 7 3. 5 weeks 4. 5 weeks 5. 5 weeks 6. 5 weeks 7. 5 weeks
Li 2016	Cluster‐randomised (villages)	China	Unclear	20.0% KCl (up to 35.0%) (with 70.0% ± 10.0% NaCl, iodine) Discretionary use LSSS for purchase at village shops (subsidised LSSS or non‐subsidised LSSS) Community‐based health education programme on salt reduction 50% of villages included in a concurrent trial (primary‐care‐based high cardiovascular risk management package delivered by village doctors)	Regular salt Discretionary use Regular salt for purchase at village shops 50% of villages included in a concurrent trial (primary‐care‐based high cardiovascular risk management package delivered by village doctors)	1268/1253/1272 Age NR Gender NR Hypertensive status NR NR NR	Change in blood pressure Blood pressure control Cardiovascular events Hyperkalaemia Change in BMI Renal function 24‐h urinary excretion	1. Change in DBP 2. Change in SBP 3. Hypertension 4. Antihypertensive medication use 5. Various other cardiovascular events 6. Hyperkalaemia 7. Change in BMI 8. Microalbuminuria 9. Macroalbuminuria 10. Change in uACR 11. 24‐h urinary sodium excretion 12. 24‐h urinary potassium excretion	1. 18 months 2. 18 months 3. 18 months 4. 18 months 5. See Table 7 6. See Table 7 7. 18 months 8. 18 months 9. 18 months 10. 18 months 11. 18 months 12. 18 months
Mu 2009	Cluster‐randomised (families)	China	Unclear	Unknown KCl LSSS Discretionary use Provided for household use (quantity NR) Co‐interventions NR	Regular salt Discretionary use Provided for household use (quantity NR) Co‐interventions NR	101/114 20.3 (3.1)/21.4 (3.9) years Female, %: 55.5/47.4 Hypertensive NR 23.6 (2.0)/23.8 (2.1) kg/m^2^	Change in blood pressure Hyperkalaemia Adverse events	1. Change in DBP 2. Change in SBP 3. Hyperkalaemia 4. Various other adverse events	1. See Table 7 2. See Table 7 3. 2 years 4. > 12 months
Omvik 1995	Individually randomised	Norway	Unclear	KCl 28% LSSS (with NaCl 57%; MgSO_4_ 12%; lysine 2%). Discretionary use 500 g provided for household use (frequency NR) Participants instructed re. salt‐restricted diet	Regular salt Discretionary use 500 g provided for household use (frequency NR) Participants instructed re.salt‐restricted diet	20/20 45.9/42.7 years Female, %: 30/35 Hypertensive None NR	Change in blood pressure Cardiovascular events Change in blood potassium Renal function 24‐h urinary excretion	1. Change in DBP 2. Change in SBP 3. Various other cardiovascular events 4. Change in blood potassium 5. Change in serum creatinine 6. 24‐h urinary sodium excretion 7. 24‐h urinary potassium excretion	1. 6 months 2. 6 months 3. > 3 to 12 months 4. 6 months 5. 6 months 6. 6 months 7. 6 months
Pereira 2005	Individually randomised	Brazil	Unclear	50% KCl LSSS (with 50% NaCl) Discretionary use 1 kg provided for household use Individualised hypocaloric diet and increased physical activity	Regular salt Discretionary use 1 kg provided for household use Individualised hypocaloric diet and increased physical activity	15/13 45.4 (13.2)/ Gender NR Hypertensive All participants on thiazide diuretics 32.5 (13.2)/30.2 (2.7) kg/m^2^	Change in blood pressure Change in blood potassium Hypokalaemia Change in BMI Renal function Change in blood lipids	1. Change in DBP 2. Change in SBP 3. Change in blood potassium 4. Hypokalaemia 5. Change in BMI 6. Change in serum creatinine 7. Change in blood triglycerides 8. Change in total blood cholesterol	1. 12 weeks 2. 12 weeks 3. 12 weeks 4. 12 weeks 5. 12 weeks 6. 12 weeks 7. 12 weeks 8. 12 weeks
Sarkkinen 2011	Individually randomised	Finland	Unclear	25% KCl LSSS (with 50% NaCl; 25% Mg) Discretionary use (quantity provided NR) Non‐discretionary use (processed main dishes, bread, sausage/cold cuts and Edam cheese; to replace 60% of usual sodium intake) Participants instructed to avoid salt‐rich products, products containing bioactive peptides, licorice, ammonium chloride products and any food supplements that may affect BP	Regular salt Discretionary use Non‐discretionary use (processed main dishes, bread, sausage/cold cuts and Edam cheese with regular salt content) Participants instructed to avoid salt‐rich products, products containing bioactive peptides, licorice, ammonium chloride products and any food supplements that may affect BP	22/23 57 (12)/54 (11) years Female, %: 59/39 Hypertensive None 28 (3)/28 (3) kg/m^2^	Change in blood pressure Cardiovascular events Adverse events Change in blood potassium Change in BMI 24‐h urinary excretion	1. Change in DBP 2. Change in SBP 3. Various other cardiovascular events 4. Various other adverse events 5. Change in blood potassium 6. Change in BMI 7. 24‐h urinary sodium excretion 8. 24‐h urinary potassium excretion	1. 8 weeks 2. 8 weeks 3. ≤ 3 months 4. ≤ 3 months 5. See Table 7 6. 8 weeks 7. 8 weeks 8. 8 weeks
Suppa 1988	Individually randomised	Italy	Unclear	KCl 25% (with 50% NaCl and 15% K_3_C_6_H_5_O_7_) Discretionary use 2 g provided twice daily Co‐interventions NR	Regular salt Discretionary use 2 g provided twice daily Co‐interventions NR	163/159 47.1 (9.8)/47.8 (10.1) years Female, %: 35.6/39 Hypertensive All participants on β‐blocker monotherapy (Metoprolol) NR	Change in blood pressure Cardiovascular events Adverse events 24‐h urinary excretion	1. Change in DBP 2. Change in SBP 3. Various other cardiovascular events 4. Various other adverse events 5. 24‐h urinary sodium excretion 6. 24‐h urinary potassium excretion	1. 4 weeks 2. 4 weeks 3. See Table 7 4. See Table 7 5. 4 weeks 6. 4 weeks
Toft 2020	Cluster‐randomised (families)	Denmark	Unclear	< 30% KCl LSSS (Per 100g: approximately 8000 mg sodium; 870 mg Mg and 100 mg‐200 mg K estimated from the technical data sheet) Non‐discretionary use (LSSS bread products to replace usual consumption), provided to families twice a week Co‐interventions NR	Regular salt Non‐discretionary use (regular wholegrain bread products for usual consumption), provided to families twice a week Co‐interventions NR	81/101 41.5 (9.5)/ 40.9 (8.0) years Female, %: 47.5/53.1 Normotensive N/A 25.8 (3.8)/24.8 (4.1) kg/m^2^	Change in blood pressure Change in BMI Change in blood glucose Change in blood lipids 24‐h urinary excretion	1. Change in DBP 2. Change in SBP 3. Change in BMI 4. Change in fasting blood glucose 5. Change in blood triglycerides 6. Change in total blood cholesterol 7. 24‐h urinary sodium excretion 8. 24‐h urinary potassium excretion	1. 4 months 2. 4 months 3. 4 months 4. 4 months 5. 4 months 6. 4 months 7. 4 months 8. 4 months
Zhou 2013	Cluster‐randomised (families)	China	Unclear	25% KCl LSSS (with 65% NaCl, 10% MgSO_4_). Discretionary use Estimated amount (based upon baseline salt intake) provided every 3 months for household use Co‐interventions NR	Regular salt Discretionary use Estimated amount (based upon baseline salt intake) provided every 3 months for household use Co‐interventions NR	224/238 45.63 (13.72)/ 47.05 (13.46) years Female, %: 50.45/50.84 NR Any antihypertensive use, %: 41.07/40.0 25.94 (3.82)/26.66 (4.27) kg/m^2^	Change in blood pressure Mortality Blood pressure control	1. Change in DBP 2. Change in SBP 3. Cardiovascular mortality 4. Stroke mortality 5. All‐cause mortality 6. Antihypertensive medication use	1. 36 months 2. 36 months 3. 13 years 4. 13 years 5. 13 years 6. 36 months
Chang 2006	Cluster‐randomised (Retirement home kitchens)	Taiwan	High	49% KCl LSSS (with 49% NaCl, 2% other additives) Discretionary use LSSS gradually replaced regular salt in the kitchens within a 4‐week period. Frequency and quantity provided NR Regular condiments and spices e.g. soy sauce and MSG, not limited	Regular salt Discretionary use Frequency and quantity provided NR Regular condiments and spices e.g. soy sauce and MSG, not limited	768/1213 75.21 (7.37)/74.67 years Male Hypertensive, % (n/N): 40.2/40.4 NR 23.3 (3.5) kg/m^2^	Mortality	1. Cardiovascular mortality 2. All‐cause mortality	1. 2.6 years mean follow‐up 2. 2.6 years mean follow‐up
Geleijnse 1994	Individually randomised	Netherlands	High	41% KCl LSSS (with 41% NaCl, 17% Mg) Discretionary use Non‐discretionary use (use of LSSS in bread, cheese, luncheon meats, canned and instant soups, smoked sausage ‐ replacement of approx. 57% of usual salt intake) Quantity provided and frequency NR Participants were instructed not to change dietary and lifestyle habits.	Regular salt Discretionary use Non‐discretionary use (use of regular bread, cheese, luncheon meats, canned and instant soups, smoked sausage) Quantity provided and frequency NR Participants were instructed not to change dietary and lifestyle habits.	49/51 65.7 (4.6)/ 67.1 (4.5) years Female, %: 47/51 Hypertensive None 27.1 (3.4)/27.2 (3.2) kg/m^2^	Change in blood pressure Change in blood potassium Change in blood lipids 24‐h urinary excretion	1. Change in DBP 2. Change in SBP 3. Change in blood potassium 4. Change in total blood cholesterol 5. 24‐h urinary sodium excretion 6. 24‐h urinary potassium excretion	1. 24 weeks 2. 24 weeks 3. 24 weeks 4. 24 weeks 5. 24 weeks 6. 24 weeks
Gilleran 1996	Individually randomised	United Kingdom	High	40% KCl LSSS (with 50% NaCl and 10% MgSO_4_). Discretionary use 680 g provided monthly for household use Co‐interventions NR	Regular salt Discretionary use 680 g provided monthly for household use Co‐interventions NR	20/20 62.5 (7.8)/ 59.2 (10.8) years Female, %: 40/40 Hypertensive None 28.1 (4.6)/28.6 (3.7) kg/m^2^	Change in blood pressure Cardiovascular events Change in blood lipids 24‐h urinary excretion	1. Change in DBP 2. Change in SBP 3. Cardiovascular events: non‐fatal stroke 4. Change in blood triglycerides 5. Change in total blood cholesterol 6. 24‐h urinary sodium excretion 7. 24‐h urinary potassium excretion	1. 9 months 2. 9 months 3. ≤ 3 months 4. 9 months 5. 9 months 6. 9 months 7. 9 months
Hu 2018	Cluster‐randomised (families)	China	High	25% KCl LSSS (with 65% NaCl, 10% MgSO_4_). Discretionary use 1 kg bags provided for household use (frequency NR) Participants instructed to avoid any changes in dietary or lifestyle habits.	Regular salt Discretionary use 1 kg bags provided for household use (frequency NR) Participants instructed to avoid any changes in dietary or lifestyle habits.	110/110 57.1 (10.9)/ 57.6 (10.1) years Female, %: 33.6/60.0 Hypertensive Antihypertensive medication use, %: 71.8/77.3 27.6 (3.3)/28.3 (3.5) kg/m^2^ 187/186 family members 45.5 (17.5)/45.7 (17.4) years Female, %: 45.5/47.4 Hypertensive, % (n/N): 31.6/24.2 None 24.9 (3.8)/25.2 (4.3) kg/m^2^	Change in blood pressure Adverse events Blood pressure control	1. Change in DBP 2. Change in SBP 3. Various other adverse events 4. Antihypertensive medication use	1. 12 months 2. 12 months 3. > 3 to 12 months 4. 12 months
Mu 2003	Individually randomised	China	High	Unknown KCl LSSS Discretionary use Provided for household use monthly (quantity NR) Co‐interventions: None	Regular salt Discretionary use Provided for household use monthly (quantity NR) Co‐interventions: None	110/110 20.7(2.0)/20.4 (2.2) years Female; %: 47.3/49 Hypertensive NR 21.9 (2.9)/22.2 (3.1) kg/m^2^	Change in blood pressure	1. Change in DBP 2. Change in SBP	1. 2 years 2. 2 years
Zhang 2015	Cluster‐randomised (nursing homes)	China	High	50% KCl (with 50% NaCl) Discretionary use of up to 10 g per person per day LSSS provided every 3 months Co‐interventions NR	Regular salt Discretionary use of up to 10 g per person per day Regular salt provided every 3 months Co‐interventions NR	NR 65 years Gender NR NR NR NR	Change in blood pressure Change in blood potassium Hyperkalaemia Renal function	1. Change in DBP 2. Change in SBP 3. Change in blood potassium 4. Hyperkalaemia 5. Change in serum creatinine 6. Microalbuminuria	1. 3 years 2. 3 years 3. 1 to 1.5 years 4. 1 to 1.5 years 5. 1 to 1.5 years 6. 3 years

Abbreviations:  24‐h: 24‐hour BMI: body mass index BP: blood pressure DBP: diastolic blood pressure K_3_C_6_H_5_0_7_: potassium citrate K(Cl): potassium (chloride) LSSS: low‐sodium salt substitutes Mg(SO_4_): magnesium (sulphate) MSG: monosodium glutamate N/A: not applicable Na(Cl): sodium (chloride) NR: not reported SBP: systolic blood pressure uACR: urinary albumin‐to‐creatinine ratio

##### Primary outcomes

###### Change in diastolic blood pressure (DBP, mmHg)

GRADE assessment suggests that LSSS probably reduce DBP slightly, on average, compared to regular salt in adults (moderate‐certainty evidence, downgraded once for inconsistency). 

Average reductions in DBP ranged from 0.6 mmHg to 11.33 mmHg with LSSS and from a reduction of 7 mmHg to an increase of 2.6 mmHg with regular salt in the 19 trials that reported this outcome. Two trials did not have average changes per group available: Li 2016 reported end values and no baseline measures; Neal 2021 reported only mean differences between groups. The meta‐analysis showed small, important effects on DBP on average between LSSS and regular salt groups (MD ‐2.43 mmHg, 95% confidence interval (CI) ‐3.50 to ‐1.36, I^2^ = 88%, 20,830 participants, 19 RCTs, moderate‐certainty evidence, Analysis 1.1). Follow‐up ranged from four weeks to 60 months. 

This small yet important effect was confirmed by sensitivity analyses, including only trials with 'low' or 'unclear' overall risk of bias (Analysis 1.12) and including only trials randomising participants at the individual level, i.e. excluding cluster‐RCTs (Analysis 1.13). This direction of effect was also seen in a large stepped‐wedge cluster trial following 2376 participants over 30 months (unclear overall risk of bias), though the magnitude of the effect was considerably diminished (Analysis 1.14).

The estimated population impact, as described in Appendix 2, indicated that the effect of the primary meta‐analysis (Analysis 1.1) corresponded to an estimated 60 (ranging from 35 to 83) stroke deaths prevented per 100,000 persons, aged 50 years and older, per year.

Subgroup analyses were undertaken for this outcome due to the presence of substantial heterogeneity. In line with Cochrane guidance (Deeks 2020) detailing the limitations of subgroup analyses, caution was taken in the interpretation of findings from these subgroup analyses. Subgrouping by study duration (Analysis 1.2) suggests there may be no important differences in average effects between subgroups. Subgrouping participants by age (Analysis 1.3), gender (Analysis 1.4), ethnicity (Analysis 1.5), BMI (Analysis 1.6), blood pressure status (Analysis 1.7) or baseline 24‐h urinary sodium (Analysis 1.10) or potassium (Analysis 1.11) excretion further suggested there may be no important clinical differences in average effects between subgroups. Subgrouping by intervention characteristics also suggests there may be no important differences in average effects between the manner of LSSS implementation (Analysis 1.8) or the type of LSSS (Analysis 1.9).

Five trials reported this outcome in an unusable format (e.g. reported only in a figure from which we could not extract exact values, or did not report standard deviations and participant numbers along with mean change), and usable data were not provided when requested from authors (Table 7). The funnel plot (Figure 4) shows that most trials had similar effect sizes despite varying inter‐trial standard errors, thereby limiting the ability to assess asymmetry. The mean difference in the fixed‐effect model, an analytical approach that gives less weight to small studies, for the primary analysis (‐2.27 mmHg, 95% CI ‐2.56 to ‐1.98) was similar to the mean difference when using the random‐effects model, which gives more weight to smaller studies, for the primary analysis (‐2.43 mmHg, 95% CI ‐3.50 to ‐1.36, Analysis 1.1). This suggests that any small‐study effects have little impact on the intervention effect estimate.

**4:**
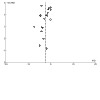
**Funnel plot for change in DBP (Analysis 1.1) in comparison 1**

###### Change in systolic blood pressure (SBP, mmHg)

GRADE assessment suggests that LSSS probably reduce SBP slightly, on average, compared to regular salt in adults (moderate‐certainty evidence, downgraded once for inconsistency).

Average reductions in SBP ranged from 1.5 mmHg to 15.25 mmHg with LSSS and from a reduction of 6.8 mmHg to an increase of 4 mmHg with regular salt in the 20 trials that reported this outcome. Three trials did not have average changes per group available: Li 2016 reported end values and no baseline measures; CSSS Collaborative Group 2007 and Neal 2021 reported only mean differences between groups. The meta‐analysis showed small, important effects on SBP on average between LSSS and regular salt groups (MD ‐4.76 mmHg, 95% CI ‐6.01 to ‐3.50, I^2^ = 78%, 21,414 participants, 20 RCTs, moderate‐certainty evidence, Analysis 1.15). Follow‐up ranged from four weeks to 60 months. 

This small yet important effect was confirmed by sensitivity analyses, including only trials with 'low' or 'unclear' overall risk of bias (Analysis 1.26) and including only trials randomising participants at the individual level, i.e. excluding cluster‐RCTs (Analysis 1.27). This direction of effect was also seen in the stepped‐wedge cluster trial that followed 2376 participants over 30 months (unclear overall risk of bias), though the magnitude of the effect was considerably diminished (Analysis 1.28).

The estimated population impact, as described in Appendix 2, indicated that the effect of the primary meta‐analysis (Analysis 1.15) corresponded to an estimated 53 (ranging from 40 to 65) stroke deaths prevented per 100,000 persons, aged 50 years and older, per year.

Subgroup analyses were undertaken for this outcome due to the presence of substantial heterogeneity. In line with Cochrane guidance (Deeks 2020) detailing the limitations of subgroup analyses, caution was taken in the interpretation of findings from these subgroup analyses. Subgrouping by study duration (Analysis 1.16), and subgrouping participants by age (Analysis 1.17), gender (Analysis 1.18), ethnicity (Analysis 1.19), BMI (Analysis 1.20), blood pressure status (Analysis 1.21) or baseline 24‐h urinary sodium (Analysis 1.24) or potassium (Analysis 1.25) excretion suggests there may be no important clinical differences in average effects between subgroups. Subgrouping by intervention characteristics also suggested there may be no important differences in average effects between the manner of LSSS implementation (Analysis 1.22) or the type of LSSS (Analysis 1.23).

Four trials reported this outcome in an unusable format (e.g. reported only between‐group P values or did not report standard deviations and participant numbers along with mean change), and usable data were not provided when requested from authors (Table 7). The funnel plot (Figure 5) shows that most trials had similar effect sizes and standard errors, with some outliers, thereby limiting the ability to assess asymmetry. The mean difference in the fixed‐effect model for the primary analysis (‐3.92 mmHg, 95% CI ‐4.37 to ‐3.47) was similar to the mean difference when using the random‐effects model for the primary analysis (‐4.76 mmHg, 95% CI ‐6.01 to ‐3.50, Analysis 1.15). This suggests that any small‐study effects have little impact on the intervention effect estimate.

**5:**
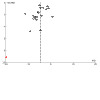
**Funnel plot for change in SBP (Analysis 1.14) in comparison 1**

###### Hypertension

GRADE assessment suggests that, on average, LSSS may result in little to no difference in hypertension in the adult population, when compared to regular salt (low‐certainty evidence, downgraded once for risk of bias and once for imprecision).

One study following participants for 18 months reported on this outcome (risk ratio (RR) 0.97, 95% CI 0.90 to 1.03, 2566 participants, 1 RCT, low‐certainty evidence, Analysis 1.29), with 725 participants in the LSSS group and 738 participants in the regular salt group having prevalent hypertension at the end of the study. The absolute effect for hypertension was 17 fewer per 1000 (95% CI 58 fewer to 17 more). The stepped‐wedge cluster trial (unclear overall risk of bias) reported on incident hypertension in 1914 participants represented by 2712.3 person‐years at risk in the LSSS group and 1961.1 person‐years at risk in the regular salt group, and found a reduction in hypertension with LSSS compared to regular salt (Analysis 1.30).

###### Blood pressure control

GRADE assessment suggests that the evidence is very uncertain about the effect of LSSS on blood pressure control in adults, when compared to regular salt (very low‐certainty evidence, downgraded once for risk of bias, once for indirectness and once for imprecision).

Two small studies reported on this outcome (RR 2.12, 95% CI 1.32 to 3.41, I^2^ = 0%, 253 participants, 2 RCTs, very low‐certainty evidence, Analysis 1.31), with the number of participants in the LSSS group achieving blood pressure control ranging from 16 to 19 and participants in the regular salt group achieving blood pressure control ranging from seven to 10. The absolute effect for blood pressure control was 143 more per 1000 (95% CI 41 more to 308 more). The two trials reporting this outcome had follow‐up at eight weeks and three months.

###### Cardiovascular events: various

GRADE assessment suggests that the evidence is very uncertain about the effect of LSSS interventions on various other cardiovascular events when compared to regular salt in the adult population (very low‐certainty evidence, downgraded once for indirectness and twice for imprecision).

It should be noted that a very small number of participants presented with various other cardiovascular events in both groups for the five trials reporting this outcome. Event numbers ranged from zero to eight with LSSS and from zero to five with regular salt. The meta‐analysis of the RR was 1.22 (95% CI 0.49 to 3.04, I^2^ = 0%, 982 participants, 5 RCTs, very low‐certainty evidence, Analysis 1.32) when comparing LSSS and regular salt. The absolute effect for various other cardiovascular events was 357 more per 100,000 (95% CI 828 fewer to 3310 more). Two trials reporting this outcome had follow‐up at ≤ 3 months, while three followed participants for > 3 to 12 months.

Two trials reported this outcome in an unusable format (i.e. numbers of events per group and numbers of participants per group not reported), and usable data were not provided when requested from authors (Table 7).

###### Cardiovascular events: non‐fatal stroke

GRADE assessment for this outcome suggests that, on average, LSSS probably reduce non‐fatal stroke events slightly in adults, when compared to regular salt (moderate‐certainty evidence, downgraded once for indirectness).

The number of participants with non‐fatal stroke in two small trials (Gilleran 1996; Pan 2017) reporting on this outcome at ≤ 3 months and > 3 to 12 months, respectively, ranged from zero to four with LSSS and one participant each with regular salt. The third, a large cluster‐RCT (Neal 2021) that followed participants for a mean of 4.75 years, reported rates of events: 22.36 events per 1000 person‐years in the LSSS group and 24.86 events per 1000 person‐years in the regular salt group (rate ratio 0.90, 95% CI 0.80 to 1.01). The meta‐analysis combining these data as risk ratios resulted in an RR of 0.90 (95% CI 0.80 to 1.01, I^2^ = 0%, 21,250 participants, 3 RCTs, moderate‐certainty evidence, Analysis 1.33) when comparing LSSS with regular salt. This result translates to an absolute effect for non‐fatal stroke of 20 fewer per 100,000 (95% CI 40 fewer to 2 more). Since this pooled effect was driven by a large secondary prevention trial with a preponderance of participants with previous stroke, we downgraded once for indirectness.

The estimated population impact, as described in Appendix 2, indicated that the effect of the primary meta‐analysis (Analysis 1.33) corresponded to an estimated 10 non‐fatal strokes prevented (ranging from 21 prevented to 1 caused) per 100,000 persons per year.

Sensitivity analyses, including only trials with 'low' or 'unclear' overall risk of bias (Analysis 1.34) and including only trials randomising participants at the individual level, i.e. excluding cluster‐RCTs (Analysis 1.35), did not reflect this benefit with LSSS; instead showing highly imprecise effects of little to no effect, or harm.

One trial reported this outcome in an unusable format (i.e. numbers of participants per group not reported), and usable data were not provided when requested from authors (Table 7).

###### Cardiovascular events: non‐fatal acute coronary syndrome

GRADE assessment suggests that LSSS probably reduce non‐fatal ACS events slightly, on average, when compared to regular salt in adults (moderate‐certainty evidence, downgraded once for indirectness).

A single large cluster‐RCT contributed data to this outcome at a mean follow‐up of 4.75 years, reporting rates of 3.79 events per 1000 person‐years in the LSSS group and 5.12 events per 1000 person‐years in the regular salt group. The rate ratio was 0.70 (95% CI 0.52 to 0.94, 20,995 participants, 1 RCT, moderate‐certainty evidence, Analysis 1.36) and the absolute effect for non‐fatal acute coronary syndrome was 150 fewer per 100,000 person‐years (95% CI 250 fewer to 30 fewer), when comparing LSSS with regular salt in this large secondary prevention trial in which most of the participants had a history of previous stroke. This setting limited generalisability to the general adult population, and the evidence was consequently downgraded once for indirectness.

The estimated population impact, as described in Appendix 2, indicated that the effect of the primary analysis (Analysis 1.36) corresponded to an estimated 50 (ranging from 10 to 80) non‐fatal ACS events prevented per 100,000 persons per year.

###### Cardiovascular mortality

GRADE assessment of this outcome suggests that LSSS probably reduce cardiovascular mortality slightly, on average, in adults when compared to regular salt (moderate‐certainty evidence, downgraded once for indirectness).

The number of cardiovascular mortality events per 1000 person‐years in the three trials reporting on this outcome ranged from 4.53 to 22.94 in the LSSS groups and 7.81 to 26.30 in the regular salt groups. The meta‐analysis comparing LSSS with regular salt resulted in a rate ratio of 0.77 (95% CI 0.60 to 1.00, I^2^ = 35%, 23,200 participants, 3 RCTs, moderate‐certainty evidence, Analysis 1.37). The absolute effect for cardiovascular mortality was 180 fewer per 100,000 person‐years (95% CI 310 fewer to 0 fewer). We downgraded this finding once for indirectness since the pooled effect was driven by the secondary prevention trial including a large proportion of participants with previous stroke (Neal 2021). Two trials reporting on this outcome had a mean follow‐up of between 2.6 and 4.75 years (Chang 2006; Neal 2021) while a third (Zhou 2013) reported on this outcome following three years of active intervention and ten years follow‐up.

The estimated population impact, as described in Appendix 2, indicated that the effect of the primary meta‐analysis (Analysis 1.37) corresponded to an estimated 53 cardiovascular deaths prevented (ranging from 92 prevented to none prevented or caused) per 100,000 persons per year.

A sensitivity analysis including only trials with 'low' or 'unclear' overall risk of bias (Analysis 1.38) confirmed this effect.

###### Stroke mortality

GRADE assessment suggests that the evidence is very uncertain about the effect of LSSS on stroke mortality in adults, when compared to regular salt (very low‐certainty evidence, downgraded once for indirectness and twice for imprecision).

The number of stroke mortality events per 1000 person‐years in the two trials reporting on this outcome ranged from 2.01 to 6.78 in the LSSS groups and 5.85 to 8.79 in the regular salt groups. The meta‐analysis comparing LSSS with regular salt resulted in a rate ratio 0.64 (95% CI 0.33 to 1.25, I^2^ = 45%, 21,423 participants, 2 RCTs, very low‐certainty evidence, Analysis 1.39). The absolute effect for stroke mortality was 145 fewer per 100,000 person‐years (95% CI 270 fewer to 100 more). The pooled effect was driven to a considerable extent by a large secondary prevention trial including a large proportion of participants with previous stroke, resulting in limited generalisability to the general adult population. This trial (Neal 2021) had a mean follow‐up of 4.75 years, while the second trial reporting on this outcome (Zhou 2013) had a follow‐up of three years of active intervention and ten years thereafter.

The estimated population impact, as described in Appendix 2, indicated that the effect of the primary meta‐analysis (Analysis 1.39) corresponded to an estimated 28 stroke deaths prevented (ranging from 53 prevented to 20 caused) per 100,000 persons per year.

###### Change in blood potassium (mmol/L)

GRADE assessment suggests that, on average, LSSS probably increase blood potassium slightly compared to regular salt in the adult population (moderate‐certainty evidence, downgraded once for risk of bias).

Average changes in blood potassium ranged from a reduction of 0.2 mmol/L to an increase of 0.38 mmol/L with LSSS and from a reduction of 0.2 mmol/L to an increase of 0.3 mmol/L with regular salt in the six trials that reported this outcome. The meta‐analysis showed small, important effects on blood potassium on average between LSSS and regular salt groups (MD 0.12, 95% CI 0.07 to 0.18, I^2^ = 0%, 784 participants, 6 RCTs, moderate‐certainty evidence, Analysis 1.40). 

This small yet important effect was confirmed by sensitivity analyses, including only trials with 'low' or 'unclear' overall risk of bias (Analysis 1.42) and including only trials randomising participants at the individual level, i.e. excluding cluster‐RCTs (Analysis 1.43). The trials reporting on this outcome reported results at 56 days, five weeks, 12 weeks and between one and 1.5 years each, while two trials reported results at approximately six months.

Subgroup analyses were undertaken for this outcome to explore whether there were differences in effects in subgroups based on hyperkalaemia risk. In line with Cochrane guidance (Deeks 2020) detailing the limitations of subgroup analyses, caution was taken in the interpretation of findings from these subgroup analyses. Subgrouping participants by risk of hyperkalaemia as per Table 6 suggests there may be no important clinical differences in average effects between participants not at risk, at unclear risk and those at possible risk of hyperkalaemia (Analysis 1.41).

One trial reported this outcome in an unusable format (i.e. reported change and significance of change in the control group only), and usable data were not provided when requested from authors (Table 7).

###### Hyperkalaemia 

GRADE assessment suggests that, on average, LSSS likely result in little to no difference in hyperkalaemia in the adult population when compared to regular salt (moderate‐certainty evidence, downgraded once for risk of bias).

It should be noted, however, that a very small number of participants presented with hyperkalaemia in both groups across the trials that reported this outcome. The number of participants with hyperkalaemia in the five trials reporting this outcome ranged from zero to 11 with LSSS and from zero to nine with regular salt. The meta‐analysis of the RR was 1.04 (95% CI 0.46 to 2.38, I^2^ = 0%, 22,849 participants, 5 RCTs, moderate‐certainty evidence, Analysis 1.44) when comparing LSSS to regular salt. The absolute effect for hyperkalaemia was 4 more per 100,000 (95% CI 47 fewer to 121 more).  

A sensitivity analysis including only trials with 'low' or 'unclear' overall risk of bias (Analysis 1.46) confirmed this effect, though this result was highly imprecise. A sensitivity analysis including only trials randomising participants at the individual level, i.e. excluding cluster‐RCTs (Analysis 1.47) was not informative due to zero events in both trial arms. These five trials reported results after three months, 12 months, one to 1.5 years, 2 years and a mean of 4.75 years follow‐up.

Subgroup analyses were undertaken for this outcome to explore whether there were differences in effects in subgroups based on hyperkalaemia risk. In line with Cochrane guidance (Deeks 2020) detailing the limitations of subgroup analyses, caution was taken in the interpretation of findings from these subgroup analyses. Subgrouping participants by risk of hyperkalaemia as per Table 6 suggests there may be no important clinical differences in average effects between participants not at risk, at unclear risk and those at possible risk, of hyperkalaemia (Analysis 1.45).

###### Hypokalaemia

GRADE assessment of this outcome suggests that the evidence is very uncertain about the effects of LSSS on hypokalaemia when compared to regular salt in the adult population (very low‐certainty evidence, downgraded once for risk of bias and twice for indirectness).

A single, small trial (Pereira 2005) reported no hypokalaemia events in either trial arm comparing LSSS and regular salt in young participants with hypertension requiring potassium supplementation due to the use of potassium‐depleting diuretics (RR and 95% CI not estimable, 22 participants, 1 RCT, very low‐certainty evidence, Analysis 1.48). This study reported outcomes at 12 weeks.

##### Secondary outcomes

For comparison 1, no studies reported on diabetes mellitus diagnosis or hyponatraemia.

###### All‐cause mortality

The number of all‐cause mortality events in two trials (CSSS Collaborative Group 2007; Pan 2017) reporting on this outcome at > 3 to 12 months ranged from three to four with LSSS and one to four with regular salt. Three additional trials (Chang 2006; Neal 2021; Zhou 2013) reported rates of events and rate ratios; these ranged from 11.08 to 93.45 events per 1000 person‐years in the LSSS groups and 13.66 to 101.29 events per 1000 person‐years in the regular salt groups and corresponded to rate ratios (95% CIs) of 0.92 (0.77 to 1.10), 0.88 (0.82 to 0.95) and 0.81 (0.46 to 1.42), respectively. The meta‐analysis combining these data as risk ratios resulted in an RR of 0.89 (95% CI 0.83 to 0.95, I^2^ = 0%, 24,005 participants, 5 RCTs, Analysis 1.49) when comparing LSSS with regular salt.

###### Adverse events (other)

GRADE assessment suggests that the evidence is very uncertain about the effect of LSSS on other adverse events when compared to regular salt in adults (very low‐certainty evidence, downgraded once for risk of bias, once for inconsistency and once for imprecision).

The number of participants with other adverse events in the eight trials reporting this outcome ranged from zero to 17 with LSSS and from zero to seven with regular salt. The events reported were highly diverse and not suitable for pooling in a meta‐analysis (2109 participants, 8 RCTs, very low‐certainty evidence, Analysis 1.50). Four trials reporting on other adverse events reported these at ≤ 3 months, three reported on this outcome at > 3 to 12 months and one trial reported other adverse events at > 12 months.

Subgroup analyses were undertaken for this outcome to explore whether there were differences in effects in subgroups based on hyperkalaemia risk. In line with Cochrane guidance (Deeks 2020) detailing the limitations of subgroup analyses, caution was taken in the interpretation of findings from these subgroup analyses. Subgrouping participants by risk of hyperkalaemia as per Table 6 suggests there may be no important clinical differences in average effects between participants not at risk, and those at possible risk, of hyperkalaemia (Analysis 1.51).

One trial reported this outcome in an unusable format (i.e. numbers of events per group not reported), and usable data were not provided when requested from authors (Table 7).

###### Antihypertensive medication use

The number of participants using antihypertensive medication across the four trials reporting this outcome ranged from 34 to 246 with LSSS and from 52 to 267 with regular salt. The meta‐analysis of the RR of the number of participants using antihypertensive medication was 0.80 (95% CI 0.67 to 0.95, I^2^ = 53%, 3301 participants, 4 RCTs, Analysis 1.52) when comparing LSSS and regular salt groups. The one individually randomised trial that reported this outcome had follow‐up at three months; the three cluster trials reporting this outcome had follow‐up at 12, 18 and 36 months. The stepped‐wedge cluster trial (unclear overall risk of bias) reported no changes in medication use, with 10.5% at baseline and 10.1% (P = 0.73) at the end of the study three years later; this was for hypertension and type 2 diabetes mellitus medication use combined.

Subgroup analyses were undertaken for this outcome due to the presence of substantial heterogeneity. In line with Cochrane guidance (Deeks 2020) detailing the limitations of subgroup analyses, caution was taken in the interpretation of findings from these subgroup analyses. Subgrouping by duration of study (Analysis 1.53) as well as age (Analysis 1.54), gender (Analysis 1.55), BMI (Analysis 1.56) or hypertensive status (Analysis 1.57) of participants at baseline suggests there may be no important clinical differences in average effects between subgroups. Subgrouping by ethnicity, type and implementation of LSSS, 24‐h sodium excretion at baseline and 24‐h potassium excretion at baseline could not be used to explore clinical differences, as all studies were categorised into the same subgroup.

###### Change in BMI (kg/m^2^)

Average change in BMI ranged from a reduction of 1.6 kg/m^2^ to no change with LSSS and from a reduction of 1.0 kg/m^2^ to no change with regular salt in the four trials that reported this outcome. We did not pool these results into an overall effect estimate due to considerable heterogeneity (I² = 96%, Tau² = 0.80, Chi² = 70.15, df = 3 (P < 0.00001)), but rather presented the individual effect sizes between LSSS and regular salt per study (2060 participants, 4 RCTs, Analysis 1.58). Trials reporting on this outcome followed participants for eight weeks, 12 weeks, four months and 18 months.

###### Change in serum creatinine (µmol/L)

Average change in serum creatinine ranged from a reduction of 0.8 µmol/L to an increase of 2 µmol/L with LSSS and from a reduction of 1.0 µmol/L to an increase of 3.54 µmol/L with regular salt in the three trials that reported this outcome. The mean difference in serum creatinine, on average, between LSSS and regular salt groups was 2.56 µmol/L (95% CI ‐0.59 to 5.71, I^2^ = 0%, 616 participants, 3 RCTs, Analysis 1.59). Trials reporting on this outcome followed participants for 12 weeks, six months and between one and 1.5 years.

Subgroup analyses were undertaken for this outcome to explore whether there are differences in effects in subgroups based on hyperkalaemia risk. In line with Cochrane guidance (Deeks 2020) detailing the limitations of subgroup analyses, caution was taken in the interpretation of findings from these subgroup analyses. Subgrouping participants by risk of hyperkalaemia as per Table 6 suggests there may be no important clinical differences in average effects between participants not at risk, and those at possible risk, of hyperkalaemia (Analysis 1.60).

###### Microalbuminuria

Two studies reported on this outcome (RR 0.67, 95% CI 0.53 to 0.84, I^2^ = 0%, 2382 participants, 2 RCTs, Analysis 1.61), with the number of participants in the LSSS group with microalbuminuria ranging from 45 to 64, and participants in the regular salt group with microalbuminuria ranging from 58 to 84 across trials. The two trials reporting this outcome had follow‐up at 18 months and three years.

Subgrouping participants by risk of hyperkalaemia could not be conducted as participants in both trials were at unclear risk of hyperkalaemia as per Table 6.

###### Macroalbuminuria

One trial reported on this outcome at 18 months (RR 0.48, 95% CI 0.16 to 1.39, 1903 participants, 1 RCT, Analysis 1.62), with five participants in the LSSS group experiencing macroalbuminuria events and 10 participants in the regular salt group experiencing macroalbuminuria events.

###### Change in urinary albumin‐to‐creatinine ratio (uACR)

One trial reported end values for this outcome at 18 months (MD ‐1.68, 95% CI ‐2.87 to ‐0.49, 1903 participants, 1 RCT, Analysis 1.63). 

###### Change in fasting blood glucose (mmol/L)

Two trials reported on this outcome for this comparison. Average increases in fasting blood glucose ranged from 0.1 mmol/L to 0.22 mmol/L with LSSS, while average changes ranged from no change to an increase of 0.10 mmol/L in fasting blood glucose with regular salt, across the two trials. We did not pool these results into an overall effect estimate due to considerable heterogeneity (I² = 94%, Tau² = 0.56, Chi² = 17.06, df = 1 (P < 0.0001)), but rather presented the individual effect sizes between LSSS and regular salt per study (338 participants, 2 RCTs, Analysis 1.64). The two trials reporting on the outcome followed up participants for four and six months each.

###### Change in blood triglycerides (mmol/L)

Five trials reported on change in blood triglycerides for this comparison. Average changes in blood triglycerides ranged from a reduction of 0.7 mmol/L to an increase of 0.15 mmol/L with LSSS and from a reduction of 0.01 mmol/L to an increase of 0.9 mmol/L in blood triglycerides with regular salt across the five trials. The change in blood triglycerides, on average, between LSSS and regular salt groups was ‐0.11 mmol/L (95% CI ‐0.91 to 0.69, I^2^ = 81%, 420 participants, 5 RCTs, Analysis 1.65). Trials reporting on the outcome followed up participants for five weeks, 12 weeks, four months, six months and nine months each.

Subgroup analyses were undertaken for this outcome due to the presence of substantial heterogeneity. In line with Cochrane guidance (Deeks 2020) detailing the limitations of subgroup analyses, caution was taken in the interpretation of findings from these subgroup analyses.  Subgrouping by study duration (Analysis 1.66), age (Analysis 1.67), ethnicity (Analysis 1.68), BMI (Analysis 1.69), blood pressure status (Analysis 1.70), type of LSSS (Analysis 1.72), baseline sodium excretion (Analysis 1.73) and baseline potassium excretion (Analysis 1.74) suggests there may be no important differences in average effects between these subgroups. Subgrouping by the method of implementation of LSSS yielded a few studies in each subgroup, resulting in an analysis that was not powered to detect any differences in effect (Analysis 1.71). Subgrouping by gender could not be used to explore clinical differences as all studies were categorised into the same subgroup.

###### Change in total blood cholesterol (mmol/L)

Six trials reported on change in total blood cholesterol for this comparison. One trial each followed up participants for five weeks, 12 weeks and four months; two trials reporting on this outcome had follow‐up of approximately six months and one trial followed up participants for nine months. Across the trials, average changes in total blood cholesterol ranged from a reduction of 0.7 mmol/L to an increase of 0.16 mmol/L with LSSS and from a reduction of 0.63 mmol/L to an increase of 0.24 mmol/L with regular salt. When LSSS were compared to regular salt, the mean difference in total cholesterol change on average was ‐0.31 mmol/L (95% CI ‐0.74 to 0.12, I^2^ = 85%, 509 participants, 6 RCTs, Analysis 1.75).

Subgroup analyses were undertaken for this outcome due to the presence of substantial heterogeneity. In line with Cochrane guidance (Deeks 2020) detailing the limitations of subgroup analyses, caution was taken in the interpretation of findings from these subgroup analyses. Subgrouping by study duration (Analysis 1.76), age (Analysis 1.77), ethnicity (Analysis 1.78), BMI (Analysis 1.79), blood pressure status (Analysis 1.80), implementation of LSSS (Analysis 1.81), type of LSSS (Analysis 1.82), baseline sodium excretion (Analysis 1.83) and baseline potassium excretion (Analysis 1.84) suggests there may be no important differences in average effects between these subgroups. Subgrouping by gender could not be used to explore clinical differences as all studies were categorised into the same subgroup.

###### Change in 24‐h urinary sodium excretion (mmol/24‐h)

Eleven trials reported on change in 24‐h urinary sodium excretion for this comparison. Average changes in this outcome ranged from a reduction of 75.5 mmol (1730 mg) sodium/24‐h to an increase of 20.2 mmol (460 mg) sodium/24‐h with LSSS and from a reduction of 31 mmol (710 mg) sodium/24‐h to an increase of 11 mmol (250 mg) sodium/24‐h with regular salt across the trials.  We did not pool these results into an overall effect estimate due to considerable heterogeneity (I² = 91%, Tau² = 595.13, Chi² = 107.72, df = 10 (P < 0.00001)), but rather presented the individual effect sizes between LSSS and regular salt per study (3885 participants, 11 RCTs, Analysis 1.85). Three trials reporting on this outcome followed up participants for four, five, and eight weeks; five trials followed up participants for three, four, nine, 18 and 60 months each; three trials reported on the outcome at approximately six months.

The stepped‐wedge cluster trial (unclear overall risk of bias) reporting on 605 participants reported little to no difference between a LSSS and regular salt for this outcome (Analysis 1.86).

###### Change in 24‐h urinary potassium excretion (mmol/24‐h)

Eleven trials reported on change in 24‐h urinary potassium excretion for this comparison. Average changes in this outcome ranged from a reduction of 4.4 mmol (170 mg) potassium/24‐h to an increase of 18.5 mmol (720 mg) potassium/24‐h with LSSS and from a reduction of 16 mmol (630 mg) potassium/24‐h to an increase of 4.6 mmol (180 mg) potassium/24‐h with regular salt across the trials. The meta‐analysis showed a difference, on average, favouring LSSS when compared to regular salt in the effect on 24‐h urinary potassium excretion between LSSS and regular salt groups (MD 11.44 mmol (450 mg) potassium/24‐h, 95% CI 7.62 to 15.26 mmol/24‐h [298 to 597 mg/24‐h], I^2^ = 82%, 3885 participants, 11 RCTs, Analysis 1.87). Three trials reporting on the outcome followed up participants for four, five, and eight weeks, five trials followed up participants for three, four, nine, 18 and 60 months each; three trials reported on the outcome at approximately six months.

The stepped‐wedge cluster trial (unclear overall risk of bias) reporting on 605 participants found a similar direction of effect, though it was far smaller (Analysis 1.98).

Subgroup analyses were undertaken for this outcome due to the presence of substantial heterogeneity. In line with Cochrane guidance (Deeks 2020) detailing the limitations of subgroup analyses, caution was taken in the interpretation of findings from these subgroup analyses. Subgrouping by study duration (Analysis 1.88), age (Analysis 1.89), gender (Analysis 1.90), ethnicity (Analysis 1.91), BMI (Analysis 1.92), type of LSSS (Analysis 1.95), baseline sodium excretion (Analysis 1.96) and baseline potassium excretion (Analysis 1.97) suggests there may be no important differences in average effects between these subgroups.

Subgrouping by blood pressure status suggested some differences between subgroups (Analysis 1.93), but this was driven mainly by a large subgroup of participants of mixed hypertensive status from one study (Neal 2021). Therefore, the observed difference in effect was not considered to be attributable to hypertensive status. Subgrouping by the method of implementation of LSSS yielded a few studies in two of the three subgroups, resulting in an analysis that was not powered to detect any differences in effect (Analysis 1.94).

#### Comparison 2. Low‐sodium salt substitutes versus regular salt or no active intervention in children

Table 2 presents the effects of LSSS compared to regular salt in children on changes in DBP and SBP.

A single RCT randomising families as clusters and reporting on 92 children was included in this comparison. Key details about the study in this comparison, including study design, setting and overall risk of bias; characteristics of the intervention, comparator, population and outcomes; method of synthesis, and time points of measurement are included in the Overview of Synthesis and Included Studies (OSIS) table (Table 9).

**7:** Comparison 2: Overview of Synthesis and Included Studies (OSIS)

**Study name (year)**	**Study design** (individual vs stepped‐wedge vs cluster‐randomised [and unit of randomisation, if applicable])	**Country of conduct**	**Overall risk of bias** (arranged low to high)	**Key details of intervention** (% KCl, LSSS implementation [discretionary, non‐discretionary, both], quantity, provided or purchased, co‐interventions [education, advice])	**Key details of the comparator** (implementation [discretionary, non‐discretionary, both], quantity, provided or household supply, co‐interventions [education, advice])	**Population** **(No. of participants randomised [intervention/control], age, gender, hypertensive status, anti‐hypertensive medication use, BMI)**	**Outcome domains with available data** (synthesis method/metric)	**Specific outcomes measure**	**Time point of measurement**
Toft 2020	Cluster‐randomised (families)	Denmark	Unclear	< 30% KCl LSSS (per 100 g: approximately 8000 mg sodium; 870 mg Mg and 100 mg‐200 mg K estimated from the technical data sheet) Non‐discretionary use (LSSS bread products to replace usual consumption), provided to families twice a week Co‐interventions NR	Regular salt Non‐discretionary use (regular wholegrain bread products for usual consumption), provided to families twice a week Co‐interventions NR	40/52 9.5 (4.2)/8.4 (3.5) years Female, %: 47.5/48.1 Normotensive N/A 18.0 (2.9)/16.9 (2.8) kg/m^2^	Change in blood pressure Change in BMI 24‐h urinary excretion	1. Change in DBP 2. Change in SBP 3. Change in BMI 4. 24‐h urinary sodium excretion 5. 24‐h urinary potassium excretion	1. 4 months 2. 4 months 3. 4 months 4. 4 months 5. 4 months

Abbreviations:  24‐h: 24‐hour BMI: body mass index DBP: diastolic blood pressure K(Cl): potassium (chloride) LSSS: low‐sodium salt substitute Mg: magnesium N/A: not applicable NR: not reported

##### Primary outcomes

For comparison 2, no studies reported on hypertension, blood pressure control, change in blood potassium, hyperkalaemia or hypokalaemia.

###### Change in diastolic blood pressure (DBP, mmHg)

GRADE assessment suggests that the evidence is very uncertain about the effect of LSSS on changes in DBP, when compared to regular salt, in children (very low‐certainty evidence, downgraded once risk of bias, once for indirectness and once for imprecision).

The average change in DBP was a reduction of 2.1 mmHg in the group that ate bread containing LSSS and a reduction of 5.87 mmHg in the group that ate bread containing regular salt for the single cluster‐RCT that reported this outcome at four months follow‐up. The mean difference, when comparing these groups, was 1.28 mmHg (95% CI ‐1.56 to 4.12, 92 participants, 1 RCT, very low‐certainty evidence, Analysis 2.1). 

###### Change in systolic blood pressure (SBP, mmHg)

GRADE assessment suggests that the evidence is very uncertain about the effect of LSSS on changes in SBP, when compared to regular salt, in children (very low‐certainty evidence, downgraded once risk of bias, once for indirectness and once for imprecision).

The average change in SBP was a reduction of 5.1 mmHg in the group that ate bread containing LSSS and a reduction of 6.05 mmHg in the group that ate bread containing regular salt for the single cluster‐RCT that reported this outcome at four months follow‐up. The mean difference when comparing these groups was 0.12 mmHg (95% CI ‐4.41 to 4.64, 92 participants, 1 RCT, very low‐certainty evidence, Analysis 2.2). 

##### Secondary outcomes

For comparison 2, no studies reported on adverse events (other), cardiovascular events, antihypertensive medication use, all‐cause mortality, cardiovascular mortality, bone densitometry measures, renal function, bone health, hyponatraemia, changes in fasting blood glucose, changes in blood triglycerides or changes in total blood cholesterol.

###### Growth changes (e.g. z‐scores for height‐ or length‐for‐age (HAZ or LAZ), weight‐for‐height (WHZ), weight‐for‐age (WAZ), BMI‐for‐age)

In the trial reporting on BMI changes in children, the unadjusted average reductions in BMI were 1.62 kg/m^2^ with bread containing LSSS and 1.50 kg/m^2^ with bread containing regular salt. The mean difference in BMI, on average, was 0.94 kg/m^2^ (95% CI 0.85 to 1.03, 92 participants, 1 RCT, Analysis 2.3) when comparing these groups at four months.

###### Change in 24‐h urinary sodium excretion (mmol/24‐h) 

The average change in 24‐h urinary sodium excretion was an increase of 11.4 mmol (262 mg) sodium/24‐h in the group that ate bread containing LSSS and a reduction of 3.2 mmol (74 mg) sodium/24‐h in the group that ate bread containing regular salt for the single cluster‐RCT that reported this outcome at four months follow‐up. The mean difference when comparing these groups was 14.60 mmol (336 mg) sodium/24‐h (95% CI ‐11.22 to 40.42 mmol/24‐h [‐258 to 929 mg/24‐h], 92 participants, 1 RCT, Analysis 2.4).

###### Change in 24‐h urinary potassium excretion (mmol/24‐h) 

The average change in 24‐h urinary potassium excretion was a reduction of 1.6 mmol (64 mg) potassium/24‐h in the group that ate bread containing LSSS and a reduction of 5.7 mmol (223 mg) potassium/24‐h in the group that ate bread containing regular salt for the single cluster‐RCT that reported this outcome at four months follow‐up. The mean difference when comparing these groups was 4.10 mmol (160 mg) potassium/24‐h (95% CI ‐5.13 to 13.33 mmol/24‐h [‐201 to 521 mg/24‐h], 92 participants, 1 RCT, Analysis 2.5).

#### Comparison 3. Low‐sodium salt substitutes versus regular salt or no active intervention in pregnant women

No eligible studies in pregnant women were found.

## Discussion

### Summary of main results

This review examined the effects and safety of LSSS compared to regular salt or no active intervention on blood pressure and cardiovascular health in adults, children and pregnant women. We included 16 RCTs and ten cluster‐RCTs (n = 26) conducted in adults from a range of different settings, including nursing homes, hospitals, rural and suburban households and communities, as well as rural villages. Importantly, these 26 trials included various clinical subpopulations, with nearly two‐thirds of trials conducted in people with existing hypertension. We also included one cluster‐RCT including healthy children. The proportion of sodium chloride replacement in the LSSS interventions varied from approximately 3% to 77% with 24 trials replacing the sodium chloride with some potassium chloride (more details in the Characteristics of included studies section). We did not find any eligible studies in pregnant women. We also did not find any eligible prospective analytical cohort studies. 

#### Low‐sodium salt substitutes versus regular salt or no active intervention in adults

Adult participants in groups allocated to LSSS had lowered DBP and SBP (range of reductions: 0.6 to 11.33 mmHg and 1.5 to 15.25 mmHg, respectively) on average, while those allocated to regular salt had smaller reductions and more variable results for both DBP (range of change: 7 mmHg reduction to 2.6 mmHg increase) and SBP (range of change: 6.8 mmHg reduction to 4 mmHg increase) on average.

In adult participants, meta‐analysis showed that LSSS probably reduce DBP slightly at up to 60 months. The 95% CIs of the pooled mean differences did not include clinically meaningful benefit (ranging from 1.36 to 3.50 mmHg lower), as we considered changes in DBP of greater than 5 mmHg to be clinically meaningful due to a 'significant' reduction in stroke risk of approximately 60% in high risk individuals (Thomopoulos 2017). However, small mean reductions for an entire population are more beneficial than very large reductions in only those at high risk (Verbeek 2021). Since the review focussed on the population‐level substitution of regular salt with LSSS, we applied a population perspective and derived a simplified population impact estimate (as described in Appendix 2) of the reduction in DBP observed in our meta‐analysis. This estimate suggested that the observed reduction in DBP corresponded to an estimated 60 (ranging from 35 to 83) stroke deaths prevented per 100,000 persons aged 50 years and older, per year. The observed small, important mean difference in DBP between LSSS and regular salt was confirmed by sensitivity analyses. Substantial heterogeneity was, however, detected in the pooled analysis for this outcome: subgrouping by various characteristics of included studies, participants and interventions suggests there may be no important clinical differences in average effects between the various subgroups.

In adult participants, meta‐analysis showed that LSSS probably reduce SBP slightly at up to 60 months. The 95% CIs of the pooled mean differences did not include clinically meaningful benefit (ranging from 3.50 to 6.01 mmHg lower), as we considered changes of at least 10 mmHg in SBP to be clinically meaningful. This cut‐off was informed by a systematic review with meta‐regression, quantifying the effects of blood pressure reduction on cardiovascular outcomes and death from large‐scale blood‐pressure lowering trials, which indicated that relative risk reductions are proportional to the magnitude of blood‐pressure reduction, with every 10 mmHg reduction in SBP significantly reducing the risk of major cardiovascular disease events (Ettehad 2016).  In addition, the overview and meta‐analysis by Thomopoulos 2017, investigating the effects of blood‐pressure lowering treatment and stratifying participants by total cardiovascular risk, reported a 'significant' stroke reduction of 60% with a 10 mmHg reduction in SBP in individuals at high risk. Our simplified population impact estimate suggested that the observed reduction in SBP with LSSS compared to regular salt corresponds to an estimated 53 (ranging from 40 to 65) stroke deaths prevented per 100,000 persons aged 50 years and older, per year. The observed small, important mean difference in SBP between LSSS and regular salt was broadly confirmed by sensitivity analyses. We explored the substantial heterogeneity detected in the pooled analysis for SBP using subgroup analyses, which suggest there may be no important clinical differences in average effects between the various subgroups.

For the presence of hypertension at 18 months, one trial showed little to no difference between the effects of LSSS and regular salt in adult participants. A stepped‐wedge cluster trial measuring incident hypertension indicated a more pronounced difference, with the hazard ratio for this outcome favouring LSSS.

We do not know whether there is a difference in the number of adult participants per group who achieve blood pressure control as the 95% CI limits of the pooled effect were consistent with the possibility for unimportant and important benefit, and most of the information for this outcome came from a study at unclear overall risk of bias. Furthermore, this study had limited generalisability as it investigated the effect of LSSS comprising 97% sodium chloride (regular salt) and therefore did not represent the composition of the majority of LSSS formulations on the market, most of which contain 65% or less sodium chloride.

We also do not know whether there is a difference in the number of adult participants per group who experience various cardiovascular events. Only 18 of these events were reported in total, including angina, serious cardiovascular events and cardiovascular symptoms, resulting in very imprecise 95% CI limits around the pooled effect. In addition, the pooled effect was driven by a large study in individuals at high risk of future vascular disease, thereby limiting the generalisability of the findings.

In adult participants, meta‐analysis showed that LSSS probably reduce non‐fatal stroke slightly at up to 60 months. The 95% CIs of the pooled risk and rate ratios included unimportant benefit and unimportant harm or no effect (ranging from a RR of 0.80 to 1.01), as we considered relative measures of less than 0.75 or greater than 1.25 to be important or 'appreciable' (Guyatt 2011). The simplified population impact estimate we derived (as described in Appendix 2) suggested that the observed relative risk when LSSS was compared to regular salt corresponds to an estimated 10 non‐fatal strokes prevented (ranging from 21 prevented to 1 caused) per 100,000 persons per year. The observed benefit with LSSS was not reflected in sensitivity analyses; with these instead showing highly imprecise effects of little to no effect, or harm.

Meta‐analysis in adult participants showed that LSSS probably reduce non‐fatal ACS slightly at up to 60 months. The 95% CIs of this effect included important and unimportant benefit (ranging from a rate ratio of 0.52 to 0.94), as we considered relative measures of less than 0.75 or greater than 1.25 to be important. The simplified population impact estimate we derived suggested that the observed relative risk when LSSS was compared to regular salt corresponds to an estimated 50 non‐fatal ACS events prevented (ranging from 10 to 80 prevented) per 100,000 persons per year.

In adult participants, meta‐analysis showed that LSSS probably reduce cardiovascular mortality slightly at up to 60 months. The 95% CIs of the pooled rate ratios included important benefit and no effect (ranging from a rate ratio of 0.60 to 1.00), as we considered relative measures of less than 0.75 or greater than 1.25 to be important.  The simplified population impact estimate we derived suggested that the observed relative risk when LSSS was compared to regular salt corresponds to an estimated 53 cardiovascular deaths prevented (ranging from 92 prevented to none caused or prevented) per 100,000 persons per year. The observed relative effect between LSSS and regular salt was confirmed by sensitivity analysis excluding trials at high risk of overall bias.

We do not know whether there is a difference in the number of stroke deaths per group in adults as the 95% CI limits of the pooled effect were consistent with the possibility for important harm and important benefit. Furthermore, the generalisability of the results to the general population was limited as the pooled effect was driven by a large secondary prevention trial in which 73% of included participants had previously had a stroke.

In adult participants, meta‐analysis showed that LSSS probably increase blood potassium slightly at up to one and a half years. The 95% CIs of the pooled mean differences did not include clinically meaningful changes (ranging from 0.07 to 0.18 mmol/L higher), as we considered changes in blood potassium of greater than 1.0 mmol/L to be clinically meaningful. This is based on the variations around the 'normal' blood potassium levels of 3.6 to 5.0 mmol/L (Cohn 2000), which can cause moderate hyperkalaemia (defined as 6.0 to 6.9 mmol/L; Hollander‐Rodriguez 2006 and Ahee 2000, respectively).

For the presence of hyperkalaemia at up to 60 months, some trials reported no events while others reported hyperkalaemic events in both the LSSS and regular salt groups. In adult participants, meta‐analyses showed that LSSS likely result in little to no difference in hyperkalaemia. The 95% CIs of the pooled risk ratios included important benefit and important harm (ranging from a RR of 0.46 to 2.38), as we considered relative measures of less than 0.75 or greater than 1.25 to be important. Only five trials reported on this outcome, though all information included in the meta‐analysis came from two trials in participants judged to be at possible and unclear risk of hyperkalaemia.

We do not know whether there is a difference in the number of adult participants per group who experience hypokalaemia events as one small trial reporting on this outcome reported zero events in both groups. In addition, this trial was at unclear overall risk of bias, and included only younger participants with hypertension treated with potassium‐sparing diuretics. Consequently, as the rationale for the administration of LSSS was potassium supplementation, we considered the generalisability of the evidence to be limited.

We also do not know whether there is a difference in the number of adult participants per group who experience other adverse events. Most of the information for this outcome was from studies at high or unclear overall risk of bias, events were very sparsely reported (39 in total), and outcomes were too diverse to pool.

#### Low‐sodium salt substitutes versus regular salt or no active intervention in children

Children (mean age 9.5 (SD 4.2) years) allocated to bread containing LSSS had lowered DBP and SBP (reduction of 2.1 mmHg and 5.1 mmHg, respectively) on average. Children (mean age 8.4 (SD 3.5) years) allocated to bread containing regular salt had larger reductions for both DBP and SBP (reduction of 5.87 mmHg and 6.05 mmHg, respectively) on average.

In all participants aged 18 or younger, results from a single included trial showed that the evidence is very uncertain about the effect of LSSS compared to regular salt on change in DBP as well as SBP at four months. The trial contributing to these outcomes was at unclear overall risk of bias, and reported effects with wide 95% CIs including both reductions and increases in DBP and SBP. Furthermore, the intervention was delivered in bread only, thereby limiting generalisability to discretionary use settings.

### Overall completeness and applicability of evidence

Our review made use of a comprehensive search strategy with no language or date restrictions to identify all RCTs and prospective analytical cohort studies assessing the effect of LSSS on cardiovascular health in adults, children and pregnant women in the general population. We searched multiple sources of information for all studies and handsearched three relevant systematic reviews to identify additional studies. We also contacted study authors in cases where we required additional data or information.

We found only one trial, included under Comparison 1 for the effect of LSSS on adults, which additionally reported certain outcomes in children. The sparse evidence in children may be due to the relatively low prevalence of elevated blood pressure in children, with two large systematic reviews conducted in low‐ and middle‐income settings reporting pooled prevalence of 5.5% and 9.8% in children and adolescents from Africa and China, respectively (Noubiap 2017; Wang 2019). A large systematic review and meta‐regression conducted in 122,000 adolescents further indicated that this prevalence was disproportionately affecting adolescents in low‐ and middle‐income countries (De Moraes 2014). Consequently, maximal population‐level effects would not be achieved through targeting children, but rather adults; a group in which the global prevalence of hypertension (defined as a SBP ≥ 140 mmHg and DBP ≥ 90 mmHg) was approximately 32.5% for adults aged 30 years and older in 2019 (NCD Risk Factor Collaboration 2021).

We found no studies assessing the effect of LSSS in pregnant women. While chronic hypertension is a known significant risk factor for pre‐eclampsia (Bilano 2014), mean arterial pressure (MAP) in the first and second trimester has been suggested as a better predictor of pre‐eclampsia than SBP and DBP (Cnossen 2008). This study additionally reported that high MAP before pregnancy can be used as a predictor of pre‐eclampsia (Cnossen 2008) suggesting that the timing of the management of blood pressure is important, and that interventions to lower blood pressure prior to pregnancy might have the most success in avoiding complications for the mother and infant. In addition, pre‐eclampsia is a complex disease involving multiple organ systems (Palei 2013) and risk factors (Bilano 2014), and there is a paucity of evidence to support lifestyle interventions, such as reducing dietary sodium intake, for preventing pre‐eclampsia (Thangaratinam 2011). 

Though our review included a reasonable distribution of studies from low‐ and middle‐income (n = 15) and high‐income countries (n = 12), the majority of trials (n = 15) were conducted in Asian populations. Furthermore, only one included study was conducted in South America; no eligible studies conducted in Africa, Oceania or North America were found. As different populations may use different quantities of discretionary salt, as a proportion of total salt intake, this may have an impact on the degree to which substitution with discretionary LSSS will alter sodium and potassium intakes. Subgroup analyses suggest there may be no important clinical differences in average effects on blood pressure between subgroups by ethnicity, although these analyses were limited. This suggested that the mix of ethnicities included in the review may not systematically bias our pooled estimates to Asian populations. It is more difficult to judge whether evidence from other countries and regions not represented in the review may have changed our pooled estimates. Such potential systematic differences could likely be categorised as biological and behavioural; such categories might plausibly include differences in baseline prevalence, and extent, of hypertension and differences in adherence to LSSS, respectively. Our review included populations with diverse baseline risks of hypertension and diverse baseline 24‐h urinary excretion of sodium and potassium, but subgroup analyses of these factors suggest there may be no important clinical differences in average effects on blood pressure.

Importantly, the findings of subgroup analyses should always be interpreted with caution as these can often be misleading (Deeks 2020). The likelihood of false negative and positive results increase rapidly when numerous subgroup analyses are undertaken and statistical power to find significant differences between subgroups is often lacking (Cuijpers 2021; Deeks 2020). In our review in particular, subgroup analyses were often limited by very few studies or participants contributing information to certain subgroups. As a result, findings from the subgroup analyses in our review may not all be sufficiently robust and should be interpreted with caution and with consideration of the described limitations.

Most of the trials included in our review assessed the effects of LSSS in participants with elevated blood pressure at enrolment. Due to limited data, we could not adequately examine effect modification for the relationship between LSSS use and outcomes by hypertension status.

All trials included in the review specifically excluded participants in whom it is known that an increased intake of potassium could cause harm, for example, people with CKD, type 1 or 2 diabetes mellitus, impaired renal function or those using potassium‐sparing medications. This limits the generalisability of our findings regarding the effects and safety of LSSS to these subpopulations, as well as to settings where a considerable proportion of the population may have undiagnosed conditions rendering increased potassium intake as potentially harmful.

Furthermore, the majority of included trials investigated the implementation of LSSS as a discretionary intervention. This limits the generalisability of our findings to non‐discretionary applications of LSSS, particularly use in condiments, and in manufactured food products or foods sold in restaurants, markets, cafeterias and street vendors.

We did not find evidence for a number of prespecified outcomes in the review. Across comparisons, no studies reported on diabetes mellitus (DM) diagnosis or hyponatraemia. The absence of evidence on hyponatraemia represents a gap in the evidence related to the safety of LSSS. While global sodium intakes are approximately double the WHO recommendation at present, thereby lowering the overall likelihood of hyponatraemia events, the individual risk of this event remains ‐ particularly in older people and those using thiazide diuretics (Filippatos 2017; Upadhyay 2009).

In Comparison 2, a single study reporting on the effects of LSSS in children did not report on hypertension, blood pressure control, hyper‐ or hypokalaemia, changes in blood potassium or adverse events. The paucity of evidence for these outcomes in children is likely due to the low general prevalence of hypertension as well as conditions and risk factors related to blood potassium imbalances.

### Quality of the evidence

The interpretation of many of the trials included in the review is constrained by small sample sizes and considerable loss to follow‐up. Lack of baseline exposure and dietary status, as well as adherence to the allocated intervention, have been identified as factors that undermine the translation of dietary clinical trials into practice (Mirmiran 2021); these were generally sparsely and diversely reported by the included trials in the review. Nine trials included in the review were judged as having low risk of bias overall; 12 were at unclear overall risk of bias.

The pooled estimates of the effect of LSSS interventions compared to regular salt on hypertension, blood pressure control, and stroke mortality were downgraded for imprecision in line with the minimally contextualised approach we used. Imprecision was also identified for the outcomes: various cardiovascular events and other adverse events. These were composite outcomes and studies reporting on them were not designed or powered to detect differences between LSSS and regular salt groups.

The evidence for blood pressure control was considered indirect because it is questionable whether the intervention for the study contributing the most data was sufficiently lower in sodium compared to regular salt (only 3% replacement).

The evidence for hypokalaemia was downgraded for indirectness since the participants included in the single study reporting on this outcome were not sufficiently generalisable, being younger hypertensive adults on potassium‐depleting diuretics.

Indirectness was also identified for all outcomes relating to cardiovascular events and mortality. This was due to most, or all, of the information for these outcomes coming from studies including participants at high risk of cardiovascular disease, or individuals who had already experienced a cardiovascular event; thereby limiting the generalisability of the findings to the general population.

Furthermore, the certainty in pooled estimates for changes in blood pressure was affected by unexplained substantial heterogeneity. This may be due to clinical heterogeneity related to the various ways in which blood pressure measurements are collected in practice; various studies have shown variability between single measures and the mean of consecutive measurements (Burkard 2018), between blood pressure measured in a clinical setting and those obtained from daytime ambulatory measurements (Banegas 2017), and between measurements obtained from aneroid (inflatable cuff) and electronic sphygmomanometers (Shahbabu 2016).

### Potential biases in the review process

The review may be affected by non‐reporting bias. For the unknowns that we are aware of ('known unknowns'), i.e. particular results from a trial not reported in a usable format, we contacted trial authors to request the data in a usable format. In cases where we did not obtain these data and they were consequently excluded, this is a limitation, as we cannot be certain how the inclusion of these data would have affected the pooled estimates. Despite this, we do observe agreements in the pooled mean differences in blood pressure changes reported in other similar reviews on this topic; though the estimated reductions are typically more conservative in our review. As a result, we think it is unlikely that these missing data would have meaningfully changed our pooled estimates. A number of studies (Li 2014; Li 2016; Mu 2009; Zhou 2013) reported allocating entire villages or households to LSSS or regular salt without explicit exclusion of children, though only one study reported separate data for the effect of the intervention in children. It is possible that data from family members aged 18 years or younger who were included as part of a randomised village or household may have changed our pooled estimates for children, though we anticipate that these data would be from a small subset of participants included in these trials. We are unsure whether these missing data would have meaningfully changed our pooled estimates of effect in children.

It is more difficult for us to judge the effect of 'unknown unknowns', i.e. entire eligible studies not detected by our comprehensive search strategy. We acknowledge the possibility that small, unpublished studies may not have been identified and included in the review as the interpretation of funnel plot asymmetry could not definitively rule this out. This was due to similar effect sizes despite varying inter‐trial standard errors for DBP and similar effect sizes as well as standard errors between trials for SBP.

We did not exclude any studies based on the duration of intervention, the formulation of LSSS, or participant characteristics. We did, however, exclude studies with multifactorial designs where the effect of LSSS could not be isolated, though it may have been relevant to the review question. The reason for excluding studies with such multi‐component interventions was that any observed changes in outcomes of interest could not be attributed to LSSS alone.

### Agreements and disagreements with other studies or reviews

Pooled mean differences in blood pressure in our review are in line with previous systematic reviews on the effects of LSSS use in adults. These recent systematic reviews reported reductions in DBP and SBP ranging from 2.00 to 4.04 mmHg and 7.81 to 8.87 mmHg, respectively (Hernandez 2019; Jafarnejad 2020; Jin 2020).

However, it is important to take note of differences between these reviews and our review. One of the reviews included only studies conducted in Chinese study participants (Jin 2020), while another restricted studies to participants with stage 2 hypertension (Jafarnejad 2020). Both of these can be considered limited in their ability to generalise to guidelines for the general population: the review by Jin 2020, through its restriction to an ethno‐geographic group with, according to a recent publication from the China Hypertension Survey, a high prevalence of hypertension (Wang 2018); the review by Jafarnejad 2020, for including only studies in participants with progressive disease (Giles 2009). The inclusion criteria for these reviews consequently resulted in enriched populations in respect of hypertension, which may have influenced treatment effect through an increased number of participants with resistant hypertension (Yaxley 2015). Jafarnejad 2020 reported stratified analyses by several effect modifiers, demonstrating that LSSS use resulted in reductions in SBP and DBP in hypertensive adults of all ages, though reductions in SBP and DBP were slightly more pronounced in hypertensive adults younger than 65 years. Though these results were numerically very similar in the subgroup analyses conducted as part of our review, they were not fully reflected since we found greater reductions in SBP and smaller reductions in DBP in younger participants compared to older participants in the general population. As discussed previously, these differences may be due to differences in the included populations. A large review investigating the effect of antihypertensive medication (therefore including participants with overt hypertension) reported larger reductions in DBP in younger participants when compared to older and very old participants (Guang Wang 2005).

Only one review evaluated the certainty of evidence and presented low‐certainty evidence for reductions of 3.96 mmHg and 7.81 mmHg, in DBP and SBP respectively, at any length of follow‐up (Hernandez 2019). Low‐certainty evidence was also presented for the effect on triglycerides. For other outcomes, Hernandez 2019 concluded, on the basis of moderate‐certainty evidence, that LSSS use probably has little or no effect on mortality, while its effects on detected hypertension, total blood cholesterol, glucose, as well as urinary sodium and potassium excretion were very uncertain. The pooled effect estimates of these outcomes were similar between the review by Hernandez 2019 and our review, despite slight differences in included studies and the exact definitions of outcomes.

## Authors' conclusions

**Implications for practice**

In an adult population, the small pooled mean differences in DBP and SBP favoured LSSS when compared to regular salt. Though these were statistically significant, they were not considered to be clinically important at the individual level (moderate‐certainty evidence). However, small mean reductions from a population‐level intervention can be more beneficial than larger reductions in at‐risk patients (Verbeek 2021). When taking this perspective and considering the general population for which the WHO NUGAG Subgroup on Diet and Health is formulating the guidance, we considered the observed reductions in DBP and SBP when comparing the use of LSSS to regular salt to be small but important from a population perspective. While maximum follow‐up for these outcomes was at 60 months, the majority of trials reporting on blood pressure outcomes followed participants for six months or less.

We observed a slight reduction in the risk ratio for non‐fatal stroke, non‐fatal ACS and cardiovascular mortality, favouring LSSS when compared to regular salt, in our review. Maximal follow‐up for these outcomes was at 60 months. The reduction in non‐fatal stroke and cardiovascular mortality was not considered to be clinically important at the individual level (both moderate‐certainty evidence). The reduction in non‐fatal ACS was considered to be clinically important at the individual level (moderate‐certainty evidence), though variation around the point estimate showed that both clinically important and unimportant benefits are possible. However, these benefits were all considered to be small but important from a population perspective.

Importantly, many of the trials included in our review restricted participants to those with elevated blood pressure or hypertension at enrolment. Therefore, the use of this evidence to inform population‐based public health guidance requires careful consideration as offered by evidence‐informed approaches to guideline development (e.g. the GRADE approach (Zhang 2019)). Our systematic review failed to show that LSSS meaningfully reduces prevalent hypertension when compared to regular salt, with little or no difference in this outcome between the two groups at maximal follow‐up of 18 months; though a large stepped‐wedge cluster trial showed a more pronounced effect on incident hypertension over 30 months. Evidence on blood pressure control, various cardiovascular events, and stroke mortality was limited and we could not draw any conclusions about these outcomes.

Furthermore, our systematic review found no meaningful increase in hyperkalaemia with LSSS when compared to regular salt, with little or no difference in effect for this important safety outcome at maximal follow‐up of five years. While global estimates of potassium intakes (Global Dietary Database 2022) are currently less than levels conditionally recommended by the WHO (WHO 2012b), thereby potentially delaying the onset of hyperkalaemia events, we consider five years likely to be sufficient follow‐up time to detect an event which usually develops over the course of weeks (Hollander‐Rodriguez 2006). It should be noted, however, that the evidence on hyperkalaemia presented in this review has several limitations. Very few studies reported on this important safety outcome, and studies that did report on hyperkalaemia also used variable criteria to define the condition (see Included studies). Most studies included in our review also had strict inclusion and exclusion criteria with regard to hyperkalaemia risk factors. All included trials specifically excluded participants in whom it is known that an increased intake of potassium could cause harm (e.g. people with chronic kidney disease). Only seven trials included participants judged to be at possible risk of hyperkalaemia, and four trials included participants at unclear risk of hyperkalaemia due mostly to limited reporting of the criteria assessed (Table 6). Though only five trials reported on this outcome, two including participants judged to be at possible risk and one including participants at unclear risk, all information in the meta‐analysis came from participants at possible or unclear risk. Therefore, caution should be taken in directly applying the results of our review to the general population, which would likely consist of some people in whom it is known that an increased intake of potassium could cause harm, as well as proportions of people with risk factors for hyperkalaemia, both diagnosed and undiagnosed.

A small pooled mean difference in blood potassium between LSSS and regular salt indicated a small increase in this outcome for the LSSS group; this was not considered to be clinically important (moderate‐certainty evidence) given its impact on the upper end of a 'normal' blood potassium level would not reach levels indicative of moderate hyperkalaemia. Again, these findings should be interpreted with caution given the limited number of studies reporting on this outcome, and the strict exclusion by all studies of participants in whom it is known that an increased intake of potassium could cause harm. Evidence on hypokalaemia events and various adverse effects was limited and we could not draw any conclusions about these safety outcomes.

Nearly all the trials in our review investigated the effects of LSSS implemented as a discretionary intervention. This restricts the generalisability of our findings to discretionary LSSS implementation, and we are unable to draw firm conclusions about non‐discretionary LSSS implementations (for example, where LSSS is added to manufactured foods). Furthermore, the contribution of discretionary salt to total sodium intake varies considerably across countries and settings. This variation is an important consideration when decisions are made about the implementation of LSSS since the absolute intakes of LSSS may vary across settings, thereby creating the possibility of variable impacts on both effectiveness and safety.

It should also be noted that the majority of trials included in our review (24/27) investigated the effects of potassium‐containing LSSS, therefore, we are unable to draw firm conclusions about the effects and safety of LSSS that do not displace sodium with potassium. Variable impacts on effectiveness and safety might also be expected as a result of the potassium content of the LSSS used. In our review, potassium content in potassium‐containing products ranged from 10.1% to 50%. The selection of potassium‐containing LSSS based on potassium content is another important implementation consideration.  

There was sparse evidence for the effect and safety of LSSS in children, with no studies assessing the discretionary use of LSSS in this population group. Given the limited evidence, we could not draw any conclusions about the effect of LSSS on DBP and SBP in children. We could not draw any conclusions about the safety of the use of LSSS in children given that no studies reported on safety outcomes. Given that no studies were found in pregnant women, we also could not draw any conclusions about the effect and safety of LSSS in pregnant women.

Importantly, multi‐component and multi‐sector strategies are usually used to reduce sodium intake, blood pressure and cardiovascular disease risk in populations, and the use of LSSS to reduce sodium intake would be regarded as one of the approaches within these overall strategies.

**Implications for research**

Given the sparse evidence available for the safety and effect of LSSS in children, and the lack of evidence in pregnant women, studies assessing the benefits and potential harms of LSSS in these groups are needed as a priority. In addition, the lack of information on hyponatraemia events in our review indicates that studies assessing this outcome in the context of LSSS use are needed, particularly since older people and those using certain classes of medication used to treat hypertension are disproportionately at risk. 

Given the limitations of the evidence relating to hyperkalaemia, robust studies with explicitly defined measures of this outcome are needed to better understand the safety implications of widespread LSSS use (discretionary and non‐discretionary). In addition, highly monitored trials including participants who may be at risk of hyperkalaemia, or evidence from prospective cohort studies including participants that are representative of the general population, would provide evidence that is more generalisable to widespread population‐level LSSS implementation. Similarly, robust studies assessing a participant population that is representative of the general population in terms of blood pressure status are required to better understand the effectiveness and safety of LSSS in people with normal blood pressure.

Important evidence is still needed to answer the question of whether LSSS use sustainably decreases overall sodium intake, or whether it results in dietary compensation through behavioural modifications; such as, for example, an increased intake of products that are sources of non‐discretionary salt. Studies using reliable measures of dietary sodium and potassium intake, such as 24‐h urinary excretion are needed to assess the extent to which LSSS use reduces sodium intake, and increases potassium intake (when potassium‐containing LSSS are used) over the longer term. Other measures of dietary intake, such as dietary recalls, food frequency questionnaires and spot urine tests, have recently been shown to exhibit poor accuracy in individuals at high cardiovascular risk (Tsirimiagkou 2022). Evidence on the use of LSSS in manufactured and processed foods, particularly sauces and condiments, which represent a large proportion of dietary sodium intake in some regions of the world is also required to understand the extent to which population‐level exclusive replacement of discretionary salt would change global sodium intakes. Finally, studies assessing sodium intakes in populations and quantifying the level and extent of iodisation in LSSS are needed to ensure proactive recalibration of iodisation levels in salt in the context of increased LSSS use. Robust studies examining the effectiveness of multi‐component, multi‐sectoral strategies that include LSSS could further inform decision‐making to reduce sodium intake and cardiovascular disease risk.

The quality and utility of future research on these questions would be optimised by prospective registration of studies, as well as publication of protocols and detailed data analysis plans of future studies. The use of appropriate reporting guidelines (e.g. CONSORT) would also support in the production of high‐quality evidence of maximum utility.

Robust evidence linked to the resource implications of LSSS use is needed to better inform considerations related to population‐level implementation.

## Appendices

### Appendix 1. Appendix 1. Search strategies

**MEDLINE (PubMed)**

Searched: 1946 to 18 August 2021

**#1** ((((((((randomized controlled trial [pt]) OR controlled clinical trial [pt]) OR randomized [tiab]) OR placebo [tiab]) OR clinical trials as topic [mesh: noexp]) OR randomly [tiab]) OR trial [ti])) NOT ((animals [mh] NOT humans [mh])) **#2** "cohort study"[Title/Abstract] OR epidemiologic*[Title/Abstract] OR longitudinal[Title/Abstract] OR "Follow‐up study"[Title/Abstract] OR "Follow up study"[Title/Abstract] OR prospective[Title/Abstract] OR "Observational study"[Title/Abstract] NOT ((animals [mh] NOT humans [mh])) **#3** "adverse effects"[MeSH Subheading] OR "complications"[MeSH Subheading] OR "deficiency"[MeSH Subheading] OR "safe"[Title/Abstract] OR "safety"[Title/Abstract] OR "side effect"[Title/Abstract] OR "side effects"[Title/Abstract] OR "undesirable effect"[Title/Abstract] OR "undesirable effects"[Title/Abstract] OR "treatment emergent"[Title/Abstract] OR "tolerability"[Title/Abstract] OR "toxicity"[Title/Abstract] OR "ADRS"[Title/Abstract] OR ("adverse"[Title/Abstract] AND ("effect"[Title/Abstract] OR "effects"[Title/Abstract] OR "reaction"[Title/Abstract] OR "reactions"[Title/Abstract] OR "event"[Title/Abstract] OR "events"[Title/Abstract] OR "outcome"[Title/Abstract] OR "outcomes"[Title/Abstract])) **#4** #1 OR #2 OR #3 **#5** "salt substitute"[Title/Abstract] OR "salt substitutes"[Title/Abstract] OR "salt substitution"[Title/Abstract] OR "sodium substitute"[Title/Abstract] OR "sodium substitutes"[Title/Abstract] OR "sodium substitution"[Title/Abstract] OR "substituting sodium"[Title/Abstract] OR "sodium chloride substitute"[Title/Abstract] OR "sodium chloride substitutes"[Title/Abstract] OR "sodium chloride substitution"[Title/Abstract] OR "salt alternative"[Title/Abstract] OR "salt alternatives"[Title/Abstract] OR "sodium alternative"[Title/Abstract] OR "low sodium salt"[Title/Abstract] OR "mineral salt"[Title/Abstract] OR "KCl salt"[Title/Abstract] OR "potassium chloride salt"[Title/Abstract] OR "potassium enriched salt"[Title/Abstract] OR "Potassium lactate"[Title/Abstract] OR "magnesium‐enriched salt"[Title/Abstract] OR "magnesium‐enriched salt"[Title/Abstract] OR "sodium replacement"[Title/Abstract] OR "salt replacement"[Title/Abstract] OR "salt replacer"[Title/Abstract] OR "salt replacers"[Title/Abstract] OR "sodium chloride replacement"[Title/Abstract] OR "sodium chloride replacer"[Title/Abstract] **#6 **"Diet, Sodium‐Restricted"[Mesh] **#7 **#5 OR #6 **#8 **#4 AND #7

**Embase (Ovid)**

Searched: 1947 to 18 August 2021

**1** ((salt or sodium) adj1 (substitut* or alternative or replace*)).tw. **2** sodium chloride substitut*.tw. **3** sodium chloride alternative.tw. **4** low* sodium salt.tw. **5** ("mineral salt" or "KCl salt").tw. **6** ("potassium chloride salt" or "potassium enriched salt" or "Potassium lactate").tw. **7** (substitut* or alternative).tw. **8** *sodium chloride/ **9** 7 and 8 **10** *magnesium salt/ **11** (magnesium chloride salt or magnesium enriched salt).tw. **12** reduced sodium salt.tw. **13** *sodium restriction/ **14** 1 or 2 or 3 or 4 or 5 or 6 or 9 or 10 or 11 or 12 or 13 **15** (random* or factorial* or crossover* or cross over or placebo* or (doubl* adj blind*) or (singl* adj blind*) or assign* or allocat* or volunteer*).tw. **16** crossover procedure/ or double blind procedure/ **17** randomized controlled trial/ or single blind procedure/ **18** (rat or rats or mouse or mice or swine or porcine or murine or sheep or lambs or pigs or piglets or rabbit or rabbits or cat or cats or dog or dogs or cattle or bovine or monkey or monkeys or trout or marmoset*).ti. and animal experiment/ **19** Animal experiment/ not (human experiment/ or human/) **20** 18 or 19 **21** 15 or 16 or 17 **22** 21 not 20 **23** 14 and 22 **24** (ae or to).fs. **25** (safe or safety or side effect* or undesirable effect* or treatment emergent or tolerability or toxicity or adrs or (adverse adj2 (effect or effects or reaction or reactions or event or events or outcome or outcomes))).ti,ab. **26** 24 or 25 **27** 26 not 20 **28** 14 and 27 **29** 23 or 28 **30** limit 29 to (conference abstract or conference paper or "conference review") **31** 29 not 30 **32** limit 30 to yr="2019 ‐Current" **33**  31 or 32

**CENTRAL, Cochrane Library**

Searched: Issue 8 of 12, 2021

**#1** MeSH descriptor: [Diet, Sodium‐Restricted] explode all trees **#2** ("salt substitute" OR "salt substitutes" OR "salt substitution" OR "sodium substitute" OR "sodium substitutes" OR "sodium substitution" OR "substituting sodium" OR "sodium chloride substitute" OR "sodium chloride substitutes" OR "sodium chloride substitution" OR "salt alternative" OR "salt alternatives" OR "sodium alternative" OR "low sodium salt" OR "mineral salt" OR "KCl salt" OR "potassium chloride salt" OR "potassium enriched salt" OR "Potassium lactate" OR "magnesium‐enriched salt" OR "magnesium enriched salt" OR "sodium replacement" OR "salt replacement" OR "salt replacer" OR "salt replacers" OR "sodium chloride replacement" OR "sodium chloride replacer"):ti,ab,kw **#3** #1 OR #2 in Trials

**Web of Science Core Collection with Indexes SCI‐Expanded, SSCI, CPCI‐S (Clarivate Analytics)**

Searched: 1970 to 18 August 2021

**#1 **TI=("salt substitute" OR "salt substitutes" OR "salt substitution" OR "sodium substitute" OR "sodium substitutes" OR "sodium substitution" OR "substituting sodium" OR "sodium chloride substitute" OR "sodium chloride substitutes" OR "sodium chloride substitution" OR "salt alternative" OR "salt alternatives" OR "sodium alternative" OR "low sodium salt" OR "mineral salt" OR "KCl salt" OR "potassium chloride salt" OR "potassium enriched salt" OR "Potassium lactate" OR "magnesium‐enriched salt" OR "magnesium‐enriched salt" OR "sodium replacement" OR "salt replacement" OR "salt replacer" OR "salt replacers" OR "sodium chloride replacement" OR "sodium chloride replacer") **#2 **AB=("salt substitute" OR "salt substitutes" OR "salt substitution" OR "sodium substitute" OR "sodium substitutes" OR "sodium substitution" OR "substituting sodium" OR "sodium chloride substitute" OR "sodium chloride substitutes" OR "sodium chloride substitution" OR "salt alternative" OR "salt alternatives" OR "sodium alternative" OR "low sodium salt" OR "mineral salt" OR "KCl salt" OR "potassium chloride salt" OR "potassium enriched salt" OR "Potassium lactate" OR "magnesium‐enriched salt" OR "magnesium‐enriched salt" OR "sodium replacement" OR "salt replacement" OR "salt replacer" OR "salt replacers" OR "sodium chloride replacement" OR "sodium chloride replacer") **#3** #1 OR #2

**Cumulative Index to Nursing and Allied Health Literature (CINAHL) (EBSCOhost)**

Searched: 1937 to 18 August 2021

**S1** MW diet, sodium restricted  **S2** TI "salt substitute" OR "salt substitutes" OR "salt substitution" OR "sodium substitute" OR "sodium substitutes" OR "sodium substitution" OR "substituting sodium" OR "sodium chloride substitute" OR "sodium chloride substitutes" OR "sodium chloride substitution" OR "salt alternative" OR "salt alternatives" OR "sodium alternative" OR "low sodium salt" OR "mineral salt" OR "KCl salt" OR "potassium chloride salt" OR "potassium enriched salt" OR "Potassium lactate" OR "magnesium‐enriched salt" OR "magnesium‐enriched salt" OR "sodium replacement" OR "salt replacement" OR "salt replacer" OR "salt replacers" OR "sodium chloride replacement" OR "sodium chloride replacer"  **S3** AB "salt substitute" OR "salt substitutes" OR "salt substitution" OR "sodium substitute" OR "sodium substitutes" OR "sodium substitution" OR "substituting sodium" OR "sodium chloride substitute" OR "sodium chloride substitutes" OR "sodium chloride substitution" OR "salt alternative" OR "salt alternatives" OR "sodium alternative" OR "low sodium salt" OR "mineral salt" OR "KCl salt" OR "potassium chloride salt" OR "potassium enriched salt" OR "Potassium lactate" OR "magnesium‐enriched salt" OR "magnesium‐enriched salt" OR "sodium replacement" OR "salt replacement" OR "salt replacer" OR "salt replacers" OR "sodium chloride replacement" OR "sodium chloride replacer"  **S4** S2 OR S3  **S5** S1 OR S4 

**ClinicalTrials.gov** (https://clinicaltrials.gov/) 

Searched: 18 August 2021

Studies: All studies Condition or disease: replacement OR replace OR substitute OR substituting OR substitution OR alternative OR reduce OR reduced OR reduction OR lower OR low Other terms: salt OR sodium

**WHO International Clinical Trials Registry Platform (ICTRP)** (https://trialsearch.who.int/) 

Searched: 18 August 2021

salt OR sodium in the title replacement OR replace OR substitute OR substituting OR substitution OR alternative OR reduce OR reduced OR reduction OR lower OR low in the Intervention Recruitment status is ALL

### Appendix 2. Appendix 2. Simplified modelling approach to estimate absolute numbers and population impact

The importance of effects of interventions are best understood as absolute numbers rather than relative numbers. Rating certainty of evidence using GRADE in relation to thresholds (other than no effect) with a minimally contextualised approach, requires the use of absolute numbers (Zeng 2021). This required us to estimate the absolute numbers of events prevented or caused for effectiveness outcomes expressed in relative terms. In addition, since changes in blood pressure are surrogate outcomes for cardiovascular health, we were also required to estimate absolute numbers of events prevented or caused by changes in key surrogate outcomes. We used a simplified model for this, making several simplifying assumptions, as detailed below.

#### Estimating absolute numbers and population impact

To be able to calculate the risk difference, number needed to treat for an additional beneficial outcome (NNTB) or number needed to treat for an additional harmful outcome (NNTH) and corresponding number of events prevented or caused, we needed to know the baseline risk of the disease or event that was being measured or estimated. This baseline risk is central to the impact of population‐level interventions, since small changes in a population with a large risk might have considerable impact. As we were investigating the intervention on a global scale, we used baseline risks from the WHO Global Health Estimates 2019 (WHO 2020) and the Global Burden of Disease Study 2016 global incidence (Institute for Health Metrics and Evaluation 2016) to inform our model. While this approach provided information for the global context, this approach did introduce our **first simplifying assumption**, i.e. that events of interest are homogeneously distributed across the world.

Due to the availability of global baseline risk information and the cardiovascular health focus of this review, the key outcomes to which we applied this approach were: change in diastolic blood pressure (DBP); change in systolic blood pressure (SBP); cardiovascular events: non‐fatal stroke; cardiovascular events: non‐fatal acute coronary syndrome; cardiovascular mortality and stroke mortality. This was only done for adults, since only blood pressure outcomes were reported in children; and age‐specific hazard ratios for blood pressure reductions were not sought for this age group.

##### 1. Approach to estimating population impacts of changes in blood pressure

In order to estimate absolute numbers of events prevented or caused by changes in blood pressure in adults, we first needed to convert changes in blood pressure into corresponding relative risks. This was done by following the approach as described in Verbeek 2021, using age‐specific hazard ratios for stroke and ischaemic heart disease (IHD) in relation to blood pressure reductions (Lewington 2002). Though Lewington 2002 reported hazard ratios for the age categories 40‐49, 50‐59, 60‐69, 70‐79 and 80‐89, we used an average of only the last four categories (i.e. an average of 50‐89); this simple averaging of hazard ratios across age categories was our **second simplifying assumption. **Averaging hazard ratios for categories starting at 50 years instead of 40 years was necessary as the WHO Global Health Estimates 2019 did not report mortality baseline risk for the 40‐49 years category alone. However, a crude analysis suggested that the contribution of stroke mortality in the 30‐49 cohort was less than 1% of the total events reported for people aged 30‐70+. A **third simplifying assumption** was the use of stroke hazard ratios rather than IHD hazard ratios from Lewington 2002, as stroke events were a prespecified outcome of this review.

Once corresponding relative risks were calculated, we used the WHO global estimates of stroke mortality, averaged for the 50‐59, 60‐69 and 70+ age categories, as baseline risk to calculate NNTB/NNTH and corresponding estimates of number of stroke deaths prevented or caused by the change in blood pressure. Therefore, our **fourth simplifying assumption** was that baseline risk of stroke mortality is homogeneously distributed across these age categories.

An example of this approach for the values obtained for change in diastolic blood pressure (DBP) in Comparison 1 (Analysis 1.1)  (mean difference (MD) ‐2.43 mmHg, 95% confidence interval (CI) ‐3.50 to ‐1.36) can be seen in Table 12.

**8:** Worked example of estimated population impact of change in diastolic blood pressure (DBP) in adults

**Model step**	**95% CI (lower) of pooled effect estimate**	**Pooled effect estimate**	**95% CI (upper) of pooled effect estimate**
Hazard ratio for 10 mmHg reduction in DBP ^a^	0.46	0.46	0.46
Observed change from meta‐analysis ^b^	(‐)3.50	(‐)2.43	(‐)1.36
Relative risk for the observed reduction ^c^	0.762	0.828	0.900
Mortality baseline risk for stroke per 1000 ^d^	3.51	3.51	3.51
Risk difference per 1000 ^e^	‐0.835	‐0.604	‐0.352
Number needed to treat for an additional beneficial outcome ^f^	1198	1657	2843
**Number of stroke deaths prevented per 100,000 people aged 50+ years ^g^**	**83**	**60**	**35**

^a^ Source: average of hazard ratios for a 10 mmHg reduction in DBP in four age categories (50‐59, 60‐69, 70‐79, 80‐89) from Lewington 2002 ^b^ Source: effect estimate (mean difference and 95% CI limits) obtained in current review ^c^ Source: calculated from the first two values using the approach by Verbeek 2021 ^d^ Source: WHO Global Health Estimates 2019 (WHO 2020); average of stroke mortality in three age categories (50‐59, 60‐69, 70+) ^e^ Source: calculated using the baseline risk and relative risk for the observed reduction using the approach by Verbeek 2021 ^f^ Source: calculated from the risk difference ^g^ Source: calculated from the number needed to treat for an additional beneficial outcome Abbreviations: CI: confidence interval DBP: diastolic blood pressure

##### 2. Approach to estimating population impacts of changes in cardiovascular events

We used a similar approach to estimating absolute numbers for relative effects, though we did not need to convert these measures using hazard ratios as for blood pressure. Our source data for baseline risk of non‐fatal outcomes were composite.

We used global incidence from the Global Burden of Disease Study 2016 (Institute for Health Metrics and Evaluation 2016), providing the total number of global stroke and acute coronary syndrome (called ischaemic heart disease in this study) events in 2016. To calculate the number of non‐fatal events, we subtracted the number of corresponding cause‐specific mortality outcomes from the WHO Global Health Estimates 2015 (WHO 2020), and divided by the total population at risk in 2015 (e.g. [total number of strokes in 2016 (GBD 2016) – total number of stroke mortality outcomes in 2015 (WHO 2015)]/[total population in 2015 (WHO 2015)]). The 2015 WHO estimates were used as they map closest to GBD 2016 estimates, representing our **fifth simplifying assumption**: i.e., that these two datasets are sufficiently comparable to use in this way. In addition, our **sixth simplifying assumption** was that the numbers estimated for 2015/2016 are still applicable five years later.

An example of this approach for the values obtained for non‐fatal stroke in Comparison 1 (Analysis 1.33) (risk ratio (RR) 0.90 mmHg, 95% CI 0.80 to 1.01) can be seen in Table 13.

**9:** Worked example of estimated population impact of non‐fatal stroke in adults

**Model step**	**95% CI (lower) of pooled effect estimate**	**Pooled effect estimate**	**95% CI (upper) of pooled effect estimate**
Observed change from meta‐analysis ^a^	0.80	0.90	1.01
Event baseline risk for non‐fatal stroke per 1000 ^b^	1.06	1.06	1.06
Risk difference per 1000 ^c^	‐0.212	‐0.106	0.011
Number needed to treat for an additional beneficial/harmful outcome (NNTB/NNTH) ^d^	4717 (NNTB)	9434 (NNTB)	94340 (NNTH)
**Number of non‐fatal strokes prevented or caused per 100,000 people ^e^**	**21 prevented**	**10 prevented**	**1 caused**

^a^ Source: effect estimate (rate ratio and 95% CI limits) obtained in current review ^b^ Source: composite baseline risk calculated from Global Burden of Disease Study 2016 estimate (Institute for Health Metrics and Evaluation 2016) and WHO Global Health Estimates 2015 (WHO 2020) ^c^ Source: calculated using the baseline risk and relative risk for the observed reduction using the approach by Verbeek 2021 ^d^ Source: calculated from the risk difference ^e^ Source: calculated from the number needed to treat for an additional beneficial/harmful outcome Abbreviations: CI: confidence interval NNTB: number needed to treat for an additional beneficial outcome NNTH: number needed to treat for an additional harmful outcome

##### 3. Approach to estimating population impacts of changes in cardiovascular mortality

We used a similar approach to estimating absolute numbers for mortality outcomes to the approach detailed for non‐fatal events. The difference was the use of WHO Global Health Estimates 2019 only, as this dataset provided us with cause‐specific mortality data as well as population numbers. Therefore, we simply divided the number of events for a cause‐specific mortality outcome by the total population to estimate a baseline risk.

For example, the dataset reported 17,863,827 cardiovascular mortality outcomes in the world in 2019 and a total global population of 7.708 billion; corresponding to a 17,863,827/7,708,260,547 = 2.32 baseline risk in 2019.

## Data and analyses

**Comparison 1:** Low‐sodium salt substitutes versus regular salt or no active intervention in adults

Outcome or subgroup title	No. of studies	No. of participants	Statistical method	Effect size
1.1 Change in DBP (mmHg)	19	20830	Mean Difference (IV, Random, 95% CI)	‐2.43 [‐3.50, ‐1.36]
1.2 Change in DBP (mmHg); subgroup study duration	19	20830	Mean Difference (IV, Random, 95% CI)	‐2.43 [‐3.50, ‐1.36]
1.2.1 ≤ 3 months	8	1631	Mean Difference (IV, Random, 95% CI)	‐2.54 [‐3.77, ‐1.31]
1.2.2 > 3 to 12 months	6	995	Mean Difference (IV, Random, 95% CI)	‐2.38 [‐4.79, 0.03]
1.2.3 > 12 months	5	18204	Mean Difference (IV, Random, 95% CI)	‐1.73 [‐3.24, ‐0.22]
1.3 Change in DBP (mmHg); subgroup age	19	20830	Mean Difference (IV, Random, 95% CI)	‐2.43 [‐3.50, ‐1.36]
1.3.1 ≥ 65 years	3	14862	Mean Difference (IV, Random, 95% CI)	‐3.16 [‐5.67, ‐0.64]
1.3.2 < 65 years	14	2906	Mean Difference (IV, Random, 95% CI)	‐2.36 [‐3.44, ‐1.28]
1.3.3 Unknown	2	3062	Mean Difference (IV, Random, 95% CI)	‐0.55 [‐2.05, 0.94]
1.4 Change in DBP (mmHg); subgroup gender	19	20830	Mean Difference (IV, Random, 95% CI)	‐2.43 [‐3.50, ‐1.36]
1.4.1 Mixed	17	17768	Mean Difference (IV, Random, 95% CI)	‐2.69 [‐3.85, ‐1.54]
1.4.2 Unknown	2	3062	Mean Difference (IV, Random, 95% CI)	‐0.55 [‐2.05, 0.94]
1.5 Change in DBP (mmHg); subgroup ethnicity	19	20830	Mean Difference (IV, Random, 95% CI)	‐2.43 [‐3.50, ‐1.36]
1.5.1 Asian	10	20115	Mean Difference (IV, Random, 95% CI)	‐1.72 [‐2.64, ‐0.80]
1.5.2 Conducted in Europe (ethnicity unspecified)	8	693	Mean Difference (IV, Random, 95% CI)	‐3.28 [‐4.76, ‐1.79]
1.5.3 Mixed	1	22	Mean Difference (IV, Random, 95% CI)	‐4.90 [‐11.87, 2.07]
1.6 Change in DBP (mmHg); subgroup BMI	19	20830	Mean Difference (IV, Random, 95% CI)	‐2.43 [‐3.50, ‐1.36]
1.6.1 Normal (18.5 to 24.9 kg/m^2^ for adult Europids, 18.5 to 22.9 kg/m^2^ for adult Asians)	1	185	Mean Difference (IV, Random, 95% CI)	‐3.50 [‐7.59, 0.59]
1.6.2 Overweight (25 to 29.9 kg/m^2^ for adult Europids, 23 to 24.9 kg/m^2^ for adult Asians)	9	15814	Mean Difference (IV, Random, 95% CI)	‐2.58 [‐4.07, ‐1.09]
1.6.3 Obese (≥ 30 kg/m^2^ for adult Europids, ≥ 25 kg/m^2^ for adult Asians)	3	964	Mean Difference (IV, Random, 95% CI)	‐2.75 [‐6.60, 1.10]
1.6.4 Unknown	6	3867	Mean Difference (IV, Random, 95% CI)	‐1.88 [‐3.41, ‐0.35]
1.7 Change in DBP (mmHg); subgroup blood pressure status	19	20830	Mean Difference (IV, Random, 95% CI)	‐2.39 [‐3.40, ‐1.39]
1.7.1 Participants with hypertension	12	1847	Mean Difference (IV, Random, 95% CI)	‐2.80 [‐4.22, ‐1.37]
1.7.2 Participants with normal blood pressure	4	561	Mean Difference (IV, Random, 95% CI)	‐2.87 [‐5.93, 0.20]
1.7.3 Unknown	2	3062	Mean Difference (IV, Random, 95% CI)	‐0.55 [‐2.05, 0.94]
1.7.4 Mixed	3	15360	Mean Difference (IV, Random, 95% CI)	‐2.07 [‐4.09, ‐0.05]
1.8 Change in DBP (mmHg); subgroup LSSS implementation	19	20830	Mean Difference (IV, Random, 95% CI)	‐2.43 [‐3.50, ‐1.36]
1.8.1 Discretionary only	16	20598	Mean Difference (IV, Random, 95% CI)	‐2.11 [‐3.00, ‐1.23]
1.8.2 Non‐discretionary only	1	90	Mean Difference (IV, Random, 95% CI)	‐0.12 [‐2.81, 2.57]
1.8.3 Discretionary and non‐discretionary	2	142	Mean Difference (IV, Random, 95% CI)	‐4.10 [‐4.56, ‐3.64]
1.9 Change in DBP (mmHg); subgroup type of LSSS	19	20830	Mean Difference (IV, Random, 95% CI)	‐2.43 [‐3.50, ‐1.36]
1.9.1 ≥ 30% KCl	8	16279	Mean Difference (IV, Random, 95% CI)	‐2.35 [‐3.86, ‐0.84]
1.9.2 < 30% KCl	8	4285	Mean Difference (IV, Random, 95% CI)	‐1.72 [‐3.01, ‐0.43]
1.9.3 Non‐potassium containing LSSS	2	81	Mean Difference (IV, Random, 95% CI)	‐6.06 [‐9.15, ‐2.96]
1.9.4 Unknown	1	185	Mean Difference (IV, Random, 95% CI)	‐3.50 [‐7.59, 0.59]
1.10 Change in DBP (mmHg); subgroup baseline sodium excretion (mmol/24‐h)	19	20830	Mean Difference (IV, Random, 95% CI)	‐2.43 [‐3.50, ‐1.36]
1.10.1 High baseline 24‐h sodium excretion (≥ 172 mmol Na/24‐h, 3.95 g Na/24‐h or 9.88 g NaCl/24‐h)	3	15087	Mean Difference (IV, Random, 95% CI)	‐2.41 [‐4.76, ‐0.05]
1.10.2 Low baseline 24‐h sodium excretion (< 172 mmol Na/24‐h, 3.95 g Na/24‐h or 9.88 g NaCl/24‐h)	8	847	Mean Difference (IV, Random, 95% CI)	‐3.07 [‐4.83, ‐1.32]
1.10.3 Unknown/Not 24‐h	8	4896	Mean Difference (IV, Random, 95% CI)	‐1.73 [‐2.93, ‐0.52]
1.11 Change in DBP (mmHg); subgroup baseline potassium excretion (mmol/24‐h)	19	20830	Mean Difference (IV, Random, 95% CI)	‐2.43 [‐3.50, ‐1.36]
1.11.1 High baseline 24‐h potassium excretion (≥ 59 mmol K/24‐h or 2.3 g K/24‐h)	8	693	Mean Difference (IV, Random, 95% CI)	‐3.28 [‐4.76, ‐1.79]
1.11.2 Low baseline 24‐h potassium excretion (< 59 mmol K/24‐h or 2.3 g K/24‐h)	3	15241	Mean Difference (IV, Random, 95% CI)	‐1.99 [‐3.72, ‐0.27]
1.11.3 Unknown/Not 24‐h	8	4896	Mean Difference (IV, Random, 95% CI)	‐1.73 [‐2.93, ‐0.52]
1.12 Change in DBP (mmHg); sensitivity analysis: study quality	14	19530	Mean Difference (IV, Random, 95% CI)	‐2.36 [‐3.37, ‐1.34]
1.13 Change in DBP (mmHg); sensitivity analysis: study design	12	1816	Mean Difference (IV, Random, 95% CI)	‐3.45 [‐4.64, ‐2.25]
1.14 Change in DBP (mmHg) stepped‐wedge trial	1		Mean Difference (IV, Fixed, 95% CI)	‐0.76 [‐1.39, ‐0.13]
1.15 Change in SBP (mmHg)	20	21414	Mean Difference (IV, Random, 95% CI)	‐4.76 [‐6.01, ‐3.50]
1.16 Change in SBP (mmHg); subgroup study duration	20	21414	Mean Difference (IV, Random, 95% CI)	‐4.76 [‐6.01, ‐3.50]
1.16.1 ≤ 3 months	8	1631	Mean Difference (IV, Random, 95% CI)	‐5.92 [‐8.20, ‐3.64]
1.16.2 > 3 to 12 months	7	1580	Mean Difference (IV, Random, 95% CI)	‐4.02 [‐6.45, ‐1.59]
1.16.3 > 12 months	5	18203	Mean Difference (IV, Random, 95% CI)	‐4.35 [‐6.68, ‐2.01]
1.17 Change in SBP (mmHg); subgroup age	20	21414	Mean Difference (IV, Random, 95% CI)	‐4.76 [‐6.01, ‐3.50]
1.17.1 ≥ 65 years	3	14862	Mean Difference (IV, Random, 95% CI)	‐4.66 [‐6.15, ‐3.18]
1.17.2 < 65 years	15	3491	Mean Difference (IV, Random, 95% CI)	‐5.08 [‐6.83, ‐3.34]
1.17.3 Unknown	2	3061	Mean Difference (IV, Random, 95% CI)	‐4.17 [‐10.91, 2.57]
1.18 Change in SBP (mmHg); subgroup gender	20	21414	Mean Difference (IV, Random, 95% CI)	‐4.76 [‐6.01, ‐3.50]
1.18.1 Mixed	18	18353	Mean Difference (IV, Random, 95% CI)	‐4.90 [‐6.20, ‐3.60]
1.18.2 Unknown	2	3061	Mean Difference (IV, Random, 95% CI)	‐4.17 [‐10.91, 2.57]
1.19 Change in SBP (mmHg); subgroup ethnicity	20	21414	Mean Difference (IV, Random, 95% CI)	‐4.76 [‐6.01, ‐3.50]
1.19.1 Asian	11	20699	Mean Difference (IV, Random, 95% CI)	‐4.08 [‐5.46, ‐2.71]
1.19.2 Conducted in Europe (ethnicity unspecified)	8	693	Mean Difference (IV, Random, 95% CI)	‐5.75 [‐8.35, ‐3.15]
1.19.3 Mixed	1	22	Mean Difference (IV, Random, 95% CI)	‐9.18 [‐18.47, 0.11]
1.20 Change in SBP (mmHg); subgroup BMI	20	21414	Mean Difference (IV, Random, 95% CI)	‐4.76 [‐6.01, ‐3.50]
1.20.1 Normal (18.5 to 24.9 kg/m^2^ for adult Europids, 18.5 to 22.9 kg/m^2^ for adult Asians)	1	185	Mean Difference (IV, Random, 95% CI)	‐6.60 [‐11.42, ‐1.78]
1.20.2 Overweight (25 to 29.9 kg/m^2^ for adult Europids, 23 to 24.9 kg/m^2^ for adult Asians)	9	15814	Mean Difference (IV, Random, 95% CI)	‐4.72 [‐5.97, ‐3.48]
1.20.3 Obese (≥ 30 kg/m^2^ for adult Europids, ≥ 25 kg/m^2^ for adult Asians)	4	1549	Mean Difference (IV, Random, 95% CI)	‐4.41 [‐7.61, ‐1.21]
1.20.4 Unknown	6	3866	Mean Difference (IV, Random, 95% CI)	‐5.01 [‐8.39, ‐1.64]
1.21 Change in SBP (mmHg); subgroup blood pressure status	20	21414	Mean Difference (IV, Random, 95% CI)	‐4.73 [‐5.92, ‐3.55]
1.21.1 Participants with hypertension	12	1847	Mean Difference (IV, Random, 95% CI)	‐5.33 [‐6.82, ‐3.83]
1.21.2 Participants with normal blood pressure	4	561	Mean Difference (IV, Random, 95% CI)	‐4.50 [‐9.14, 0.14]
1.21.3 Unknown	2	3061	Mean Difference (IV, Random, 95% CI)	‐4.17 [‐10.91, 2.57]
1.21.4 Mixed	4	15945	Mean Difference (IV, Random, 95% CI)	‐3.80 [‐5.34, ‐2.26]
1.22 Change in SBP (mmHg); subgroup LSSS implementation	20	21414	Mean Difference (IV, Random, 95% CI)	‐4.76 [‐6.01, ‐3.50]
1.22.1 Discretionary only	17	21182	Mean Difference (IV, Random, 95% CI)	‐5.02 [‐6.48, ‐3.55]
1.22.2 Non‐discretionary only	1	90	Mean Difference (IV, Random, 95% CI)	0.73 [‐3.85, 5.31]
1.22.3 Discretionary and non‐discretionary	2	142	Mean Difference (IV, Random, 95% CI)	‐5.12 [‐5.85, ‐4.39]
1.23 Change in SBP (mmHg); subgroup type of LSSS	20	21414	Mean Difference (IV, Random, 95% CI)	‐4.76 [‐6.01, ‐3.50]
1.23.1 ≥ 30% KCl	8	16279	Mean Difference (IV, Random, 95% CI)	‐4.65 [‐5.96, ‐3.33]
1.23.2 < 30% KCl	9	4869	Mean Difference (IV, Random, 95% CI)	‐3.45 [‐5.18, ‐1.72]
1.23.3 Non‐potassium containing LSSS	2	81	Mean Difference (IV, Random, 95% CI)	‐9.31 [‐14.23, ‐4.40]
1.23.4 Unknown	1	185	Mean Difference (IV, Random, 95% CI)	‐6.60 [‐11.42, ‐1.78]
1.24 Change in SBP (mmHg); subgroup baseline sodium excretion (mmol/24‐h)	20	21414	Mean Difference (IV, Random, 95% CI)	‐4.76 [‐6.01, ‐3.50]
1.24.1 High baseline 24‐h sodium excretion (≥ 172 mmol Na/24‐h, 3.95 g Na/24‐h or 9.88 g NaCl/24‐h)	3	15087	Mean Difference (IV, Random, 95% CI)	‐4.02 [‐5.76, ‐2.29]
1.24.2 Low baseline 24‐h sodium excretion (< 172 mmol Na/24‐h, 3.95 g Na/24‐h or 9.88 g NaCl/24‐h)	8	847	Mean Difference (IV, Random, 95% CI)	‐5.64 [‐7.65, ‐3.63]
1.24.3 Unknown/Not 24‐h	9	5480	Mean Difference (IV, Random, 95% CI)	‐4.16 [‐5.94, ‐2.38]
1.25 Change in SBP (mmHg); subgroup baseline potassium excretion (mmol/24‐h)	20	21414	Mean Difference (IV, Random, 95% CI)	‐4.76 [‐6.01, ‐3.50]
1.25.1 High baseline 24‐h potassium excretion (≥ 59 mmol K/24‐h or 2.3 g K/24‐h)	8	693	Mean Difference (IV, Random, 95% CI)	‐5.75 [‐8.35, ‐3.15]
1.25.2 Low baseline 24‐h potassium excretion (< 59 mmol K/24‐h or 2.3 g K/24‐h)	3	15241	Mean Difference (IV, Random, 95% CI)	‐4.27 [‐5.65, ‐2.88]
1.25.3 Unknown/Not 24‐h	9	5480	Mean Difference (IV, Random, 95% CI)	‐4.16 [‐5.94, ‐2.38]
1.26 Change in SBP (mmHg); sensitivity analysis: study quality	15	20114	Mean Difference (IV, Random, 95% CI)	‐4.72 [‐6.21, ‐3.24]
1.27 Change in SBP (mmHg); sensitivity analysis: study design	13	2401	Mean Difference (IV, Random, 95% CI)	‐6.03 [‐7.22, ‐4.84]
1.28 Change in SBP (mmHg), stepped‐wedge trial	1		Mean Difference (IV, Fixed, 95% CI)	‐1.29 [‐2.17, ‐0.41]
1.29 Hypertension (as reported, or SBP > 140 mmHg or DBP > 85 mmHg)	1	2566	Risk Ratio (M‐H, Fixed, 95% CI)	0.97 [0.90, 1.03]
1.30 Hypertension (as reported, or SBP > 140 mmHg or DBP > 85 mmHg), stepped‐wedge trial	1		Hazard Ratio (IV, Fixed, 95% CI)	0.45 [0.31, 0.65]
1.31 Blood pressure control (achieving blood pressure threshold or 'control' as prespecified)	2	253	Risk Ratio (M‐H, Random, 95% CI)	2.12 [1.32, 3.41]
1.32 Cardiovascular events: various events	5	982	Risk Ratio (M‐H, Random, 95% CI)	1.22 [0.49, 3.04]
1.33 Cardiovascular events: non‐fatal stroke	3	21250	Risk Ratio (IV, Random, 95% CI)	0.90 [0.80, 1.01]
1.34 Cardiovascular events: non‐fatal stroke; sensitivity analysis: study quality	2	21220	Risk Ratio (IV, Random, 95% CI)	1.03 [0.48, 2.20]
1.35 Cardiovascular events: non‐fatal stroke; sensitivity analysis: study design	2	255	Risk Ratio (IV, Random, 95% CI)	1.27 [0.13, 12.11]
1.36 Cardiovascular events: non‐fatal acute coronary syndrome	1	20995	Rate Ratio (IV, Fixed, 95% CI)	0.70 [0.52, 0.94]
1.37 Cardiovascular mortality	3	23200	Rate Ratio (IV, Random, 95% CI)	0.77 [0.60, 1.00]
1.38 Cardiovascular mortality; sensitivity analysis: study quality	2	21423	Rate Ratio (IV, Random, 95% CI)	0.87 [0.79, 0.95]
1.39 Stroke mortality	2	21423	Rate Ratio (IV, Random, 95% CI)	0.64 [0.33, 1.25]
1.40 Change in blood potassium (mmol/L)	6	784	Mean Difference (IV, Random, 95% CI)	0.12 [0.07, 0.18]
1.41 Change in blood potassium (mmol/L); subgroup risk of hyperkalaemia	6	784	Mean Difference (IV, Random, 95% CI)	0.12 [0.07, 0.18]
1.41.1 Adults not at risk of hyperkalaemia	4	141	Mean Difference (IV, Random, 95% CI)	0.16 [0.06, 0.26]
1.41.2 Adults at possible risk of hyperkalaemia	1	88	Mean Difference (IV, Random, 95% CI)	0.12 [‐0.01, 0.25]
1.41.3 Adults at unclear risk of hyperkalaemia	1	555	Mean Difference (IV, Random, 95% CI)	0.10 [0.02, 0.18]
1.42 Change in blood potassium (mmol/L); sensitivity analysis: study quality	4	141	Mean Difference (IV, Random, 95% CI)	0.16 [0.06, 0.26]
1.43 Change in blood potassium (mmol/L); sensitivity analysis: study design	5	229	Mean Difference (IV, Random, 95% CI)	0.14 [0.07, 0.22]
1.44 Hyperkalaemia (> 5.5 mmol/L, or as reported by study authors)	5	22849	Risk Ratio (M‐H, Random, 95% CI)	1.04 [0.46, 2.38]
1.45 Hyperkalaemia (> 5.5 mmol/L, or as reported by study authors); subgroup risk of hyperkalaemia	5	22849	Risk Ratio (M‐H, Random, 95% CI)	1.04 [0.46, 2.38]
1.45.1 Adults not at risk of hyperkalaemia	2	823	Risk Ratio (M‐H, Random, 95% CI)	Not estimable
1.45.2 Adults at possible risk of hyperkalaemia	2	21471	Risk Ratio (M‐H, Random, 95% CI)	1.00 [0.06, 15.97]
1.45.3 Adults at unclear risk of hyperkalaemia	1	555	Risk Ratio (M‐H, Random, 95% CI)	1.05 [0.44, 2.49]
1.46 Hyperkalaemia (> 5.5 mmol/L, or as reported by study authors); sensitivity analysis: study quality	4	22294	Risk Ratio (M‐H, Random, 95% CI)	1.00 [0.06, 15.97]
1.47 Hyperkalaemia (> 5.5 mmol/L, or as reported by study authors); sensitivity analysis: study design	2	1084	Risk Ratio (M‐H, Random, 95% CI)	Not estimable
1.48 Hypokalaemia (< 3.5 mmol/L, or as reported by study authors)	1	22	Risk Ratio (M‐H, Fixed, 95% CI)	Not estimable
1.49 All‐cause mortality	5	24005	Risk Ratio (IV, Random, 95% CI)	0.89 [0.83, 0.95]
1.50 Adverse events: other	8		Risk Ratio (M‐H, Random, 95% CI)	Totals not selected
1.51 Adverse events: other; subgroup risk of hyperkalaemia	8		Risk Ratio (M‐H, Random, 95% CI)	Totals not selected
1.51.1 Adults not at risk of hyperkalaemia	5		Risk Ratio (M‐H, Random, 95% CI)	Totals not selected
1.51.2 Adults at possible risk of hyperkalaemia	3		Risk Ratio (M‐H, Random, 95% CI)	Totals not selected
1.52 Antihypertensive medication use	4	3301	Risk Ratio (M‐H, Random, 95% CI)	0.80 [0.67, 0.95]
1.53 Antihypertensive medication use; subgroup study duration	4	3301	Risk Ratio (M‐H, Random, 95% CI)	0.80 [0.67, 0.95]
1.53.1 ≤ 3 months	1	213	Risk Ratio (M‐H, Random, 95% CI)	0.75 [0.54, 1.06]
1.53.2 > 3 to 12 months	1	193	Risk Ratio (M‐H, Random, 95% CI)	0.86 [0.70, 1.07]
1.53.3 > 12 months	2	2895	Risk Ratio (M‐H, Random, 95% CI)	0.75 [0.50, 1.12]
1.54 Antihypertensive medication use; subgroup age	4	3301	Risk Ratio (M‐H, Random, 95% CI)	0.80 [0.67, 0.95]
1.54.1 < 65 years	3	735	Risk Ratio (M‐H, Random, 95% CI)	0.74 [0.59, 0.93]
1.54.2 Unknown	1	2566	Risk Ratio (M‐H, Random, 95% CI)	0.91 [0.78, 1.06]
1.55 Antihypertensive medication use; subgroup gender	4	3301	Risk Ratio (M‐H, Random, 95% CI)	0.80 [0.67, 0.95]
1.55.1 Mixed	3	735	Risk Ratio (M‐H, Random, 95% CI)	0.74 [0.59, 0.93]
1.55.2 Unknown	1	2566	Risk Ratio (M‐H, Random, 95% CI)	0.91 [0.78, 1.06]
1.56 Antihypertensive medication use; subgroup BMI	4	3301	Risk Ratio (M‐H, Random, 95% CI)	0.80 [0.67, 0.95]
1.56.1 Overweight (25 to 29.9 kg/m^2^ for adult Europids, 23 to 24.9 kg/m^2^ for adult Asians)	1	213	Risk Ratio (M‐H, Random, 95% CI)	0.75 [0.54, 1.06]
1.56.2 Obese (≥ 30 kg/m^2^ for adult Europids, ≥ 25 kg/m^2^ for adult Asians)	2	522	Risk Ratio (M‐H, Random, 95% CI)	0.73 [0.50, 1.05]
1.56.3 Unknown	1	2566	Risk Ratio (M‐H, Random, 95% CI)	0.91 [0.78, 1.06]
1.57 Antihypertensive medication use; subgroup blood pressure status	4	3301	Risk Ratio (M‐H, Random, 95% CI)	0.80 [0.67, 0.95]
1.57.1 Participants with hypertension	2	406	Risk Ratio (M‐H, Random, 95% CI)	0.83 [0.69, 0.99]
1.57.2 Unknown	1	2566	Risk Ratio (M‐H, Random, 95% CI)	0.91 [0.78, 1.06]
1.57.3 Mixed	1	329	Risk Ratio (M‐H, Random, 95% CI)	0.60 [0.45, 0.80]
1.58 Change in BMI (kg/m^2^)	4		Mean Difference (IV, Random, 95% CI)	Totals not selected
1.59 Change in serum creatinine (µmol/L)	3	616	Mean Difference (IV, Random, 95% CI)	2.56 [‐0.59, 5.71]
1.60 Change in serum creatinine (µmol/L); subgroup risk of hyperkalaemia	3	616	Mean Difference (IV, Random, 95% CI)	2.56 [‐0.59, 5.71]
1.60.1 Adults not at risk of hyperkalaemia	2	61	Mean Difference (IV, Random, 95% CI)	1.64 [‐3.90, 7.18]
1.60.2 Adults at unclear risk of hyperkalaemia	1	555	Mean Difference (IV, Random, 95% CI)	3.00 [‐0.83, 6.83]
1.61 Microalbuminuria	2	2382	Risk Ratio (M‐H, Random, 95% CI)	0.67 [0.53, 0.84]
1.62 Macroalbuminuria	1	1903	Risk Ratio (M‐H, Fixed, 95% CI)	0.48 [0.16, 1.39]
1.63 Change in urinary albumin‐to‐creatinine ratio (uACR)	1	1903	Mean Difference (IV, Fixed, 95% CI)	‐1.68 [‐2.87, ‐0.49]
1.64 Change in fasting blood glucose (mmol/L)	2		Mean Difference (IV, Random, 95% CI)	Totals not selected
1.65 Change in blood triglycerides (mmol/L)	5	420	Mean Difference (IV, Random, 95% CI)	‐0.11 [‐0.91, 0.69]
1.66 Change in blood triglycerides (mmol/L); subgroup study duration	5	420	Mean Difference (IV, Random, 95% CI)	‐0.11 [‐0.91, 0.69]
1.66.1 ≤ 3 months	2	63	Mean Difference (IV, Random, 95% CI)	‐0.34 [‐0.77, 0.08]
1.66.2 > 3 to 12 months	3	357	Mean Difference (IV, Random, 95% CI)	‐0.12 [‐1.64, 1.39]
1.67 Change in blood triglycerides (mmol/L); subgroup age	5	420	Mean Difference (IV, Random, 95% CI)	‐0.11 [‐0.91, 0.69]
1.67.1 ≥ 65 years	2	289	Mean Difference (IV, Random, 95% CI)	‐0.45 [‐0.90, ‐0.00]
1.67.2 < 65 years	3	131	Mean Difference (IV, Random, 95% CI)	‐0.01 [‐1.18, 1.15]
1.68 Change in blood triglycerides (mmol/L); subgroup ethnicity	5	420	Mean Difference (IV, Random, 95% CI)	‐0.11 [‐0.91, 0.69]
1.68.1 Asian	2	289	Mean Difference (IV, Random, 95% CI)	‐0.45 [‐0.90, ‐0.00]
1.68.2 Conducted in Europe (ethnicity unspecified)	2	109	Mean Difference (IV, Random, 95% CI)	‐0.41 [‐3.36, 2.54]
1.68.3 Mixed	1	22	Mean Difference (IV, Random, 95% CI)	‐0.04 [‐0.74, 0.66]
1.69 Change in blood triglycerides (mmol/L); subgroup BMI	5	420	Mean Difference (IV, Random, 95% CI)	‐0.11 [‐0.91, 0.69]
1.69.1 Overweight (25 to 29.9 kg/m^2^ for adult Europids, 23 to 24.9 kg/m^2^ for adult Asians)	4	398	Mean Difference (IV, Random, 95% CI)	‐0.19 [‐1.26, 0.89]
1.69.2 Obese (≥ 30 kg/m^2^ for adult Europids, ≥ 25 kg/m^2^ for adult Asians)	1	22	Mean Difference (IV, Random, 95% CI)	‐0.04 [‐0.74, 0.66]
1.70 Change in blood triglycerides (mmol/L); subgroup blood pressure status	5	420	Mean Difference (IV, Random, 95% CI)	‐0.06 [‐0.68, 0.56]
1.70.1 Participants with hypertension	3	167	Mean Difference (IV, Random, 95% CI)	‐0.53 [‐1.76, 0.71]
1.70.2 Participants with normal blood pressure	3	253	Mean Difference (IV, Random, 95% CI)	0.13 [‐0.68, 0.95]
1.71 Change in blood triglycerides (mmol/L); subgroup LSSS implementation	5	420	Mean Difference (IV, Random, 95% CI)	‐0.11 [‐0.91, 0.69]
1.71.1 Discretionary only	3	289	Mean Difference (IV, Random, 95% CI)	‐0.38 [‐1.33, 0.57]
1.71.2 Non‐discretionary only	1	90	Mean Difference (IV, Random, 95% CI)	0.93 [0.43, 1.43]
1.71.3 Discretionary and non‐discretionary	1	41	Mean Difference (IV, Random, 95% CI)	‐0.50 [‐0.97, ‐0.03]
1.72 Change in blood triglycerides (mmol/L); subgroup type of LSSS	5	420	Mean Difference (IV, Random, 95% CI)	‐0.11 [‐0.91, 0.69]
1.72.1 ≥ 30% KCl	3	289	Mean Difference (IV, Random, 95% CI)	‐0.38 [‐1.33, 0.57]
1.72.2 < 30% KCl	2	131	Mean Difference (IV, Random, 95% CI)	0.21 [‐1.19, 1.62]
1.73 Change in blood triglycerides (mmol/L); subgroup baseline sodium excretion (mmol/24‐h)	5	420	Mean Difference (IV, Random, 95% CI)	‐0.11 [‐0.91, 0.69]
1.73.1 High baseline 24‐h sodium excretion (≥ 172 mmol Na/24‐h, 3.95 g Na/24‐h or 9.88 g NaCl/24‐h)	2	289	Mean Difference (IV, Random, 95% CI)	‐0.45 [‐0.90, ‐0.00]
1.73.2 Low baseline 24‐h sodium excretion (< 172 mmol Na/24‐h, 3.95 g Na/24‐h or 9.88 g NaCl/24‐h)	2	109	Mean Difference (IV, Random, 95% CI)	‐0.41 [‐3.36, 2.54]
1.73.3 Unknown/Not 24‐h	1	22	Mean Difference (IV, Random, 95% CI)	‐0.04 [‐0.74, 0.66]
1.74 Change in blood triglycerides (mmol/L); subgroup baseline potassium excretion (mmol/24‐h)	5	420	Mean Difference (IV, Random, 95% CI)	‐0.11 [‐0.91, 0.69]
1.74.1 High baseline 24‐h potassium excretion (≥ 59 mmol K/24‐h or 2.3 g K/24‐h)	2	109	Mean Difference (IV, Random, 95% CI)	‐0.41 [‐3.36, 2.54]
1.74.2 Low baseline 24‐h potassium excretion (< 59 mmol K/24‐h or 2.3 g K/24‐h)	2	289	Mean Difference (IV, Random, 95% CI)	‐0.45 [‐0.90, ‐0.00]
1.74.3 Unknown/Not 24‐h	1	22	Mean Difference (IV, Random, 95% CI)	‐0.04 [‐0.74, 0.66]
1.75 Change in total blood cholesterol (mmol/L)	6	509	Mean Difference (IV, Random, 95% CI)	‐0.31 [‐0.74, 0.12]
1.76 Change in total blood cholesterol (mmol/L); subgroup study duration	6	509	Mean Difference (IV, Random, 95% CI)	‐0.31 [‐0.74, 0.12]
1.76.1 ≤ 3 months	2	63	Mean Difference (IV, Random, 95% CI)	‐0.41 [‐0.76, ‐0.05]
1.76.2 > 3 to 12 months	4	446	Mean Difference (IV, Random, 95% CI)	‐0.28 [‐0.88, 0.32]
1.77 Change in total blood cholesterol (mmol/L); subgroup age	6	509	Mean Difference (IV, Random, 95% CI)	‐0.31 [‐0.74, 0.12]
1.77.1 ≥ 65 years	3	378	Mean Difference (IV, Random, 95% CI)	0.00 [‐0.52, 0.52]
1.77.2 < 65 years	3	131	Mean Difference (IV, Random, 95% CI)	‐0.64 [‐1.31, 0.02]
1.78 Change in total blood cholesterol (mmol/L); subgroup ethnicity	6	509	Mean Difference (IV, Random, 95% CI)	‐0.31 [‐0.74, 0.12]
1.78.1 Asian	2	289	Mean Difference (IV, Random, 95% CI)	‐0.24 [‐0.58, 0.10]
1.78.2 Conducted in Europe (ethnicity unspecified)	3	198	Mean Difference (IV, Random, 95% CI)	‐0.36 [‐1.18, 0.46]
1.78.3 Mixed	1	22	Mean Difference (IV, Random, 95% CI)	‐0.37 [‐0.93, 0.19]
1.79 Change in total blood cholesterol (mmol/L); subgroup BMI	6	509	Mean Difference (IV, Random, 95% CI)	‐0.31 [‐0.74, 0.12]
1.79.1 Overweight (25 to 29.9 kg/m^2^ for adult Europids, 23 to 24.9 kg/m^2^ for adult Asians)	5	487	Mean Difference (IV, Random, 95% CI)	‐0.30 [‐0.79, 0.19]
1.79.2 Obese (≥ 30 kg/m^2^ for adult Europids, ≥ 25 kg/m^2^ for adult Asians)	1	22	Mean Difference (IV, Random, 95% CI)	‐0.37 [‐0.93, 0.19]
1.80 Change in total blood cholesterol (mmol/L); subgroup blood pressure status	6	509	Mean Difference (IV, Random, 95% CI)	‐0.31 [‐0.73, 0.12]
1.80.1 Participants with hypertension	5	274	Mean Difference (IV, Random, 95% CI)	‐0.40 [‐1.14, 0.34]
1.80.2 Participants with normal blood pressure	3	235	Mean Difference (IV, Random, 95% CI)	‐0.22 [‐0.44, 0.00]
1.81 Change in total blood cholesterol (mmol/L); subgroup LSSS implementation	6	509	Mean Difference (IV, Random, 95% CI)	‐0.31 [‐0.74, 0.12]
1.81.1 Discretionary only	3	289	Mean Difference (IV, Random, 95% CI)	‐0.60 [‐1.37, 0.17]
1.81.2 Non‐discretionary only	1	90	Mean Difference (IV, Random, 95% CI)	‐0.25 [‐0.51, 0.00]
1.81.3 Discretionary and non‐discretionary	2	130	Mean Difference (IV, Random, 95% CI)	0.03 [‐0.84, 0.89]
1.82 Change in total blood cholesterol (mmol/L); subgroup type of LSSS	6	509	Mean Difference (IV, Random, 95% CI)	‐0.31 [‐0.74, 0.12]
1.82.1 ≥ 30% KCl	4	378	Mean Difference (IV, Random, 95% CI)	‐0.32 [‐1.05, 0.41]
1.82.2 < 30% KCl	2	131	Mean Difference (IV, Random, 95% CI)	‐0.29 [‐0.52, ‐0.07]
1.83 Change in total blood cholesterol (mmol/L); subgroup baseline sodium excretion (mmol/24‐h)	6	509	Mean Difference (IV, Random, 95% CI)	‐0.31 [‐0.74, 0.12]
1.83.1 High baseline 24‐h sodium excretion (≥ 172 mmol Na/24‐h, 3.95 g Na/24‐h or 9.88 g NaCl/24‐h)	2	289	Mean Difference (IV, Random, 95% CI)	‐0.24 [‐0.58, 0.10]
1.83.2 Low baseline 24‐h sodium excretion (< 172 mmol Na/24‐h, 3.95 g Na/24‐h or 9.88 g NaCl/24‐h)	3	198	Mean Difference (IV, Random, 95% CI)	‐0.36 [‐1.18, 0.46]
1.83.3 Unknown/Not 24‐h	1	22	Mean Difference (IV, Random, 95% CI)	‐0.37 [‐0.93, 0.19]
1.84 Change in total blood cholesterol (mmol/L); subgroup baseline potassium excretion (mmol/24‐h)	6	509	Mean Difference (IV, Random, 95% CI)	‐0.31 [‐0.74, 0.12]
1.84.1 High baseline 24‐h potassium excretion (≥ 59 mmol K/24‐h or 2.3 g K/24‐h)	3	198	Mean Difference (IV, Random, 95% CI)	‐0.36 [‐1.18, 0.46]
1.84.2 Low baseline 24‐h potassium excretion (< 59 mmol K/24‐h or 2.3 g K/24‐h)	2	289	Mean Difference (IV, Random, 95% CI)	‐0.24 [‐0.58, 0.10]
1.84.3 Unknown/Not 24‐h	1	22	Mean Difference (IV, Random, 95% CI)	‐0.37 [‐0.93, 0.19]
1.85 Change in 24‐h urinary sodium excretion (mmol/24‐h)	11		Mean Difference (IV, Random, 95% CI)	Totals not selected
1.86 Change in 24‐h urinary sodium excretion (mmol/24‐h) stepped‐wedge trial	1		Mean Difference (IV, Fixed, 95% CI)	0.01 [‐0.23, 0.25]
1.87 Change in 24‐h urinary potassium excretion (mmol/24‐h)	11	3885	Mean Difference (IV, Random, 95% CI)	11.44 [7.62, 15.26]
1.88 Change in 24‐h urinary potassium excretion (mmol/24‐h); subgroup study duration	11	3885	Mean Difference (IV, Random, 95% CI)	11.44 [7.62, 15.26]
1.88.1 ≤ 3 months	4	712	Mean Difference (IV, Random, 95% CI)	11.18 [4.36, 17.99]
1.88.2 > 3 to 12 months	5	488	Mean Difference (IV, Random, 95% CI)	9.47 [0.88, 18.06]
1.88.3 > 12 months	2	2685	Mean Difference (IV, Random, 95% CI)	16.03 [‐0.15, 32.21]
1.89 Change in 24‐h urinary potassium excretion (mmol/24‐h); subgroup age	11	3885	Mean Difference (IV, Random, 95% CI)	11.44 [7.62, 15.26]
1.89.1 ≥ 65 years	4	1163	Mean Difference (IV, Random, 95% CI)	15.71 [5.88, 25.54]
1.89.2 < 65 years	6	819	Mean Difference (IV, Random, 95% CI)	9.22 [2.30, 16.13]
1.89.3 Unknown	1	1903	Mean Difference (IV, Random, 95% CI)	8.00 [6.01, 9.99]
1.90 Change in 24‐h urinary potassium excretion (mmol/24‐h); subgroup gender	11	3885	Mean Difference (IV, Random, 95% CI)	11.44 [7.62, 15.26]
1.90.1 Mixed	10	1982	Mean Difference (IV, Random, 95% CI)	12.32 [7.28, 17.35]
1.90.2 Unknown	1	1903	Mean Difference (IV, Random, 95% CI)	8.00 [6.01, 9.99]
1.91 Change in 24‐h urinary potassium excretion (mmol/24‐h); subgroup ethnicity	11	3885	Mean Difference (IV, Random, 95% CI)	11.44 [7.62, 15.26]
1.91.1 Asian	5	3379	Mean Difference (IV, Random, 95% CI)	10.61 [6.37, 14.85]
1.91.2 Conducted in Europe (ethnicity unspecified)	6	506	Mean Difference (IV, Random, 95% CI)	13.48 [3.67, 23.28]
1.92 Change in 24‐h urinary potassium excretion (mmol/24‐h); subgroup BMI	11	3885	Mean Difference (IV, Random, 95% CI)	11.44 [7.62, 15.26]
1.92.1 Overweight (25 to 29.9 kg/m^2^ for adult Europids, 23 to 24.9 kg/m^2^ for adult Asians)	8	1722	Mean Difference (IV, Random, 95% CI)	12.15 [6.33, 17.97]
1.92.2 Unknown	3	2163	Mean Difference (IV, Random, 95% CI)	8.82 [6.14, 11.51]
1.93 Change in 24‐h urinary potassium excretion (mmol/24‐h); subgroup blood pressure status	11	3885	Mean Difference (IV, Random, 95% CI)	10.99 [7.51, 14.47]
1.93.1 Participants with hypertension	8	965	Mean Difference (IV, Random, 95% CI)	11.52 [6.99, 16.04]
1.93.2 Participants with normal blood pressure	3	235	Mean Difference (IV, Random, 95% CI)	4.25 [‐5.74, 14.25]
1.93.3 Unknown	1	1903	Mean Difference (IV, Random, 95% CI)	8.00 [6.01, 9.99]
1.93.4 Mixed	1	782	Mean Difference (IV, Random, 95% CI)	24.52 [18.75, 30.29]
1.94 Change in 24‐h urinary potassium excretion (mmol/24‐h); subgroup LSSS implementation	11	3885	Mean Difference (IV, Random, 95% CI)	11.44 [7.62, 15.26]
1.94.1 Discretionary only	7	3617	Mean Difference (IV, Random, 95% CI)	11.10 [7.23, 14.98]
1.94.2 Non‐discretionary only	1	90	Mean Difference (IV, Random, 95% CI)	‐3.30 [‐12.49, 5.89]
1.94.3 Discretionary and non‐discretionary	3	178	Mean Difference (IV, Random, 95% CI)	18.79 [6.89, 30.68]
1.95 Change in 24‐h urinary potassium excretion (mmol/24‐h); subgroup type of LSSS	11	3885	Mean Difference (IV, Random, 95% CI)	11.44 [7.62, 15.26]
1.95.1 ≥ 30% KCl	5	1546	Mean Difference (IV, Random, 95% CI)	13.76 [7.07, 20.45]
1.95.2 < 30% KCl	6	2339	Mean Difference (IV, Random, 95% CI)	9.42 [3.55, 15.29]
1.96 Change in 24‐h urinary potassium excretion (mmol/24‐h); subgroup baseline sodium excretion (mmol/24‐h)	11	3885	Mean Difference (IV, Random, 95% CI)	11.44 [7.62, 15.26]
1.96.1 High baseline 24‐h sodium excretion (≥ 172 mmol Na/24‐h, 3.95 g Na/24‐h or 9.88 g NaCl/24‐h)	4	1292	Mean Difference (IV, Random, 95% CI)	13.48 [5.49, 21.47]
1.96.2 Low baseline 24‐h sodium excretion(< 172 mmol Na/24‐h, 3.95 g Na/24‐h or 9.88 g NaCl/24‐h)	6	690	Mean Difference (IV, Random, 95% CI)	11.88 [2.54, 21.21]
1.96.3 Unknown/Not 24‐h	1	1903	Mean Difference (IV, Random, 95% CI)	8.00 [6.01, 9.99]
1.97 Change in 24‐h urinary potassium excretion (mmol/24‐h); subgroup baseline potassium excretion (mmol/24‐h)	11	3885	Mean Difference (IV, Random, 95% CI)	11.44 [7.62, 15.26]
1.97.1 High baseline 24‐h potassium excretion	6	506	Mean Difference (IV, Random, 95% CI)	13.48 [3.67, 23.28]
1.97.2 Low baseline 24‐h potassium excretion	4	1476	Mean Difference (IV, Random, 95% CI)	11.69 [5.11, 18.27]
1.97.3 Unknown/Not 24‐h	1	1903	Mean Difference (IV, Random, 95% CI)	8.00 [6.01, 9.99]
1.98 Change in 24‐h urinary potassium excretion (mmol/24‐h) stepped‐wedge trial	1		Mean Difference (IV, Fixed, 95% CI)	0.63 [0.48, 0.78]

**Comparison 2:** Low‐sodium salt substitutes versus regular salt or no active intervention in children

Outcome or subgroup title	No. of studies	No. of participants	Statistical method	Effect size
2.1 Change in DBP (mmHg) at > 3 to 12 months	1	92	Mean Difference (IV, Fixed, 95% CI)	1.28 [‐1.56, 4.12]
2.2 Change in SBP (mmHg) at > 3 to 12 months	1	92	Mean Difference (IV, Fixed, 95% CI)	0.12 [‐4.41, 4.64]
2.3 Change in BMI (kg/m^2^) at > 3 to 12 months	1	92	Mean Difference (IV, Fixed, 95% CI)	0.94 [0.85, 1.03]
2.4 Change in 24‐h urinary sodium (mmol/24‐h)	1	92	Mean Difference (IV, Fixed, 95% CI)	14.60 [‐11.22, 40.42]
2.5 Change in 24‐h urinary potassium (mmol/24‐h)	1	92	Mean Difference (IV, Fixed, 95% CI)	4.10 [‐5.13, 13.33]

## Characteristics of studies

### Characteristics of included studies [ordered by study ID]

**Table:** Allaert 2013

***Study characteristics***		
Methods	**Study design:** Randomised controlled trial **Study grouping:** Cross‐over **Country:** France **Setting:** Intervention conducted in households; outcomes measured at 13 centres by community medical practitioners **Aim of study:** To investigate whether added 'Chitosan' salt (3g/day), when used in conjunction with lifestyle alterations may contribute to a greater reduction in SBP and DBP, compared to standard sea salt. **Unit of allocation:** individuals **Start date:** NR **End date:** NR **Relevant study limitations as reported by study authors:** Small sample size may not result in optimal assessment of safety (only frequent side effects likely to be observed). **Sample size calculation:** Yes, "the study was powered to detect a 5 mmHg SBP difference between NaCl salt and chitosan salt at an α of 0.05 (two‐sided) and a β of 0.1."	
Participants	**Baseline Characteristics** LSSS intervention *Age*: in years, mean (SD): 59.1 (11.6) *Gender*: Female, % (n/N): 61.9 (13/21) *Ethnicity/race*: European *Smoking*: Smoker, % (n/N): 4.8 (1/21) *Body Mass Index (BMI)*: in kg per m^2^, mean (SD): 25.1 (3.8) *Blood pressure status*: Hypertensive, % (n/N): 100 (21/21) *Antihypertensive medication used: *Use of antihypertensive medication, % (n/N): 0 (0/21) *Cardiovascular disease or stroke*: High cardiovascular risk, % (n/N): 0 (0/21) *Diabetes mellitus*: NR *Renal impairment*: NR *Dietary potassium intake*: NR *Dietary sodium intake*: NR *Urinary potassium excretion*: mean (SD): 60.4 (28.8) mmol/L *Urinary sodium excretion*: mean (SD): 117.9 (48.2) mmol/L Control *Age*: in years, mean (SD): 58.0 (12.7) *Gender*: Female, % (n/N): 57.9 (11/19) *Ethnicity/race*: European *Smoking*: Smoker, % (n/N): 21.1 (4/19) *Body Mass Index (BMI)*: in kg per m^2^, mean (SD): 27.7 (5.8) *Blood pressure status*: Hypertensive, % (n/N): 100 (19/19) *Antihypertensive medication used*: Use of antihypertensive medication, % (n/N): 0 (0/19) *Cardiovascular disease or stroke*: High cardiovascular risk, % (n/N): 0 (0/19) *Diabetes mellitus*: NR *Renal impairment*: NR *Dietary potassium intake*: NR *Dietary sodium intake*: NR *Urinary potassium excretion*: mean (SD): 63.1(25.2) mmol/L *Urinary sodium excretion*: mean (SD): 155.3 (71.0) mmol/L **Inclusion criteria:** "French‐speaking outpatients of either sex, aged 18 to 85 years, with stage 1 hypertension (SBP 140‐159 mmHg or DBP 90‐99 mmHg) who had never previously received antihypertensive treatment." **Exclusion criteria:** Participants with allergic reactions to seafood, serious illnesses, or high‐risk cardiovascular profiles were excluded. Pregnant and lactating women, as well as those who were not under effective contraception or had been in menopause for less than two years, were excluded. **Pretreatment:** none **Method of recruitment of participants:** Community medical practitioners recruited outpatients **Informed consent obtained:** yes (written) **Clusters:** n/a **Subgroups planned/measured:** NR **Subgroups reported:** none *Participant flow* **Assessed for eligibility:** NR **Excluded (number with reasons):** NR **Randomised:** n = 40 **Allocated to LSSS intervention/s:** n = 21 (first time period) **Allocated to control:** n = 19 (first time period) **Received allocated LSSS intervention/s:** NR **Did not receive allocated LSSS intervention/s:** NR **Lost to follow‐up (LSSS intervention group):** n = 0 **Discontinued intervention (LSSS intervention group):** n = 1 (second time period) **Analysed (LSSS intervention group):** n = 21 (first time period); n = 20 (second time period) **Excluded from analysis (LSSS intervention group):** n = 0 (first time period); n = 0 (second time period) **Received allocated control:** NR **Did not receive allocated control:** NR **Lost to follow‐up (control group):** n = 0 **Discontinued intervention (control group):** n = 1 (first time period); study authors describe this as "patients's own decision" **Analysed (control group):** n = 18 (first time period); n = 17 (second time period) **Excluded from analysis (control group):** n = 1 (second time period) ‐ lack of washout period	
Interventions	**Intervention Characteristics** LSSS intervention *Theoretical basis*: Chitosan (polysaccharide derived from chitin found in shellfish) may reduce SBP by inhibiting the angiotensin converting enzyme (ACE). *Description*: Chitosan salt or Symbiosal (NaCl combined with 3% chitosan). Restrict added salt to a maximum of 3 g a day. *LSSS category*: no KCl *Contains fortificant*: NR *Delivery*: discretionary use *Duration of run‐in period*: 2 weeks *Duration of active intervention*: 8 weeks *Duration of follow‐up (as reported)*: none *Total duration of study (as reported)*: 18 weeks *Timing*: NR *Implementation*: Salt cellar with a measuring spoon (0.5 g) *Providers*: Gun‐Sik CHO (Biotech Co Ltd) *Co‐interventions*: Lifestyle advice was given about eating less fat and sugar, and doing more physical exercise. Participants to avoid liquorice. *Resource requirements*: NR *Integrity of delivery*: Added salt intake in g/day (first time period), mean (SD): 2.9 (1.6) Control *Theoretical basis*: To enable comparison of LSSS intervention with standard practice *Description*: NaCl salt or sea salt. Restrict added salt to a maximum of 3 g a day. *Contains fortificant*: NR *Delivery*: discretionary use *Duration of run‐in period*: 2 weeks *Duration of active intervention*: 8 weeks *Duration of follow‐up (as reported)*: none *Total duration of study (as reported)*: 18 weeks *Timing*: NR *Implementation*: Salt cellar with a measuring spoon (0.5 g) *Providers*: Gun‐Sik CHO (Biotech Co Ltd) *Co‐interventions*: Lifestyle advice was given about eating less fat and sugar, and doing more physical exercise. Participants to avoid liquorice. *Resource requirements*: NR *Integrity of delivery*: Added salt intake in g/day (first time period), mean (SD): 3 (1.5)	
Outcomes	Primary outcomes **Diastolic Blood Pressure (DBP):** Outcome measurement: Measurements performed using an automated digital sphygmomanometer after 10 mins rest, with patient in seating position, with three measurements that were 3 mins apart, average of 3 measurements calculated; time points: 8 weeks (first time period) **Systolic Blood Pressure (SBP):** Outcome measurement: Measurements performed using an automated digital sphygmomanometer after 10 mins rest, with patient in seating position, with three measurements that were 3 mins apart, average of 3 measurements calculated; time points: 8 weeks (first time period) **Hypertension (as reported, or SBP > 140 mmHg or DBP > 85 mmHg):** NR **Blood pressure control (achieving blood pressure threshold or 'control' as prespecified):** Outcome measured: Achieving SBP <= 140 mmHg, or DBP <= 90 mmHg, or both. Time points: 8 weeks (first time period) **Cardiovascular events‐stroke:** NR **Cardiovascular events‐myocardial infarction:** NR **Cardiovascular events‐other:** NR (first time period) **Cardiovascular mortality:** NR **Blood potassium:** NR (first time period) **Hyperkalaemia:** NR **Hypokalaemia:** NR Secondary outcomes **All‐cause mortality:** NR **Adverse events:** NR (first time period) **Antihypertensive medication use:** NR **Body Mass index (BMI):** NR **Serum creatinine:** NR **Albuminuria:** NR **Urinary albumin‐to‐creatinine ratio (uACR):** NR **Fasting blood glucose:** NR **Blood triglycerides:** NR **Total blood cholesterol:** NR **24‐h urinary sodium excretion:** NR **24‐h urinary potassium excretion:** NR	
Notes	**Funding source:** NR **Authors name:** Francois‐Andre Allaert **Institution:** University of Burgundy **Email:** allaert@cenbiotech.com **Possible conflicts of interest (for study authors):** NR **Sources used for data extraction:** Journal article(s) with results of the trial **Trial registration details:** NR	
***Risk of bias***		
**Bias**	**Authors' judgement**	**Support for judgement**
Random sequence generation (selection bias)	Unclear risk	Study stated it was randomised but method of randomisation not reported
Allocation concealment (selection bias)	Unclear risk	Insufficient information available
Blinding of participants and personnel (performance bias) All outcomes	Unclear risk	Blinding of participants and personnel was not described.
Blinding of outcome assessment (detection bias) All outcomes	Low risk	Although it was unclear whether the medical practitioners were blinded, the SBP and DBP measurements not likely to be influenced by the lack of blinding (use of automatic BP device)
Incomplete outcome data (attrition bias) All outcomes	Low risk	Study used ITT analysis. Missing outcome data were reported in the control group during the first time period 5.3% (1/19). The details of the ITT analysis were not described.
Selective reporting (reporting bias)	Unclear risk	No study protocol or prospective trial registry entry available
Other bias	Low risk	None identified
Overall risk of bias	Unclear risk	Unclear risk of bias for allocation concealment; low risk of bias for incomplete outcome data

**Table:** Allaert 2017

***Study characteristics***		
Methods	**Study design:** Randomised controlled trial **Study grouping:** Parallel group **Country:** France **Setting:** Intervention conducted at household level; outcomes assessed at a clinical investigation centre **Aim of study:** To evaluate whether the use of LSSS (Symbiosal) reduces prehypertension more than standard table marine salt (NaCl) **Unit of allocation:** adults (aged 18 years or older) **Start date:** NR **End date:** NR **Relevant study limitations as reported by study authors:** NR **Sample size calculation:** Yes, using an estimated reduction of 10 mmHg (SD 10) in SBP in the LSSS group, compared to the control group, at a power of 80%	
Participants	**Baseline Characteristics** LSSS intervention *Age*: NR *Gender*: NR *Ethnicity/race*: European *Smoking*: NR *Body Mass Index (BMI)*: NR *Blood pressure status*: Pre‐hypertensive, % (n/N): 100 (22/22) *Antihypertensive medication used*: Current and previous use (up to 12 months prior), % (n/N): 0 (0/22) *Cardiovascular disease or stroke*: Heart failure, coronary artery disease, history of stroke or transient ischaemic attack, peripheral arterial disease, % (n/N): 0 (0/22) *Diabetes mellitus*: % (n/N): 0 (0/22) *Renal impairment*: Kidney failure or kidney disease, % (n/N): 0 (0/22) *Dietary potassium intake*: NR *Dietary sodium intake*: NR *Urinary potassium excretion*: in mmol/L, mean (SD): 77.4 (35.5) *Urinary sodium excretion*: in mmol, mean (SD): 115.0 (51.6) Control *Age*: NR *Gender*: NR *Ethnicity/race*: European *Smoking*: NR *Body Mass Index (BMI)*: NR *Blood pressure status*: Pre‐hypertensive, % (n/N): 100 (19/19) *Antihypertensive medication used: *Current* *and previous use (up to 12 months prior), % (n/N): 0 (0/19) *Cardiovascular disease or stroke*: Heart failure, coronary artery disease, history of stroke or transient ischaemic attack, peripheral arterial disease, % (n/N): 0 (0/19) *Diabetes mellitus*: % (n/N): 0 (0/19) *Renal impairment*: Kidney failure or kidney disease, % (n/N): 0 (0/19) *Dietary potassium intake*: NR *Dietary sodium intake*: NR *Urinary potassium excretion*: in mmol/L, mean (SD): 80.6 (32.0) *Urinary sodium excretion*: in mmol/L, mean (SD): 146.8 (51.9) Overall *Age*: in years, mean (SD); 51 (16) *Gender*: Male, % (n/N): 51.2 (21/41) *Ethnicity/race*: NR *Smoking*: Never smoked, % (n/N); 57.5% (24/41); previous smokers, % (n/N): 27.5% (11/41); current smokers, % (n/N): 15% (6/41) *Body Mass Index (BMI)*: Overweight, % (n/N): 36.6 (15/41); obese, % (n/N): 29.3 (12/41) *Blood pressure status*: Pre‐hypertensive, % (n/N): 100 (41/41) *Antihypertensive medication used*: Current* *and previous use (up to 12 months prior), % (n/N): 0 (0/41) *Cardiovascular disease or stroke*: Heart failure, coronary artery disease, history of stroke or transient ischemic attack, peripheral arterial disease, % (n/N): 0 (0/41) *Diabetes mellitus*: % (n/N): 0 (0/41) *Renal impairment*: Kidney failure or kidney disease, % (n/N): 0 (0/41) *Dietary potassium intake*: NR *Dietary sodium intake*: NR *Urinary potassium excretion*: NR *Urinary sodium excretion*: NR **Inclusion criteria:** Men and women > 18 and < 70 years with systolic pre‐hypertension (SBP > 130 and < 139 mmHg, with or without DBP between 80 and 89 mmHg) on 2 successive visits 7 days apart and on the average daily measurements of participants taken during a 7‐day run‐in period **Exclusion criteria:** Participants taking antihypertensive medication during the past 12 months, allergic reaction to seafood, or had a high risk cardiovascular profile with at least one of the following comorbidities: heart failure, coronary artery disease, history of stroke or transient ischaemic attack, peripheral arterial disease, kidney failure or kidney disease and diabetes **Pretreatment:** NR **Method of recruitment of participants:** NR **Informed consent obtained:** Yes (written) **Clusters:** n/a **Subgroups planned/measured:** NR **Subgroups reported:** NR *Participant flow* **Assessed for eligibility:** n = 58 **Excluded:** n = 17 excluded as they did not have a mean SBP of > 130 but < 140mmHg during the run‐in period **Randomised:** n = 41 **Allocated to LSSS intervention/s:** n = 22 **Allocated to control:** n = 19 **Received allocated LSSS intervention/s:** NR **Did not receive allocated LSSS intervention/s:** NR **Lost to follow‐up (LSSS intervention group):** n = 0 (SBP; DBP); n = 1 (blood potassium): no reason provided **Discontinued intervention (LSSS intervention group):** n = 0 **Analysed (LSSS intervention group):** n = 22 (SBP; DBP); n = 21 (blood potassium) **Excluded from analysis (LSSS intervention group):** n = 0 **Received allocated control (number):** NR **Did not receive allocated control:** NR **Lost to follow‐up (control group):** n = 0 (SBP; DBP); n = 1 (blood potassium): no reason provided **Discontinued intervention (control group):** n = 0 **Analysed (control group):** n = 19 (SBP; DBP); n = 18 (blood potassium) **Excluded from analysis (control group):** n = 0	
Interventions	**Intervention Characteristics** LSSS intervention *Theoretical basis*: Chitosan is a polysaccharide, derived from chitin (component of shellfish). It chelates chloride (Cl), thereby inhibiting the stimulation of both renin and angiotensin I converting enzyme (ACE), resulting in an antihypertensive effect. *Description*: Symbiosal (marine NaCl plus 3% added chitosan). Participants not to use more than 3 g per day. *LSSS category*: no KCl *Contains fortificant*: NR *Delivery*: discretionary use *Duration of run‐in period*: 1 week *Duration of active intervention*: 8 weeks *Duration of follow‐up (as reported)*: none *Total duration of study (as reported)*: 8 weeks *Timing*: NR *Implementation*: One 300 g salt cellar for the study period, with a measuring spoon (0.5 g) *Providers*: NR *Co‐interventions*: None. Participants were not to change their dietary, physical activity, smoking habits during the study period. *Resource requirements*: NR *Integrity of delivery*: Participants were required to return the salt cellar at the 8‐week follow‐up visit. A FFQ was completed by each participant at baseline and at follow‐up in order to assess any changes in their dietary intake during the study period. LSSS consumption: 42.5 (21.8) % of the provided salt cellar, corresponding to 2.2 (1.1) g LSSS/day Control *Theoretical basis*: To enable comparison of LSSS intervention with standard practice *Description*: Standard NaCl. Participants not to consume more than 3 g salt daily. *Contains fortificant*: NR *Delivery*: discretionary use *Duration of run‐in period*: 1 week *Duration of active intervention*: 8 weeks *Duration of follow‐up (as reported)*: none *Total duration of study (as reported)*: 8 weeks *Timing*: NR *Implementation*: One 300 g salt cellar for the study period, with a measuring spoon (0.5 g) *Providers*: NR *Co‐interventions*: None. Participants were not to change their dietary, physical activity, smoking habits during the study period. *Resource requirements*: NR *Integrity of delivery*: Participants were required to return the salt cellar at the 8‐week follow‐up visit. A FFQ was completed by each participant at baseline and at follow‐up in order to assess any changes in their dietary intake during the study period. Regular salt consumption: 47.0 (21.8) % of the provided salt cellar, corresponding to 2.5 (1.2) g/day. Increased proportion of participants reporting the consumption of bread, rusks or breakfast cereals (8/19 vs. 1/22; P = 0.0128)	
Outcomes	Primary outcomes **Diastolic Blood Pressure (DBP):** Outcome measurement: Three measurements conducted with 3 mins intervals in between, in the lying position after 10 mins rest with a validated BP monitor by the investigator. Home‐based BP monitoring: participants instructed to measure their BP at baseline (‐7 days to day 0) and end of study period (days 50 to days 56) with a validated BP monitor at 7 pm according to the same protocol. Time points: 8 weeks **Systolic Blood Pressure (SBP):** Outcome measurement: Three measurements conducted with 3 mins intervals in between, in the lying position after 10 mins rest with a validated BP monitor by the investigator. Home‐based BP monitoring: Participants instructed to measure their BP at baseline (‐7 days to day 0) and end of study period (days 50 to days 56) with a validated BP monitor at 7 pm according to the same protocol. Time points: 8 weeks **Hypertension (as reported, or SBP > 140 mmHg or DBP > 85 mmHg):** NR **Blood pressure control (achieving blood pressure threshold or 'control' as prespecified):** NR **Cardiovascular events‐stroke:** NR **Cardiovascular events‐myocardial infarction:** NR **Cardiovascular events‐other:** Angina; outcome measurement: NR; time point: during the study period **Cardiovascular mortality:** NR **Blood potassium:** Outcome measurement: NR; time points: 8 weeks **Hyperkalaemia:** NR **Hypokalaemia:** NR Secondary outcomes **All‐cause mortality:** NR **Adverse events:** Influenza; dorsalgia; outcome measurement: NR; time points: during the study period **Antihypertensive medication use:** NR **Body Mass index (BMI):** NR **Serum creatinine:** NR **Albuminuria:** NR **Urinary albumin‐to‐creatinine ratio (uACR):** NR **Fasting blood glucose:** NR **Blood triglycerides:** NR **Total blood cholesterol:** NR **24‐h urinary sodium excretion:** NR **24‐h urinary potassium excretion:** NR	
Notes	**Funding source:** NR **Authors name:** Francois‐Andre Allaert **Institution:** NR **Email:** allaert@cenbiotech.com **Possible conflicts of interest (for study authors):** NR **Sources used for data extraction:** Journal article with results of the trial **Trial registration details:** NR	
***Risk of bias***		
**Bias**	**Authors' judgement**	**Support for judgement**
Random sequence generation (selection bias)	Unclear risk	Insufficient information available
Allocation concealment (selection bias)	Low risk	Study products were pre‐packaged and numbered according to the randomised code and delivered to the clinical investigation centre.
Blinding of participants and personnel (performance bias) All outcomes	Low risk	LSSS and regular salt were visually comparable and pre‐packaged (not at the investigation centre), therefore both participants and study personnel at the centre were blinded.
Blinding of outcome assessment (detection bias) All outcomes	Low risk	SBP, DBP measurements (use of automatic BP device) not likely to be influenced by the lack of blinding
Incomplete outcome data (attrition bias) All outcomes	Low risk	SBP and DBP outcomes: no incomplete outcome data for investigator measurements; 9% (2/22) missing data in intervention group and no incomplete data in control group for home BP measurements. Blood potassium reported for 21 out of 22 participants in the intervention group (95.5%), and 18 out of 19 participants (94.7%), respectively. Reasons for missing data not provided
Selective reporting (reporting bias)	Unclear risk	No protocol or prospective trial registry entry available
Other bias	Low risk	None identified
Overall risk of bias	Low risk	Low risk of bias for allocation concealment and incomplete outcome data

**Table:** Arzilli 1986

***Study characteristics***		
Methods	**Study design:** Randomised controlled trial **Study grouping:** Parallel group **Country:** Italy **Setting:** Intervention conducted in a hospital; outcomes measured in hospital **A. Aim of study:** To investigate whether a salt high in potassium and low in sodium can reduce blood pressure **B. Unit of allocation:** adults (aged 18 years or older) **C. Start date:** NR **D. End date:** NR **E. Relevant study limitations as reported by study authors:** NR **F. Sample size calculation:** NR	
Participants	**Baseline Characteristics** Overall *Age*: in years, range: 28 to 53 *Gender*: Female, % (n/N): 40 (4/10) *Ethnicity/race*: European *Smoking*: NR *Body Mass Index (BMI)*: NR *Blood pressure status*: Hypertensive, % (n/N): 100 (10/10) *Antihypertensive medication used*: NR *Cardiovascular disease or stroke*: NR *Diabetes mellitus*: NR *Renal impairment*: NR *Dietary potassium intake*: NR *Dietary sodium intake*: NR *Urinary potassium excretion*: NR *Urinary sodium excretion*: NR **Inclusion criteria:** Hospital inpatients with mild hypertension, defined as supine diastolic blood pressure of 95 mmHg after five days in hospital, eating a standard diet containing approximately 20 mmol sodium plus 4 g common salt **Exclusion criteria:** NR **Pretreatment:** NR **Method of recruitment of participants:** NR **Informed consent obtained:** NR **Clusters:** n/a **Subgroups planned/measured:** NR **Subgroups reported:** NR *Participant flow* **Assessed for eligibility:** NR **Excluded (number with reasons):** NR **Randomised:** n = 10 **Allocated to LSSS intervention/s:** n = 5 **Allocated to control:** n = 5 **Received allocated LSSS intervention/s:** NR **Did not receive allocated LSSS intervention/s:** NR **Lost to follow‐up (LSSS intervention group):** NR **Discontinued intervention (LSSS intervention group):** NR **Analysed (LSSS intervention group):** n = 5 **Excluded from analysis (LSSS intervention group):** n = 0 **Received allocated control:** NR **Did not receive allocated control:** NR **Lost to follow‐up (control group):** NR **Discontinued intervention (control group):** NR **Analysed (control group):** n = 5 **Excluded from analysis (control group):** n = 0	
Interventions	**Intervention Characteristics** LSSS intervention *Theoretical basis*: A potassium‐rich, sodium‐poor salt may reduce blood pressure. *Description*: K‐rich/Na‐poor salt (4 g per day) *LSSS category*: unknown *Contains fortificant*: NR *Delivery*: discretionary use *Duration of run‐in period*: 5 days *Duration of active intervention*: 8 days *Duration of follow‐up (as reported)*: none *Total duration of study (as reported)*: 8 days *Timing*: Participants received 2 g twice daily *Implementation*: Combined with a standard hospital diet containing 20 mmol Na *Providers*: Details of manufacturer and persons providing the LSSS not reported *Co‐interventions*: NR *Resource requirements*: NR *Integrity of delivery*: NR Control *Theoretical basis*: To enable comparison of LSSS intervention with standard practice *Description*: 'Common' salt (4 g per day) *Contains fortificant*: NR *Delivery*: discretionary use *Duration of run‐in period*: 5 days *Duration of active intervention*: 8 days *Duration of follow‐up (as reported)*: none *Total duration of study (as reported)*: 8 days *Timing*: Participants received 2 g twice daily *Implementation*: Combined with a standard hospital diet containing 20 mmol Na *Providers*: Details of manufacturer and persons providing the common salt not reported *Co‐interventions*: NR *Resource requirements*: NR *Integrity of delivery*: NR	
Outcomes	Primary outcomes: **Diastolic Blood Pressure (DBP):** Outcome measurement: NR. Time points: 4 and 8 days **Systolic Blood Pressure (SBP):** Outcome measurement: NR. Time points: 4 and 8 days **Hypertension (as reported, or SBP > 140 mmHg or DBP > 85 mmHg):** NR **Blood pressure control (achieving blood pressure threshold or 'control' as prespecified):** NR **Cardiovascular events‐stroke:** NR **Cardiovascular events‐myocardial infarction:** NR **Cardiovascular events‐other:** NR **Cardiovascular mortality:** NR **Blood potassium:** NR **Hyperkalaemia:** NR **Hypokalaemia:** NR Secondary outcomes: **All‐cause mortality:** NR **Adverse events:** NR **Antihypertensive medication use:** NR **Body Mass index (BMI):** NR **Serum creatinine:** NR **Albuminuria:** NR **Urinary albumin‐to‐creatinine ratio (uACR):** NR **Fasting blood glucose:** NR **Blood triglycerides:** NR **Total blood cholesterol:** NR **24‐h urinary sodium excretion:** NR **24‐h urinary potassium excretion:** NR	
Notes	**Funding source:** NR **Authors name:** Antonio Salvetti **Institution:** Department of Internal Medicine, University of Pisa **Email:** a.salvetti@med.unipi.it **Possible conflicts of interest (for study authors):** NR **Sources used for data extraction:** Conference abstract about the trial **Trial registration details:** NR **Author correspondence:** We contacted Dr. Salvetti 07/07/2020 in an attempt to obtain the full text. Received no reply	
***Risk of bias***		
**Bias**	**Authors' judgement**	**Support for judgement**
Random sequence generation (selection bias)	Unclear risk	Insufficient information available
Allocation concealment (selection bias)	Unclear risk	Insufficient information available
Blinding of participants and personnel (performance bias) All outcomes	Unclear risk	Study authors reported that the intervention and control salts were administered in 'double‐blind' conditions. Insufficient information available to assess what this meant
Blinding of outcome assessment (detection bias) All outcomes	Low risk	Outcomes were unlikely to be affected by a lack of blinding.
Incomplete outcome data (attrition bias) All outcomes	Unclear risk	Attrition was not reported in sufficient detail to determine whether any participants were lost.
Selective reporting (reporting bias)	Unclear risk	Study protocol or prospective trial registry entry not available
Other bias	Low risk	None identified
Overall risk of bias	Unclear risk	Unclear risk of bias for allocation concealment and incomplete outcome data

**Table:** Bernabe‐Ortiz 2014

***Study characteristics***		
Methods	**Study design:** Cluster‐randomised trial **Study grouping: **Stepped‐wedge **Country:** Peru **Setting:** Intervention conducted in semi‐urban villages in Tumbes, northern Peru; outcome measurement conducted at each household **Aim of study:** To assess the efficacy of a pragmatic salt‐substitution strategy on blood pressure and the incidence of hypertension at population level **Unit of allocation:** Villages **Start date:** April 2014 **End date:** March 2017 **Relevant study limitations as reported by study authors:** The authors reported absence of dietary assessment of other sources of sodium and potassium, excluding persons with kidney disease and those who were on digoxin, and potential under‐collection of 24‐hour urine at baseline due to lower levels of creatinine at baseline (when compared to follow‐up) as limitations **Sample size calculation:** Based on an expected difference of 3 mmHg BP between groups (assuming a SD of 20 mmHg of BP within clusters (villages), no. of time periods as 6, a mean cluster size of 300 participants, and an approximation for the ICC of 0.2) using a power of 90% and significance level of 5%	
Participants	**Baseline Characteristics** Overall *Age*: in years, mean (SD): 43.3 (17.2) [village A 44.5 (16.8); village B 44.8 (18.8); village C 42.6 (16.8); village D 38.9 (14.5); village E 48.8 (18.4); village F 40.0 (15.9)] *Gender*: Female, % (n/N): 50.4 (1197/2376) [village A 45.7 (245/534); village B 49.4 (221/449); village C 50.8 (167/329); village D 54.1 (224/414); village E 52.1 (171/328); village F 52.5 (169/322)] *Ethnicity/race*: South American *Smoking*: NR *Body Mass Index (BMI)*: in kg/m^2^, mean (SD): 27.2 (4.6) [village A 27.4 (4.9); village B 26.4 (4.3); village C 26.8 (4.5); village D 27.5 (4.4); village E 27.2 (4.9); village F 27.8 (4.6)] *Blood pressure status*: Hypertensive, % (n/N): 18.3 (428/2342) [village A 17.1 (91/534); village B 20.5 (90/449); village C 18.2 (59/329); village D 13.6 (56/414); village E 24.8 (79/328); village F 16.9 (53/322)] *Antihypertensive medication used*: NR *Cardiovascular disease or stroke*: NR *Diabetes mellitus*: Type 2 diabetes, % (n/N): village A 4.3 (23/534); village B 2.2 (10/449); village C 3.3 (11/329); village D 3.1 (13/414); village E 4.6 (15/328); village F 4.7 (15/322) *Renal impairment*: NR *Dietary potassium intake*: NR *Dietary sodium intake*: NR *Urinary potassium excretion*: Subsample (n = 602), in mmol/24 h, mean (SD): 51 (31) *Urinary sodium excretion*: Subsample (n = 602), in mmol/24 h, mean (SD): 171 (81) **Inclusion criteria:** Villages: mid‐sized villages (350 to 700 people living in 130 to 250 households) in the semi‐urban area of the Tumbes region. Individuals: men and women who are full‐time residents of one of the six randomly selected villages; aged 18 years or older; capable of understanding procedures and providing informed consent **Exclusion criteria:** Households: any with a family member meeting individual exclusion criteria. Individuals: persons with a history of terminal or severe chronic kidney disease (receiving any form of dialysis); taking digoxin or potassium‐sparing diuretics for heart conditions. Participants with mental illness that impaired their ability of providing consent, were excluded. **Pretreatment:** The study authors reported that there were baseline differences between the clusters (villages) in terms of age, education, wealth index, BMI, SBP, DBP and the proportion of participants with hypertension. **Method of recruitment of participants:** Potentially eligible individuals from six villages were identified from the latest census in the area at the time (2010). Engagement with authorities and village leaders was initiated first, through a presentation and explanation of the study at village level. Once the research activities were explained, potential participants were contacted through home visits with the intention of enrolling all members of the family in eligible households. **Informed consent obtained:** Yes, unclear whether written or oral **Clusters:** Six clusters (villages) were included in the stepped‐wedge design; comprising n = 536 participants in village A, n = 447 in village B, n = 329 in village C, n = 414 in village D, n = 328 in village E, and n = 322 in village F. The clustering of villages were taken into account in the primary analysis, with a random intercept for cluster used in the modelling of SBP and DBP (standard errors allowed for intra‐class correlation of SBP and DBP). **Subgroups planned/measured:** The study authors stated the following: "The analysis will compare the information obtained from different subgroups of participants in order to identify and describe the similarities and divergences between men and women, patients from different age groups, and health workers and stakeholders". **Subgroups reported:** normotensive vs. hypertensive; age < 40 years vs. 40 to 59 years vs. >= 60 years *Participant flow* **Assessed for eligibility:** n = 100 clusters (villages); n = 2605 participants from n = 6 randomly selected clusters (villages) **Excluded (number with reasons):** n = 94 clusters (n = 80 wrong village size; n = 14 randomly excluded); n = 229 participants from n = 6 clusters (reasons NR) **Randomised:** n = 6 clusters; n = 2376 participants **Allocated to LSSS intervention(s):** n = 6 clusters; 3605.3 person‐years (n = 536 for 1366.1 person‐years in village A, n = 447 for 883.1 person‐years in village B, n = 329 for 518.3 person‐years in village C, n = 414 for 460.2 person‐years in village D, n = 328 for 256.3 person‐years in village E, and n = 322 for 121.3 person‐years in village F) **Allocated to control:** n = 6 clusters; 2547.3 person‐years (n = 536 for 1.7 person‐years in village A, n = 447 for 286.9 person‐years in village B, n = 329 for 329.0 person‐years in village C, n = 414 for 542.1 person‐years in village D, n = 328 for 637.0 person‐years in village E, and n = 322 for 750.6 person‐years in village F) **Received allocated LSSS intervention(s):** NR **Did not receive allocated LSSS intervention(s):** NR **Lost to follow‐up (LSSS intervention group):** Cumulative total: n = 0/6 clusters; n = 950/8987 (10.6%) participants. Step 1: n = 0 clusters; n = 54 in village A. Step 2: n = 76 (n = 0 clusters; n = 46 in village A, and n = 30 in village B). Step 3: n = 0 clusters; n = 115 (n = 56 in village A, n = 51 in village B, and n = 8 in village C). Step 4: n = 0 clusters; n = 116 (n = 44 in village A, n = 34 in village B, n = 27 in village C, and n = 61 in village D). Step 5: n = 0 clusters; n = 224 (n = 57 in village A, n = 43 in village B, n = 27 in village C, n = 68 in village D, and n = 29 in village E). Step 6: n = 0 clusters; n = 315 (n = 77 in village A, n = 62 in village B, n = 29 in village C, n = 84 in village D, n = 28 in village E, and n = 35 in village F). **Discontinued intervention (LSSS intervention group):** NR **Analysed (LSSS intervention group):** Step 1: n = 1 clusters; n = 482 in village A. Step 2: n = 2 clusters; n = 907 (n = 490 in village A, and n = 417 in village B). Step 3: n = 3 clusters; n = 1177 (n = 480 in village A, n = 396 in village B, and n = 301 in village C). Step 4: n = 4 clusters; n = 1560 (n = 492 in village A, n = 413 in village B, n = 302 in village C, and n = 353 in village D). Step 5: n = 5 clusters; n = 1830 (n = 479 in village A, n = 404 in village B, n = 302 in village C, n = 346 in village D, and n = 299 in village E). Step 6: n = 6 clusters; n = 2011 (n = 459 in village A, n = 385 in village B, n = 300 in village C, n = 330 in village D, n = 300 in village E, and n = 237 in village F) **Excluded from analysis (LSSS intervention group):** NR **Received allocated control:** NR **Did not receive allocated control:** NR **Lost to follow‐up (control group):** Cumulative total: n = 0/6 clusters; n = 536/5269 (10.2%) participants. Step 1: n = 0 clusters; n = 183 from five villages (B to F). Step 2: n = 0 clusters; n = 146 from four villages (C to F). Step 3: n = 0 clusters; n = 125 from three villages (D to F). Step 4: n = 0 clusters; n = 52 from two villages (E and F). Step 5: n = 0 clusters; n = 30 from one village (F) **Discontinued intervention (control group):** NR **Analysed (control group):** Step 1: n = 5 clusters; n = 1657 from five villages (B to F). Step 2: n = 4 clusters; n = 1247 from four villages (C to F). Step 3: n = 3 clusters; n = 939 from three villages (D to F). Step 4: n = 2 clusters; n = 598 from two villages (E and F). Step 5: n = 1 clusters; n = 292 from one village (F) **Excluded from analysis (control group):** NR	
Interventions	**Intervention Characteristics** LSSS intervention *Theoretical basis*: Population level LSSS intervention may reduce BP and the incidence of hypertension. *Description*: Salt substitute (75% NaCl; 25% KCl) *LSSS category*: < 30% KCl *Contains fortificant*: NR *Delivery*: discretionary use *Duration of run‐in period*: none *Duration of active intervention*: ranged from 5 to 30 months (median duration of 15 months); 3605.3 person‐years (median 489.25 person‐years) *Duration of follow‐up (as reported)*: none *Total duration of study (as reported)*: 30 months *Timing*: Daily use throughout the intervention period *Implementation*: Replacement of 'ordinary' salt with LSSS via the salt supply chain at the population level: in households, food vendors, bakeries, community kitchens and restaurants in each village. Social marketing strategy in each village aimed at women responsible for food preparation at home. Additional salt substitute packs were also made freely available during the study period in case any household required additional salt. *Providers*: Manufacturer of LSSS and details of persons providing the LSSS not reported *Co‐interventions*: NR *Resource requirements*: Cost of LSSS was 14 Peruvian Sol (PEN; ~4 USD) at scaled price reduction for study (usually 35 PEN; ~10 USD). LSSS provided free of charge to participants. Other costs specified but not reported were costs associated with marketing campaign, training, administration, production, and delivery. *Integrity of delivery*: The assessments of salt consumption were carried out using questionnaires and weighing of salt containers at randomly selected households over time, and also by evaluating supply chain management indicators such as rate of delivery of the salt substitute to each family or to food vendors. There was a delay in salt substitute delivery of, on average, 15 d. Control *Theoretical basis*: To enable comparison of LSSS intervention with standard practice *Description*: 'ordinary' salt (100% NaCl) *Contains fortificant*: NR *Delivery*: discretionary use *Duration of run‐in period*: none *Duration of active intervention*: ranged from 5 to 25 months (median duration of 12.5 months); 2547.3 (median 435.55) person‐years *Duration of follow‐up (as reported)*: none *Total duration of study (as reported)*: 30 months *Timing*: Daily use throughout the control period *Implementation*: 'Ordinary' salt used in households, food vendors, bakeries, community kitchens and restaurants in each village. *Providers*: Normal salt supply chain *Co‐interventions*: NR *Resource requirements*: Cost of 1 kg of common salt: from $US0.15 to $US0.17 (about 0.50 PEN) *Integrity of delivery*: n/a	
Outcomes	Primary outcomes: **Diastolic Blood Pressure (DBP):** Outcome measurement: performed with the participants seated, after a 5‐min resting period, using an automated device, validated in adults. Three different measurements, at least 1 min apart, were carried out, and the average of the second and third measurements was used for the analyses; time points: time and cluster regression for all time points **Systolic Blood Pressure (SBP):** Outcome measurement: performed with the participants seated, after a 5‐min resting period, using an automated device, validated in adults. Three different measurements, at least 1 min apart, were carried out, and the average of the second and third measurements was used for the analyses; time points: time and cluster regression for all time points **Hypertension (as reported, or SBP > 140 mmHg or DBP > 85 mmHg):** Outcome measurement: at baseline defined as SBP >= 140 mm Hg, DBP >= 90 mm Hg for the average of the second and third measurements, or self‐reported physician diagnosis, or current antihypertensive treatment. At follow‐up, visit defined as SBP >= 140 mmHg, DBP >= 90 mmHg or using repeated assessments every five months of these cut‐offs; time points: duration of study period **Blood pressure control (achieving blood pressure threshold or 'control' as prespecified):** NR **Cardiovascular events‐stroke:** NR **Cardiovascular events‐myocardial infarction:** NR **Cardiovascular events‐other:** NR **Cardiovascular mortality:** NR **Blood potassium:** NR **Hyperkalaemia:** NR **Hypokalaemia:** NR Secondary outcomes: **All‐cause mortality:** NR **Adverse events:** NR **Antihypertensive medication use:** Outcome measurement: participant‐reported medication use obtained through questionnaire. Time points: duration of the study **Body Mass index (BMI):** NR **Serum creatinine:** NR **Albuminuria:** NR **Urinary albumin‐to‐creatinine ratio (uACR):** NR **Fasting blood glucose:** NR **Blood triglycerides:** NR **Total blood cholesterol:** NR **24‐h urinary sodium excretion:** 24‐hour urine sample. Time points: 30 months **24‐h urinary potassium excretion:** 24‐hour urine sample. Time points: 30 months	
Notes	**Funding source:** The study, its authors and the CRONICAS Center of Excellence in Chronic Diseases were supported by the National Heart, Lung, and Blood Institute: the study under the Global Alliance for Chronic Diseases (GACD) programme; the authors and their affiliate centre under the contract Global Health Activities in Developing Countries to Combat Non‐Communicable Chronic Diseases. Investigators were supported by grants from the Wellcome Trust Research Training Fellowship in Public Health and Tropical Medicine and the Dirección de Gestión de la Investigación at the Pontifica Universidad Católica del Perú. **Authors name:** Antonio Bernabe‐Ortiz; J. Jaime Miranda **Institution:** CRONICAS Center of Excellence in Chronic Diseases, Universidad Peruana Cayetano Heredia and Department of Medicine, School of Medicine, Universidad Peruana Cayetano Heredia (both in Lima, Peru) **Email:** Antonio.Bernabe@upch. pe; jaime.miranda@upch.pe **Possible conflicts of interest (for study authors):** "The authors declare no competing interests." **Sources used for data extraction:** Journal article with results of the trial; trial protocol (published; trial registry) **Trial registration details:** NCT01960972	
***Risk of bias***		
**Bias**	**Authors' judgement**	**Support for judgement**
Random sequence generation (selection bias)	Low risk	Use of a computer‐generated list of random numbers to obtain randomisation sequence of the six villages (clusters)
Allocation concealment (selection bias)	Low risk	The allocation of all the villages or clusters was performed by a statistician at the beginning of the study according to the randomisation sequence.
Blinding of participants and personnel (performance bias) All outcomes	Unclear risk	Inhabitants were not blinded to the intervention, but were only informed of the allocation at the moment of implementation in their village (which included a social marketing campaign). The research team were aware of treatment assignment. It was not clear how the lack of blinding may have introduced performance bias.
Blinding of outcome assessment (detection bias) All outcomes	Low risk	Outcomes were assessed by a separate team of fieldworkers who were blinded to treatment assignment. Primary study outcomes were assessed objectively by standardised techniques.
Incomplete outcome data (attrition bias) All outcomes	Unclear risk	Cumulative loss to follow‐up was n = 950/8987 (10.6%) participants in the intervention and n = 536/5269 (10.2%) participants in the control group. Attrition in the intervention group ranged from 7.7% to 10% at the different time points or steps; with 13.3% at the last time point. For the control group, it ranged between 8% and 10% at the different time points, with 11.7% at the third time point. Reasons for attrition not reported. The study authors stated that a "per‐protocol intention to treat" analysis was conducted. However, no imputation of any data were reported.
Selective reporting (reporting bias)	Low risk	The primary outcomes were reported according to those prespecified in the study protocol (SBP, DBP).
Other bias	Unclear risk	In some clusters (villages), a reduction in blood pressure was observed before the intervention, and potential explanations for this could be a community‐like white coat effect, which, in the case of rural or semi‐urban areas with limited access to healthcare, can also be present.
Recruitment bias (cluster‐RCTs)	Low risk	Recruitment of participants in all selected villages (clusters) conducted before the start of the intervention in the first village (cluster)
Comparability with individually randomised trials (cluster‐RCTs)	Low risk	Results comparable with findings reported by a meta‐analysis of individually randomised RCTs (Peng 2014). "Salt substitutes have been previously tested, mostly in China and mainly on patients with established hypertension, and they show reductions in blood pressure, with a larger effect observed among individuals with hypertension. Similar results have been obtained using home blood pressure measurements."
Loss of clusters (cluster‐RCTs)	Low risk	No loss of clusters
Baseline imbalance (cluster‐RCTs)	Low risk	Differences were observed between villages for age, education, wealth index, BMI, SBP, DBP and hypertension. Models were adjusted for baseline sex, age, years of education, wealth index and BMI.
Incorrect analysis (cluster‐RCTs)	Low risk	The study authors used generalised estimating equations (GEEs) which uses within‐cluster and between‐cluster information to estimate the treatment effect. In addition, they also adjusted the estimates for time (due to the stepped‐wedge design).
Overall risk of bias	Unclear risk	Unclear risk of bias for incomplete outcome data; low risk for baseline imbalance, recruitment bias and loss of clusters

**Table:** Chang 2006

***Study characteristics***		
Methods	**Study design:** Cluster‐randomised controlled trial **Country:** Taiwan **Setting:** Intervention conducted in a veteran's retirement home in the northern region; setting where mortality outcomes were recorded was the Department of Health **Aim of study:** To examine the effects of using potassium‐sparing salt on cardiovascular disease mortality and medical expenditure in elderly veterans **Unit of allocation:** Retirement home kitchens **Start date:** October 1995 **End date:** June 1999 **Relevant study limitations as reported by study authors:** NR **Sample size calculation:** NR	
Participants	**Baseline Characteristics** LSSS intervention *Age*: in years, mean (SD): 75.21 (7.37) *Gender*: Male, % (n/N): 100 (768/768) *Ethnicity/race*: Chinese *Smoking*: NR *Body Mass Index (BMI)*: n = 248, in kg/m^2^, mean (SD): 23.3 (3.5) *Blood pressure status*: Hypertensive, % (n/N): 40.2 (309/768) *Antihypertensive medication used*: NR *Cardiovascular disease or stroke*: NR *Diabetes mellitus*: NR *Renal impairment*: Serum creatinine ≥ 3.5 mg/dL (>= 309 µmol/L), % (n/N): 0 (0/768) *Dietary potassium intake*: NR *Dietary sodium intake*: NR *Urinary potassium excretion*: Potassium‐to‐creatinine ratio, mean (SD): 0.277 (0.143) *Urinary sodium excretion*: Sodium‐to‐creatinine ratio, mean (SD): 1.34 (0.92) Control *Age*: in years, mean (SD): 74.67 (6.7) *Gender*: Male, % (n/N): 100 (1213/1213) *Ethnicity/race*: Chinese *Smoking*: NR *Body Mass Index (BMI)*: n = 391, in kg/m^2^, mean (SD): 23.0 (3.3) *Blood pressure status*: Hypertensive, % (n/N): 40.4 (490/1213) *Antihypertensive medication used*: NR *Cardiovascular disease or stroke*: NR *Diabetes mellitus*: NR *Renal impairment*: Serum creatinine ≥ 3.5 mg/dL (>= 309 µmol/L), % (n/N): 0 (0/1213) *Dietary potassium intake*: NR *Dietary sodium intake*: NR *Urinary potassium excretion*: Potassium‐to‐creatinine ratio, mean (SD): 0.284 (0.142) *Urinary sodium excretion*: Sodium‐to‐creatinine ratio, mean (SD): 1.14 (0.74) **Inclusion criteria:** Kitchens of a veteran's retirement home. Veterans registering into the retired home during the study period, as well as those already resident in the home **Exclusion criteria:** Kitchen preparing meals for a squad of bedridden residents, among others; residents with abnormal kidney function as assessed by serum creatinine concentrations of ≥ 3.5 mg/dL (>= 309 µmol/L) **Pretreatment:** None reported **Method of recruitment of participants:** Potential participants included veterans already resident in the retired home, as well as those who registered into the retired home during the study period. At baseline, resident participants from 10 squads were screened for eligibility before randomisation of the clusters (kitchens). **Informed consent obtained:** Yes, unclear whether written or oral **Clusters:** 5 clusters (Intervention group n = 395; n = 373; Control group n = 390; n = 410; n = 413). ICC calculation and outcome: NR **Subgroups planned/measured:** NR **Subgroups reported:** NR *Participant flow* **Assessed for eligibility:** n = 6 clusters; n = 1553 participants from n = 5 included clusters **Excluded (number with reasons):** n = 1 cluster (preparing meals for a squad of bedridden participants, among others); participants excluded through cluster exclusion NR, n = 2 excluded from other clusters (kidney function did not meet serum creatinine < 3.5 mg/dL) **Randomised:** n = 5 clusters with n = 1551 participants **Allocated to LSSS intervention(s):** Clusters n = 2; participants n = 768 (n = 635 at baseline; n = 133 during the study period) **Allocated to control:** Clusters n = 3; participants n = 1213 (n = 916 at baseline; n = 297 during the study period) **Received allocated LSSS intervention(s):** NR **Did not receive allocated LSSS intervention(s):** NR **Lost to follow‐up (LSSS intervention group):** n = 0 clusters; n = 265 (died n = 189; institutionalised n = 30; moved out n = 46) **Discontinued intervention (LSSS intervention group):** n = 0 clusters; n = 0 participants **Analysed (LSSS intervention group):** n = 2 clusters with n = 692 participants **Excluded from analysis (LSSS intervention group):** n = 0 clusters; n = 16 participants excluded from overall group (n = 1 invalid date of birth, n = 15 dates missing on death certificates; trial arm distribution not reported) **Received allocated control:** NR **Did not receive allocated control:** NR **Lost to follow‐up (control group):** n = 0 clusters; n = 435 (died n = 307; institutionalised n = 50; moved out n = 78) **Discontinued intervention (control group):** n = 0 clusters; n = 0 participants **Analysed (control group):** n = 3 clusters with n = 1085 participants **Excluded from analysis (control group):** n = 0 clusters; n = 16 participants excluded from overall group (n = 1 invalid date of birth, n = 15 dates missing on death certificates; trial arm distribution not reported)	
Interventions	**Intervention Characteristics** LSSS intervention *Theoretical basis*: The long‐term effects of potassium‐enriched salt substitute on cardiovascular mortality needs to be determined. *Description*: Potassium‐enriched salt (49% NaCl; 49% KCl; 2% other additives) *LSSS category*: ≥ 30%KCl *Contains fortificant*: NR *Delivery*: discretionary use *Duration of run‐in period*: none *Duration of active intervention*: mean of 2.6 years; up to 3.7 years *Duration of follow‐up (as reported)*: none *Total duration of study (as reported)*: mean of 2.6 years; up to 3.7 years *Timing*: Gradually introduced by cooks in the intervention kitchens; replaced 'regular' salt within a 4 week‐period. Used throughout for the daily preparation of all meals *Implementation*: LSSS salt used by cooks in intervention kitchens *Providers*: Taiwan Salt Work manufactured the LSSS, which was weighed and delivered to kitchens by research staff. *Co‐interventions*: NR *Resource requirements*: NR *Integrity of delivery*: Amount of LSSS salt used per month recorded in each kitchen. Regular condiments and spices e.g. soy sauce and MSG, not limited. Mean LSSS salt use: 1.41 (0.22) kg/day per kitchen serving approx. 400 participants (approx. 3.8 g per person per day). Mean consumption of monosodium glutamate, soy sauce, vinegar, hot sauce, ketchup, and pickled vegetables during the first 3 mo of the trial in a subsample of 248 participants were 560 mg, 389 mg, 91 mg, 84 mg, 10 mg, and 225 mg per person per day. Control *Theoretical basis*: To enable comparison of LSSS intervention with standard practice *Description*: Regular salt (99.6% NaCl; 0.4% other additives) *Contains fortificant*: NR *Delivery*: discretionary use *Duration of run‐in period*: none *Duration of active intervention*: mean of 2.6 years; up to 3.7 years *Duration of follow‐up (as reported)*: none *Total duration of study (as reported)*: mean of 2.6 years; up to 3.7 years *Timing*: Used throughout for the daily preparation of all meals *Implementation*: Regular salt used by cooks in the control kitchens *Providers*: Taiwan Salt Work manufactured the regular salt, which was weighed and delivered to kitchens by research staff. *Co‐interventions*: NR *Resource requirements*: NR *Integrity of delivery*: Amount of regular salt used per month recorded in each kitchen. Regular condiments and spices e.g. soy sauce and MSG, not limited. Mean regular salt use: NR per kitchen (approx. 5.8 g per person per day reported). Mean consumption of monosodium glutamate, soy sauce, vinegar, hot sauce, ketchup, and pickled vegetables during the first 3 mo of the trial in a subsample of 391 participants: 536 mg, 394 mg, 88mg, 55 mg, 10 mg, and 224 mg per person per day.	
Outcomes	Primary outcomes: **Diastolic Blood Pressure (DBP):** NR **Systolic Blood Pressure (SBP):** NR **Hypertension (as reported, or SBP > 140 mmHg or DBP > 85 mmHg):** NR **Blood pressure control (achieving blood pressure threshold or 'control' as prespecified):** NR **Cardiovascular events‐stroke:** NR **Cardiovascular events‐myocardial infarction:** NR **Cardiovascular events‐other:** NR **Cardiovascular mortality:** Outcome measurement: death registry of Department of Health, using the International Classification of Diseases 9th revision (ICD‐9) code for cause of CVD‐related death. Time points: duration of study period **Blood potassium:** NR **Hyperkalaemia:** NR **Hypokalaemia:** NR Secondary outcomes: **All‐cause mortality:** Outcome measurement: death registry of Department of Health, using the International Classification of Diseases 9th revision (ICD‐9) code for cause of death. Time points: duration of study period **Adverse events:** NR **Antihypertensive medication use:** NR **Body Mass index (BMI):** NR **Serum creatinine:** NR **Albuminuria:** NR **Urinary albumin‐to‐creatinine ratio (uACR):** NR **Fasting blood glucose:** NR **Blood triglycerides:** NR **Total blood cholesterol:** NR **24‐h urinary sodium excretion:** NR **24‐h urinary potassium excretion:** NR	
Notes	**Funding source:** Taiwan Salt Work Company; Academia Sinica **Authors name:** Hsing‐Yi Chang; Wen‐Harn Pan **Institution:** Institute of Biomedical Sciences, Academia Sinica, Taipei **Email:** pan@ibms.sinica.edu.tw **Possible conflicts of interest (for study authors):** "None of the authors had any conflict of interest or any financial relations with the funding agent." **Sources used for data extraction:** Journal article with results of the trial **Trial registration details:** NR	
***Risk of bias***		
**Bias**	**Authors' judgement**	**Support for judgement**
Random sequence generation (selection bias)	Low risk	Randomisation was done by drawing lots.
Allocation concealment (selection bias)	Unclear risk	Insufficient information available
Blinding of participants and personnel (performance bias) All outcomes	Unclear risk	The study authors reported that only participants were blinded. Thus, there may be a possibility for performance bias for study personnel e.g. cooks, research personnel.
Blinding of outcome assessment (detection bias) All outcomes	Low risk	Official coders of the Department of Health used the International Classification of Diseases 9th revision to assign cause of death. Good agreement between these judgements and those of four physicians. Unlikely that the coders would be aware of treatment allocation; the outcomes were also not likely to be influenced by unblinded assessment.
Incomplete outcome data (attrition bias) All outcomes	Low risk	Overall attrition was 76/768 (9.9%) in the intervention and 128/1213 (10.6%) in the control group. No difference in reasons for dropout
Selective reporting (reporting bias)	Unclear risk	No study protocol or prospective trial registry entry available
Other bias	Unclear risk	There was a considerable risk of contamination in the intervention group: "Other condiments and spices such as soy sauce and monosodium glutamate were not limited, because reasonably priced low‐sodium soy sauce and monosodium glutamate were not available at the time of the trial." These condiments accounted for approx. 30% of total sodium intake in a subsample of participants during the first three months of the study. New participants joined the study as they moved into the home; it was not clear whether these persons were assessed for renal function.
Recruitment bias (cluster‐RCTs)	High risk	A significant number of participants were recruited after the study clusters (kitchens) were randomised (LSSS intervention group n = 133; Control group n = 295).
Comparability with individually randomised trials (cluster‐RCTs)	Low risk	"Recently, Cook et al (31) presented the long‐term effects of sodium reduction on CVD based on the data from the Trials of Hypertension Prevention."
Loss of clusters (cluster‐RCTs)	Low risk	No loss of clusters reported
Baseline imbalance (cluster‐RCTs)	Unclear risk	Baseline characteristics were reported as not significantly different, however, height, weight, BMI, blood pressure, hypertension as well as sodium‐ and potassium‐to‐creatinine ratios were determined in a subsample of participants; 248/768 (32.3%) in the intervention and 391/1213 (32.2%) in the control group. Presence of comorbidities not reported. No information was provided on whether this subsample was randomly selected.
Incorrect analysis (cluster‐RCTs)	Low risk	Cluster effects were accounted for in the survival curves.
Overall risk of bias	High risk	Low risk of bias for incomplete outcome data and loss of clusters, unclear risk of bias for baseline imbalance, high risk of recruitment bias

**Table:** CSSS Collaborative Group 2007

***Study characteristics***		
Methods	**Study design:** Randomised controlled trial **Study grouping:** Parallel group **Country:** China **Setting:** Intervention conducted in rural households in northern region; outcomes measured in six regional coordinating centres (Heilongjiang, Tianjin, Liaoning, Shanxi and two centres in rural/suburban Beijing) **Aim of study:** To test the effect of a salt substitute on blood pressure among rural Chinese individuals at high‐risk from cardiovascular disease **Unit of allocation:** adults (aged 18 years or older) **Start date:** May 2004 **End date:** August 2005 **Relevant study limitations as reported by study authors:** The authors reported that they could not know by how much sodium intake was reduced (since 24‐hour urine collections were not conducted), and not performing 24‐hour BP measurements (may increase the ability to detect smaller BP differences). **F. Sample size calculation:** The study was designed to provide 90% power at P = 0.05 to detect a 3.1/1.7 mmHg or greater difference between randomised groups in mean casual follow‐up blood pressure levels.	
Participants	**Baseline Characteristics** LSSS intervention *Age*: in years, mean (SD): 59 (10) *Gender*: Female, % (n/N): 54 (166/306) *Ethnicity/race*: Chinese *Smoking*: Current smoker, % (n/N): 32 (97/306); past smoker, % (n/N): 15 (45/306) *Body Mass Index (BMI)*: in kg/m^2^, mean (SD): 26 (3.6); 25 (3.9) *Blood pressure status*: hypertensive (SBP >= 160 mmHg), % (n/N): 57 (173/306) *Antihypertensive medication used*: any antihypertensive medication, % (n): 61 (185/306); diuretic, % (n): 6 (19/306); ACE inhibitor or ARB, % (n): 10 (31/306); beta‐blocker, % (n): 6 (17/306); calcium antagonist, % (n): 23 (70/306) *Cardiovascular disease or stroke*: History of vascular disease, % (n/N): 62 (189/306) *Diabetes mellitus*: Treated diabetes and aged > 55 years, % (n/N): 16 (48/306) *Renal impairment*: Serum creatinine, in µmol/L, mean (SD): 74.0 (20.0) *Dietary potassium intake*: NR *Dietary sodium intake*: NR *Urinary potassium excretion*: in mmol/L, median (IQR): 20 (12‐31) *Urinary sodium excretion*: in mmol/L, median (IQR): 151 (92‐201) Control *Age*: in years, mean (SD): 61 (9.7) *Gender*: Female, % (n/N): 58 (174/302) *Ethnicity/race*: Chinese *Smoking*: Current smoker, % (n/N): 28 (85/302); past smoker, % (n/N): 14 (41/302) *Body Mass Index (BMI)*: in kg/m^2^, mean (SD): 25 (3.9) *Blood pressure status*: hypertensive (SBP >= 160 mmHg), % (n): 57 (172/302) *Antihypertensive medication used*: any antihypertensive medication, % (n/N): 61 (184/302); diuretic, % (n/N): 3 (10/302); ACE inhibitor or ARB, % (n/N): 11 (32/302); beta‐blocker, % (n/N): 4 (13/302); calcium antagonist, % (n/N): 23 (68/302) *Cardiovascular disease or stroke*: History of vascular disease, % (n/N): 66 (200/302) *Diabetes mellitus*: Treated diabetes and aged > 55 years, % (n/N): 19 (57/302) *Renal impairment*: Serum creatinine, in µmol/L, mean (SD): 74.5 (19.1) *Dietary potassium intake*: NR *Dietary sodium intake*: NR *Urinary potassium excretion*: in mmol/L, median (IQR): 20 (14‐31) *Urinary sodium excretion*: in mmol/L, median (IQR): 154 (94‐200) **Inclusion criteria:** Adults (>= 18 years), judged by a doctor's diagnosis, to be at high risk of future vascular disease, defined as a history of any of the following: stroke or transient ischaemic attack; hospitalisation for management of any acute coronary syndrome; surgery or angioplasty for peripheral vascular disease; diabetes and >= 55 years, or SBP > 160 mmHg. In addition, participants were required to have an estimated sodium intake of 260 mmol/24hrs or the expectation that at least 50% of daily dietary salt intake could be replaced with the LSSS (estimated via interview of the potential participant and the individual responsible for daily food preparation (if this was not the patient) using a structured questionnaire). **Exclusion criteria:** Participants were excluded if they were on potassium‐sparing medication, or if there was a history of significant renal impairment. Persons with physician‐determined abnormal blood tests for serum creatinine and potassium before and after run‐in on the salt substitute; any family member in the household of otherwise eligible participants with a contraindication to the intervention or control, as assessed by a physician **Pretreatment:** Age, anticoagulant use and BMI were reported as showing 'chance baseline imbalance' (no formal hypothesis tests reported). **Method of recruitment of participants:** All potential participants attended regional co‐ordinating centres. **Informed consent obtained:** Yes, unclear whether written or oral **Clusters:** n/a **Subgroups planned/measured:** Based on baseline characteristics (not specified) **Subgroups reported:** NR *Participant flow* **Assessed for eligibility:** n = 752 **Excluded (number with reasons):** n = 144 in total (n = 52 withdrew before run‐in period; reasons not recorded); n = 92 during run‐in period (died n = 2; serious event n = 2; reluctant to continue with follow‐up visits n = 57; other n = 31) **Randomised:** n = 608 **Allocated to LSSS intervention(s):** n = 306 **Allocated to control:** n = 302 **Received allocated LSSS intervention(s):** NR **Did not receive allocated LSSS intervention(s):** NR **Lost to follow‐up (LSSS intervention group):** n = 14 (died n = 4; withdrew n = 1; hospitalised n = 3; lost contact n = 6) **Discontinued intervention (LSSS intervention group):** n = 1 **Analysed (LSSS intervention group):** n = 292 (blood pressure); n = 287 (urinary measurements) **Excluded from analysis (LSSS intervention group):** n = 0 **Received allocated control:** NR **Did not receive allocated control:** NR **Lost to follow‐up (control group):** n = 9 (died n = 4; hospitalised n = 3; lost contact n = 2) **Discontinued intervention (control group):** n = 0 **Analysed (control group):** n = 293 (blood pressure); n = 290 (urinary measurements) **Excluded from analysis (control group):** n = 0	
Interventions	**Intervention Characteristics** LSSS intervention *Theoretical basis*: Salt intake is high in rural northern China where most dietary salt comes from foods cooked at home. Replacement of salt with LSSS intervention may result in large effects on blood pressure. *Description*: 'Reduced‐sodium high potassium' salt substitute (65% NaCl, 25% KCl, 10% MgSO_4_) replaced household salt. *LSSS category*: < 30% KCl *Contains fortificant*: NR *Delivery*: discretionary use *Duration of run‐in period*: 4 weeks (using salt substitute) *Duration of active intervention*: 12 months *Duration of follow‐up (as reported)*: none *Total duration of study (as reported)*: 12 months *Timing*: Used throughout for all cooking, pickling and other uses in the household. *Implementation*: In 1 kg identical bags, with up to 3 kg delivered monthly *Providers*: Beijing Salt Industrial Company; details of persons providing the LSSS salt not reported *Co‐interventions*: NR *Resource requirements*: NR *Integrity of delivery*: Existing household salt and foods previously pickled in salt were not removed from participants' households. Use of salt substitute after randomisation was determined at each visit, by asking participants which proportion of household salt used was provided by the study and recorded as 'all', 'nearly all', 'half', 'less than half' or 'none'. Self‐reported use of study salt (use of salt for all or nearly all of their day‐to‐day food preparation): 99% (both groups). Control *Theoretical basis*: To enable comparison of LSSS intervention with standard practice *Description*: 'Normal' salt (100% NaCl) used by household *Contains fortificant*: NR *Delivery*: discretionary use *Duration of run‐in period*: 4 weeks (using salt substitute) *Duration of active intervention*: 12 months *Duration of follow‐up (as reported)*: none *Total duration of study (as reported)*: 12 months *Timing*: Used throughout for all cooking, pickling and other uses in the household *Implementation*: In identical 1 kg bags, with up to 3 kg delivered monthly *Providers*: Beijing Salt Industrial Company; details of persons providing the regular salt not reported *Co‐interventions*: NR *Resource requirements*: NR *Integrity of delivery*: Use of 'normal' salt after randomisation was determined at each visit, by asking participants which proportion of household salt used was provided by the study and recorded as 'all', 'nearly all', 'half', 'less than half' or 'none'. Self‐reported use of study salt (use of salt for all or nearly all of their day‐to‐day food preparation): 99% (both groups)	
Outcomes	Primary outcomes: **Diastolic Blood Pressure (DBP):** Outcome measurement: participants seated at rest for at least 5 minutes beforehand, using an automatic sphygmomanometer and taking the average of two measurements done at least 2 minutes apart at follow‐up visits; home self‐BP measurements taken by participants with hypertension and their family members. Time points: 2, 3, 6, 9 and 12 months **Systolic Blood Pressure (SBP):** Outcome measurement: participants seated at rest for at least 5 minutes beforehand, using an automatic sphygmomanometer and taking the average of two measurements done at least 2 minutes apart at follow‐up visits; home self‐BP measurements taken by participants with hypertension and their family members. Time points: 2, 3, 6, 9 and 12 months **Hypertension (as reported, or SBP > 140 mmHg or DBP > 85 mmHg):** NR **Blood pressure control (achieving blood pressure threshold or 'control' as prespecified):** NR **Cardiovascular events‐stroke:** NR **Cardiovascular events‐myocardial infarction:** NR **Cardiovascular events‐other:** Serious cardiovascular events; outcome measurement: NR; time points: during the study period **Cardiovascular mortality:** NR **Blood potassium:** NR **Hyperkalaemia:** Outcome measurement: NR; time points: during the study period **Hypokalaemia:** NR Secondary outcomes: **All‐cause mortality:** Outcome measurement: NR; time points: during the study period **Adverse events:** Outcome measurement: NR; time points: during the study period **Antihypertensive medication use:** NR **Body Mass index (BMI):** NR **Serum creatinine:** NR **Albuminuria:** NR **Urinary albumin‐to‐creatinine ratio (uACR):** NR **Fasting blood glucose:** NR **Blood triglycerides:** NR **Total blood cholesterol:** NR **24‐h urinary sodium excretion:** NR **24‐h urinary potassium excretion:** NR	
Notes	**Funding source:** The study received financial support from The George Institute for International Health (Australia), The Clinical Trials Research Unit (New Zealand), The Capital Medical Science Development Fund (China) and the National Health and Medical Research Council of Australia (grand ID 358395). Study staff received grants from the High Blood Pressure Research Council of Australia, the University of Sydney, the National Health and Medical Research Council of Australia and the National Heart Foundation of Australia. **China Salt Substitute Study (CSSS)** **Authors name:** Bruce Neal, Wu Yangfeng, on behalf of The China Salt Substitute Study Collaborative Group **Institution:** The George Institute for International Health, University of Sydney; The George Institute for International Health, China **Email:** bneal @george.org.au; ywu@george.org.cn **Possible conflicts of interest (for study authors):** "None of the collaborative group has any conflict to declare in regard to this work." **Sources used for data extraction:** Journal articles with results of the trial **Trial registration details:** NCT00145756	
***Risk of bias***		
**Bias**	**Authors' judgement**	**Support for judgement**
Random sequence generation (selection bias)	Low risk	Computerised random number sequence generation with stratification within each centre by baseline SBP (3 strata) and use of antihypertensive therapy
Allocation concealment (selection bias)	Low risk	Central computerised randomisation, accessed by study physicians via the study website (with back‐up phone and fax services)
Blinding of participants and personnel (performance bias) All outcomes	Low risk	Participants and investigators were blinded.
Blinding of outcome assessment (detection bias) All outcomes	Low risk	Centre physicians were blinded.
Incomplete outcome data (attrition bias) All outcomes	Low risk	Overall attrition was low, with 14/306 (4.6%) in the intervention and 9/302 (3.0%) in the control group. Intention‐to‐treat analyses with no imputation were conducted.
Selective reporting (reporting bias)	High risk	All primary and secondary outcomes reported according to prespecified outcomes in the study protocol (NCT 00145756). However, the study protocol was registered after completion of the study. A substudy reported on primary outcomes such as central systolic and diastolic blood pressure (CSP; CDP), central pulse pressure (CPP), reflection time (RT), AUG and AIx that were not prespecified in the protocol.
Other bias	Low risk	None identified
Overall risk of bias	Low risk	Low risk of bias for allocation concealment and incomplete outcome data

**Table:** Geleijnse 1994

***Study characteristics***		
Methods	**Study design:** Randomised controlled trial **Study grouping:** Parallel group **Country:** The Netherlands **Setting:** Intervention conducted in households in Rotterdam; outcomes measured at a university study centre **Aim of study:** To decrease blood pressure in older subjects with mild to moderate hypertension by reducing sodium and increasing potassium and magnesium intake **Unit of allocation:** adults (aged 18 years or older) **Start date:** NR **End date:** NR **Relevant study limitations as reported by study authors:** Urinary excretion of magnesium did not reflect a higher level of intake in the intervention group when compared to control (even though differences in sodium and potassium were observed). Blood pressure was measured at follow‐up, but no data on urinary electrolytes were collected post‐intervention. The authors acknowledged that an older study population, manipulating three minerals at the same time, as well as the characteristics of the intervention salt (such as double salt structures), may have led to larger blood pressure reductions than what was observed in other studies. **Sample size calculation:** The study was powered to detect a difference of 5 mmHg systolic blood pressure and 4 mmHg diastolic blood pressure, with 90% power and alpha of 5% using a two‐tailed test of significance.	
Participants	**Baseline Characteristics** LSSS intervention *Age*: in years, mean (SD): 65.7 (4.6) *Gender*: Female, % (n/N): 47% (23/49) *Ethnicity/race*: European *Smoking*: Smokers, % (n/N): 18.4 (9/49) *Body Mass Index (BMI)*: in kg/m^2^, mean (SD): 27.1 (3.4) *Blood pressure status*: Hypertensive, % (n/N): 100 (49/49) *Antihypertensive medication used*: Use of antihypertensive medication, % (n/N): 0 (0/49) *Cardiovascular disease or stroke*: NR *Diabetes mellitus*: Excluded *Renal impairment*: Serum creatinine > 200 µmol/L, % (n/N): 0 (0/49) *Dietary potassium intake*: NR *Dietary sodium intake*: NR *Urinary potassium excretion*: in mmol/24 h, mean (SD): 86 (22) *Urinary sodium excretion*: in mmol/24 h, mean (SD): 139 (52) Control *Age*: in years, mean (SD): 67.1 (4.5) *Gender*: Female, % (n/N): 51 (26/51) *Ethnicity/race*: European *Smoking*: Smokers, % (n/N): 23.5 (12/51) *Body Mass Index (BMI)*: in kg/m^2^, mean (SD): 27.2 (3.2) *Blood pressure status*: Hypertensive, % (n/N): 100 (51/51) *Antihypertensive medication used*: Use of antihypertensive medication, % (n/N): 0 (0/51) *Cardiovascular disease or stroke*: NR *Diabetes mellitus*: Excluded *Renal impairment*: Serum creatinine > 200 µmol/L, % (n/N): 0 (0/51) *Dietary potassium intake*: NR *Dietary sodium intake*: NR *Urinary potassium excretion*: in mmol/24 h, mean (SD): 81 (25) *Urinary sodium excretion*: in mmol/24 h, mean (SD): 138 (50) **Inclusion criteria:** Non‐hospitalised men and women who were aged 55 to 75 years; not on antihypertensive medication; and had a systolic blood pressure between 140 and 200 mmHg as well as diastolic blood pressure between 85 and 110 mmHg at two measurements taken a week apart **Exclusion criteria:** Participants with systolic blood pressure below 130 mmHg or diastolic blood pressure below 70 mmHg; a history of myocardial infarction, angina pectoris, diabetes mellitus or impaired renal function (defined as serum creatinine > 200 µmol/L); or on a salt‐restricted diet on medical advice **Pretreatment:** None **Method of recruitment of participants:** Potentially eligible participants were identified as hypertensive from a population‐based cohort study (the Rotterdam study) measuring their blood pressure. They were invited, either by letter or telephone, for remeasurement of their blood pressure for this study. **Informed consent obtained:** Yes, written **Clusters:** n/a **Subgroups planned/measured:** Sex and age **Subgroups reported:** Sex and age (narratively) *Participant flow* **Assessed for eligibility:** n = 419 **Excluded (number with reasons):** n = 319 (n = 30 could not be contacted; n = 56 showed no interest; n = 69 met one of the exclusion criteria; n = 17 did not fulfil blood pressure criteria; n = 40 started antihypertensive treatment; n = 55 met other exclusion criteria; n = 52 refused the trial) **Randomised:** n = 100 **Allocated to LSSS intervention(s):** n = 49 **Allocated to control:** n = 51 **Received allocated LSSS intervention(s):** NR **Did not receive allocated LSSS intervention(s):** NR **Lost to follow‐up (LSSS intervention group):** n = 1 **Discontinued intervention (LSSS intervention group):** n = 1 (disliked intervention foods) **Analysed (LSSS intervention group):** n = 49 **Excluded from analysis (LSSS intervention group):** n = 0 **Received allocated control:** NR **Did not receive allocated control:** NR **Lost to follow‐up (control group):** n = 2 **Discontinued intervention (control group):** n = 2 (n = 2 admitted to hospital for unrelated complaints) **Analysed (control group):** n = 51 **Excluded from analysis (control group):** n = 0	
Interventions	**Intervention Characteristics** LSSS intervention *Theoretical basis*: It has been suggested that minerals play a role in blood pressure regulation; with sodium increasing blood pressure, and potassium and magnesium lowering blood pressure. Dietary measures to reduce blood pressure may be more effective when the intake of several minerals is manipulated. *Description*: Mineral salt (41% NaCl; 41% KCl; 17% Mg salts (potassium magnesium double salts‐carnallite and kainite); 1% trace minerals) combined with trial foods *LSSS category*: ≥ 30% KCl *Contains fortificant*: NR *Delivery*: Discretionary and non‐discretionary use *Duration of run‐in period*: none *Duration of active intervention*: 24 weeks *Duration of follow‐up (as reported)*: 4 to 9 months *Total duration of study (as reported)*: 10 to 15 months *Timing*: Salt used throughout for cooking and as table salt. Trial foods prepared with LSSS used throughout the study *Implementation*: All interventions were provided which included e.g. bread, cheese, luncheon meats, canned and instant soups, smoked sausage (57% of the salt intake of elderly Dutch). Salt and foods were supplied at a ward building. *Providers*: Akzo Nobel manufactured and provided the LSSS; Friesland Frico Domo, Nistria Dieetvleeswaren BV, UVG Nederland BV, Nestlé Nederland BV, Meneba Meel BV and Quality Bakers developed the trial foods. Ladies at the ward building provided the LSSS and foods to participants. *Co‐interventions*: Participants were instructed not to change dietary and lifestyle habits and to adhere to trial salt and foods as much as possible and to record any deviations in a diary. *Resource requirements*: NR *Integrity of delivery*: Participants had to note deviations in a diary and were contacted monthly to improve compliance. Salt use was assessed by weighing. Recorded salt and foods provided and diaries showed good adherence to assigned intervention. Discretionary salt use was reported as higher in 16/40 (40%) of subjects; salt use was mean 5.2 g (SD 4.9 g; ranging from 0 to 26.2 g). Control *Theoretical basis*: To enable comparison of LSSS intervention with standard practice *Description*: Common salt combined with trial foods *Contains fortificant*: NR *Delivery*: Discretionary and non‐discretionary use *Duration of run‐in period*: none *Duration of active intervention*: 24 weeks *Duration of follow‐up (as reported)*: 4 to 9 months *Total duration of study (as reported)*: 10 to 15 months *Timing*: Salt used throughout for cooking and as table salt. Trial foods prepared with common salt used throughout the study *Implementation*: All interventions were provided which included e.g. bread, cheese, luncheon meats, canned and instant soups, smoked sausage (57% of the salt intake of elderly Dutch). Salt and foods were supplied at a ward building. *Providers*: Friesland Frico Domo, Nistria Dieetvleeswaren BV, UVG Nederland BV, Nestlé Nederland BV, Meneba Meel BV and Quality Bakers developed the trial foods. Ladies at the ward building provided the common salt and foods to participants. *Co‐interventions*: Participants were instructed not to change dietary and lifestyle habits and to adhere to trial salt and foods as much as possible and to record any deviations in a diary. *Resource requirements*: NR *Integrity of delivery*: Participants had to note deviations in a diary and were contacted monthly to improve compliance. Salt use was assessed by weighing. Recorded salt and foods provided and diaries showed good adherence to assigned control. Discretionary salt use was reported as higher in 3/44 (7%) of subjects; salt use was mean 4.9 g (SD 4.8 g; ranging from 0 to 24.1 g).	
Outcomes	Primary outcomes: **Diastolic Blood Pressure (DBP):** Outcome measurement: Measurements taken with automatic device in erect position by 2 investigators. Four measurements taken after 5 minutes rest; last three measurements were averaged. Measurements were taken at two time intervals (with one week apart) around weeks 8 and 16, and at 3 intervals for week 24. For each time point, the average of these measurements was calculated. Time points: 8, 16, 24 weeks **Systolic Blood Pressure (SBP):** Outcome measurement: Measurements taken with automatic device in erect position by 2 investigators. Four measurements taken after 5 minutes rest; last three measurements were averaged. Measurements were taken at two time intervals (with one week apart) around weeks 8 and 16, and at 3 intervals for week 24. For each time point, the average of these measurements was calculated. Time points: 8, 16, 24 weeks **Hypertension (as reported, or SBP > 140 mmHg or DBP > 85 mmHg):** NR **Blood pressure control (achieving blood pressure threshold or 'control' as prespecified):** NR **Cardiovascular events‐stroke:** NR **Cardiovascular events‐myocardial infarction:** NR **Cardiovascular events‐other:** NR **Cardiovascular mortality:** NR **Blood potassium:** Outcome measurement: Performed using standard methods in serum. Time points: 24 weeks **Hyperkalaemia:** NR **Hypokalaemia:** NR Secondary outcomes: **All‐cause mortality:** NR **Adverse events:** NR **Antihypertensive medication use:** NR **Body Mass index (BMI):** NR **Serum creatinine:** NR **Albuminuria:** NR **Urinary albumin‐to‐creatinine ratio (uACR):** NR **Fasting blood glucose:** NR **Blood triglycerides:** NR **Total blood cholesterol: **Outcome measurements: Analysed from serum frozen at ‐20° C using an automated enzymatic procedure. Time points: 24 weeks **24‐h urinary sodium excretion:** 24‐hour urine sample. Time points: 24 weeks **24‐h urinary potassium excretion:** 24‐hour urine sample. Time points: 24 weeks	
Notes	**Funding source:** The study was funded by Akzo Nobel (the provider of the intervention salt) and the Rotterdam Medical Research Foundation (ROMERES). **Authors name:** J.M. Geleijnse **Institution:** Department of Epidemiology and Biostatistics, Erasmus University Medical School **Email:** Marianne.Geleijnse@wur.nl **Possible conflicts of interest (for study authors):** NR **Sources used for data extraction:** Journal articles with results of the trial **Trial registration details:** NR	
***Risk of bias***		
**Bias**	**Authors' judgement**	**Support for judgement**
Random sequence generation (selection bias)	Low risk	Randomisation was done using a computerised randomisation table.
Allocation concealment (selection bias)	Unclear risk	Insufficient information on how the randomisation sequence was protected
Blinding of participants and personnel (performance bias) All outcomes	Low risk	Salt and foods looked identical and were provided based on a double‐blind coding system. Outcomes were unlikely to have been influenced by a lack of blinding.
Blinding of outcome assessment (detection bias) All outcomes	Low risk	It was not reported whether outcome assessors were blinded to allocation. Blood pressure was measured by two investigators, which would minimise any potential detection bias. Blood electrolytes and lipids were done using standard laboratory procedures.
Incomplete outcome data (attrition bias) All outcomes	High risk	Overall attrition was very low, with 1/49 (2%) participants in the intervention and 2/51 (4%) participants in the control group withdrawing. Missing data was considerably higher for blood potassium (4/49 (8%) participants in the intervention and 8/51 (16%) participants in the control group not contributing data) and total cholesterol (3/49 (6%) participants in the intervention and 8/51 (16%) participants in the control group not contributing data).
Selective reporting (reporting bias)	Unclear risk	No study protocol or prospective trial registry entry available
Other bias	Low risk	None identified
Overall risk of bias	High risk	Unclear risk of bias for allocation concealment; high risk of bias for incomplete outcome data

**Table:** Gilleran 1996

***Study characteristics***		
Methods	**Study design:** Randomised controlled trial **Study grouping:** Parallel group **Country:** United Kingdom **Setting:** Intervention conducted in households in Birmingham; outcomes measured at a diabetic outpatient clinic **Aim of study:** To assess the effect of dietary sodium reduction, and supplementation of potassium and magnesium, on blood pressure and metabolic parameters of hypertensive type 2 diabetics **Unit of allocation:** adults (aged 18 years or older) **Start date:** NR **End date:** NR **Relevant study limitations as reported by study authors:** Biochemical parameters, both in serum and urine, did not change to reflect changes in potassium and magnesium intake, and the authors ascribed this to the possibility that changes in dietary intake were not large enough to detect a difference. It was not possible to determine from this trial which of the three mineral interventions (sodium reduction, potassium increase, or magnesium increase) reduced blood pressure. **Sample size calculation:** NR	
Participants	**Baseline Characteristics** LSSS intervention *Age*: in years, mean (SD): 62.5 (7.8) *Gender*: Female, % (n/N): 40 (8/20) *Ethnicity/race*: European *Smoking*: NR *Body Mass Index (BMI)*: in kg/m^2^, mean (SD): 28.1 (4.6) *Blood pressure status*: Hypertensive, % (n/N): 100 (20/20) participants with hypertension; duration of hypertension in years, mean (SD): 3.3 (4.6) *Antihypertensive medication used*: On prior antihypertensive medication (stopped 1 month prior to randomisation), % (n/N): 45 (9/20) *Cardiovascular disease or stroke*: NR *Diabetes mellitus*: Type 2 diabetic, % (n/N): 100 (20/20); on hypoglycaemics, % (n/N): 50 (10/20); duration of DM in years, mean (SD): 3.9 (3.4) *Renal impairment*: Hypertensive nephropathy (persistent proteinuria or raised serum creatinine > 130 µmol/L), % (n/N): 0 (0/20) *Dietary potassium intake*: NR *Dietary sodium intake*: NR *Urinary potassium excretion*: mmol/24 h estimation, mean (SD): 58.8 (27) *Urinary sodium excretion*: mmol/24 h estimation, mean (SD): 146.4 (73) Control *Age*: in years, mean (SD): 59.2 (10.8) *Gender*: Female, % (n/N): 40 (8/20) *Ethnicity/race*: European *Smoking*: NR *Body Mass Index (BMI)*: in kg/m^2^, mean (SD): 28.6 (3.7) *Blood pressure status*: Hypertensive, % (n/N): 100 (20/20) participants with hypertension; duration of hypertension in years, mean (SD): 2 (4.5) *Antihypertensive medication used*: On prior antihypertensive medication (stopped 1 month prior to randomisation), % (n/N): 50 (10/20) *Cardiovascular disease or stroke*: NR *Diabetes mellitus*: Type 2 diabetic, % (n/N): 100 (20/20); on hypoglycaemics, % (n/N): 45 (9/20); duration of DM in years, mean (SD): 5.8 (5.8) *Renal impairment*: Hypertensive nephropathy (persistent proteinuria or raised serum creatinine > 130 µmol/L), % (n/N): 0 (0/20) *Dietary potassium intake*: NR *Dietary sodium intake*: NR *Urinary potassium excretion*: mmol/24 h estimation, mean (SD): 80.4 (32) *Urinary sodium excretion*: mmol/24 h estimation, mean (SD): 168.9 (83) **Inclusion criteria:** Established diabetic patients with hypertension, defined as three consecutive blood pressure readings of > 160 mmHg for systolic and > 95 mmHg for diastolic blood pressure. Participants who were on antihypertensives were included if they had ceased medication at least one month prior to the trial and met hypertensive criteria. **Exclusion criteria:** Persons with poor or unstable diabetic control; on antihypertensive medication at the start of the study; who are treated with insulin; who had evidence of diabetic or hypertensive nephropathy, defined as persistent proteinuria on Albustix or elevated serum creatinine of 130 µmol/L; who have cardiac failure, are pregnant, or are already on a low‐sodium diet were excluded. **Pretreatment:** None. **Method of recruitment of participants:** Recruited from a diabetic clinic at a hospital in Birmingham **Informed consent obtained:** Yes (written) **Clusters:** n/a **Subgroups planned/measured:** NR **Subgroups reported:** NR *Participant flow* **Assessed for eligibility:** NR **Excluded (number with reasons):** NR **Randomised:** n = 40 **Allocated to LSSS intervention(s):** n = 20 **Allocated to control:** n = 20 **Received allocated LSSS intervention(s):** NR **Did not receive allocated LSSS intervention(s):** NR **Lost to follow‐up (LSSS intervention group):** n = 9 (n = 4 due to hypertensive criteria; n = 5 for other reasons) **Discontinued intervention (LSSS intervention group):** n = 9 (n = 4 due to hypertensive criteria; n = 5 for other reasons) **Analysed (LSSS intervention group):** n = 11 **Excluded from analysis (LSSS intervention group):** n = 0 **Received allocated control:** NR **Did not receive allocated control:** NR **Lost to follow‐up (control group):** n = 12 (n = 10 due to hypertensive criteria; n = 2 for other reasons) **Discontinued intervention (control group):** n = 12 (n = 10 due to hypertensive criteria; n = 2 for other reasons) **Analysed (control group):** n = 8 **Excluded from analysis (control group):** n = 0	
Interventions	**Intervention Characteristics** LSSS intervention *Theoretical basis*: Longer‐term dietary increases in potassium and magnesium combined with sodium reduction may have a therapeutic effect on the blood pressure and metabolic parameters of type 2 diabetics. *Description*: Salt substitute (Seltin) (50% NaCl, 40% KCl, 10% MgSO4) used as added salt at the table by participants *LSSS category*: ≥ 30% KCl *Contains fortificant*: NR *Delivery*: Discretionary use *Duration of run‐in period*: 1 month *Duration of active intervention*: 9 months *Duration of follow‐up (as reported)*: none *Total duration of study (as reported)*: 9 months *Timing*: LSSS to be used throughout the study in cooking as well as for table salt. *Implementation*: Unlabelled packs containing one container of LSSS (500 g) and two salt cellars (90 g) of LSSS were given monthly. *Providers*: LSSS supplied by Cederoths (Sweden); unlabelled salt packs provided by investigators *Co‐interventions*: NR *Resource requirements*: NR *Integrity of delivery*: Participants were asked about their salt use during dietary recall. Unlabelled packs and salt cellars were returned at each monthly visit and the amount returned was recorded, with good compliance defined as < 25% of the allotted LSSS. Good compliance was reported for 19/20 participants. Control *Theoretical basis*: To enable comparison of LSSS intervention with standard practice *Description*: 'Ordinary' salt used as added salt at the table by participants *Contains fortificant*: NR *Delivery*: Discretionary use *Duration of run‐in period*: 1 month *Duration of active intervention*: 9 months *Duration of follow‐up (as reported)*: none *Total duration of study (as reported)*: 9 months *Timing*: Ordinary salt to be used throughout the study in cooking as well as for table salt *Implementation*: Unlabelled packs containing one container of ordinary salt (500 g) and two salt cellars (90 g) of ordinary salt were given monthly. *Providers*: Ordinary salt supplied by Cederoths (Sweden); unlabelled salt packs provided by investigators. Ordinary salt supplied by Cederoths (Sweden); unlabelled salt packs provided by investigators *Co‐interventions*: NR *Resource requirements*: NR *Integrity of delivery*: Participants were asked about their salt use during dietary recall. Unlabelled packs and salt cellars were returned at each monthly visit and the amount returned was recorded, with good compliance defined as < 25% of the allotted ordinary salt. Good compliance was reported for 19/20 participants.	
Outcomes	Primary outcomes: **Diastolic Blood Pressure (DBP):** Outcome measurement: Measurements with Hawksley random zero sphygmomanometer in supine (after 5‐min rest) and erect (after 2‐min rest) position by a blinded observer at follow‐up visits. Time points: 3, 6, 9 months **Systolic Blood Pressure (SBP):** Outcome measurement: Measurements with Hawksley random zero sphygmomanometer in supine (after 5‐min rest) and erect (after 2‐min rest) position by a blinded observer at follow‐up visits. Time points: 3, 6, 9 months **Hypertension (as reported, or SBP > 140 mmHg or DBP > 85 mmHg):** NR **Blood pressure control (achieving blood pressure threshold or 'control' as prespecified):** NR **Cardiovascular events‐stroke:** Outcome measurement: NR. Time points: 3 months **Cardiovascular events‐myocardial infarction:** NR **Cardiovascular events‐other:** NR **Cardiovascular mortality:** NR **Blood potassium:** NR **Hyperkalaemia:** NR **Hypokalaemia:** NR Secondary outcomes: **All‐cause mortality:** NR **Adverse events:** NR **Antihypertensive medication use:** NR **Body Mass index (BMI):** NR **Serum creatinine:** NR **Albuminuria:** NR **Urinary albumin‐to‐creatinine ratio (uACR):** NR **Fasting blood glucose:** NR **Blood triglycerides:** Outcome measurement: Determined using specific enzymatic methods on Hitachi 737 and DAX 48 analysers. Time points: 3, 6, 9 months **Total blood cholesterol:** Outcome measurement: Determined using specific enzymatic methods on Hitachi 737 and DAX 48 analysers. Time points: 3, 6, 9 months **24‐h urinary sodium excretion:** 24‐hour urine sample. Time points: 9 months **24‐h urinary potassium excretion:** 24‐hour urine sample. Time point: 9 months	
Notes	**Funding source:** NR **Authors name:** P.M. Dodson **Institution:** Department of Diabetes and Chemical Pathology, Birmingham Heartlands Hospital, **Email:** paul.dodson@heartofengland.nhs.uk **Possible conflicts of interest (for study authors):** NR **Sources used for data extraction:** Journal article(s) with results of the trial **Trial registration details:** NR	
***Risk of bias***		
**Bias**	**Authors' judgement**	**Support for judgement**
Random sequence generation (selection bias)	Unclear risk	No information on how the randomisation sequence was generated
Allocation concealment (selection bias)	Unclear risk	Insufficient information on how the randomisation sequence was protected
Blinding of participants and personnel (performance bias) All outcomes	Low risk	Participants were assigned to the intervention and control in a blinded manner. The intervention and control salts were provided in unlabelled packs, and participants found the LSSS and unlabelled salt to be palatable and indistinguishable from their salt intake prior to the intervention. Outcomes were unlikely to be affected by a lack of blinding.
Blinding of outcome assessment (detection bias) All outcomes	Low risk	Blood pressure was measured by an observer blinded to treatment allocation using standard methods. It was not reported whether triglycerides and cholesterol were assessed blindly, but blood lipids are laboratory‐assessed outcomes unlikely to be influenced by a lack of blinding.
Incomplete outcome data (attrition bias) All outcomes	High risk	Overall attrition was 5/20 (25%) in the intervention and 2/20 (10%) in the control group, all for 'other reasons'. These reasons were not explained in sufficient detail to determine whether withdrawal was differential by reason. An additional 4/20 (20%) in the intervention and 10/20 (50%) in the control group were withdrawn due to hypertensive criteria, and did not contribute further data to blood pressure and blood lipid outcomes.
Selective reporting (reporting bias)	Unclear risk	No study protocol or prospective trial registry entry available
Other bias	Low risk	None identified
Overall risk of bias	High risk	Unclear risk of bias for allocation concealment; high risk of bias for incomplete outcome data

**Table:** Hu 2018

***Study characteristics***		
Methods	**Study design:** Cluster‐randomised controlled trial **Country:** China **Setting:** Intervention conducted in rural households in northern region; outcomes measured at community health centres in Beijing **Aim of study:** "... to examine the home BP response to a salt substitute by age, baseline BP and gender subgroup among 220 households including 220 patients with hypertension and 380 families aged 18 years or older who participated in the China Salt Substitute Study." **Unit of allocation:** Households/families **Start date:** September 2005 **End date:** NR **Relevant study limitations as reported by study authors:** Use of morning mid‐stream urine sample instead of a 24‐hour urine sample or conduct dietary recalls to obtain more reliable salt consumption data; seasonal BP changes; duration of intervention too short; limited generalisability of study findings to other areas **Sample size calculation:** Calculated using an expected difference of 5.0 mmHg SBP (based on the data from the China Salt Substitute study), with a SD of 15 mmHg, power of 90% and confidence level of 5%	
Participants	**Baseline Characteristics** LSSS intervention (participants with hypertension) *Age*: in years, mean (SD): 57.1 (10.9) *Gender*: Female, % (n/N): 33.6 (37/110) *Ethnicity/race*: Chinese *Smoking*: NR *Body Mass Index (BMI)*: in kg/m^2^, mean (SD): 27.6 (3.3) *Blood pressure status*: Hypertensive, % (n/N):100 (110/110) *Antihypertensive medication used*, % (n/N): 71.8 (79/110) *Cardiovascular disease or stroke*: NR *Diabetes mellitus*: NR *Renal impairment*: Serum creatinine > 177 µmol/L, % (n/N): 0 (0/110) *Dietary potassium intake*: NR *Dietary sodium intake*: NR *Urinary potassium excretion*: in mmol/L, median (IQR): 21.2 (14.3 to 33.1) *Urinary sodium excretion*: in mmol/L, median (IQR): 125.1 (80.0 to 149.8) Control (participants with hypertension) *Age*: in years, mean (SD): 57.6 (10.1) *Gender*: Female, % (n/N): 60 (66/110) *Ethnicity/race*: Chinese *Smoking*: NR *Body Mass Index (BMI)*: in kg/m^2^, mean (SD): 28.3 (3.5) *Blood pressure status*: Hypertensive, % (n/N):100 (110/110) *Antihypertensive medication used*, % (n/N): 77.3 (85/110) *Cardiovascular disease or stroke*: NR *Diabetes mellitus*: NR *Renal impairment*: Serum creatinine > 177 µmol/L, % (n/N): 0 (0/110) *Dietary potassium intake*: NR *Dietary sodium intake*: NR *Urinary potassium excretion*: in mmol/L, median (IQR): 24.5 (14.0 to 25.3) *Urinary sodium excretion*: in mmol/L, median (IQR): 127.2 (98.5 to 153.2) LSSS intervention (family members) *Age*: in years, mean (SD): 45.5 (17.5) *Gender*: Female, % (n/N): 45.5 (85/187) *Ethnicity/race*: Chinese *Smoking*: NR *Body Mass Index (BMI)*: in kg/m^2^, mean (SD): 24.9 (3.8) *Blood pressure status*: Hypertensive, % (n/N): 31.6 (59/187) *Antihypertensive medication used*, % (n/N): 0 (0/187) *Cardiovascular disease or stroke*: NR *Diabetes mellitus*: NR *Renal impairment*: Serum creatinine > 177 µmol/L, % (n/N): 0 (0/187) *Dietary potassium intake*: NR *Dietary sodium intake*: NR *Urinary potassium excretion*: in mmol/L, median (IQR): 20.5 (13.2 to 31.5) *Urinary sodium excretion*: in mmol/L, median (IQR): 125.8 (81.1 to 151.2) Control (family members) *Age*: in years, mean (SD): 45.7 (17.4) *Gender*: Female, % (n/N): 47.4 (88/186) *Ethnicity/race*: Chinese *Smoking*: NR *Body Mass Index (BMI)*: in kg/m^2^, mean (SD): 25.2 (4.3) *Blood pressure status*: Hypertensive, % (n/N): 24.2 (45/186) *Antihypertensive medication used*, % (n/N): 0 (0/186) *Cardiovascular disease or stroke*: NR *Diabetes mellitus*: NR *Renal impairment*: Serum creatinine > 177 µmol/L, % (n/N): 0 (0/186) *Dietary potassium intake*: NR *Dietary sodium intake*: NR *Urinary potassium excretion*: in mmol/L, median (IQR): 24.1 (13.8 to 24.8) mmol/L *Urinary sodium excretion*: in mmol/L, median (IQR): 126.1 (96.7 to 152.6) **Inclusion criteria:** Households or families with men or women aged 18 years and older, with a SBP > 140 mm Hg or a DBP > 90 mm Hg at two measurements. Men and women with hypertension and their family members aged >= 18 years had to agree to replace at least half of their dietary salt with the LSSS intervention. **Exclusion criteria:** Households or families with contra‐indication to the use of a LSSS, such as the use of a potassium‐sparing medication or significant renal impairment (serum creatinine > 177 µmmol/L; eGFR for females aged 45 years: 29mL/min; eGFR for males aged 45 years: 39 mL/min). Households were excluded if they relocated during the study period. **Pretreatment:** None **Method of recruitment of participants:** Participants were referred from either general‐practitioner referrals or volunteers at two sites in Beijing. They were invited by letter or telephone for a re‐measurement of BP. **Informed consent obtained:** yes (written) **Clusters:** n = 220 households (1 participant with hypertension per household (n = 220 in total); mean no. of family members per household NR (n = 380 in total) **Subgroups planned/measured:** Age, gender and baseline BP **Subgroups reported:** Participants with hypertension: < 60 years vs. >= 60 years; male vs. female; stage 1 hypertension vs. stage 2 hypertension; family members: < 60 years vs. >= 60 years; male vs. female; participants with normal blood pressure vs. participants with hypertension *Participant flow* **Assessed for eligibility:** NR **Excluded (number with reasons):** NR **Randomised:** n = 220 **Allocated to LSSS intervention/s:** n = 110 households (n = 110 participants with hypertension; n = 191 family members) **Allocated to control:** n = 110 households (n = 110 participants with hypertension; n = 189 family members) **Received allocated LSSS intervention/s:** NR **Did not receive allocated LSSS intervention/s:** NR **Lost to follow‐up (LSSS intervention group):** n = 0 participants with hypertension, n = 17 family members **Discontinued intervention (LSSS intervention group):** n = 2 households (participants with hypertension: n = 2 disliked the salt); n = 5 family members withdrew due to loss of participants with hypertension **Analysed (LSSS intervention group):** 110 participants with hypertension; 187 family members. The study authors reported that all participants who had complete data and a valid home blood pressure measurement at baseline were included in an ITT analysis (baseline home BP values were used in the case of incomplete follow‐up data). **Excluded from analysis (LSSS intervention group):** n = 6 family members excluded due to invalid BP measurements **Received allocated control:** NR **Did not receive allocated control:** NR **Lost to follow‐up (control group):** n = 5 households (n = 5 participants with hypertension: n = 1 dislike the salt; n = 1 stroke; n = 1 appendicitis; n = 1 nephritis; n = 1 AMI; n = 14 family members: n = 5 withdrawal due to loss of participants with hypertension; n = 1 go to college; n = 2 marriage/divorce; n = 6 NR) **Discontinued intervention (control group):** n = 1 households (participants with hypertension: n = 1 disliked the salt; n = 5 family members withdrew due to loss of participants with hypertension) **Analysed (control group):** 110 participants with hypertension; 186 family members. The study authors reported that all participants who had complete data and a valid home blood pressure measurement at baseline were included in an ITT analysis (baseline home BP values were used in the case of incomplete follow‐up data). **Excluded from analysis (control group):** n = 6 family members excluded due to invalid BP measurements	
Interventions	**Intervention Characteristics** LSSS intervention *Theoretical basis*: 90% of salt in the Chinese diet come from home‐cooked foods. Therefore, the use of LSSS may result in lowering the home BP of individuals, irrespective of their starting BP measurements. *Description*: Salt substitute (65% NaCl, 25% KCl, 10% MgSO_4_) *LSSS category*: < 30% KCl *Contains fortificant*: NR *Delivery*: Discretionary use *Duration of run‐in period*: none *Duration of active intervention*: 12 months *Duration of follow‐up (as reported)*: none *Total duration of study (as reported)*: 12 months *Timing*: Used in all daily cooking, pickling, and other household uses *Implementation*: Identical 1 kg bags; frequency of delivery not reported *Providers*: Manufactured, packaged, and labelled by the Beijing Salt Industrial Company *Co‐interventions*: None. Participants strictly instructed to avoid any changes in dietary or lifestyle habits *Resource requirements*: Salt provided free of charge *Integrity of delivery*: Self‐reported compliance assessed at each follow‐up visit: proportion of household salt replaced by LSSS salt ('all'; 'nearly' all', 'half' 'less than half' or 'none') Control *Theoretical basis*: : To enable comparison of LSSS intervention with standard practice *Description*: Normal salt (100% NaCl) *Contains fortificant*: NR *Delivery*: Discretionary use *Duration of run‐in period*: none *Duration of active intervention*: 12 months *Duration of follow‐up (as reported)*: none *Total duration of study (as reported)*: 12 months *Timing*: Use in all daily cooking, pickling, and other household uses *Implementation*: Identical 1 kg bags; frequency of delivery not reported *Providers*: Manufactured, packaged, and labelled by the Beijing Salt Industrial Company *Co‐interventions*: None. Participants strictly instructed to avoid any changes in dietary or lifestyle habits *Resource requirements*: Salt provided free of charge *Integrity of delivery*: Self‐reported compliance assessed at each follow‐up visit: proportion of household salt replaced by control salt ('all'; 'nearly' all', 'half' 'less than half' or 'none')	
Outcomes	Primary outcomes: **Diastolic Blood Pressure (DBP):** Outcome measurement: Repeated self‐measurements of BP three times daily at home (early morning, noon, evening) on three consecutive days at each follow‐up point, with an automatic electronic device. Time points: 3, 6, 12 months **Systolic Blood Pressure (SBP):** Outcome measurement: Repeated self‐measurements of BP three times daily at home (early morning, noon, evening) on three consecutive days at each follow‐up point, with an automatic electronic device. Time points: 3, 6, 12 months **Hypertension (as reported, or SBP > 140 mmHg or DBP > 85 mmHg):** NR **Blood pressure control (achieving blood pressure threshold or 'control' as prespecified):** NR **Cardiovascular events‐stroke:** NR **Cardiovascular events‐myocardial infarction:** NR **Cardiovascular events‐other:** NR **Cardiovascular mortality:** NR **Blood potassium:** NR **Hyperkalaemia:** NR **Hypokalaemia:** NR Secondary outcomes: **All‐cause mortality:** NR **Adverse events:** fever; appendicitis; nephritis; nephrosis; outcome measurement: NR; time points: 3, 6, 12 months **Antihypertensive medication use:** Outcome measurement: number of participants on antihypertensive medication; time point: 3, 6, 12 months **Body Mass index (BMI):** NR **Serum creatinine:** NR **Albuminuria:** NR **Urinary albumin‐to‐creatinine ratio (uACR):** NR **Fasting blood glucose:** NR **Blood triglycerides:** NR **Total blood cholesterol:** NR **24‐h urinary sodium excretion:** NR **24‐h urinary potassium excretion:** NR	
Notes	**Funding source:** Danone Nutrition Fund, People’s Republic of China; Beijing Salt Industrial Company (LSSS and regular salt) **Authors name:** Jihong Hu; Yangfeng Wu **Institution:** Department of Epidemiology, Cardiovascular Institute and Fuwai Hospital, Chinese Academy of Medical Sciences and Peking Union Medical College and Public Health School, Gansu University of Chinese Medicine **Email:** hujihonghappy@163.com; wuyf@bjmu.edu.cn **Possible conflicts of interest (for study authors):** NR **Sources used for data extraction:** Journal article with results of the trial **Trial registration details:** The study authors cited NCT00145756 (China Salt Substitute Study). **Author correspondence:** Dr Wu (wuyf@bjmu.edu.cn) was contacted on 27/05/2020 to clarify whether Hu 2018 was part of the China Salt Substitute Study (CSSS). Received a reply from him on 28/05/2020 confirming that Hu 2018 was a different study from CSSS. Dr. Hu (hujihonghappy@163.com) was contacted on 10/07/2020 to request outcome data (mean changes in DBP, SBP). Received data from author on 17/07/2020.	
***Risk of bias***		
**Bias**	**Authors' judgement**	**Support for judgement**
Random sequence generation (selection bias)	Low risk	Computer random number generator used
Allocation concealment (selection bias)	Low risk	Interventions were packaged and labelled with randomised codes at central allocation. Sequentially numbered envelopes used for allocation
Blinding of participants and personnel (performance bias) All outcomes	Low risk	Interventions were packaged and labelled with randomised codes at central allocation. Sequentially numbered envelopes used for allocation
Blinding of outcome assessment (detection bias) All outcomes	Low risk	Participants (performing home‐based BP monitoring) and investigators (assessing compliance, adverse events) were blinded until the study base was unlocked.
Incomplete outcome data (attrition bias) All outcomes	High risk	Incomplete BP outcome data reported among participants with hypertension in both groups: 7.2% (8/110) in LSSS intervention group vs 3.6 % (4/110) in control group. However, there was differential attrition present among family members: 17.2 % (33/191) in LSSS intervention group vs. 9.5% (18/189) in control group.
Selective reporting (reporting bias)	Unclear risk	The study authors reported the trial registration number of another trial (China Salt Substitute study; NCT00145756). Therefore, study protocol not available
Other bias	Low risk	None identified
Recruitment bias (cluster‐RCTs)	Low risk	Individuals recruited before randomisation of clusters
Comparability with individually randomised trials (cluster‐RCTs)	Low risk	A previous RCT has investigated home BP response to a salt substitute, finding a significant reduction in home SBP among hypertensive patients (Hu 2014).
Loss of clusters (cluster‐RCTs)	Low risk	Attrition of households was low (7.2% (8/110) in LSSS intervention group vs 4.5 % (5/110) in control group). ITT analysis performed
Baseline imbalance (cluster‐RCTs)	Low risk	The study reported no significant differences in gender, age, BMI, home SBP and DBP, hypertension rate and salt consumption of participants with hypertension and family members. There was a difference in the proportion of family member who were hypertensive (31.6% in the intervention group vs. 24.2% in the control group).
Incorrect analysis (cluster‐RCTs)	High risk	Adjustment to account for the effect of clustering was not reported in the statistical analysis or results section for family members and combined data with participants who have hypertension.
Overall risk of bias	High risk	Low risk of bias for baseline imbalance, recruitment bias and loss of clusters; high risk of bias for incomplete outcome data

**Table:** Kawasaki 1998

***Study characteristics***		
Methods	**Study design:** Randomised controlled trial **Study grouping:** Parallel group **Country:** Japan **Setting:** Intervention conducted in households; setting where outcomes were measured not reported **Aim of study:** To explore the antihypertensive effects of mineral salt by performing a trial of mild dietary sodium reduction with mild potassium and magnesium supplementation in normotensive and hypertensive middle‐aged and elderly subjects **Unit of allocation:** adults (aged 18 years or older) **Start date:** NR **End date:** NR **Relevant study limitations as reported by study authors:** NR **Sample size calculation:** NR	
Participants	**Baseline Characteristics** LSSS intervention *Age*: in years, mean (SD): 65.9 (7.4) *Gender*: Female, % (n/N): 47.6 (11/21) *Ethnicity/race*: Japanese *Smoking*: NR *Body Mass Index (BMI)*: mean (SD): 22.9 (2.7) *Blood pressure status*: Normotensive, % (n/N): 52.4 (11/21); hypertensive, % (n/N): 47.6 (10/21) *Antihypertensive medication used*: Beta‐blockers, calcium channel blockers or both, n/N: 19.0 (4/21) *Cardiovascular disease or stroke*: NR *Diabetes mellitus*: NR *Renal impairment*: NR *Dietary potassium intake*: NR *Dietary sodium intake*: NR *Urinary potassium excretion*: in mmol/day, mean (SD): 51.5 (12.6) *Urinary sodium excretion*: in mmol/day, mean (SD): 192 (61) Control *Age*: in years, mean (SD): 65.8 (7.6) *Gender*: Female, % (n/N): 50 (10/20) *Ethnicity/race*: Japanese *Smoking*: NR *Body Mass Index (BMI)*: mean (SD): 23.3 (2.3) *Blood pressure status*: Normotensive, % (n/N): 60.0 (12/20); hypertensive, % (n/N): 40.0 (8/20) *Antihypertensive medication used*: Beta‐blockers, calcium channel blockers or both, % (n/N): 20.0 (4/20) *Cardiovascular disease or stroke*: NR *Diabetes mellitus*: NR *Renal impairment*: NR *Dietary potassium intake*: NR *Dietary sodium intake*: NR *Urinary potassium excretion*: in mmol/day, mean (SD): 56.9 (17.3) *Urinary sodium excretion*: in mmol/day, mean (SD): 171 (47) LSSS intervention (participants with normal blood pressure) *Age*: NR *Gender*: NR *Ethnicity/race*: Japanese *Smoking*: NR *Body Mass Index (BMI)*: NR *Blood pressure status*: n/a *Antihypertensive medication used*: NR *Cardiovascular disease or stroke*: NR *Diabetes mellitus*: NR *Renal impairment*: NR *Dietary potassium intake*: NR *Dietary sodium intake*: NR *Urinary potassium excretion*: in mmol/day, mean (SD): 51.7 (14.3) *Urinary sodium excretion*: in mmol/day, mean (SD): 174 (63) Control (participants with normal blood pressure) *Age*: NR *Gender*: NR *Ethnicity/race*: Japanese *Smoking*: NR *Body Mass Index (BMI)*: NR *Blood pressure status*: n/a *Antihypertensive medication used*: NR *Cardiovascular disease or stroke*: NR *Diabetes mellitus*: NR *Renal impairment*: NR *Dietary potassium intake*: NR *Dietary sodium intake*: NR *Urinary potassium excretion*: in mmol/day, mean (SD): 53.8 (15.5) *Urinary sodium excretion*: in mmol/day, mean (SD):174 (57) LSSS intervention (participants with hypertension) *Age*: NR *Gender*: NR *Ethnicity/race*: Japanese *Smoking*: NR *Body Mass Index (BMI)*: NR *Blood pressure status*: n/a *Antihypertensive medication used*: NR *Cardiovascular disease or stroke*: NR *Diabetes mellitus*: NR *Renal impairment*: NR *Dietary potassium intake*: NR *Dietary sodium intake*: NR *Urinary potassium excretion*: in mmol/day, mean (SD): 52.3 (10.7) *Urinary sodium excretion*: in mmol/day, mean (SD): 211 (55) Control (participants with hypertension) *Age*: NR *Gender*: NR *Ethnicity/race*: Japanese *Smoking*: NR *Body Mass Index (BMI)*: NR *Blood pressure status*: n/a *Antihypertensive medication used*: NR *Cardiovascular disease or stroke*: NR *Diabetes mellitus*: NR *Renal impairment*: NR *Dietary potassium intake*: NR *Dietary sodium intake*: NR *Urinary potassium excretion*: in mmol/day, mean (SD): 55.0 (21.4) *Urinary sodium excretion*: in mmol/day, mean (SD): 174 (50) **Inclusion criteria:** Clinically healthy middle‐aged and elderly volunteers with ordinary lifestyles **Exclusion criteria:** Participants who planned to travel during study period, or frequently dined away from home, or had a high risk of noncompliance with study protocol **Pretreatment:** No significant differences between groups at baseline **Method of recruitment of participants:** Questionnaire **Informed consent obtained:** Yes (written) **Clusters:** n/a **Subgroups planned/measured:** NR **Subgroups reported:** participants with normal blood pressure; participants with hypertension *Participant flow* **Assessed for eligibility:** "More than 50" **Excluded (number with reasons):** NR **Randomised:** n = 41 **Allocated to LSSS intervention(s):** n = 21 **Allocated to control:** n = 20 **Received allocated LSSS intervention(s):** NR **Did not receive allocated LSSS intervention(s):** NR **Lost to follow‐up (LSSS intervention group):** n = 0 **Discontinued intervention (LSSS intervention group):** n = 0 **Analysed (LSSS intervention group):** n = 21 **Excluded from analysis (LSSS intervention group):** n = 0 **Received allocated control:** NR **Did not receive allocated control:** NR **Lost to follow‐up (control group):** n = 0 **Discontinued intervention (control group):** n = 0 **Analysed (control group):** n = 20 **Excluded from analysis (control group):** n = 0	
Interventions	**Intervention Characteristics** LSSS intervention *Theoretical basis*: A LSSS may aid in the prevention and management of hypertension by lowering the intake of dietary sodium from a traditional Japanese diet. *Description*: 'Reduced sodium mineral' salt (22.9 % Na; 10.1 % K; 1.2% Mg per g) used instead of regular salt; also used in the preparation of soy sauce (4.3% Na; 1.2% K; 0.1% Mg) and miso (2.9 % Na; 1.6% K; 0.2% Mg) *LSSS category*: < 30% KCl *Contains fortificant*: NR *Delivery*: mixed (discretionary and non‐discretionary use) *Duration of run‐in period*: 2 weeks *Duration of active intervention*: 5 weeks *Duration of follow‐up (as reported)*: none *Total duration of study (as reported)*: 5 weeks *Timing*: Daily use by household; refrain from eating way from home. If they had to dine out, they were instructed to take lunch boxes prepared with seasonings from home. *Implementation*: NR *Providers*: Seasonings (miso, soy sauce) prepared in research laboratory with LSSS salt. Details of persons providing the LSSS interventions not reported *Co‐interventions*: None. Participants instructed not to alter their lifestyle *Resource requirements*: NR *Integrity of delivery*: 'Regular' telephonic contact with participants; "good compliance was maintained throughout the study". Control *Theoretical basis*: To enable comparison of LSSS intervention with standard practice *Description*: Regular'' salt (39% Na; 0.1% K, 0.02 % Mg) used as salt; regular soy sauce (5.4% Na; 0.3% K; 0.07% Mg) and miso (4.3% Na; 0.2% K; 0.05% Mg) *Contains fortificant*: NR *Delivery*: mixed (discretionary and non‐discretionary use) *Duration of run‐in period*: 2 weeks *Duration of active intervention*: 5 weeks *Duration of follow‐up (as reported)*: none *Total duration of study (as reported)*: 5 weeks *Timing*: Daily use by each household. Participants to refrain from eating away from home. *Implementation*: NR *Providers*: NR *Co‐interventions*: None. Participants instructed not to alter their lifestyle *Resource requirements*: NR *Integrity of delivery*: 'Regular' telephonic contact with participants	
Outcomes	Primary outcomes: **Diastolic Blood Pressure (DBP):** Outcome measurement: At baseline three consecutive measurements were taken with an automatic sphygmomanometer every 2 mins (after urination and 10 mins rest in sitting position) during early morning. The 3 measurements were averaged. Follow‐up measurements were taken after 30 mins rest in the supine position. Time points: 2 and 5 weeks **Systolic Blood Pressure (SBP):** Outcome measurement: At baseline three consecutive measurements were taken with an automatic sphygmomanometer every 2 mins (after urination and 10 mins rest in sitting position) during early morning. The 3 measurements were averaged. Follow‐up measurements were taken after 30 mins rest in the supine position. Time points: 2 and 5 weeks **Hypertension (as reported, or SBP > 140 mmHg or DBP > 85 mmHg):** NR **Blood pressure control (achieving blood pressure threshold or 'control' as prespecified):** NR **Cardiovascular events‐stroke:** NR **Cardiovascular events‐myocardial infarction:** NR **Cardiovascular events‐other:** NR **Cardiovascular mortality:** NR **Blood potassium:** Outcome measurement: 12‐hour fasting blood samples collected and measurement of serum concentrations by flame photometry; time points: 5 weeks **Hyperkalaemia:** NR **Hypokalaemia:** NR Secondary outcomes: **All‐cause mortality:** NR **Adverse events:** NR **Antihypertensive medication use:** NR **Body Mass index (BMI):** NR **Serum creatinine:** NR **Albuminuria:** NR **Urinary albumin‐to‐creatinine ratio (uACR):** NR **Fasting blood glucose:** NR **Blood triglycerides:** Outcome measurement: 12‐hour fasting blood samples collected and analysed by autoanalysers. Time points: 5 weeks **Total blood cholesterol:** Outcome measurement: 12‐hour fasting blood samples collected and analysed by autoanalysers. Time points: 5 weeks **24‐h urinary sodium excretion:** 24‐hour urine sample. Time points: 5 weeks **24‐h urinary potassium excretion:** 24‐hour urine sample. Time points: 5 weeks	
Notes	**Funding source:** The LSSS, described as 'mineral' salt (Pansalt), was supplied by Time Associates Co. Ltd, Tokyo, Japan. **Authors name:** Terukazu Kawasaki **Institution:** Institute of Health Science, Kyushu University **Email:** itohK@k7.dion.ne.jp **Possible conflicts of interest (for study authors):** NR **Sources used for data extraction:** Journal article(s) with results of the trial **Trial registration details:** NR **Author correspondence:** Dr. Itoh (co‐author) was contacted on 26/06/2020 to obtain relevant outcome data for control group (SBP, DBP). Received no reply.	
***Risk of bias***		
**Bias**	**Authors' judgement**	**Support for judgement**
Random sequence generation (selection bias)	Unclear risk	Insufficient information available
Allocation concealment (selection bias)	Unclear risk	Not reported
Blinding of participants and personnel (performance bias) All outcomes	Unclear risk	Taste tests showed no differences between LSSS and control seasonings for savouriness or palatability. It was not clear whether control participants received study seasonings, or whether they used their own.
Blinding of outcome assessment (detection bias) All outcomes	Low risk	Blinding not reported. Objective study outcomes: blood pressure (using a standardised procedure and the use of an automatic sphygmanometer) and biochemical assays of blood and urine samples
Incomplete outcome data (attrition bias) All outcomes	Low risk	No missing data
Selective reporting (reporting bias)	Unclear risk	No study protocol or prospective trial registry entry available
Other bias	Low risk	None identified
Overall risk of bias	Unclear risk	Unclear risk of bias for allocation concealment; low risk of bias for incomplete outcome data

**Table:** Li 2014

***Study characteristics***		
Methods	**Study design:** Cluster‐randomised controlled trial **Country:** China **Setting:** Intervention conducted in local residents' family; outcomes measured at their homes **Aim of study:** To explore the effects of low‐sodium salt on blood pressure and to find out an economical, effective and easy‐to‐implement method to reduce the blood pressure **Unit of allocation:** households **Start date:** NR **End date:** NR **Relevant study limitations as reported by study authors:** NR **Sample size calculation:** NR	
Participants	**Baseline Characteristics** LSSS intervention *Age*: in years, mean (SD): 59.3 (11.7) *Gender*: Female, % (n/N): 50.9 (129/253) *Ethnicity/race*: Chinese *Smoking*: NR *Body Mass Index (BMI)*: NR *Blood pressure status*: DBP at baseline, mean (SD): 82.0 (10.1); SBP at baseline, mean (SD): 128.9 (13.5) *Antihypertensive medication used*: NR *Cardiovascular disease or stroke*: NR *Diabetes mellitus*: NR *Renal impairment*: Serious kidney diseases, % (n/N): 0 (0/253) *Dietary potassium intake*: NR *Dietary sodium intake*: NR *Urinary potassium excretion*: NR *Urinary sodium excretion*: NR Control *Age*: in years, mean (SD): 59.2 (8.7) *Gender*: Female, % (n/N): 48.7 (128/263) *Ethnicity/race*: Chinese *Smoking*: NR *Body Mass Index (BMI)*: NR *Blood pressure status*: DBP at baseline, mean (SD): 81.3 (11.7); SBP at baseline, mean (SD): 126.8 (13.5) *Antihypertensive medication used*: NR *Cardiovascular disease or stroke*: NR *Diabetes mellitus*: NR *Renal impairment*: Serious kidney diseases, % (n/N): 0 (0/263) *Dietary potassium intake*: NR *Dietary sodium intake*: NR *Urinary potassium excretion*: NR *Urinary sodium excretion*: NR **Inclusion criteria:** At least 2 members in each family meet the following conditions: (1) Family members ≥ 50 years old. (2) Eating at home every week > 5 d.(3) Can participate in the whole project, without long‐term arrangement **Exclusion criteria:** Exclude serious kidney diseases (not defined by study authors) and potassium‐preserving diuretic treatment **Pretreatment:** Baseline gender, age, SBP and DBP were not significantly different between the intervention group and the control group. In addition, other factors such as television, newspapers and other important information sources, as well as some common food sources of salt were similar between the groups. **Method of recruitment of participants:** Quote: "66 journalists working for a local newspaper were responsible for recommending participating families and family members. Each reporter was responsible for recommending four families." **Informed consent obtained:** NR **Clusters:** n = 66 clusters (n = 253 participants in LLLS group, n = 63 participants in control group) **Subgroups planned/measured:** NR **Subgroups reported:** male vs. female; age (50‐59 years vs. 60‐69 years vs. > 70 years) *Participant flow* **Assessed for eligibility:** 78 journalists **Excluded (number with reasons):** 12 journalists (declined to participate) **Randomised:** n = 264 families recommended by 66 journalists (n = 528 participants) **Allocated to LSSS intervention(s):** n = 258 participants; n = 129 (clusters) **Allocated to control:** n = 270 participants; n = 135 (clusters) **Received allocated LSSS intervention(s):** All participants **Did not receive allocated LSSS intervention(s):** n = 0 **Lost to follow‐up (LSSS intervention group):** n = 3 (n = 1 hospitalisation; n = 2 ‘went out’) **Discontinued intervention (LSSS intervention group):** n = 2 **Analysed (LSSS intervention group):** n = 253 participants from 129 clusters **Excluded from analysis (LSSS intervention group):** n = 0 **Received allocated control:** All participants **Did not receive allocated control:** n = 0 **Lost to follow‐up (control group):** n = 3 (n = 3 ‘went out’) **Discontinued intervention (control group):** n = 4 **Analysed (control group):** n = 263 participants from 133 clusters **Excluded from analysis (control group):** n = 0	
Interventions	**Intervention Characteristics** LSSS intervention *Theoretical basis*: *Description*: Low‐sodium salt (NaCl (70.0 ± 10.0)g/100.0 g, and KCl (30.0 ± 10.0)g/100.0 g) *LSSS category*: ≥ 30% KCl *Contains fortificant*: NR *Delivery*: discretionary use *Duration of run‐in period*: none *Duration of active intervention*: 2 months *Duration of follow‐up (as reported)*: none *Total duration of study (as reported)*: 2 months *Timing*: NR *Implementation*: 350g bags *Providers*: LSSS produced by Shanxi Salt Co., Ltd. and produced by Jiangsu Ruifeng Salt Co., Ltd *Co‐interventions*: NR *Resource requirements*: NR *Integrity of delivery*: NR Control *Theoretical basis*: To enable comparison of LSSS intervention with standard practice *Description*: regular salt *Contains fortificant*: NR *Delivery*: discretionary use *Duration of run‐in period*: none *Duration of active intervention*: 2 months *Duration of follow‐up (as reported)*: none *Total duration of study (as reported)*: 2 months *Timing*: NR *Implementation*: Own household supply *Providers*: NR *Co‐interventions*: NR *Resource requirements*: Automatic BP devices (Omron HEM‐7200 *Integrity of delivery*: NR	
Outcomes	Primary outcomes: **Diastolic Blood Pressure (DBP):** Outcome measurement: Automatic device calibrated by the manufacturer before using. Time points: measured before and 2 months after the intervention. **Systolic Blood Pressure (SBP):** Outcome measurement: Automatic device calibrated by the manufacturer before using. Time points: measured before and 2 months after the intervention. **Hypertension (as reported, or SBP > 140 mmHg or DBP > 85 mmHg):** NR **Blood pressure control (achieving blood pressure threshold or 'control' as prespecified):** NR **Cardiovascular events‐stroke:** NR **Cardiovascular events‐myocardial infarction:** NR **Cardiovascular events‐other:** NR **Cardiovascular mortality:** NR **Blood potassium:** NR **Hyperkalaemia:** NR **Hypokalaemia:** NR Secondary outcomes: **All‐cause mortality:** NR **Adverse events:** NR **Antihypertensive medication use:** NR **Body Mass index (BMI):** NR **Serum creatinine:** NR **Albuminuria:** NR **Urinary albumin‐to‐creatinine ratio (uACR):** NR **Fasting blood glucose:** NR **Blood triglycerides:** NR **Total blood cholesterol:** NR **24‐h urinary sodium excretion:** NR **24‐h urinary potassium excretion:** NR	
Notes	**Funding source:** China International Center for Chronic Disease Prevention **Authors name:** Xiang‐Xian, Feng **Institution:** Department of Preventive Medicine, Changzhi Medical College **Email:** xfeng66@163.com **Possible conflicts of interest (for study authors):** NR **Sources used for data extraction:** Journal article with results of the trial **Trial registration details:** NCT03074851	
***Risk of bias***		
**Bias**	**Authors' judgement**	**Support for judgement**
Random sequence generation (selection bias)	Low risk	Use of random number table
Allocation concealment (selection bias)	Unclear risk	Insufficient information available
Blinding of participants and personnel (performance bias) All outcomes	Unclear risk	Participants not blinded; BP not likely to be influenced by lack of blinding
Blinding of outcome assessment (detection bias) All outcomes	Low risk	Outcome assessors not blinded, but SBP and DBP measurements conducted with an automatic device
Incomplete outcome data (attrition bias) All outcomes	Low risk	Total attrition was minimal.
Selective reporting (reporting bias)	Unclear risk	Clinical trial registry entry available, but not prospectively registered
Other bias	Low risk	None identified
Recruitment bias (cluster‐RCTs)	Low risk	Journalists recruited all participants before randomisation.
Comparability with individually randomised trials (cluster‐RCTs)	Low risk	Comparability of the effect estimates of RCTs in the same comparison
Loss of clusters (cluster‐RCTs)	Low risk	Loss of two clusters in the control arm; however minimal effect on attrition
Baseline imbalance (cluster‐RCTs)	Low risk	No baseline imbalances reported between intervention and control participants
Incorrect analysis (cluster‐RCTs)	High risk	No adjustment for clustering in data analysis
Overall risk of bias	Low risk	Low risk of bias for incomplete outcome data, baseline imbalance, recruitment bias and loss of clusters

**Table:** Li 2016

***Study characteristics***		
Methods	**Study design:** Cluster‐randomised controlled trial **Country:** China **Setting:** Intervention conducted in townships/villages from five northern provinces Hebei, Liaoning, Ningxia, Shanxi and Shaanxi; outcomes measured at village level **Aim of study:** To define the effects of a community‐based sodium reduction strategy on the average sodium intake **Unit of allocation:** Villages **Start date:** May 2011 **End date:** November 2012 **Relevant study limitations as reported by study authors:** Lack of baseline survey data resulted in less statistical power to reliably detect any effect on blood pressure of the observed changes in sodium consumption; did not systematically collect tolerability data **Sample size calculation:** To detect a difference in mean 24‐hour excretion of sodium of at least 11 mmol/day (0.65g/day salt) between intervention and control clusters with 90% power (with a two‐sided alpha = 0.05), assuming a standard deviation of 24‐hour sodium excretion of 60 mmol/day, an ICC of 0.05, and a sample of 2400 individuals drawn from 120 clusters randomised equally between intervention and control groups	
Participants	**Baseline Characteristics** LSSS intervention (with price subsidy) *Age*: NR *Gender*: NR *Ethnicity/race*: NR *Smoking*: NR *Body Mass Index (BMI)*: NR *Blood pressure status*: NR *Antihypertensive medication used*: NR *Cardiovascular disease or stroke*: NR *Diabetes mellitus*: NR *Renal impairment*: NR *Dietary potassium intake*: NR *Dietary sodium intake*: NR *Urinary potassium excretion*: NR *Urinary sodium excretion*: NR LSSS intervention (without price subsidy) *Age*: NR *Gender*: NR *Ethnicity/race*: NR *Smoking*: NR *Body Mass Index (BMI)*: NR *Blood pressure status*: NR *Antihypertensive medication used*: NR *Cardiovascular disease or stroke*: NR *Diabetes mellitus*: NR *Renal impairment*: NR *Dietary potassium intake*: NR *Dietary sodium intake*: NR *Urinary potassium excretion*: NR *Urinary sodium excretion*: NR LSSS intervention *Age*: NR *Gender*: NR *Ethnicity/race*: NR *Smoking*: NR *Body Mass Index (BMI)*: NR *Blood pressure status*: NR *Antihypertensive medication used*: NR *Cardiovascular disease or stroke*: NR *Diabetes mellitus*: NR *Renal impairment*: NR *Dietary potassium intake*: NR *Dietary sodium intake*: NR *Urinary potassium excretion*: NR *Urinary sodium excretion*: NR Control *Age*: NR *Gender*: NR *Ethnicity/race*: NR *Smoking*: NR *Body Mass Index (BMI)*: NR *Blood pressure status*: NR *Antihypertensive medication used*: NR *Cardiovascular disease or stroke*: NR *Diabetes mellitus*: NR *Renal impairment*: NR *Dietary potassium intake*: NR *Dietary sodium intake*: NR *Urinary potassium excretion*: NR *Urinary sodium excretion*: NR **Inclusion criteria:** Villages within each township (total of 120 included townships) from that were closest to the geographic centre of the township. If the village declined to participate, the next most geographically central village was asked to participate. **Exclusion criteria:** Villages where the township healthcare centre was situated **Pretreatment:** The study presenting the main results of the trial stated the following: "Baseline survey data were not available because of resource constraints." During the end‐line survey, no group differences were reported in terms of age, gender, education, smoking and drinking history and BMI (Li 2016). **Method of recruitment of participants:** Two counties from each province were selected on the basis of their interest in and willingness to participate, their proximity to the local research team, and their representativeness of the socioeconomic development level of counties in each province. In each county, 12 townships were selected primarily through discussion with the leadership of the local county Bureau of Health to identify willing and accessible partners. Finally, within each township one village was selected, typically the village closest to the geographic centre of the township. **Informed consent obtained:** yes (verbal consent from village chiefs; written consent from participants) **Clusters:** n = 120 villages (n = 60 intervention villages [n = 30 with price subsidy; n = 30 without price subsidy]; n = 60 control villages); average population size of villages: 1867 individuals living in 512 households **Subgroups planned/measured:** NR **Subgroups reported:** NR *Participant flow* **Assessed for eligibility:** 10 to 20 villages per township (n = 12) **Excluded (number with reasons):** NR **Randomised:** n = 120 villages **Allocated to LSSS intervention(s):** n = 60 villages [n = 30 villages and n = 1268 participants (LSSS intervention with price subsidy) and n = 30 villages and n = 1253 participants (LSSS intervention without price subsidy)] **Allocated to control:** n = 60 villages **Received allocated LSSS intervention(s):** NR **Did not receive allocated LSSS intervention(s):** NR **Lost to follow‐up (LSSS intervention group):** n = 0 **Discontinued intervention (LSSS intervention group):** n = 0 **Analysed (LSSS intervention group):** SBP, DBP outcomes: n = 650 individuals (LSSS intervention with price subsidy); n = 644 (LSSS intervention without price subsidy); urinary creatinine ratio; microalbuminuria outcomes: n = 528 individuals (LSSS intervention with price subsidy); n = 447 (LSSS intervention without price subsidy) **Excluded from analysis (LSSS intervention group):** n = 122 individuals (LSSS intervention with price subsidy); n = 197 individuals (LSSS intervention without price subsidy) (ineligible urine samples) **Received allocated control:** NR **Did not receive allocated control:** NR **Lost to follow‐up (control group):** n = 1 village **Discontinued intervention (control group):** n = 1 village (due to urbanisation of the village and relocation of participants) **Analysed (control group):** SBP, DBP outcomes: n = 1272 individuals; urinary creatinine ratio; microalbuminuria outcomes: n = 928 individuals **Excluded from analysis (control group):** n = 344 individuals (ineligible urine samples)	
Interventions	**Intervention Characteristics** LSSS intervention (with price subsidy) *Theoretical basis*: A community salt reduction programme, incorporating the use of a LSSS, may decrease the sodium intake of rural village inhabitants, who typically consume high sodium intakes. The use of a price subsidy will encourage village residents to purchase LSSS. *Description*: Reduced‐sodium, added‐potassium salt substitute (NaCl: 70.00% ± 10.00%, KCl: 20.00% to 35.00%) *LSSS category*: < 30% KCl *Contains fortificant*: Iodine *Delivery*: Discretionary use *Duration of run‐in period*: none *Duration of active intervention*: 18 months *Duration of follow‐up (as reported)*: none *Total duration of study (as reported)*: 18 months *Timing*: Daily household use of LSSS in cooking and food pickling practices *Implementation*: Food supply: LSSS available for purchase at village shops at a subsidised price (same as regular salt). Community‐based health education programme: implemented via public announcement systems, bulletin boards, and specially developed promotional materials *Providers*: Study staff working with the existing township health educators, the village council and village doctors *Co‐interventions*: Half of the LSSS intervention villages were included in a concurrent cluster‐RCT providing primary‐care based high cardiovascular risk management package delivered by village doctors (Yan 2014) *Resource requirements*: NR *Integrity of delivery*: NR *Compliance*: LSSS intervention (without price subsidy) *Theoretical basis*: A community salt reduction programme, incorporating the use of a LSSS, may decrease the sodium intake of village inhabitants, who typically consume high sodium intakes. The higher price of LSSS may result in village residents not purchasing LSSS. *Description*: Reduced‐sodium, added‐potassium salt substitute (NaCl: 70.00% ± 10.00%, KCl: 20.00% to 35.00%); iodised *LSSS category*: < 50% KCl *Contains fortificant*: Iodine *Delivery*: Discretionary use *Duration of run‐in period*: none *Duration of active intervention*: 18 months *Duration of follow‐up (as reported)*: none *Total duration of study (as reported)*: 18 months *Timing*: Daily household use of LSSS in cooking and food pickling practices *Implementation*: Food supply: LSSS available for purchase at village shops at a standard price (approximately double the price of regular salt). Community‐based health education programme: implemented via public announcement systems, bulletin boards, and specially developed promotional materials *Providers*: Study staff working with the existing township health educators, the village council and village doctors *Co‐interventions*: Half of the LSSS intervention villages were included in a concurrent cluster‐RCT providing primary‐care based high cardiovascular risk management package delivered by village doctors (Yan 2014) *Resource requirements*: NR *Integrity of delivery*: NR Control *Theoretical basis*: To enable comparison of LSSS intervention with standard practice *Description*: Regular salt *Contains fortificant*: NR *Delivery*: Discretionary use *Duration of run‐in period*: none *Duration of active intervention*: 18 months *Duration of follow‐up (as reported)*: none *Total duration of study (as reported)*: 18 months *Timing*: Daily household use of salt in cooking and food pickling practices *Implementation*: No sodium reduction interventions in villages *Providers*: n/a *Co‐interventions*: Half of the control villages were included in a concurrent cluster‐RCT providing primary‐care based high cardiovascular risk management package delivered by village doctors (Yan 2014) *Resource requirements*: NR *Integrity of delivery*: NR	
Outcomes	Primary outcomes: **Diastolic Blood Pressure (DBP):** Outcome measurement: Measured in duplicate with the participant seated after 5 minutes rest, using an automated electronic sphygmomanometer with measurements made at least two minutes apart; time points: 18 months **Systolic Blood Pressure (SBP):** Outcome measurement: Measured in duplicate with the participant seated after 5 minutes rest, using an automated electronic sphygmomanometer with measurements made at least two minutes apart; time points: 18 months **Hypertension (as reported, or SBP > 140 mmHg or DBP > 85 mmHg):** Outcome measurement: defined as a measured SBP >= 140 mmHg, DBP >= 90 mmHg, or the use of blood‐pressure lowering therapy in the last two weeks; time points: 18 months **Blood pressure control (achieving blood pressure threshold or 'control' as prespecified):** NR **Cardiovascular events‐stroke:** NR **Cardiovascular events‐myocardial infarction:** NR **Cardiovascular events‐other:** NR **Cardiovascular mortality:** NR **Blood potassium:** NR **Hyperkalaemia:** NR **Hypokalaemia:** NR Secondary outcomes: **All‐cause mortality:** NR **Adverse events:** NR **Antihypertensive medication use:** Outcome measurement: self‐reported use by participants; time points: 18 months **Body Mass index (BMI):** Outcome measurement: NR; time points: 18 months **Serum creatinine:** NR **Albuminuria:** Outcome measurement: any albuminuria, microalbuminuria (defined as uACR of 22.1‐220.35 mg/g for men and 31.0–308.85 mg/g for women), or macroalbuminuria (defined as uACR of >= 220.35 mg/g for men and >= 309.73 mg/g for women); time points: 18 months **Urinary albumin‐to‐creatinine ratio (uACR):** Outcome measurement: NR; time points: 18 months **Fasting blood glucose:** NR **Blood triglycerides:** NR **Total blood cholesterol:** NR **24‐h urinary sodium excretion:** 24‐hour urine sample. Time points: 18 months **24‐h urinary potassium excretion:** 24‐hour urine sample. Time points: 18 months	
Notes	**Funding source:** The National Heart, Lung, and Blood Institute, National Institutes of Health, Department of Health and Human Services, and National Center for Chronic Disease Prevention and Health Promotion (CDC). Additional support from the United Health Group Chronic Disease Initiative. Study authors supported by fellowships from an Australian Research Council; National Health and Medical Research Council; MRC‐PHE Centre for Environment and Health, Imperial College London, and by the National Institute for Health Research (NIHR) Biomedical Research Centre at Imperial College Healthcare NHS Trust and Imperial College London **The China Rural Health Initiative Sodium Reduction Study (CHRS‐SRS)** **Authors name:** Nicole Li; Lijing Yan; Yangfeng Wu **Institution:** The George Institute for Global Health at Peking University Health Science Center, China; the George Institute for Global Health, Sydney, Australia; Sydney Medical School, the University of Sydney, Australia **Email:** ywu@georgeinstitute.org.cn; lyan@georgeinstitute.org.cn (LY) **Possible conflicts of interest (for study authors):** "Bruce Neal is the Chair of Australian Division of World Action on Salt and Health." **Sources used for data extraction:** Journal articles with results of the trial; trial protocol (published); conference abstracts **Trial registration details:** NCT01259700 (China Rural Health Initiative: High cardiovascular risk management and salt reduction in rural villages in China ‐ two parallel large cluster‐randomised controlled trials) **Author correspondence:** Drs. Yan and Wu were contacted to clarify the baseline survey characteristics, as reported by Li 2013. Received no reply	
***Risk of bias***		
**Bias**	**Authors' judgement**	**Support for judgement**
Random sequence generation (selection bias)	Low risk	Computerised sequence generation
Allocation concealment (selection bias)	Low risk	Central computerised randomisation, stratified by country to intervention or control group. LSSS intervention villages further assigned at random to receive subsidisation of the price of LSSS
Blinding of participants and personnel (performance bias) All outcomes	Unclear risk	Not possible to blind participants or personnel, such as study staff, township health educators, the village council and village doctors. Outcomes were objective measures unlikely to be prone to performance bias.
Blinding of outcome assessment (detection bias) All outcomes	Unclear risk	Endline survey was carried out by a study team independent of those involved in the intervention implementation, including village doctors, local project officers, intervention trainers and officials in the county Bureau of Health. The study authors stated: "Since blinding of data collection to the randomized allocation of the villages may be difficult to maintain, there will be a strong focus on training staff to apply rigorously standardized evaluation techniques for every outcome assessed ". Some objective study outcomes, such as BP measurements, were measured using automated devices.
Incomplete outcome data (attrition bias) All outcomes	Unclear risk	We assessed the incompleteness of outcome data collected from participants who participated in the endline survey (with no baseline data). Complete data was collected for antihypertensive medication use; minimal incomplete data for SBP (n = 1 in LSSS with price subsidy intervention group) and DBP (n = 1 in LSSS without price subsidy group). However, incomplete urinary outcome data (urinary creatinine:albumin ratio; albuminuria): 19% in the LSSS with price subsidy intervention group; 31% in the LSSS without price subsidy intervention group, and 27% in the control group. In a sensitivity analysis of the urinary outcomes, the study authors included data from all urine samples and reported the effect estimates as unchanged (data not shown) (see Jardine 2019).
Selective reporting (reporting bias)	High risk	The primary outcomes cited in the trial registry protocol for the overall study differed from those reported by the published study protocol (see Li 2013). For example, SBP is listed as a primary outcome in the trial registry protocol, but only as a secondary outcome in the published study protocol. In addition, a substudy (see Jardine 2019) stated that their primary outcome (urinary creatinine‐albumin ratio) was prespecified; however this outcome was not described in the trial register study protocol.
Other bias	Low risk	None identified
Recruitment bias (cluster‐RCTs)	Low risk	Individual participants from all the selected clusters (villages) participated in a baseline survey before any interventions were implemented.
Comparability with individually randomised trials (cluster‐RCTs)	Unclear risk	"Eight RCTs of LSSS interventions report reductions or trends towards lowering sodium excretion and SBP, however these trials achieved much larger reductions in sodium excretion (and thus intake of sodium), compared to the present study."
Loss of clusters (cluster‐RCTs)	Low risk	One cluster in the control group was lost to follow‐up (due to relocation of all village inhabitants). No clusters were lost to follow‐up in the intervention group.
Baseline imbalance (cluster‐RCTs)	Unclear risk	The paper with the main results of the trial (Li 2016) stated the following: "Baseline survey data were not available because of resource constraints." Therefore, we have not evaluated the baseline data presented in the study protocol publication (see Li 2013).
Incorrect analysis (cluster‐RCTs)	Low risk	All statistical analyses were adjusted for the effects of clustering.
Overall risk of bias	Unclear risk	Unclear risk of bias for incomplete outcome data and baseline imbalance, low risk of bias for loss of clusters and recruitment bias

**Table:** Mu 2003

***Study characteristics***		
Methods	**Study design:** Randomised controlled trial **Study grouping:** Parallel group **Country:** China **Setting:** Intervention conducted in households; outcomes measured at research site, Hanzhong city **Aim of study:** To observe the effect of long‐term supplementation of potassium and calcium on the blood pressure and sodium metabolism of adolescents by adding potassium and calcium to salt used by the family **Unit of allocation:** adults (aged 18 years or older) **Start date:** March 1997 **End date:** March 1999 **Relevant study limitations as reported by study authors:** Low follow‐up rate (80%) may have resulted in loss of statistical power. **Sample size calculation:** NR	
Participants	**Baseline Characteristics** LSSS intervention *Age*: before run‐in period in years, mean (SD): 20.7 (2.0) *Gender*: Female; % (n/N): 47.3 (52/110) *Ethnicity/race*: Chinese *Smoking*: NR *Body Mass Index (BMI)*: before run‐in period in kg/m^2^, mean (SD): 21.9 (2.9) *Blood pressure status*: Hypertensive, % (n/N): 100 (110/110) *Antihypertensive medication used*: NR *Cardiovascular disease or stroke*: NR *Diabetes mellitus*: NR *Renal impairment*: NR *Dietary potassium intake*: NR *Dietary sodium intake*: NR *Urinary potassium excretion*: in mmol, mean (SD): 4.8 (2.3) *Urinary sodium excretion*: in mmol, mean (SD): 62.4 (28.2) Control *Age*: before run‐in period in years, mean (SD): 20.4 (2.2) *Gender*: Female, % (n/N): 49 (54/110) *Ethnicity/race*: Chinese *Smoking*: NR *Body Mass Index (BMI)*: before run‐in period in kg/m^2^, mean (SD): 22.2 (3.1) *Blood pressure status*: Hypertensive, % (n/N): 100 (110/110) *Antihypertensive medication used*: NR *Cardiovascular disease or stroke*: NR *Diabetes mellitus*: NR *Renal impairment*: NR *Dietary potassium intake*: NR *Dietary sodium intake*: NR *Urinary potassium excretion*: in mmol, mean (SD): 4.7 (2.2) *Urinary sodium excretion*: in mmol, mean (SD): 63.9 (27.6) **Inclusion criteria:** Adults, aged 18 to 22 years with high blood pressure (above 90th percentile for age and gender) at multiple follow‐up visits during the previous 8‐year period **Exclusion criteria:** NR **Pretreatment:** None **Method of recruitment of participants:** Recruited from 4623 adolescents in the cardiovascular disease prevention and treatment area of Hanzhong city **Informed consent obtained:** NR **Clusters:** n/a **Subgroups planned/measured:** NR **Subgroups reported:** NR *Participant flow* **Assessed for eligibility:** NR **Excluded (number with reasons):** NR **Randomised:** n = 220 **Allocated to LSSS intervention(s):** n = 110 **Allocated to control:** n = 110 **Received allocated LSSS intervention(s):** NR **Did not receive allocated LSSS intervention(s):** NR **Lost to follow‐up (LSSS intervention group):** n = 22 (no reasons reported) **Discontinued intervention (LSSS intervention group):** NR **Analysed (LSSS intervention group):** n = 88 **Excluded from analysis (LSSS intervention group):** NR **Received allocated control:** NR **Did not receive allocated control:** NR **Lost to follow‐up (control group):** n = 13 (no reasons reported) **Discontinued intervention (control group):** NR **Analysed (control group):** n = 97 **Excluded from analysis (control group):** NR	
Interventions	**Intervention Characteristics** LSSS intervention *Theoretical basis*: Low potassium and calcium intakes are important risk factors for the development of hypertension in Chinese population. Potassium and calcium supplementation may therefore prevent the development of hypertension in adolescents in China. *Description*: Salt with added potassium and calcium (10 mmol per person per day) *LSSS category*: Unknown *Contains fortificant*: NR *Delivery*: Discretionary use *Duration of run‐in period*: 1 month *Duration of active intervention*: 2 years *Duration of follow‐up (as reported)*: none *Total duration of study (as reported)*: 2 years *Timing*: Daily use by household *Implementation*: "provided to each family once a month by a specially assigned person to understand the consumption and adverse reactions." *Providers*: NR *Co‐interventions*: None. "No other interventions were performed". *Resource requirements*: Salt provided free of charge *Integrity of delivery*: NR Control *Theoretical basis*: To enable comparison of LSSS intervention with standard practice *Description*: Salt without added potassium and calcium *Contains fortificant*: NR *Delivery*: Discretionary use *Duration of run‐in period*: 1 month *Duration of active intervention*: 2 years *Duration of follow‐up (as reported)*: none *Total duration of study (as reported)*: 2 years *Timing*: Daily use by household *Implementation*: Provided once a month during home follow‐up visit *Providers*: NR *Co‐interventions*: None. "No other interventions were performed". *Resource requirements*: Salt provided free of charge *Integrity of delivery*: Participants recorded their salt consumption. Amount of salt consumed: NR	
Outcomes	Primary outcomes: **Diastolic Blood Pressure (DBP):** Outcome measurement: measured according to the standard set by WHO; time points: 2 years **Systolic Blood Pressure (SBP):** Outcome measurement: measured according to the standard set by WHO; time points: 2 years **Hypertension (as reported, or SBP > 140 mmHg or DBP > 85 mmHg):** NR **Blood pressure control (achieving blood pressure threshold or 'control' as prespecified):** NR **Cardiovascular events‐stroke:** NR **Cardiovascular events‐myocardial infarction:** NR **Cardiovascular events‐other:** NR **Cardiovascular mortality:** NR **Blood potassium:** NR **Hyperkalaemia:** NR **Hypokalaemia:** NR Secondary outcomes: **All‐cause mortality:** NR **Adverse events:** NR **Antihypertensive medication use:** NR **Body Mass index (BMI):** NR **Serum creatinine:** NR **Albuminuria:** NR **Urinary albumin‐to‐creatinine ratio (uACR):** NR **Fasting blood glucose:** NR **Blood triglycerides:** NR **Total blood cholesterol:** NR **24‐h urinary sodium excretion:** NR **24‐h urinary potassium excretion:** NR	
Notes	**Funding source:** NR **Authors name:** Mu Jian‐jun **Institution:** The First Hospital of Xi of Jiaotong University **Email:** NR **Possible conflicts of interest (for study authors):** NR **Sources used for data extraction:** Journal article with results of the trial **Trial registration details:** NR	
***Risk of bias***		
**Bias**	**Authors' judgement**	**Support for judgement**
Random sequence generation (selection bias)	Unclear risk	Study reported to be randomised but insufficient information on method reported
Allocation concealment (selection bias)	Unclear risk	Insufficient information available
Blinding of participants and personnel (performance bias) All outcomes	Unclear risk	The study authors referred to the study as 'single‐blind'; unclear what this meant.
Blinding of outcome assessment (detection bias) All outcomes	Unclear risk	Study only reported 'single blinding'. The study authors reported the use of standardised BP measurements; however, it was unclear if an automated device was used.
Incomplete outcome data (attrition bias) All outcomes	High risk	Attrition was greater than 10% in both groups: 20% (22/110) intervention group and 11.8% (13/110) in the control group. Reasons for lost to follow‐up data were not reported.
Selective reporting (reporting bias)	Unclear risk	Study protocol or prospective trial registry entry not available
Other bias	Low risk	None identified
Overall risk of bias	High risk	Unclear risk of bias for allocation concealment; high risk of bias for incomplete outcome data

**Table:** Mu 2009

***Study characteristics***		
Methods	**Study design:** Cluster‐randomised controlled trial **Country:** China **Setting:** Intervention was conducted in rural villages in Hanzhong; setting where outcomes were measured: NR **Aim of study:** To observe the effects of dietary salt with added potassium and calcium on the BP of adolescents and their family members **Unit of allocation:** families or households **Start date:** NR **End date:** NR **Relevant study limitations as reported by study authors:** NR **Sample size calculation:** NR	
Participants	**Baseline Characteristics** LSSS intervention (participants with hypertension) *Age*: in years, mean (SD): 20.3 (3.1) *Gender*: Female, % (n/N): 55.5 (56/101) *Ethnicity/race*: Chinese *Smoking*: NR *Body Mass Index (BMI)*: in kg/m^2^, mean (SD): 23.6 (2.0) *Blood pressure status*: Hypertensive, % (n/N): 100 (101/101) *Antihypertensive medication used*: NR *Cardiovascular disease or stroke*: NR *Diabetes mellitus*: NR *Renal impairment*: Serum creatinine above the normal range, % (n/N): 0 (0/101) *Dietary potassium intake*: 3‐day dietary recall, in mmol/day, mean (SD) 37.1 (16.6) *Dietary sodium intake*: 3‐day dietary recall, in mmol/day, mean (mean (SD): 140 (59)) *Urinary potassium excretion*: overnight 8‐hour in mmol, mean (SD): 4.8 (2.3) *Urinary sodium excretion*: overnight 8‐hour in mmol, mean (SD): 61.8 (28.9) Control (participants with hypertension) *Age*: in years, mean (SD): 21.4 (3.9) *Gender*: Female, % (n/N): 47.4 (54/114) *Ethnicity/race*: Chinese *Smoking*: NR *Body Mass Index (BMI)*: in kg/m^2^, mean (SD): 23.8 (2.1) *Blood pressure status*: Hypertensive, % (n/N): 100 (114/114) *Antihypertensive medication used*: NR *Cardiovascular disease or stroke*: NR *Diabetes mellitus*: NR *Renal impairment*: Serum creatinine above the normal range, % (n/N): 0 (0/114) *Dietary potassium intake*: 3‐day dietary recall, in mmol/day, mean (SD: 36.6 (13.7) *Dietary sodium intake*: 3‐day dietary recall, in mmol/day, mean (SD): mean (SD): 137 (49) *Urinary potassium excretion*: overnight 8‐hour in mmol, mean (SD): 4.7 (2.2) *Urinary sodium excretion*: overnight 8‐hour in mmol, mean (SD): 64.1 (27.0) LSSS intervention (family members) *Age*: NR *Gender*: NR *Ethnicity/race*: NR *Smoking*: NR *Body Mass Index (BMI)*: NR *Blood pressure status*: NR *Antihypertensive medication used*: NR *Cardiovascular disease or stroke*: NR *Diabetes mellitus*: NR *Renal impairment*: NR *Dietary potassium intake*: NR *Dietary sodium intake*: NR *Urinary potassium excretion*: NR *Urinary sodium excretion*: NR Control (family members) *Age*: NR *Gender*: NR *Ethnicity/race*: NR *Smoking*: NR *Body Mass Index (BMI)*: NR *Blood pressure status*: NR *Antihypertensive medication used*: NR *Cardiovascular disease or stroke*: NR *Diabetes mellitus*: NR *Renal impairment*: NR *Dietary potassium intake*: NR *Dietary sodium intake*: NR *Urinary potassium excretion*: NR *Urinary sodium excretion*: NR **Inclusion criteria:** Families with young adults with high BP (defined as SBP = 90th percentile by age and sex) and other family members who were prepared to eat all dinners at home throughout the study period **Exclusion criteria:** Families with any family member who reported the use of a potassium‐sparing medication or with significant renal impairment (defined by study authors and confirmed by responsible physician as an abnormal serum creatinine concentration) or hyperkalaemia **Pretreatment:** There were more family members included in the LSSS intervention, compared to the control group (334 vs. 254) at baseline. **Method of recruitment of participants:** Adolescents who had been followed up for 10 years were screened (recruitment method not described) **Informed consent obtained:** Yes (unclear whether verbal or written) **Clusters:** families or households **Subgroups planned/measured:** NR **Subgroups reported:** Young adults with hypertension vs. family members; salt‐sensitive (SS) subjects vs. non‐salt‐sensitive (NSS) group *Participant flow* **Assessed for eligibility:** n = 350 families with young adults with hypertension **Excluded (number with reasons):** n = 25 families with young adults with hypertension (inability to have their dinners at home all or nearly all the time throughout the study) **Randomised:** n = 325 families; n = 325 young adults with hypertension; (n = 101 LSSS intervention group; n = 110 salt‐restricted intervention group; n = 114 control group and n = 334 family members) and n = 978 family members (n = 334 LSSS intervention group; n = 338 salt‐restricted intervention group; n = 114 control group) **Allocated to LSSS intervention/s:** n = 101 families; n = 110 young adults with hypertension (n = 334 family members) **Allocated to control:** n = 114 families; n = 114 young adults with hypertension (n = 254 family members) **Received allocated LSSS intervention/s:** NR **Did not receive allocated LSSS intervention/s:** NR **Lost to follow‐up (LSSS intervention group):** NR [Young adults with hypertension: Total loss to follow‐up reported for 2 intervention groups and control group as 9.8% (32/325): n = 18 moved away; n = 4 joined the army; n = 8 remote places to work; n = 2 other reasons; family members: total loss to follow‐up reported as 14.3%] **Discontinued intervention (LSSS intervention group):** NR **Analysed (LSSS intervention group):** NR **Excluded from analysis (LSSS intervention group):** NR **Received allocated control:** NR **Did not receive allocated control:** NR **Lost to follow‐up (control group):** NR [Young adults with hypertension: Total loss to follow‐up reported for 2 intervention groups and control group as 9.8% (32/325): n = 18 moved away; n = 4 joined the army; n = 8 remote places to work; n = 2 other reasons; family members: total loss to follow‐up reported as 14.3 **Discontinued intervention (control group):** NR **Analysed (control group):** NR **Excluded from analysis (control group):** NR	
Interventions	**Intervention Characteristics** LSSS intervention (total) *Theoretical basis*: Adding potassium and calcium to cooking salt will elevate the ratios of K and Ca to Na, and thereby may reduce the rise in BP with age. *Description*: Added potassium and calcium salt (each family member to consume approx. additional 10 mmol K and 10 mmol Ca per day) *LSSS category*: unknown *Contains fortificant*: NR *Delivery*: discretionary use *Duration of run‐in period*: none *Duration of active intervention*: 2 years *Duration of follow‐up (as reported)*: none *Total duration of study (as reported)*: 2 years *Timing*: Daily household use at the table and for cooking *Implementation*: NR *Providers*: NR *Co‐interventions*: NR *Resource requirements*: Salt supplied free of charge to households *Integrity of delivery*: Researchers visited each family kitchen to assess the amount of salt consumed every month. Data not reported. Participants were asked to eat all their dinners at home throughout the study duration. Every 6 months, an interview about the past 3 days’ food consumption was conducted (Potassium intake, in mmol/day, mean (SD): 46 (15) at 6 months; 35 (13) at 12 months and 39 (15) at 2 years; sodium intake, in mmol/day, mean (SD): 144 (54) at 6 months; 134 (39) at 12 months and 136 (40) at 2 years). Control (total) *Theoretical basis*: To enable comparison of LSSS intervention with standard practice *Description*: Salt without added potassium *Contains fortificant*: NR *Delivery*: discretionary *Duration of run‐in period*: none *Duration of active intervention*: 2 years *Duration of follow‐up (as reported)*: none *Total duration of study (as reported)*: 2 years *Timing*: Daily household use at the table and for cooking *Implementation*: NR *Providers*: NR *Co‐interventions*: NR *Resource requirements*: Salt supplied free of charge to households *Integrity of delivery*: Researchers visited each family kitchen to assess the amount of salt consumed every month. Data not reported. Participants were asked to eat all their dinners at home throughout the study duration. Every 6 months, an interview about the past 3 days’ food consumption was conducted (Potassium intake, in mmol/day, mean (SD): 47 (15) at 6 months; 36 (14) at 12 months and 36 (18) at 2 years; sodium intake, in mmol/day, mean (SD): 127 (31) at 6 months; 138 (48) at 12 months and 135 (46) at 2 years).	
Outcomes	**Primary outcomes: ** **Diastolic Blood Pressure (DBP):** Outcome measurement: Clinic‐based measurements performed using the auscultatory method after 5 mins rest, with patient in seating position; Mean value of three measurements (performed with 30 s in between measurements); time points: 6, 12, 24 months **Systolic Blood Pressure (SBP):** Outcome measurement: Clinic‐based measurements performed using the auscultatory method after 5 mins rest, with patient in seating position; Mean value of three measurements (performed with 30 s in between measurements); time points: 6, 12, 24 months **Hypertension (as reported, or SBP > 140 mmHg or DBP > 85 mmHg):** NR **Blood pressure control (achieving blood pressure threshold or 'control' as prespecified):** NR **Cardiovascular events‐stroke:** NR **Cardiovascular events‐myocardial infarction:** NR **Cardiovascular events‐other:** NR **Cardiovascular mortality:** NR **Blood potassium:** NR **Hyperkalaemia:** Outcome measurement: NR; time points: duration of the study **Hypokalaemia:** NR **Secondary outcomes: ** **All‐cause mortality:** NR **Adverse events:** Any (including hypercalcemia, kidney calculi); outcome measurement: NR; time points: duration of the study **Antihypertensive medication use:** NR **Body Mass index (BMI):** NR **Serum creatinine:** NR **Albuminuria:** NR **Urinary albumin‐to‐creatinine ratio (uACR):** NR **Fasting blood glucose:** NR **Blood triglycerides:** NR **Total blood cholesterol:** NR **24‐h urinary sodium excretion:** NR **24‐h urinary potassium excretion:** NR	
Notes	**Funding source:** National Health Ministry of China **Authors name:** Jianjun Mu **Institution:** Department of Cardiology, First Affiliated Hospital of Medical school of Xi’an Jiaotong University, Xi’an, People’s Republic of China **Email:** mujjun@163.com **Possible conflicts of interest (for study authors):** "The authors declared no conflict of interest." **Sources used for data extraction:** Journal article with results of the trial **Trial registration details:** NR **Author correspondence:** Dr. Mu was contacted on 23/07/2020 to provide us with outcome data (mean changes in SBP, DBP). Received no reply	
***Risk of bias***		
**Bias**	**Authors' judgement**	**Support for judgement**
Random sequence generation (selection bias)	Low risk	Participants were randomised by coin toss to either intervention or control.
Allocation concealment (selection bias)	Unclear risk	Insufficient information available
Blinding of participants and personnel (performance bias) All outcomes	Unclear risk	The study was described as 'single‐blinded' ‐ unclear what this meant.
Blinding of outcome assessment (detection bias) All outcomes	Unclear risk	The study only reported 'single‐blinded' but no detail on who was blinded. BP was not measured with an automatic device.
Incomplete outcome data (attrition bias) All outcomes	Unclear risk	Attrition not reported per treatment group. Overall attrition for hypertensive young adults was reported as 9.8%, and 14.3% overall for family members.
Selective reporting (reporting bias)	Unclear risk	No study protocol or prospective trial registry entry available
Other bias	Low risk	None identified
Recruitment bias (cluster‐RCTs)	Unclear risk	It was unclear whether all the eligible family members within families were identified before randomisation of the families. More family members were included in the LSSS intervention, compared to the control group (334 vs. 254) at baseline, suggesting differential selection bias.
Comparability with individually randomised trials (cluster‐RCTs)	Unclear risk	The China Salt Substitute study (RCT) demonstrated a lowering effect on SBP in high‐risk adults with the use of a LSSS. A RCT (Liu 1996) among school children showed that calcium and potassium supplements (capsules) may limit the rise of BP with age; however, this was not a LSSS intervention.
Loss of clusters (cluster‐RCTs)	Unclear risk	Insufficient information provided on the number of families or households that were lost to follow‐up
Baseline imbalance (cluster‐RCTs)	Unclear risk	The baseline characteristics of the hypertensive young adults were similar between the 3 groups. However, the characteristics of the family members were not reported.
Incorrect analysis (cluster‐RCTs)	Low risk	Family codes were included as a random factor in the general linear modelling analysis.
Overall risk of bias	Unclear risk	Unclear risk of bias for incomplete outcome data, baseline imbalance, recruitment bias and loss of clusters

**Table:** Neal 2021

***Study characteristics***		
Methods	**Study design:** Cluster‐randomised controlled trial **Country:** China **Setting:** Intervention conducted in villages from five provinces Hebei, Liaoning, Ningxia, Shanxi and Shaanxi in northern region. NR where outcomes were measured **Comments:** SSaSS: Salt Substitute and Stroke Study **Aim of study:** The primary objective of the SSaSS is to determine the effects of sodium reduction achieved through the use of a reduced‐sodium, added potassium salt substitute on the risk of fatal or non‐fatal stroke. The secondary objectives are to determine the effects of the intervention on (1) major vascular events, a composite of non‐fatal stroke, non‐fatal acute coronary syndrome, and vascular death, and (2) total mortality. **Unit of allocation:** Villages **Start date:** April 2014 **End date:** March 2021 **Relevant study limitations as reported by study authors:** Neal 2021: No serial assessments of blood potassium, therefore, hyperkalaemia may have been missed in some participants. Dose‐response analysis could not be conducted since only one type of LSSS was used. No concealment of intervention delivery; however, objective primary and secondary outcomes were reported. In addition, systematic searches of routinely collected health data and verification by an independent end‐point adjudication committee, the members of which were unaware of the trial group assignments, was conducted, but authors stated that "Information that could be used for adjudication of outcome events was limited, and definitive assignment of causation was difficult in many cases.” No evidence was found to suggest certainty of adjudications had any substantive implications for the primary conclusions of the trial. Huang 2020: Hyperkalaemia risk in the study did not include vulnerable populations (e.g. CKD). Increased uncertainty of the effect estimates for intermediate outcomes due to the cluster‐randomised trial design (compared to an individual‐randomised trial). Incomplete participation of invited participants and incomplete collection of 24‐hour urine collections in interim surveys (however: those that participated were broadly representative of overall study population); and absence of 24‐hour urine for two provinces at baseline **Sample size calculation:** Estimated sample size of 21,000 participants in 600 clusters (n = 300 in the LSSS intervention group; n = 300 in control group, with 35 participants in each cluster) to detect a >= 13% relative risk reduction for stroke in the intervention villages compared with control villages, with 90% statistical power and 95% confidence level (two‐sided). The estimate assumed a primary outcome event rate of 3.5% per year, a systolic blood pressure difference of 3.0 mmHg between randomised groups, and an ICC of 0.04.	
Participants	**Baseline Characteristics** LSSS intervention *Age*: in years, mean (SD): 65.2 (8.5) *Gender*: Female, % (n/N): 49.7 (5221/10 505) *Ethnicity/race*: Chinese *Smoking*: Current smoking, % (n/N): 18.7 (1964/10505); past smoking, % (n/N): 32.8 (3446/10505) *Body Mass Index (BMI)*: in kg per m^2^, mean (SD): 24.8 (3.6) *Blood pressure status*: Uncontrolled hypertension, % (n/N): 59.4 (6240/10505) *Antihypertensive medication used*: Any antihypertensive medication, % (n/N): 79.9 (8394/10 505); diuretic, % (n/N): 11.5 (1208/10 505); ACE inhibitor or ARB, % (n/N): 23.1 (2427/10 505); alpha‐blocker, % (n/N): 0.7 (95/10 505); beta‐blocker, % (n/N): 5 (525/10 505); calcium antagonist, % (n/N): 43.3 (4549/10 505) *Cardiovascular disease or stroke*: History of stroke, % (n/N): 73.3 (7700/10 505); transient ischaemic attack, % (n/N): 13.2. (1387/10 505); ischaemic heart disease, % (n/N): 16.4 (1723/10 505) *Diabetes mellitus*: Type 2 DM, % (n/N): 10.6 (1114/10505) *Renal impairment*: Known serious kidney disease, % (n/N): 0 (0/10505) *Dietary potassium intake*: NR *Dietary sodium intake*: NR *Urinary potassium excretion*: in (g/d), mean (SD): 1.4 (0.6) *Urinary sodium excretion*: in (g/d), mean (SD): 4.4 (1.8) Control *Age*: in years, mean (SD): 65.5 (8.5) *Gender*: Female, % (n/N): 49.2 (5162/10491) *Ethnicity/race*: Chinese *Smoking*: Current smoking, % (n/N): 18.9 (1983/10491); past smoking, % (n/N): 34.2 (3588/10491) *Body Mass Index (BMI)*: in kg per m^2^, mean (SD): 24.9 (3.7) *Blood pressure status*: Uncontrolled hypertension, % (n/N): 59.2 (6211/10,491) *Antihypertensive medication used*: Any antihypertensive medication, % (n/N): 78.7 (8256/10 491); diuretic, % (n/N): 11.2 (1175/10 491); ACE inhibitor or ARB, % (n/N):23 (2413/10 491); alpha‐blocker, % (n/N): 1.1 (115/10 491); beta‐blocker, % (n/N): 6.3 (661/10 491); calcium antagonist, % (n/N): 40.6 (4259/10 491) *Cardiovascular disease or stroke*: History of stroke, % (n/N): 72.0 (7554/10 491); transient ischaemic attack, % (n/N): 14.9 (1563/10 491); ischaemic heart disease, % (n/N): 15.7 (1647/10 491) *Diabetes mellitus*: Type 2 DM, % (n/N): 10.5 (1102/10491) *Renal impairment*: Known serious kidney disease, % (n/N): 0 (0/10491) *Dietary potassium intake*: NR *Dietary sodium intake*: NR *Urinary potassium excretion*: in (g/d), mean (SD): 1.4 (0.6) *Urinary sodium excretion*: in (g/d), mean (SD): 4.2 (1.8) Overall *Age*: NR *Gender*: NR *Ethnicity/race*: NR *Smoking*: NR *Body Mass Index (BMI)*: NR *Blood pressure status*: NR *Antihypertensive medication used*: NR *Cardiovascular disease or stroke*: NR *Diabetes mellitus*: NR *Renal impairment*: NR *Dietary potassium intake*: NR *Dietary sodium intake*: NR *Urinary potassium excretion*: NR *Urinary sodium excretion*: NR **Inclusion criteria:** Individuals in the selected villages with an elevated risk of stroke, defined as a history of stroke (regardless of type or knowledge of aetiology) and/or age 60 years or greater with uncontrolled high blood pressure (systolic blood pressure >= 140 mm Hg at visit if on blood pressure–lowering medication or systolic blood pressure >= 160 mm Hg if not on blood pressure–lowering medication). Participants were also required to be contactable by telephone and be able to nominate a friend or relative through which they could also be contacted, if necessary. **Exclusion criteria:** Participant or family members were excluded if they: (1) or someone living in their household had a potential contraindication to the salt substitute used in the trial; contraindications included use of a potassium‐sparing diuretic, use of a potassium supplement, or known serious kidney disease; (2) had serious renal impairment; (3) were taking potassium‐sparing diuretic; (4) were taking potassium supplement;(5) had concerns about using salt substitute; (6) ate most meals outside home; (7) were expected to live less than 6 months from date of baseline assessment by village doctors **Pretreatment:** No group differences **Method of recruitment of participants:** Two counties per province were selected by the research team based upon their willingness to participate, their proximity to the local research team and being representative of the socioeconomic level of each province. Sixty villages in each of the counties were recruited through a consent process involving the leadership of the local county Bureaus of Health (identified villages that were willing and accessible). About 35 individuals at elevated risk of stroke were recruited in each village. **Informed consent obtained:** Yes (written) **Clusters:** n = 600; cluster size: n = 35 individuals **Subgroups planned/measured:** NR **Subgroups reported:** Age > 65 years vs. age <= 65 years; female vs. male; less educated vs. more educated; cerebrovascular disease vs. no cerebrovascular disease; diabetes vs. no diabetes; hypertensive vs. non‐hypertensive; using antihypertensive medication vs. not using antihypertensive medication; SBP > 153 mmHg vs. SBP <= 153 mmHg; DBP > 89 mmHg vs. <= 89 mmHg and BMI > 24.6 kg/m^2^ vs. BMI <= 24.6 kg/m^2^ *Participant flow* **Assessed for eligibility:** NR **Excluded (number with reasons):** n = 1 individual (death) **Randomised:** n = 600 villages (n = 20,995 individuals) **Allocated to LSSS intervention(s):** n = 300 villages (n = 10,504 individuals) **Allocated to control:** n = 300 villages (n = 10,491 individuals) **Received allocated LSSS intervention(s):** NR **Did not receive allocated LSSS intervention(s):** NR **Lost to follow‐up (LSSS intervention group):** n = 1969 individuals (death) **Discontinued intervention (LSSS intervention group):** NR **Analysed (LSSS intervention group):** n = 10,504 (cardiovascular outcomes ‐ fatal or non‐fatal stroke; major cardiovascular outcomes; all‐cause mortality) n = 786 at 12 months (DBP, SBP); n = 1412 at 24 months (DBP, SBP); n = 584 at 36 months (DBP, SBP); n = 587 at 48 months (DBP, SBP); n = 7436 (60 months) (DBP, SBP) **Excluded from analysis (LSSS intervention group):** NR **Received allocated control:** NR **Did not receive allocated control:** NR **Lost to follow‐up (control group):** 2203 individuals (death) **Discontinued intervention (control group):** NR **Analysed (control group):** n = 10,491 (cardiovascular outcomes ‐ fatal or non‐fatal stroke; major cardiovascular outcomes; all‐cause mortality) n = 658 at 12 months (DBP, SBP); n = 1374 at 24 months (DBP, SBP); n = 584 at 36 months (DBP, SBP); n = 559 at 48 months (DBP, SBP); n = 7081 (60 months) (DBP, SBP) **Excluded from analysis (control group):** NR	
Interventions	**Intervention Characteristics** LSSS intervention *Theoretical basis*: Lowering sodium intake and increasing potassium intake with a reduced‐sodium, added‐potassium salt substitute may reduce the risk of stroke by lowering BP. *Description*: Reduced sodium, added‐potassium salt substitute (30 ± 10% KCl; 70 ± 10% NaCl at average intake of 20 g per person per day, to a maximum of 20 kg per year for a household with 3 members *LSSS category*: ≥ 30% KCl *Contains fortificant*: NR *Delivery*: Discretionary use *Duration of run‐in period*: None *Duration of active intervention*: mean duration of 4.74 years *Duration of follow‐up (as reported)*: none *Total duration of study (as reported)*: mean duration of 4.74 years *Timing*: Participants provided with sufficient quantity to cover all cooking and food preservation activities *Implementation*: Village doctors advise participants to use the salt substitute more sparingly, and not more frequently, than they previously used salt. Distributed quarterly. *Providers*: Local provider in each county. Village doctors provided regular supply of LSSS to each household. *Co‐interventions*: NR *Resource requirements*: LSSS purchased from local providers; provided free of charge to trial households *Integrity of delivery*: Oral, written and other reminders (e.g. cooking apron) to reinforce use of LSSS. Adherence assessed with questionnaire in random subsets of participants at 12, 24, 36, 48 and 60 months Control *Theoretical basis*: To enable comparison of LSSS intervention with standard practice *Description*: Usual salt used by household; advice about reducing salt intake given at study commencement *Contains fortificant*: NR *Delivery*: Discretionary use *Duration of run‐in period*: none *Duration of active intervention*: mean duration of 4.74 years *Duration of follow‐up (as reported)*: none *Total duration of study (as reported)*: mean duration of 4.74 years *Timing*: Participants provided with sufficient quantity to cover all cooking and food preservation activities *Implementation*: Advice about reducing salt intake given at study commencement *Providers*: Own household supply *Co‐interventions*: NR *Resource requirements*: NR *Integrity of delivery*: "Participants in the control villages continue with their use of usual salt with advice about reducing salt intake given only at the commencement of the study."	
Outcomes	**Primary outcomes: ** **Diastolic Blood Pressure (DBP):** Outcome measurement: automated devices (Omron Electronic Blood Pressure Monitor) with participants in a sitting position. Two measurements were taken at least 5 minutes apart with the average of the two measurements used for the analysis; time points: 12, 24, 36, 48 months (random samples of at least 20 participants drawn from a stratified random sample of at least 60 villages) and 60 months (all available and surviving participants) **Systolic Blood Pressure (SBP):** Outcome measurement: automated devices (Omron Electronic Blood Pressure Monitor) with participants in a sitting position. Two measurements were taken at least 5 minutes apart with the average of the two measurements used for the analysis; time points: 12, 24, 36, 48 months (random samples of at least 20 participants drawn from a stratified random sample of at least 60 villages) and 60 months (all available and surviving participants) **Hypertension (as reported, or SBP > 140 mmHg or DBP > 85 mmHg):** NR **Blood pressure control (achieving blood pressure threshold or 'control' as prespecified):** NR **Cardiovascular events‐stroke:** Outcome measurement: fatal and non‐fatal stroke defined as an acute disturbance of focal neurological function resulting in death or symptoms lasting more than 24 hours; imaging, clinical and laboratory data will be collected wherever possible. Time points: every 6 months for the duration of the study period (5 years) **Cardiovascular events‐myocardial infarction:** NR **Cardiovascular events‐other:** Outcome measurement: non‐fatal stroke (defined as an acute disturbance of focal neurological function resulting in symptoms lasting more than 24 hours); acute coronary syndrome (myocardial infarction or unstable angina event that leads to a hospitalisation, defined by 2 of the following: a clinical history, electrocardiogram changes, and enzyme levels). Time points: every 6 months for the duration of the study (5 years) **Cardiovascular mortality:** Outcome measurement: Vascular death from an acute coronary syndrome event, stroke, heart failure, suspected vascular causes, or sudden cardiac death. Time points: every 6 months for the duration of the study (5 years) **Blood potassium:** NR **Hyperkalaemia:** Outcome measurement: defined as either a serum potassium concentration greater than 6.0 mmol/L with or without other positive findings or a serum potassium concentration greater than 5.5 mmol/L with evidence of typical electrocardiogram changes; time points: duration of study period (5 years) **Hypokalaemia:** NR **Secondary outcomes: ** **All‐cause mortality:** Outcome measurement: A verbal autopsy completed with the help of a caregiver that witnessed the circumstances leading to death; cause of death grouped according to the chapter headings of the Revision of International Statistical Classification of Diseases and Related Health Problems (ICD‐10) version 2016, with additional granularity of reporting done for selected common outcomes of the circulatory system; time points: every 6 months for the duration of the study (5 years) **Adverse events:** NR **Antihypertensive medication use:** NR **Body Mass index (BMI):** NR **Serum creatinine:** NR **Albuminuria:** NR **Urinary albumin‐to‐creatinine ratio (uACR):** NR **Fasting blood glucose:** NR **Blood triglycerides:** NR **Total blood cholesterol:** NR **24‐h urinary sodium excretion:** 24‐hour urine sample. Time points: 60 months **24‐h urinary potassium excretion:** 24‐hour urine sample. Time points: 60 months	
Notes	**Funding source:** Supported by research grants (APP1164206 and APP1049417) and an investigator grant (APP1197709, to Dr. Neal) from the National Health and Medical Research Council of Australia. Dr J HY Wu is supported by a UNSW Scientia Fellowship. Salt substitute donated by Jiangsu Sinokone Technology for years 3 to 4 of the study **Authors name:** Bruce Neal **Institution:** Sydney School of Public Health, University of Sydney, Sydney, Australia. **Email:** bneal@georgeinstitute.org.au **Possible conflicts of interest (for study authors):** Paul Elliott declared various grants from non‐profit organisations and is an unpaid member of 'Action on Salt and health; World Action on Salt, Sugar and health'; Dr. Yangfeng Wu received a research grant from Health Source (Chongqing) Cardiovascular Health Technology Co. (a pilot study on the effect of very low‐sodium salt substitute among patients with hypertension); other study authors declared no conflict of interest. **Sources used for data extraction:** Journal article(s) with results of the trial; trial protocol (published; trial registry); conference abstract **Trial registration details:** NCT02092090	
***Risk of bias***		
**Bias**	**Authors' judgement**	**Support for judgement**
Random sequence generation (selection bias)	Low risk	The authors stated that county‐stratified random assignment occurred through a central computerised process, done by an independent biostatistician.
Allocation concealment (selection bias)	Low risk	Randomisation was conducted by an independent biostatistician through a central computerised process.
Blinding of participants and personnel (performance bias) All outcomes	Low risk	Although participants and personnel involved with study implementation, e.g. village doctors, were not blinded, the risk of bias was minimised by the measurement of objective outcomes such as clinical events or measurements such as blood pressure.
Blinding of outcome assessment (detection bias) All outcomes	Low risk	The trial registry indicated that investigators and outcomes assessors were blinded to allocation, measurement of process indicators were intended to be measured separately to avoid unblinding, and it appeared as though an analysis plan was finalised before data unblinding. However, Huang and colleagues 2020 reported that ‘the trial has an unavoidable open design and biases consequent upon the unblinded design cannot be entirely excluded’. Endpoint adjudication committees were blinded.
Incomplete outcome data (attrition bias) All outcomes	Low risk	Mortality: Both groups had complete follow‐up outcome data. Cardiovascular events: Non‐fatal events were assessed among the participants for 99,473 of 99,522 person‐years (> 99.9%) of scheduled follow‐up. SBP and DBP: Death was the main reason for the attrition in both groups at 60‐months follow‐up. Reported attrition, other than death, was 10.5% (1099/10504) in the intervention vs. 11.5% (1207/10491) in the control group. Data for mortality were complete, and data for events could also be found for all surviving participants (albeit from sources with different quality and risk for misclassification). Attrition for blood pressure measurements in participants surviving at 60 months was 1099/8535 (13%) in the intervention and 1207/8288 (15%) in the control arm, however, there appeared to be no difference in the reasons for most of the missingness (followed up through nominated contact, followed up through record linkage, followed up at previous visit). Blood pressure measurements for participants followed up in person remained unaccounted for in 286/7722 (4%) participants in the intervention and 327/7408 (4%) participants in the control arm.
Selective reporting (reporting bias)	Unclear risk	Some prespecified outcomes e.g. total major vascular events (as a composite outcome) were not reported. The study authors reported subgroup analyses, which was not prespecified.
Other bias	High risk	Misclassification bias: The study authors reported that information used for adjudication of outcome events was limited, and definitive assignment of causation was difficult in many cases. However, they reported no evidence of a potential effect of misclassification of clinical end‐points on effect estimates. Further, the authors stated that the lack of serial monitoring of blood potassium in the study may have resulted in missing participants with hyperkalaemia. Definite and probable hyperkalaemia events were defined on the basis of medical reports documenting a serum potassium > 5.5 mmol/L or ECG or enzyme changes. A post hoc decision was taken to classify participants with possible hyperkalaemia (based on self‐report; not requiring any supporting documentation).
Recruitment bias (cluster‐RCTs)	Low risk	All participants in the selected villages were recruited before villages were allocated to the intervention or control group.
Comparability with individually randomised trials (cluster‐RCTs)	Unclear risk	There were no RCTs that reported on long‐term cardiovascular clinical outcomes, following LSSS interventions. To date, some RCTs have reported short term benefits in cardiovascular risk factors, such as reductions in blood pressure (CSSS Collaborative Group 2007; Zhao 2014), while others have not (Gilleran 1996; Mu 2003; Sarkkinen 2011; Suppa 1988).
Loss of clusters (cluster‐RCTs)	Low risk	The complete data on vital status for all participants indicated that it was very unlikely that entire clusters were lost from the study.
Baseline imbalance (cluster‐RCTs)	Low risk	No baseline imbalances reported between the intervention and control groups in terms of demographic characteristics or comorbidity risk
Incorrect analysis (cluster‐RCTs)	Low risk	Adjustment for clustering was made with the use of a hierarchical Poisson regression model.
Overall risk of bias	Low risk	Low risk of bias for incomplete outcome data, baseline imbalance, recruitment bias and loss of clusters

**Table:** Omvik 1995

***Study characteristics***		
Methods	**Study design:** Randomised controlled trial **Study grouping:** Parallel group **Country:** Norway **Setting:** Intervention conducted in households; outcomes measured at an outpatient clinic in Bergen **Aim of study:** To examine whether reductions in intake of dietary sodium, combined with low‐sodium high‐potassium salt substitute instead of standard table salt, would result in clinically meaningful blood pressure reduction among people with hypertension. The study also aimed to determine whether this combination would reduce total peripheral resistance. **Unit of allocation:** adults (aged 18 years or older) **Start date:** NR **End date:** NR **Relevant study limitations as reported by study authors:** NR **Sample size calculation:** NR	
Participants	**Baseline Characteristics** LSSS intervention *Age*: in years, mean (SD): 45.9 (NR) *Gender*: Female, % (n/N): 30 (6/20) *Ethnicity/race*: European *Smoking*: Smokers, % (n/N): 15 (3/20) *Body Mass Index (BMI)*: NR *Blood pressure status*: Primary hypertension, % (n/N): 100 (20/20) *Antihypertensive medication used, *% (n/N): 0 (0/20) *Cardiovascular disease or stroke*: NR *Diabetes mellitus*: NR *Renal impairment*: Serum creatinine above normal range, % (n/N): 0 (0/20) *Dietary potassium intake*: NR *Dietary sodium intake*: NR *Urinary potassium excretion*: in mmol/24 h, mean (SD): 89 (44) *Urinary sodium excretion*: in mmol/24 h, mean (SD): 156 (62) Control *Age*: in years, mean (SD): 42.7 (NR) *Gender*: Female, % (n/N): 35 (7/20) *Ethnicity/race*: European *Smoking*: Smokers, % (n/N): 15 (3/20) *Body Mass Index (BMI)*: NR *Blood pressure status*: Primary hypertension, % (n/N): 100 (20/20) *Antihypertensive medication used, *% (n/N): 0 (0/20) *Cardiovascular disease or stroke*: NR *Diabetes mellitus*: NR *Renal impairment*: Serum creatinine above normal range, % (n/N): 0 (0/20) *Dietary potassium intake*: NR *Dietary sodium intake*: NR *Urinary potassium excretion*: in mmol/24 h, mean (SD): 94 (29) *Urinary sodium excretion*: in mmol/24 h, mean (SD): 156 (57) Overall *Age*: in years, mean (range): 44.3 (25 to 67) *Gender*: Female, % (n/N): 32.5 (13/40) *Ethnicity/race*: European *Smoking*: Smokers, % (n/N): 15 (6/40) *Body Mass Index (BMI)*: NR *Blood pressure status*: Primary hypertension, % (n/N): 100 (40/40) *Antihypertensive medication used, *% (n/N): 0 (0/40) *Cardiovascular disease or stroke*: NR *Diabetes mellitus*: NR *Renal impairment*: Serum creatinine above normal range, % (n/N): 0 (40) *Dietary potassium intake*: NR *Dietary sodium intake*: NR *Urinary potassium excretion*: NR *Urinary sodium excretion*: NR **Inclusion criteria:** Men and women with mild or moderate essential hypertension, according to World Health Organization stages I and II, which were untreated at the time of recruitment were included, but it is not clear whether these were inclusion criteria per se. **Exclusion criteria:** Serum creatinine outside normal limits and secondary hypertension were determined by routine clinical examination, concentration of serum electrolytes, urine analysis, chest X‐ray and, on specific indications, hormonal analysis, radio‐isotope pyelography and renal arteriography. Both were excluded in all participants, but it was not clear whether these were exclusion criteria per se. **Pretreatment:** None **Method of recruitment of participants:** Patients were recruited from an outpatient clinic at the cardiology department of Haukeland Hospital. **Informed consent obtained:** Yes, unclear whether written or oral **Clusters:** n/a **Subgroups planned/measured:** NR **Subgroups reported:** NR *Participant flow* **Assessed for eligibility:** NR **Excluded (number with reasons):** NR **Randomised:** n = 40 **Allocated to LSSS intervention(s):** n = 20 **Allocated to control:** n = 20 **Received allocated LSSS intervention(s):** NR **Did not receive allocated LSSS intervention(s):** NR **Lost to follow‐up (LSSS intervention group):** n = 1 (newly developed angina pectoris) **Discontinued intervention (LSSS intervention group):** n = 0 **Analysed (LSSS intervention group):** n = 19 **Excluded from analysis (LSSS intervention group):** n = 0 **Received allocated control:** NR **Did not receive allocated control:** NR **Lost to follow‐up (control group):** n = 0 **Discontinued intervention (control group):** n = 0 **Analysed (control group):** n = 20 **Excluded from analysis (control group):** n = 0	
Interventions	**Intervention Characteristics** LSSS intervention *Theoretical basis*: To introduce an intervention in participants with primary hypertension which was realistic in its expectations, i.e. moderate sodium reductions, to maintain low salt intake over the long term. Potassium is known to induce peripheral vasodilatation and in clinical studies potassium supplement or potassium rich food may enhance the antihypertensive effect of a low‐sodium diet. *Description*: Pansalt (NaCl 57%; KCl 28%; MgSO_4_ 12%; lysine 2%) *LSSS category*: < 30% KCl *Contains fortificant*: NR *Delivery*: Discretionary use *Duration of run‐in period*: 2 months *Duration of active intervention*: 6 months *Duration of follow‐up (as reported)*: none *Total duration of study (as reported)*: 6 months *Timing*: LSSS to replace all household salt, and be used in small quantities as cooking and table salt throughout the study *Implementation*: Pre‐numbered and identically wrapped 1/2 kg packets of LSSS were handed out to participants, frequency NR. This was combined with instructions to follow a salt‐restricted diet (30% sodium reduction), verbally and in writing. *Providers*: LSSS supplied by Reformi‐Keskus Oriola Oy as well as Searle Norge A/S. Details of persons providing the LSSS and dietary advice not reported *Co‐interventions*: Low‐sodium diet (reduction of 30%) for 6 months *Resource requirements*: NR *Integrity of delivery*: Patients were encouraged to comply with the diet at each follow‐up visit and were asked to remove all the ordinary household salt and replace it with the salt from the Clinic. The study found that patients were able to reduce their sodium intake by approximately 20% over six months. Control *Theoretical basis*: To enable comparison of LSSS intervention with standard practice *Description*: Table salt (standard sodium chloride) *Contains fortificant*: NR *Delivery*: Discretionary use *Duration of run‐in period*: 2 months *Duration of active intervention*: 6 months *Duration of follow‐up (as reported)*: none *Total duration of study (as reported)*: 6 months *Timing*: Standard sodium chloride to replace all household salt, and be used in small quantities as cooking and table salt throughout the study *Implementation*: Pre‐numbered and identically wrapped 1/2 kg packets of standard salt were handed out to participants, frequency NR. This was combined with instructions to follow a salt‐restricted diet (30% sodium reduction), verbally and in writing. *Providers*: NR *Co‐interventions*: Low‐sodium diet (reduction of 30%) for 6 months *Resource requirements*: NR *Integrity of delivery*: Patients were encouraged to comply with the diet at each follow‐up visit and were asked to remove all the ordinary household salt and replace it with the salt from the Clinic. The study found that patients were able to reduce their sodium intake by approximately 20% over six months.	
Outcomes	**Primary outcomes: ** **Diastolic Blood Pressure (DBP):** Outcome measurement: Clinic measurements taken at each visit after 5 mins rest in the seated position. The mean of the last 2 of the 3 readings used for the analysis. Ambulatory 24‐hour BP monitoring was performed every 15 mins during daytime; every 30 mins nighttime; 24‐hour mean values, daytime, nighttime and hourly values calculated. Time points: 6 months **Systolic Blood Pressure (SBP):** Outcome measurement: Clinic measurements taken at each visit after 5 mins rest in the seated position. The mean of the last 2 of the 3 readings used for the analysis. Ambulatory 24‐hour BP monitoring was performed every 15 mins during daytime; every 30 mins nighttime; 24‐hour mean values, daytime, nighttime and hourly values calculated. Time points: 6 months **Hypertension (as reported, or SBP > 140 mmHg or DBP > 85 mmHg):** NR **Blood pressure control (achieving blood pressure threshold or 'control' as prespecified):** NR **Cardiovascular events‐stroke:** NR **Cardiovascular events‐myocardial infarction:** NR **Cardiovascular events‐other:** Outcome measurement: NR time points: duration of study period **Cardiovascular mortality:** NR **Blood potassium:** Outcome measurement: Measured in arterial blood samples drawn at the beginning and end of the intervention period. Time points: 6 months **Hyperkalaemia:** NR **Hypokalaemia:** NR **Secondary outcomes:** **All‐cause mortality:** NR **Adverse events:** NR **Antihypertensive medication use:** NR **Body Mass index (BMI):** NR **Serum creatinine:** Outcome measurement: Measured in arterial blood samples drawn at the beginning and end of the intervention period. Time points: 6 months **Albuminuria:** NR **Urinary albumin‐to‐creatinine ratio (uACR):** NR **Fasting blood glucose:** NR **Blood triglycerides:** NR **Total blood cholesterol:** NR **24‐h urinary sodium excretion:** 24‐hour urine sample. Time points: 6 months **24‐h urinary potassium excretion:** 24‐hour urine sample. Time points: 6 months	
Notes	**Funding source:** The study was supported by The Norwegian National Health Association, The Norwegian Council for Cardiovascular Disease. Pansalt was sponsored by Reformi‐Keskus Oriola Oy, Espoo, Finland and Searle Norge A/S Oslo Norway. **Authors name:** Per Omvik **Institution:** Department of Cardiology, Haukeland Hospital **Email:** Per.Omvik@uib.no **Possible conflicts of interest (for study authors):** NR **Sources used for data extraction:** Journal article with results of the trial **Trial registration details:** NR	
***Risk of bias***		
**Bias**	**Authors' judgement**	**Support for judgement**
Random sequence generation (selection bias)	Unclear risk	Insufficient information on how the randomisation sequence was generated
Allocation concealment (selection bias)	Unclear risk	No information on how the randomisation sequence was protected
Blinding of participants and personnel (performance bias) All outcomes	Low risk	The study was reported as 'double‐blind'. Intervention and control salts were pre‐numbered, according to the participants' randomisation numbers, and identically wrapped. It was not explicitly stated who were blinded, but outcomes were unlikely to have been affected by any potential lack of blinding.
Blinding of outcome assessment (detection bias) All outcomes	Low risk	The study was reported as 'double‐blind'. It was not clear whether outcome assessors were blinded; however, blood pressure measurements were taken using a standard technique and automated monitor and serum values were laboratory‐determined.
Incomplete outcome data (attrition bias) All outcomes	Low risk	Overall attrition was low, with 1/20 (5%) in the intervention and 0/20 (0%) in the control group.
Selective reporting (reporting bias)	Unclear risk	No study protocol or prospective trial registry entry available
Other bias	Low risk	None identified
Overall risk of bias	Unclear risk	Unclear risk of bias for allocation concealment; low risk of bias for incomplete outcome data

**Table:** Pan 2017

***Study characteristics***		
Methods	**Study design:** Randomised controlled trial **Study grouping:** Parallel group **Country:** Taiwan **Setting:** Intervention conducted in households; outcomes measured at 8 clinical centres **Aim of study:** To evaluate the effects of potassium‐ and magnesium‐enriched salts on the neurologic performance of stroke patients **Unit of allocation:** Adults (age 45 years and older) **Start date:** December 2009 **End date:** May 2013 **Relevant study limitations as reported by study authors:** Lack of statistical power due to small sample size; synergistic effects of magnesium and potassium may be present; large dropout rate; under‐consumption of intervention salt and collection of first morning urine samples (instead of 24‐hour urine samples) **Sample size calculation:** NR	
Participants	**Baseline Characteristics** LSSS intervention (≥ 30% KCl) *Age*: in years, mean (SD): 64.4 (9.8) *Gender*: Female, % (n/N): 42.3 (41/97) *Ethnicity/race*: Taiwanese *Smoking*: NR *Body Mass Index (BMI)*: NR *Blood pressure status*: Hypertensive, %(n/N): 56.7 (55/97) *Antihypertensive medication used*: NR *Cardiovascular disease or stroke*: Ischaemic stroke, % (n/N): 94.8 (92/97); stroke, haemorrhagic stroke, % (n/N): 5.2 (5/97); hyperlipidaemia, % (n/N): 37.1 (36/97) *Diabetes mellitus*: %, (n/N): 33.0 (32/97) *Renal impairment*: GFR < 60 mL/min, % (n/N): 0 (0/97) *Dietary potassium intake*: NR *Dietary sodium intake*: NR *Urinary potassium excretion*: NR *Urinary sodium excretion*: NR LSSS intervention (≥ 30% KCl) *Age*: in years, mean (SD): 64.7 (9.9) *Gender*: Female, % (n/N): 34.7 (33/95) *Ethnicity/race*: Taiwanese *Smoking*: NR *Body Mass Index (BMI)*: NR *Blood pressure status*: Hypertensive, %(n/N): 68.4 (62/95) *Antihypertensive medication used*: NR *Cardiovascular disease or stroke*: Ischaemic stroke, % (n/N): 93.7 (89/95); haemorrhagic stroke, % (n/N): 6.3 (6/95); hyperlipidaemia, % (n/N): 44.2 (42/95) *Diabetes mellitus*: %, (n/N): 30.5 (29/95) *Renal impairment*: GFR < 60mL/min, % (n/N): 0 (0/95) *Dietary potassium intake*: NR *Dietary sodium intake*: NR *Urinary potassium excretion*: NR *Urinary sodium excretion*: NR Control *Age*: in years, mean (SD): 64.8 (10.3) *Gender*: Female, % (n/N): 32.3 (32/99) *Ethnicity/race*: Taiwanese *Smoking*: NR *Body Mass Index (BMI)*: NR *Blood pressure status*: Hypertensive, %(n/N): 50.5 (50/99) *Antihypertensive medication used*: NR *Cardiovascular disease or stroke*: Ischaemic stroke, % (n/N): 91.9 (91/99); haemorrhagic stroke, % (n/N): 8.1 (8/99); hyperlipidaemia, % (n/N): 39.4 (39/99) *Diabetes mellitus*: %, (n/N): 40.4 (40/99) *Renal impairment*: GFR < 60mL/min, % (n/N): 0 (0/99) *Dietary potassium intake*: NR *Dietary sodium intake*: NR *Urinary potassium excretion*: NR *Urinary sodium excretion*: NR **Inclusion criteria:** Adults, aged 45 years and older, who were hospitalised for <= 1 month because of cerebral infarction or haemorrhage, with a modified Rankin scale (mRS) score of <= 4 at the time of discharge, who agreed to prepare foods with salt provided by the study **Exclusion criteria:** Patients with poor kidney function (GFR < 60 mL/min), secondary hypertension, cancer, or liver diseases, those with eating disorders; those taking potassium‐sparing medicines, or consuming salt substitutes **Pretreatment:** None reported at baseline. However, the proportion of participants with hypertension in the control group (50.5%) was lower compared to the intervention groups, especially the < 50% KCl group (68.4%). **Method of recruitment of participants:** Medical practitioners at 8 participating hospitals identified potentially eligible patients. **Informed consent obtained:** yes (written) **Clusters:** n/a **Subgroups planned/measured:** NR **Subgroups reported:** NR *Participant flow* **Assessed for eligibility:** NR **Excluded (number with reasons):** NR **Randomised:** n = 291 **Allocated to LSSS intervention/s:** n = 97 (LSSS 50% KCl); n = 95 (LSSS < 50% KCl) **Allocated to control:** n = 99 **Received allocated LSSS intervention/s:** NR **Did not receive allocated LSSS intervention/s:** NR **Lost to follow‐up (LSSS intervention group):** At 3 months: n = 21 (LSSS 50% KCl) ‐ Reasons: Moved away (n = 2), hospital transfer (n = 1), left study (n = 15), lost to follow‐up (n = 3)n = 20; (LSSS < 50% KCl)‐ Reasons: Suicidal death (n = 1), hospital transfer (n = 2), left study (n = 16), lost to follow‐up (n = 1) At 6 months: n = 14 (LSSS 50% KCl)‐ Reasons: Hospital transfer (n = 3), left study (n = 9), lost to follow‐up (n = 2) n = 11; (LSSS < 50% KCl) ‐ Reasons: Hospital transfer (n = 1), left study (n = 8), lost to follow‐up (n = 2) **Discontinued intervention (LSSS intervention group):** At 3 months: n = 15 (LSSS 50% KCl) n = 16 (LSSS < 50% KCl) At 6 months: n = 9 (LSSS 50% KCl) n = 8 (LSSS < 50% KCl) **Analysed (LSSS intervention group):** n = 97 (LSSS 50% KCl); n = 95 (LSSS < 50% KCl) ‐ ITT analysis was performed (the last‐observation‐carried‐forward method) **Excluded from analysis (LSSS intervention group):** NR **Received allocated control:** NR **Did not receive allocated control:** NR **Lost to follow‐up (control group):** At 3 months: n = 15 Reasons ‐ Suicidal death (n = 1), hospital transfer (n = 1), left study (n = 12), lost to follow‐up (n = 1) At 6 months: n = 13 Reasons ‐ Hospital transfer (n = 1), left study (n = 10), lost to follow‐up (n = 2) **Discontinued intervention (control group):** At 3 months: n = 12 At 6 months: n = 10 **Analysed (control group):** n = 99 ‐ ITT analysis was performed (the last‐observation‐carried‐forward method) **Excluded from analysis (control group):** NR	
Interventions	**Intervention Characteristics** LSSS intervention (50% KCl) *Theoretical basis*: Provision of an adequate intake of potassium to stroke patients, for the maintenance of neurologic and cardiovascular health *Description*: K salt (NaCl 50%; KCl 50%). Approx. consumption of 6.5g 'K' salt per person/day (1278 mg or 55.6 mmol Na; 1701 mg or 43.6 mmol K) *LSSS category*: ≥ 30% KCl *Contains fortificant*: NR *Delivery*: discretionary use *Duration of run‐in period*: none *Duration of active intervention*: 6 months *Duration of follow‐up (as reported)*: none *Total duration of study (as reported)*: 6 months *Timing*: daily household use *Implementation*: 1 kg salt was provided at study entry and replenished at the 3‐month outpatient visit. *Providers*: Medical practitioners were responsible for salt distribution; manufacturer NR *Co‐interventions*: NR *Resource requirements*: NR *Integrity of delivery*: Salt consumption, in grams per 3 months, mean (SD): 361.5 (301.5); n = 71; 328.5 (220.2); n = 60 LSSS intervention (< 50% KCl) *Theoretical basis*: Provision of an adequate intake of potassium and magnesium to stroke patients, for the maintenance of neurologic and cardiovascular health *Description*: 'K/Mg' salt (42.85% NaCl, 42.85% KCl, 14.3% MgSO_4_). Approx. consumption of 6.5g 'K/Mg' salt per person/day (2785 mg or 47.6 mmol Na; 2785 mg or 44.9 mmol K and 929.5 mg (3.8 mmol)) *LSSS category*: ≥ 30% KCl *Contains fortificant*: NR *Delivery*: discretionary use *Duration of run‐in period*: none *Duration of active intervention*: 6 months *Duration of follow‐up (as reported)*: none *Total duration of study (as reported)*: 6 months *Timing*: daily household use *Implementation*: 1 kg salt was provided at study entry and replenished at the 3‐month outpatient visit. *Providers*: Medical practitioners were responsible for salt distribution; manufacturer NR *Co‐interventions*: NR *Resource requirements*: NR *Integrity of delivery*: Salt consumption, in grams per 3 months, mean (SD): 328.2 (238.3); n = 72; 350.7 (346.0); n = 59 Control *Theoretical basis*: To enable comparison of LSSS intervention with standard practice *Description*: Regular salt. Approx. consumption of 6.5 g salt per person/day (2555 mg Na or 111 mmol Na) *Contains fortificant*: NR *Delivery*: discretionary use *Duration of run‐in period*: none *Duration of active intervention*: 6 months *Duration of follow‐up (as reported)*: none *Total duration of study (as reported)*: 6 months *Timing*: daily household use *Implementation*: 1 kg salt was provided at study entry and replenished at the 3‐month outpatient visit. *Providers*: Medical practitioners were responsible for salt distribution; manufacturer NR *Co‐interventions*: NR *Resource requirements*: NR *Integrity of delivery*: Salt consumption, in grams per 3 months, mean (SD): 425.2 (344.4); n = 81; 332.4 (223.3); n = 68	
Outcomes	**Primary outcomes: ** **Diastolic Blood Pressure (DBP):** NR **Systolic Blood Pressure (SBP):** NR **Hypertension (as reported, or SBP > 140 mmHg or DBP > 85 mmHg):** NR **Blood pressure control (achieving blood pressure threshold or 'control' as prespecified):** NR **Cardiovascular events‐stroke:** Outcome measurement: NR; time points: 3, 6 months **Cardiovascular events‐myocardial infarction:** NR **Cardiovascular events‐other:** NR **Cardiovascular mortality:** NR **Blood potassium:** NR **Hyperkalaemia:** NR **Hypokalaemia:** NR **Secondary outcomes** **All‐cause mortality:** Outcome measurement: NR; time points: 3, 6 months **Adverse events:** Outcome measurement (serious AEs): NR; time points: duration of the study **Antihypertensive medication use:** NR **Body Mass index (BMI):** NR **Serum creatinine:** NR **Albuminuria:** NR **Urinary albumin‐to‐creatinine ratio (uACR):** NR **Fasting blood glucose:** NR **Blood triglycerides:** NR **Total blood cholesterol:** NR **24‐h urinary sodium excretion:** NR **24‐h urinary potassium excretion:** NR	
Notes	**Funding source:** Supported by grants from the Institute of Biomedical Sciences, Academia Sinica, and from the Ministry of Health and Welfare (DOH098‐TD‐F‐113‐098030). **Authors name:** Wen‐Harn Pan **Institution:** Institute of Biomedical Sciences, Academia Sinica, Taipei, Taiwan **Email:** pan@ibms.sinica.edu.tw **Possible conflicts of interest (for study authors):** None. **Sources used for data extraction:** Journal article with results of the trial **Trial registration details:** NCT02910427	
***Risk of bias***		
**Bias**	**Authors' judgement**	**Support for judgement**
Random sequence generation (selection bias)	Low risk	Computerised block randomisation sequence generation. A stratified randomisation method was used according to a clinical history of ischaemic or haemorrhagic stroke, diabetes or no diabetes, and the use of magnesium‐containing medication or not.
Allocation concealment (selection bias)	Low risk	Salts were labelled by numbers 1, 2, 3 during manufacturing. Allocation was performed by software once the participant's details were entered.
Blinding of participants and personnel (performance bias) All outcomes	Low risk	The study authors reported that participants and personnel, such as the medical practitioners involved in the salt distribution, were blinded.
Blinding of outcome assessment (detection bias) All outcomes	Low risk	The medical practitioners performing the patient evaluations were blinded.
Incomplete outcome data (attrition bias) All outcomes	Low risk	Incomplete data reported at 6 months were 34% (33/97)(LSSS 50% KCl) and 34.7% (33/95) (LSSS < 50% KCl), compared to 28.3% (28/99) in the control group. The study authors stated that no significant difference was found in baseline characteristics between participants who remained in the study at month 3 and at month 6, compared to those who did not. The study authors conducted an ITT analysis (last‐observation‐carried‐forward).
Selective reporting (reporting bias)	Low risk	Primary outcomes reported according to those pre‐specified in the study protocol (NCT02910427)
Other bias	Low risk	None identified
Overall risk of bias	Low risk	Low risk of bias for allocation concealment and incomplete outcome data

**Table:** Pereira 2005

***Study characteristics***		
Methods	**Study design:** Randomised controlled trial **Study grouping:** Parallel group **Country:** Brazil **Setting:** Intervention conducted in households in São Paulo; outcomes measured at the outpatient clinic of a federal university **Aim of study:** To evaluate the effect of potassium supplementation of table salt, weight reduction through caloric restriction as well as increased physical activity on blood pressure and insulin resistance in people who are overweight, have hypertension and are treated with diuretics **Unit of allocation:** adults (aged 18 years or older) **Start date:** NR **End date:** NR **Relevant study limitations as reported by study authors:** Limited adherence to the LSSS resulted in sodium chloride replacement of approximately 30%, presumably as some participants did not eat all their meals at home. **Sample size calculation:** NR	
Participants	** Baseline Characteristics** LSSS intervention *Age:* in years, mean (SD): 45.4 (13.2) *Gender:* NR *Ethnicity/race:* "5 white/4 black" *Smoking:* NR *Body Mass Index (BMI)*: in kg/m^2^, mean (SD): 32.5 (13.2) *Blood pressure status: *Participants with hypertension, % (n/N): 100 (12/12); SBP in mmHg, mean (SD): 136.2 (6); DBP in mmHg, mean (SD): 93.80 (4.3) *Antihypertensive medication used:* On chlorthalidone 25 mg, % (n/N): 67 (8/12); on hydrochlorothiazide 25 mg, % (n/N): 33 (4/12) *Cardiovascular disease or stroke: *NR *Diabetes mellitus:* %, (n/N): 0 (0/12) *Renal impairment:* Serum creatinine in µmol/L, mean (SD): 81.35 (8.84) *Dietary potassium intake:* NR *Dietary sodium intake: *NR *Urinary potassium excretion: *in mmol/g creatinine, mean (SD): 38.80 (18.60) *Urinary sodium excretion:* in mmol/g creatinine, mean (SD): 159.90 (62.40) Control *Age:* in years, mean (SD): 50.1 (8.1) *Gender:* NR *Ethnicity/race:* "5 white/4 brown" *Smoking:* NR *Body Mass Index (BMI):* in kg/m^2^, mean (SD): 30.2 (2.7) *Blood pressure status: *Participants with hypertension, % (n/N): 100 (10/10); SBP in mmHg, mean (SD): 139.6 (11.9); DBP in mmHg, mean (SD): 92.3 (5.1) *Antihypertensive medication used: *On chlorthalidone 25 mg, % (n/N): 70 (7/10); on hydrochlorothiazide 25 mg, % (n/N): 30 (3/10) *Cardiovascular disease or stroke:* NR *Diabetes mellitus:*% (n/N): 0 (0/10) *Renal impairment: *Serum creatinine in µmol/L, mean (SD): 80.46 (8.84) *Dietary potassium intake:* NR *Dietary sodium intake:* NR *Urinary potassium excretion:* in mmol/g creatinine, mean (SD): 48.20 (22.50) *Urinary sodium excretion:* in mmol/g creatinine, mean (SD): 171.50 (59.30) *Overall* *Age:* in years, mean (SD; range): 47.5 (11.2; 23 to 67) *Gender:* Female, % (n/N): 86 (24/28) *Ethnicity/race:* "10 white/4 black/4 brown" *Smoking:* NR *Body Mass Index (BMI): *NR *Blood pressure status: *Hypertensive, % (n/N): 100 (28/28) *Antihypertensive medication used: *On chlorthalidone 25 mg, % (n/N): 68 (15/22); on hydrochlorothiazide 25 mg, % (n/N): 32 (7/22) *Cardiovascular disease or stroke:* NR *Diabetes mellitus: *Excluded *Renal impairment:* NR *Dietary potassium intake: *NR *Dietary sodium intake:* NR *Urinary potassium excretion: *NR *Urinary sodium excretion: *NR **Inclusion criteria:** Men and women who are overweight or living with obesity, and have hypertension, aged 20 to 70 years; exclusively using diuretics (hydrochlorothiazide or chlorthalidone) for at least three weeks; systolic blood pressure > 140 mmHg and < 160 mmHg and diastolic blood pressure > 90 mmHg and < 105 mmHg; with BMI of 27 kg/m^2^ calculated during medical consultation; and central body fat distribution, defined as waist‐to‐hip ratio of 0.85 for women and 0.95 for men **Exclusion criteria: **Participants with clinical or laboratory evidence of heart disease, stroke blood dyscrasias, kidney or liver disease, diabetes mellitus; serum potassium concentrations of 5.0 or < 3.0 mEq/L; or use of any antihypertensives other than diuretics **Pretreatment:** None **Method of recruitment of participants:** Patients were recruited from a hypertension outpatient clinic and diabetes hospital which they attended. **Informed consent obtained:** Yes, written **Clusters:** n/a **Subgroups planned/measured: **NR **Subgroups reported: **NR **Assessed for eligibility: **NR **Excluded (number with reasons): **NR **Randomised:** n = 28 **Allocated to LSSS intervention(s):** n = 15 **Allocated to control: **n = 13 **Received allocated LSSS intervention(s): **NR **Did not receive allocated LSSS intervention(s):** NR **Lost to follow‐up (LSSS intervention group):** Total group n = 4 (needed to change medication); distribution by trial arm not reported **Discontinued intervention (LSSS intervention group): **Total group n = 4 (needed to change medication); distribution by trial arm not reported **Analysed (LSSS intervention group): **n = 12 **Excluded from analysis (LSSS intervention group):** Total group n = 2 (lack of adherence to treatment); distribution by trial arm not reported **Received allocated control: **NR **Did not receive allocated control:** NR **Lost to follow‐up (control group):** Total group n = 4 (needed to change medication); distribution by trial arm not reported **Discontinued intervention (control group): **Total group n = 4 (needed to change medication); distribution by trial arm not reported **Analysed (control group):** n = 10 **Excluded from analysis (control group):** Total group n = 2 (lack of adherence to treatment); distribution by trial arm not reported	
Interventions	**Intervention Characteristics** LSSS intervention *Theoretical basis:* Reduction of dietary sodium and increases in potassium intake may lower blood pressure, while the latter may also increase plasma potassium and decrease mortality from stroke and heart disease. Increases in serum potassium have been linked to decreased blood pressure and decreased mortality from stroke cerebral and heart disease via reduced neural adrenergic activity, decreased levels of plasma renin, inhibition of the formation of free radicals and increased enzyme activity Na + / K + ‐ATPase, increasing cell uptake of potassium and reducing intracellular sodium. *Description:* Potassium chloride salt (50% NaCl; 50% KCl) *LSSS category:* ≥ 30% KCl *Contains fortificant:* NR *Delivery:* discretionary use *Duration of run‐in period:* none *Duration of active intervention:* 12 weeks *Duration of follow‐up (as reported):* none *Total duration of study (as reported):* 12 weeks *Timing:* LSSS to be used throughout the study as table and cooking salt *Implementation:* Packs, similar in appearance to control salt, of 1 kg LSSS were used; frequency of distribution NR *Providers:* NR *Co‐interventions:* Individualised hypocaloric diet using body composition to calculate basal metabolic rate with an activity factor to determine energy needs with 1000 Kcal deficit (min prescription 1200 kCal); increased physical activity advised *Resource requirements:* NR *Integrity of delivery:* Urine collections were done at all study visits to assess adherence to the salt intervention. Participants were asked to prepare all meals at home and to take home‐cooked meals to work. Two participants were excluded from the total group due to a lack of adherence. Control *Theoretical basis:* To enable comparison of LSSS intervention with standard practice. Description: normal salt (100% NaCl) *LSSS category:* n/a *Contains fortificant:* NR *Delivery:* discretionary use *Duration of run‐in period:* none *Duration of active intervention:* 12 weeks *Duration of follow‐up (as reported):* none *Total duration of study (as reported):* 12 weeks *Timing:* Normal salt to be used throughout the study as table and cooking salt *Implementation*: Packs, similar in appearance to LSSS, of 1 kg control salt were used; frequency of distribution NR *Providers:* NR *Co‐interventions:* Individualised hypocaloric diet using body composition to calculate basal metabolic rate with an activity factor to determine energy needs with 1000 Kcal deficit (min prescription 1200 kCal) ; increased physical activity advised *Resource requirements:* NR *Integrity of delivery:* Urine collections were done at all study visits to assess adherence to the salt intervention. Participants were asked to prepare all meals at home and to take home‐cooked meals to work. Two participants were excluded from the total group due to a lack of adherence.	
Outcomes	**Primary outcomes:** **Diastolic Blood Pressure (DBP):** Outcome measurement: Clinic‐based measurements performed using the auscultatory method after 5 mins rest, with patient in seating position; 24‐hour ambulatory BP monitoring with automatic device (MAPA); average calculated for 24 hours; daytime and nighttime. Time points: 12 weeks **Systolic Blood Pressure (SBP):** Outcome measurement: Clinic‐based measurements performed using the auscultatory method after 5 mins rest, with patient in seating position; 24‐hour ambulatory BP monitoring with automatic device (MAPA); average calculated for 24 hours; daytime and nighttime. Time points: 12 weeks **Hypertension (as reported, or SBP > 140 mmHg or DBP > 85 mmHg):** NR **Blood pressure control (achieving blood pressure threshold or 'control' as prespecified):** NR **Cardiovascular events‐stroke:** NR **Cardiovascular events‐myocardial infarction:** NR **Cardiovascular events‐other:** NR **Cardiovascular mortality:** NR **Blood potassium:** Outcome measurement: Determined at 120 minutes following glucose overload. Time points: 12 weeks **Hyperkalaemia:** NR **Hypokalaemia:** Outcome measurement: NR time points: duration of study **Secondary outcomes:** **All‐cause mortality:** NR **Adverse events:** NR **Antihypertensive medication use:** NR **Body Mass index (BMI):** Outcome measurement: weight with body mass index calculation. Time points: 12 weeks **Serum creatinine:** Outcome measurement: Determined at 120 minutes following glucose overload. Time points: 12 weeks **Albuminuria:** NR **Urinary albumin‐to‐creatinine ratio (uACR):** NR **Fasting blood glucose:** NR **Blood triglycerides:** Outcome measurement: Determined at 120 minutes following glucose overload. Time points: 12 weeks **Total blood cholesterol:** Outcome measurement: Determined at 120 minutes following glucose overload. Time points: 12 weeks **24‐h urinary sodium excretion:** NR **24‐h urinary potassium excretion:** NR	
Notes	**Funding source:** NR **Authors name:** Maria Alice de Gouveia Pereira **Institution:** Disciplines of Nutrition and Metabolism, Kidney and Hypertension Hospital, Federal University of São Paulo **Email:** alicegouveia@ig.com.br **Possible conflicts of interest (for study authors):** NR **Sources used for data extraction:** Journal article with results of the trial **Trial registration details:** NR	
***Risk of bias***		
**Bias**	**Authors' judgement**	**Support for judgement**
Random sequence generation (selection bias)	Unclear risk	Insufficient information on how the randomisation sequence was generated
Allocation concealment (selection bias)	Unclear risk	No information on how the randomisation sequence was protected
Blinding of participants and personnel (performance bias) All outcomes	Low risk	The authors reported that the study was 'double‐blind', and intervention and control salts were packaged to look the same. It was not explicitly stated who was blinded, but outcomes were unlikely to have been affected by any potential lack of blinding.
Blinding of outcome assessment (detection bias) All outcomes	Unclear risk	The study reported 'double‐blinding', but it was not clear whether outcome assessors were blinded. Blood pressure was determined using the auscultatory method, which may be more prone to detection bias than automated techniques. Other outcomes were laboratory‐determined measures unlikely to be affected by a lack of blinding.
Incomplete outcome data (attrition bias) All outcomes	Unclear risk	Overall attrition was 3/15 (20%) in the intervention and 3/13 (23%) in the control group. Reasons for attrition (exclusion due to non‐adherence and change in antihypertensive medication) were not reported per trial arm.
Selective reporting (reporting bias)	Unclear risk	No study protocol or prospective trial registry entry available
Other bias	Low risk	None identified
Overall risk of bias	Unclear risk	Unclear risk of bias for allocation concealment and incomplete outcome data

**Table:** Sarkkinen 2011

***Study characteristics***		
Methods	**Study design:** Randomised controlled trial **Study grouping:** Parallel group **Country:** Finland **Setting:** Intervention conducted in households in Kuopio; outcomes measured at Food and Health Research Centre, University of Eastern Finland **Aim of study:** To determine the feasibility of replacing sodium chloride with a novel mineral salt (50% sodium chloride, 25% potassium chloride and 25% magnesium ammonium potassium chloride hydrate); and the effect of replacement on blood pressure **Unit of allocation:** adults (aged 18 years or older) **Start date:** NR **End date:** NR **Relevant study limitations as reported by study authors:** Small sample size **Sample size calculation:** NR	
Participants	**Baseline Characteristics** LSSS intervention *Age*: in years, mean (SD): 57 (12) *Gender*: Female, % (n/N): 59 (13/22) *Ethnicity/race*: European *Smoking*: Smokers, % (n/N): 14 (3/22) *Body Mass Index (BMI)*: in kg/m^2^, mean (SD): 28 (3) *Blood pressure status*: Mildly hypertensive (SBP 130 to 159; DBP 85 to 99), %: 100 (25/25) *Antihypertensive medication used*: Use of antihypertensive medication, % (n/N): 0 (0/22) *Cardiovascular disease or stroke*: Active heart disease, % (n/N): 0 (0/22) *Diabetes mellitus*: Type 1 and type 2 DM, % (n/N): 0 (0/22) *Renal impairment*: Abnormal kidney function, % (n/N): 0 (0/22) *Dietary potassium intake*: NR *Dietary sodium intake*: in points according to Finnish Heart association, mean (SD): 11.7 (2.5) *Urinary potassium excretion*: mmol/24 h, mean (SD): 79 (31) *Urinary sodium excretion*: mmol/24 h, mean (SD): 130 (47) Control *Age*: in years, mean (SD): 54 (11) *Gender*: Female, % (n/N): 39 (9/23) *Ethnicity/race*: European *Smoking*: Smokers, % (n/N): 13 (3/23) *Body Mass Index (BMI)*: in kg/m^2^, mean (SD): 28 (3) *Blood pressure status*: Mildly hypertensive (SBP 130 to 159; DBP 85 to 99), %: 100 (25/25) *Antihypertensive medication used*: Use of antihypertensive medication, % (n/N): 0 (0/25) *Cardiovascular disease or stroke*: Active heart disease, % (n/N): 0 (0/23) *Diabetes mellitus*: Type 1 and type 2 DM, % (n/N): 0 (0/23) *Renal impairment*: Abnormal kidney function, % (n/N): 0 (0/23) *Dietary potassium intake*: NR *Dietary sodium intake*: in points according to Finnish Heart association, mean (SD): 11.9 (1.4) *Urinary potassium excretion*: mmol/24 h, mean (SD): 82 (22) *Urinary sodium excretion*: mmol/24 h, mean (SD): 151 (44) **Inclusion criteria:** Subjects aged between 25 and 75 years; with systolic blood pressure between 130 and 159 mmHg and/or diastolic blood pressure between 85 and 99 mmHg; with BMI between 23 and 40 kg/m^2^, and stable body weight **Exclusion criteria:** Subjects on antihypertensive medication, nonsteroidal anti‐inflammatory drugs, cyclosporine or tacrolimus; with secondary hypertension, type 1 or 2 diabetes, history of active heart disease or cancer, abnormal electrolyte concentrations, proteinuria; with abnormal liver, kidney or thyroid function; currently following a low salt diet (six points or fewer on the salt intake test of the Finnish Heart Association); abusing drugs or alcohol; who are pregnant, or lactating **Pretreatment:** None **Method of recruitment of participants:** Recruitment of subjects was done through announcements in local newspapers and from the volunteer register of Oy Foodfiles (affiliation of the first author), after which potential participants were screened by telephone. **Informed consent obtained:** Yes, written **Clusters:** n/a **Subgroups planned/measured:** NR **Subgroups reported:** NR *Participant flow* **Assessed for eligibility:** n = 168 **Excluded (number with reasons):** n = 118 (reasons NR) **Randomised:** n = 50 **Allocated to LSSS intervention(s):** n = 25 **Allocated to control:** n = 25 **Received allocated LSSS intervention(s):** NR **Did not receive allocated LSSS intervention(s):** NR **Lost to follow‐up (LSSS intervention group):** n = 3 (reasons NR) **Discontinued intervention (LSSS intervention group):** NR **Analysed (LSSS intervention group):** n = 22 **Excluded from analysis (LSSS intervention group):** NR **Received allocated control:** NR **Did not receive allocated control:** NR **Lost to follow‐up (control group):** n = 2 (reasons NR)** ** **Discontinued intervention (control group):** NR **Analysed (control group):** n = 23 **Excluded from analysis (control group):** NR	
Interventions	**Intervention Characteristics** LSSS intervention *Theoretical basis*: Restriction in dietary salt lowers blood pressure. Substituting potassium and/or magnesium salts may increase the feasibility of salt restriction and reduce blood pressure more than restriction of sodium alone. *Description*: "Smart Salt" SMS50 (50% NaCl; 25% KCl; 25% Mg salt) *LSSS category*: < 30% KCl *Contains fortificant*: NR *Delivery*: Non‐discretionary and discretionary use *Duration of run‐in period*: 4 weeks on habitual diet *Duration of active intervention*: 8 weeks *Duration of follow‐up (as reported)*: none *Total duration of study (as reported)*: 8 weeks *Timing*: The daily amount of test foods in the diet was based on national dietary data in Finland (FinDiet 2007), and LSSS was used throughout the study for cooking, baking and at the table. *Implementation*: Test foods provided were industrially processed main dishes (casseroles, soups, pastas, pizza and minced meat dishes), bread (70% rye bread and 30% multigrain), frankfurters sausage/cold cuts and Edam cheese which replace 60% of sodium. The analysed concentration of sodium (expressed as NaCl) in the test foods varied between 0.38‐1.41% in SmartSalt foods. *Providers*: Manufactured by SmartSalt, Inc., California, USA *Co‐interventions*: Participants instructed to avoid salt‐rich products (e.g. salty snacks, soy sauce, olives, salt‐rich cheeses, stock cubes, salty and smoked fish), products containing bioactive peptides (Evolus), liquorice, ammonium chloride products and any food supplements that may affect BP. *Resource requirements*: NR *Integrity of delivery*: The sodium chloride reduction was intended to be reduced by 3.1 to 5.6 grams (approximately 1.2 to 2.2 grams of sodium), depending on energy intake and dietary habits. Individual diaries with use of test products were kept by participants. Good compliance was reported by study authors. Control *Theoretical basis*: To enable comparison of LSSS intervention with standard practice *Description*: Regular salt (100% NaCl) *Contains fortificant*: NR *Delivery*: Non‐discretionary and discretionary use *Duration of run‐in period*: 4 weeks on habitual diet *Duration of active intervention*: 8 weeks *Duration of follow‐up (as reported)*: none *Total duration of study (as reported)*: 8 weeks *Timing*: The daily amount of test foods in the diet was based on national dietary data in Finland (FinDiet 2007), and regular salt was used throughout the study for cooking, baking and at the table. *Implementation*: Test foods provided were industrially processed main dishes (casseroles, soups, pastas, pizza and minced meat dishes), bread (70% rye bread and 30% multigrain), frankfurters sausage/cold cuts and Edam cheese. The analysed concentration of sodium (expressed as NaCl) in the test foods varied between 0.64‐2.03% in regular salt foods. *Providers*: Manufactured by Akzo Nobel Salt, The Netherlands *Co‐interventions*: Participants instructed to avoid salt‐rich products (e.g. salty snacks, soy sauce, olives, salt‐rich cheeses, stock cubes, salty and smoked fish), products containing bioactive peptides (Evolus), liquorice, ammonium chloride products and any food supplements that may affect BP. *Resource requirements*: NR *Integrity of delivery*: Individual diaries with use of test products were kept by participants. Good compliance was reported by study authors.	
Outcomes	**Primary outcomes: ** **Diastolic Blood Pressure (DBP):** Outcome measurement: Measurements taken with an automatic sphygmomanometer (after 10 mins rest) in sitting position. Three measurements with 2 minutes in between taken during the morning. The last 2 measurements were averaged. Time points: 3, 6, 8 weeks **Systolic Blood Pressure (SBP):** Outcome measurement: Measurements taken with an automatic sphygmomanometer (after 10 mins rest) in sitting position. Three measurements with 2 minutes in between taken during the morning. The last 2 measurements were averaged. Time points: 3, 6, 8 weeks **Hypertension (as reported, or SBP > 140 mmHg or DBP > 85 mmHg):** NR **Blood pressure control (achieving blood pressure threshold or 'control' as prespecified):** NR **Cardiovascular events‐stroke:** NR **Cardiovascular events‐myocardial infarction:** NR **Cardiovascular events‐other:** Cardiovascular symptoms; outcome measurement: Subjects recorded any adverse events in their daily diary. Time points: duration of the study **Cardiovascular mortality:** NR **Blood potassium:** Outcome measurement: Blood samples were taken following 10 to 12 hours of overnight fasting, and were analysed using standard methods, ion‐specific electrode analysis for serum potassium, at ISLAB laboratories. Time points: 8 weeks **Hyperkalaemia:** NR **Hypokalaemia:** NR **Secondary outcomes: ** **All‐cause mortality:** NR **Adverse events:** Respiratory, abdominal/intestinal symptoms; outcome measurement: Subjects recorded any adverse events in their daily diary. Time points: duration of the study **Antihypertensive medication use:** NR **Body Mass index (BMI):** Outcome measurement: Calculated from standard equation using weight determined by calibrated digital scale and height measured by telescoping measuring rod, to the nearest crossed half a centimetre. Time points: 8 weeks **Serum creatinine:** NR **Albuminuria:** NR **Urinary albumin‐to‐creatinine ratio (uACR):** NR **Fasting blood glucose:** NR **Blood triglycerides:** NR **Total blood cholesterol:** NR **24‐h urinary sodium excretion:** 24‐hour urine sample. Time points: 8 weeks **24‐h urinary potassium excretion:** 24‐hour urine sample. Time points: 8 weeks	
Notes	**Funding source:** The study was supported by SmartSalt® Inc, California, USA. **Authors name:** Essi S. Sarkkinen **Institution:** Oy Foodfiles Ltd, CRO in the field of nutrition **Email:** essi.sarkkinen@foodfiles.com **Possible conflicts of interest (for study authors):** "The authors declare that they have no competing interests." **Sources used for data extraction:** Journal article with results of the trial; non‐commercial trial registry record **Trial registration details:** ISRCTN01739816	
***Risk of bias***		
**Bias**	**Authors' judgement**	**Support for judgement**
Random sequence generation (selection bias)	Unclear risk	Insufficient information on how the randomisation sequence was generated
Allocation concealment (selection bias)	Unclear risk	Insufficient information on how the randomisation sequence was protected
Blinding of participants and personnel (performance bias) All outcomes	Low risk	The study was reported as 'double‐blind', but it was not clear whether participants and personnel were blinded (outcome assessors were reported as blinded). Given that the intervention involved test foods in addition to salt intervention, leading to minimal potential lifestyle modification, it was unlikely that outcomes of interest would be affected by a lack of blinding.
Blinding of outcome assessment (detection bias) All outcomes	Low risk	It was reported that the study nurse was unaware of treatment allocation.
Incomplete outcome data (attrition bias) All outcomes	Low risk	Overall attrition was 3/25 (12%) in the intervention and 2/25 (8%) in the control group, with no reasons provided.
Selective reporting (reporting bias)	Unclear risk	No study protocol or prospective trial registry entry available
Other bias	Low risk	None identified
Overall risk of bias	Unclear risk	Unclear risk of bias for allocation concealment; low risk of bias for incomplete outcome data

**Table:** Suppa 1988

***Study characteristics***		
Methods	**Study design:** Randomised controlled trial **Study grouping:** Parallel group **Country:** Italy **Setting:** Intervention conducted in households; outcomes measured at 31 clinical centres **Aim of study:** To evaluate the effects of a low‐sodium/high potassium salt as an additional treatment in patients with mild and moderate hypertension on beta‐blockers **Unit of allocation:** adults (aged 18 years or older) **Start date:** NR **End date:** NR **Relevant study limitations as reported by study authors:** NR **Sample size calculation:** NR	
Participants	**Baseline Characteristics** LSSS intervention *Age*: in years, mean (SD): 47.1 (9.8) *Gender*: Female, % (n/N): 35.6 (58/163) *Ethnicity/race*: European *Smoking*: NR *Body Mass Index (BMI)*: NR *Blood pressure status*: WHO stage I hypertension, % (n/N): 65 (106/163); stage II hypertension, % (n/N): 35 (57/163) *Antihypertensive medication used*: β‐blocker monotherapy (metoprolol), % (n/N): 100 (163/163) *Cardiovascular disease or stroke*: NR *Diabetes mellitus*: NR *Renal impairment*: Serum creatinine >= 1.5 mg/dL (133 µmol/L), % (n/N): 0 (0/163) *Dietary potassium intake*: NR *Dietary sodium intake*: NR *Urinary potassium excretion*: in mmol/24‐h, mean (SD): 65.5 (20.6) *Urinary sodium excretion*: in mmol/24‐h, mean (SD): 205.5 (76.9) Control *Age*: in years, mean (SD): 47.8 (10.1) *Gender*: Female, % (n/N): 39 (62/159) *Ethnicity/race*: European *Smoking*: NR *Body Mass Index (BMI)*: NR *Blood pressure status*: WHO stage I hypertension, % (n/N): 65.4 (104/159); stage II hypertension, % (n/N): 34.6 (55/159) *Antihypertensive medication used*: β‐blocker monotherapy (metoprolol), % (n/N): 100 (159/159) *Cardiovascular disease or stroke*: NR *Diabetes mellitus*: NR *Renal impairment*: Serum creatinine >= 1.5 mg/dL (133 µmol/L), % (n/N): 0 (0/159) *Dietary potassium intake*: NR *Dietary sodium intake*: NR *Urinary potassium excretion*: in mmol/24‐h, mean (SD): 67.4 (21.8) *Urinary sodium excretion*: in mmol/24‐h, mean (SD): 211.1 (90.6 **Inclusion criteria:** Participants with supine diastolic BP greater or equal to 95 mmHg only taking metoprolol 200 mg daily **Exclusion criteria:** Patients with contraindications for β‐blockers; secondary hypertension; renal failure (serum creatinine >= 1.5 mg/dL or 133 µmol/L; eGFR females, mean age 47 years: 41mL/min; males, mean age 47 years: 54 mL/min) or other major diseases, and women of childbearing potential **Pretreatment:** None **Method of recruitment of participants:** NR **Informed consent obtained:** NR **Clusters:** n/a **Subgroups planned/measured:** NR **Subgroups reported:** NR *Participant flow* **Assessed for eligibility:** n = 358 **Excluded (number with reasons):** n = 36 (n = 6 due to undesirable side effects such as asthenia, insomnia, hypotension and headaches, n = 4 due to severe hypertension, n = 26 for poor compliance mainly to collecting urine samples) **Randomised:** n = 322 **Allocated to LSSS intervention(s):** n = 163 **Allocated to control:** n = 159 **Received allocated LSSS intervention(s):** NR **Did not receive allocated LSSS intervention(s):** NR **Lost to follow‐up (LSSS intervention group):** n = 0 **Discontinued intervention (LSSS intervention group):** n = 0 **Analysed (LSSS intervention group):** n = 163 **Excluded from analysis (LSSS intervention group):** n = 0 (DBP; SBP) n = 50; (urinary sodium and potassium): Study authors only analysed data from participants if 3 urine samples were correctly collected. **Received allocated control:** NR **Did not receive allocated control:** NR **Lost to follow‐up (control group):** n = 0 **Discontinued intervention (control group):** n = 0 **Analysed (control group):** n = 159 **Excluded from analysis (control group):** n = 0 (DBP, SBP) n = 51 (urinary sodium and potassium): Study authors only analysed data from participants who collected 3 urine samples correctly.	
Interventions	**Intervention Characteristics** LSSS intervention *Theoretical basis*: Increased potassium salt intake may exert antihypertensive effects by means of vasodilation, natriuresis, decreased sympathetic tone and stimulation of Na/K pump activation. *Description*: Dietary salt (Novasal) (NaCl 50%, KCl 25% and K3C6H5 O7 15%): 4 g (34 mmol Na; 19.3 mmol K) *LSSS category*: < 30% KCl *Contains fortificant*: NR *Delivery*: Discretionary use *Duration of run‐in period*: 6 weeks (2‐week washout period; 4‐week period of β‐blocker monotherapy) *Duration of active intervention*: 4 weeks *Duration of follow‐up (as reported)*: none *Total duration of study (as reported)*: 4 weeks *Timing*: 2 g twice daily at lunch and dinner, used at the table *Implementation*: identical 2 g packets *Providers*: NR *Co‐interventions*: Metoprolol 200 mg daily *Resource requirements*: NR *Integrity of delivery*: 'Common' salt used in cooking; dietary habits remained unchanged. The number of residual salt packets and metoprolol tablets were counted at each visit. Number of salt packets consumed per day: NR; pill counts (metoprolol tablets): NR Control *Theoretical basis*: To enable comparison of LSSS intervention with standard practice *Description*: 'Common' salt (100% NaCl): 4 g (174 mmol Na) *Contains fortificant*: NR *Delivery*: Discretionary use *Duration of run‐in period*: 6 weeks (2‐week washout period; 4‐week period of β‐blocker monotherapy) *Duration of active intervention*: 4 weeks *Duration of follow‐up (as reported)*: none *Total duration of study (as reported)*: 4 weeks *Timing*: 2 g twice daily at lunch and dinner used at the table *Implementation*: identical 2 g packets *Providers*: NR *Co‐interventions*: Metoprolol 200 mg daily *Resource requirements*: NR *Integrity of delivery*: 'Common' salt used in cooking; dietary habits remained unchanged. The number of residual salt packets and metoprolol tablets were counted at each visit. Number of salt packets consumed per day: NR; pill counts (metoprolol tablets): NR	
Outcomes	Outcomes **Primary outcomes: ** **Diastolic Blood Pressure (DBP):** Outcome measurement: measurement with conventional mercury sphygmomanometer according to WHO guidelines. Time point: supine DBP, standing DBP 2, 4 weeks **Systolic Blood Pressure (SBP):** Outcome measurement: measurement with conventional mercury sphygmomanometer according to WHO guidelines. Time point: supine, standing SBP at 2, 4 weeks **Hypertension (as reported, or SBP > 140 mmHg or DBP > 85 mmHg):** NR **Blood pressure control (achieving blood pressure threshold or 'control' as prespecified):** NR **Cardiovascular events‐stroke:** NR **Cardiovascular events‐myocardial infarction:** NR **Cardiovascular events‐other:** bradycardia; outcome measurement: NR; time points: 2, 4 weeks **Cardiovascular mortality:** NR **Blood potassium:** NR **Hyperkalaemia:** NR **Hypokalaemia:** NR **Secondary outcomes: ** **All‐cause mortality:** NR **Adverse events:** mild drowsiness, insomnia, decreased libido, depression, asthenia; outcome measurement: NR; time points: duration of study **Antihypertensive medication use:** NR **Body Mass index (BMI):** NR **Serum creatinine:** NR **Albuminuria:** NR **Urinary albumin‐to‐creatinine ratio (uACR):** NR **Fasting blood glucose:** NR **Blood triglycerides:** NR **Total blood cholesterol:** NR **24‐h urinary sodium excretion:** 24‐hour urine sample. Time points: 4 weeks **24‐h urinary potassium excretion:** 24‐hour urine sample. Time points: 4 weeks	
Notes	**Funding source:** NR **Authors name:** Giuseppe Suppa **Institution:** The Italian Group for the Prevention and Care of Arterial Hypertension **Email:** NR **Possible conflicts of interest (for study authors):** NR **Sources used for data extraction:** Journal article with results of the trial **Trial registration details:** NR	
***Risk of bias***		
**Bias**	**Authors' judgement**	**Support for judgement**
Random sequence generation (selection bias)	Unclear risk	Insufficient information on how the randomisation sequence was generated
Allocation concealment (selection bias)	Unclear risk	Insufficient information on how the randomisation sequence was protected
Blinding of participants and personnel (performance bias) All outcomes	Unclear risk	The study was reported as 'double‐blinded' but it was unclear what this meant. The salt was packaged in identical packets; therefore it was likely that participants were blinded. However, it was unclear whether the personnel were blinded.
Blinding of outcome assessment (detection bias) All outcomes	Unclear risk	The study was reported as 'double blinded', but it was unclear what this meant. BP measurements were conducted with a non‐automated conventional device and therefore detection bias was possible if outcome assessors were not blinded.
Incomplete outcome data (attrition bias) All outcomes	Low risk	No incomplete data reported for SBP, DBP outcomes
Selective reporting (reporting bias)	Unclear risk	Study protocol or prospective trial registry entry not available
Other bias	Low risk	None identified
Overall risk of bias	Unclear risk	Unclear risk of bias for allocation concealment; low risk of bias for incomplete outcome data

**Table:** Toft 2020

***Study characteristics***		
Methods	**Study design:** Cluster‐randomised controlled trial **Country:** Denmark **Setting:** Intervention conducted in households from five municipalities ((Albertslund, Ballerup, Egedal, Glostrup and Rødovre) in the Southwestern part of the Capital Region; outcomes measured at research centre **Comments:** SalT Reduction InterVEntion (STRIVE) study **Aim of study:** To examine the effects of two different salt reduction strategies on selected cardiovascular risk factors **Unit of allocation:** Families **Start date:** February 2018 **End date:** June 2018 **Relevant study limitations as reported by study authors:** Low statistical power due to a lower number of participants than planned in power calculations, low level of salt reduction in the intervention groups making it difficult to investigate the potential adverse effect of larger salt reduction; a confounding factor in these analyses could be changes in other dietary factors. This paper did not include dietary data. **Sample size calculation:** Estimated sample size of 25 families (100 participants) in each of the three groups, at a 5% confidence level and 80% power, based on expected reductions of 1.25 g salt (0.5 g sodium) per day in the intervention group receiving low‐sodium bread and 3 g salt (1.2 g sodium) per day in the intervention group receiving low‐sodium bread combined with dietary counselling. ICC within families = 0.33	
Participants	**Baseline Characteristics** LSSS intervention (adults) *Age*: in years, mean (SD): 41.5 (9.5) *Gender*: Male, % (n/N): 43.9 (18/41) *Ethnicity/race*: European *Smoking*: Smokers, % (n/N): 10.0 (4/41) *Body Mass Index (BMI)*: in kg/m^2^, mean (SD): 25.8 (3.8) *Blood pressure status*: Normotensive, % (n/N): 100 (41/41) *Antihypertensive medication used*: Any antihypertensive medication, % (n/N): 0 (0/41) *Cardiovascular disease or stroke*: CVD, % (n/N): 0 (0/41) *Diabetes mellitus*: DM, % (n/N): 0 (0/41) *Renal impairment*: Urinary albumin > 300 mg, % (n/N): 0 (0/41) *Dietary potassium intake*: NR *Dietary sodium intake*: in grams sodium per day, mean (SD): 3.64 (1.16) *Urinary potassium excretion*: NR *Urinary sodium excretion*: NR LSSS intervention (children) *Age*: in years, mean (SD): 9.5 (4.2) *Gender*: Male, % (n/N): 52.5 (21/40) *Ethnicity/race*: European *Smoking*: Smokers, % (n/N): 5.0 (2/40) *Body Mass Index (BMI)*: in kg/m^2^, mean (SD): 18.0 (2.9) *Blood pressure status*: Normotensive, % (n/N): 100 (40/40) *Antihypertensive medication used*: Any hypertensive medication used, % (n/N): 0 (0/40) *Cardiovascular disease or stroke*: CVD, % (n/N): 0 (0/40) *Diabetes mellitus*: DM, % (n/N): 0 (0/40) *Renal impairment*: Urinary albumin > 300 mg, % (n/N): 0 (0/40) *Dietary potassium intake*: NR *Dietary sodium intake*: in grams sodium per day, mean (SD): 2.48 (1.16) *Urinary potassium excretion*: NR *Urinary sodium excretion*: NR Control (adults) *Age*: Adults: in years, mean (SD): 40.9 (8.0) *Gender*: Male, % (n/N): 46.9 (23/49) *Ethnicity/race*: European *Smoking*: Smokers, % (n/N): 10.4 (5/49) *Body Mass Index (BMI)*: in kg per m^2^, mean (SD): 24.8 (4.1) *Blood pressure status*: Normotensive, % (n/N): 100 (49/49) *Antihypertensive medication used*: Any antihypertensive medication, % (n/N): 0 (0/49) *Cardiovascular disease or stroke*: CVD, % (n/N): 0 (0/49) *Diabetes mellitus*: DM, % (n/N): 0 (0/49) *Renal impairment*: Urinary albumin > 300 mg, % (n/N): 0 (0/49) *Dietary potassium intake*: NR *Dietary sodium intake*: in grams sodium per day, mean (SD): 3.64 (1.16) *Urinary potassium excretion*: NR *Urinary sodium excretion*: NR Control (children) *Age*: in years, mean (SD): 8.4 (3.5) *Gender*: Male, % (n/N): 51.9 (27/52) *Ethnicity/race*: European *Smoking*: Smokers, % (n/N): 0 (0/52) *Body Mass Index (BMI)*: in kg/m^2^, mean (SD): 16.9 (2.8) *Blood pressure status*: Normotensive, % (n/N): 100 (52/52) *Antihypertensive medication used*: Antihypertensive medication used, mean (SD): 0 (0/52) *Cardiovascular disease or stroke*: CVD, % (n/N): 0 (0/52) *Diabetes mellitus*: DM, % (n/N): 0 (0/52) *Renal impairment*: Urinary albumin > 300 mg, % (n/N): 0 (0/52) *Dietary potassium intake*: NR *Dietary sodium intake*: in grams sodium per day, mean (SD): 2.2 (0.88) *Urinary potassium excretion*: NR *Urinary sodium excretion*: NR Overall *Age*: NR *Gender*: NR *Ethnicity/race*: European *Smoking*: NR *Body Mass Index (BMI)*: NR *Blood pressure status*: NR *Antihypertensive medication used*: NR *Cardiovascular disease or stroke*: NR *Diabetes mellitus*: NR *Renal impairment*: NR *Dietary potassium intake*: NR *Dietary sodium intake*: NR *Urinary potassium excretion*: NR *Urinary sodium excretion*: NR Overall (children) *Age*: NR *Gender*: NR *Ethnicity/race*: European *Smoking*: NR *Body Mass Index (BMI)*: NR *Blood pressure status*: NR *Antihypertensive medication used*: NR *Cardiovascular disease or stroke*: NR *Diabetes mellitus*: NR *Renal impairment*: NR *Dietary potassium intake*: NR *Dietary sodium intake*: NR *Urinary potassium excretion*: NR *Urinary sodium excretion*: NR **Inclusion criteria:** Families with at least one child (3–17 years) and one parent (18–65 years) and a daily bread consumption among adults. Children of divorced parents had to stay more than half the time with the participating parent, if both parents were not participating. **Exclusion criteria:** Antihypertensive and lipid‐lowering treatment, pregnancy, diabetes, coronary heart disease and urine albumin > 300 mg/day **Pretreatment:** The study authors reported differences in BMI, activity levels and alcohol consumption between the groups, but did not confirm these differences with the use of statistical analysis. **Method of recruitment of participants:** Families were recruited through social media at schools, kindergartens and large companies, word‐of‐mouth and posters in the local area during the period January–February 2018. **Informed consent obtained:** Yes (written) **Clusters:** Families [family size: LSSS Intervention 3.2 (0.8); control 3.5 (1.1)] **Subgroups planned/measured:** NR **Subgroups reported:** Participants in the intervention groups that decreased their estimated daily salt intake by at least 3 g; or the sodium to potassium ratio by at least 20% from baseline to 4‐month follow‐up *Participant flow* **Assessed for eligibility:** n = 152 families (581 individuals) **Excluded (number with reasons):** n = 63 (n = 272 individuals): withdrew or did not fulfil eligibility criteria **Randomised:** n = 89 families (n = 309 individuals) **Allocated to LSSS intervention(s):** Salt‐reduced bread group: n = 25 families (n = 81 individuals) **Allocated to control:** Standard bread group: n = 29 families (n = 101 individuals) **Received allocated LSSS intervention(s):** NR **Did not receive allocated LSSS intervention(s):** NR **Lost to follow‐up (LSSS intervention group):** n = 7 individuals (n = 2 families); Reasons ‐ Lack of time: n = 3 individuals (1 family); Reason for not attending follow‐up not reported: n = 4 individuals (1 family) **Discontinued intervention (LSSS intervention group):** n = 0 **Analysed (LSSS intervention group):** Multiple imputation analysis: n = 41 adults; n = 40 children **Excluded from analysis (LSSS intervention group):** n = 0 **Received allocated control:** NR **Did not receive allocated control:** NR **Lost to follow‐up (control group):** n = 4 individuals (2 families); Reasons ‐ Family issues (n = 3 individuals from 1 family); Moved (n = 1 individual) **Discontinued intervention (control group):** n = 2 individuals (n = 1 family) **Analysed (control group):** Multiple imputation analysis: n = 49 adults; n = 52 children **Excluded from analysis (control group):** n = 0	
Interventions	**Intervention Characteristics** LSSS intervention *Theoretical basis*: To assess the effects of lowering sodium intake on cardiovascular risk factors *Description*: Gradually reduced salt whole grain bread products (0.48 g sodium/100 g or 1.2 g salt/100 g) for the first 2 weeks; gradually reduced by 0.08 g sodium/100g or 0.2 g salt/100g each week until 0.24 g sodium/100g or 0.6 g salt/100g was reached, which was maintained for the rest of the intervention period. This was achieved with the use of Viva Salt (per 100g: 8000 mg sodium; 870 mg and 100 mg‐200 mg K) *LSSS category*: < 30% KCl *Contains fortificant*: No *Delivery*: non‐discretionary use (bread products: rye bread, loaf, buns) *Duration of run‐in period*: none *Duration of active intervention*: 4 months *Duration of follow‐up (as reported)*: none *Total duration of study (as reported)*: 4 months *Timing*: Participants were instructed to replace their usual consumption of bread with the bread products provided in the study, and the amount was adjusted to fit the usual bread habits within the family. *Implementation*: The families collected the bread free of charge twice a week at the bakery or at the research centre, depending on preference. *Providers*: NR *Co‐interventions*: NR *Resource requirements*: NR *Integrity of delivery*: Missed bread hand‐outs were recorded, and all family members were asked to register their daily intake of intervention bread in compliance sheets given to the families on a weekly basis. Participants were regarded as compliant, if they collected 80% of the intervention bread and reported consumption of the intervention bread in the returned sheets at least 80% of the days. Control *Theoretical basis*: To enable comparison of LSSS intervention with standard practice *Description*: Standard wholegrain bread products (0.48 g sodium/100 g or 1.2 g salt/100 g) *Contains fortificant*: NR *Delivery*: non‐discretionary use (bread products: rye bread, loaf, buns) *Duration of run‐in period*: none *Duration of active intervention*: 4 months *Duration of follow‐up (as reported)*: none *Total duration of study (as reported)*: 4 months *Timing*: Participants were instructed to replace their usual consumption of bread with the bread products provided in the study, and the amount was adjusted to fit the usual bread habits within the family. *Implementation*: The families collected the bread free of charge twice a week at the bakery or at the research centre, depending on preference. *Providers*: NR *Co‐interventions*: NR *Resource requirements*: NR *Integrity of delivery*: Missed bread hand‐outs were recorded, and all family members were asked to register their daily intake of intervention bread in compliance sheets given to the families on a weekly basis. Participants were regarded as compliant, if they collected 80% of the intervention bread and reported consumption of the intervention bread in the returned sheets at least 80% of the days.	
Outcomes	**Primary outcomes: ** **Diastolic Blood Pressure (DBP):** Outcome measurement: Measured with an automatic BP monitor (Medidyne) 3 times in sitting position, after resting for 5 minutes; average taken of the last 2 measurements; time points: 4 months **Systolic Blood Pressure (SBP):** Outcome measurement: Measured with an automatic BP monitor (Medidyne) 3 times in sitting position, after resting for 5 minutes; average taken of the last 2 measurements; time points: 4 months **Hypertension (as reported, or SBP > 140 mmHg or DBP > 85 mmHg):** NR **Blood pressure control (achieving blood pressure threshold or 'control' as prespecified):** NR **Cardiovascular events‐stroke:** NR **Cardiovascular events‐myocardial infarction:** NR **Cardiovascular events‐other:** NR **Cardiovascular mortality:** NR **Blood potassium:** NR **Hyperkalaemia:** NR **Hypokalaemia:** NR **Secondary outcomes: ** **All‐cause mortality:** NR **Adverse events:** NR **Antihypertensive medication use:** NR **Body Mass index (BMI):** Outcome measurement: Body Mass Index (BMI) was calculated (kg/m^2^). Height was measured without shoes to the nearest cm, weight without shoes and coat to the nearest kg. Time point: baseline, 4 months **Serum creatinine:** NR **Albuminuria:** NR **Urinary albumin‐to‐creatinine ratio (uACR):** NR **Fasting blood glucose:** Outcome measurement: Blood samples from participants who fasted for at least 2 hours before arrival at research centre; measurement performed using colorimetric analysis; time points: 4 months **Blood triglycerides:** Outcome measurement: Blood samples from participants who fasted for at least 2 hours before arrival at research centre; measurement performed using colorimetric analysis; time points: 4 months **Total blood cholesterol:** Outcome measurement: Blood samples from participants who fasted for at least 2 hours before arrival at research centre; measurement performed using colorimetric analysis; time points: 4 months **24‐h urinary sodium excretion:** 24‐hour urine sample. Time points: 4 months **24‐h urinary potassium excretion:** 24‐hour urine sample. Time points: 4 months	
Notes	**Funding source:** Danish Heart Foundation (2016‐R107‐A6700‐22998¸2018‐R124‐A8520‐22098), The Research Fond of the Capital Region of Denmark, The Toyota Foundation, Ministry of Environment and Food of Denmark, Axel Muusfeldt’s Foundation and Sofus Friis’ Foundation **Authors name:** Ulla Toft **Institution:** Center for Clinical Research and Prevention, Bispebjerg and Frederiksberg Hospital; Department of Public Health, Faculty of Health and Medical Sciences, University of Copenhagen **Email:** ulla.toft@regionh.dk **Possible conflicts of interest (for study authors):** The authors declared no conflict of interest. The funders had no role in the design of the study; in the collection, analyses or interpretation of data; in the writing of the manuscript or in the decision to publish the results. **Sources used for data extraction:** Journal article with results of the trial; unpublished trial protocol and trial registry entry **Trial registration details:** NCT03810885	
***Risk of bias***		
**Bias**	**Authors' judgement**	**Support for judgement**
Random sequence generation (selection bias)	Low risk	Computer‐generated sequence of random group assignment
Allocation concealment (selection bias)	Low risk	Study authors reported the use of a computer‐generated sequence of random group assignment, and that the secretary who screened and enrolled participants was blinded with respect to group allocation.
Blinding of participants and personnel (performance bias) All outcomes	Low risk	Blinding of participants was achieved through colour‐coding of bread packaging. Study personnel were not blinded and the reduction in the salt content of the intervention bread may have resulted in taste changes in the intervention groups, However, the outcomes measured were objective clinical outcomes such as BP and blood concentrations e.g. blood lipids.
Blinding of outcome assessment (detection bias) All outcomes	Low risk	Outcome assessors (nurses) were blinded.
Incomplete outcome data (attrition bias) All outcomes	Low risk	Total attrition was low, 20/309 (6.5%), with no differential attrition between any group (9% in intervention A and 6% each in intervention B and the control group). Reasons for attrition were similar. Missing data were imputed using a multiple imputation approach.
Selective reporting (reporting bias)	Unclear risk	Some outcomes listed in the trial registry (NCT03810885), such as hip and waist circumference, baseline urine albumin and change in urinary creatinine, were not reported in the main results.
Other bias	Low risk	None identified
Recruitment bias (cluster‐RCTs)	Low risk	Baseline assessments were done before randomisation.
Comparability with individually randomised trials (cluster‐RCTs)	Unclear risk	The comparability of this study with other RCTs with similar LSSS interventions (i.e. non‐discretionary LSSS use), or at similar time points, was unclear.
Loss of clusters (cluster‐RCTs)	Low risk	Loss of clusters was low and similar between the groups (LSSS intervention group n = 2 families; control group n = 3 families).
Baseline imbalance (cluster‐RCTs)	Unclear risk	The study authors reported differences in BMI, activity levels and alcohol consumption between the groups, but did not confirm these differences with the use of statistical analysis.
Incorrect analysis (cluster‐RCTs)	Low risk	Missing data at baseline and follow‐up were imputed using multiple imputation with 100 samples and taking into account cluster effects. Linear mixed modelling was used with the cardiovascular risk factors at follow‐up as outcome variables, treatment groups and baseline measures of the risk factor as fixed‐effects and family ID as random‐effects.
Overall risk of bias	Unclear risk	Low risk of bias for incomplete outcome data, loss of clusters and recruitment bias; unclear risk of bias for baseline imbalance

**Table:** Yu 2021

***Study characteristics***		
Methods	**Study design:** Randomised controlled trial **Study grouping:** Parallel group **Country:** India **Setting:** Intervention conducted in villages in northern region of the Indian state of Telangana; outcomes measured during face‐to‐face follow‐up visits of households **Comments:** Salt Substitute in India Study (SSiIS) **Aim of study:** The primary aim of SSiIS is to evaluate the effect of a 3‐month reduced‐sodium, added‐potassium salt substitute intervention on SBP among patients with hypertension in rural India. The secondary aims are to determine the effects of the salt substitute on DBP, urinary sodium and potassium excretion, as well as to assess dietary sources of sodium and the acceptability of the salt substitute. **Unit of allocation:** Adults (age 18 years and older) **Start date:** November 2019 **End date:** April 2020 **Relevant study limitations as reported by study authors:** Short intervention duration; findings may not be generalisable to urban Indian hypertensive populations; exclusion of hypertensive patients with kidney disease; cannot rule out presence of undetected hyperkalaemia in some participants; no collection of 24‐hour urine biomarkers at one month follow‐up to determine trajectory of changes in urinary sodium and potassium over the study period, and did not assess the completeness of 24‐hour urine collections by administration of para‐aminobenzoic acid **Sample size calculation:** Estimated sample size was 498 participants, at 80% power and a significance level of 5% (one‐sided test). The power estimate assumed a mean baseline SBP of 140 mmHg (SD 20 mmHg) and a 20% loss to follow‐up. The study was powered to detect a 5 mmHg or greater difference in mean SBP between groups.	
Participants	**Baseline Characteristics** LSSS intervention *Age*: in years, mean (SD): 61.5 (11.1) *Gender*: Female, % (n/N): 58.3 (147/252) *Ethnicity/race*: Asian *Smoking*: Current smoking, % (n/N): 6.3 (16/252); past smoking, % (n/N): 8.3 (21/252) *Body Mass Index (BMI)*: in kg per m^2^, mean (SD): 23.1 (4.7) *Blood pressure status*: Hypertensive, % (n/N): 100 (252/252) *Antihypertensive medication used*: Any hypertensive medication use, % (n/N): 97.2 (245/252); diuretic, % (n/N): 0 (0/252); calcium channel blockers, % (n/N): 2.4 (6/252); ACE inhibitor or ARB, % (n/N): 27.8 (70/252); beta‐blockers, % (n/N): 25.0 (63/252); alpha‐blocker, % (n/N): 42.5 (107/252) *Cardiovascular disease or stroke*: History of CVD, % (n/N): 1.2 (3/252) *Diabetes mellitus*: History of DM, % (n/N): 23.4 (59/252) *Renal impairment*: Acute or chronic kidney disease, % (n/N): 0 (0/252) *Dietary potassium intake*: NR *Dietary sodium intake*: NR *Urinary potassium excretion*: in grams (24 hours), mean (SD): 0.82 (0.45) *Urinary sodium excretion*: in grams (24 hours), mean (SD): 3.80 (1.86) Control *Age*: in years, mean (SD): 61.7 (12.9) *Gender*: Female, % (n/N): 59.2 (148/250) *Ethnicity/race*: Asian *Smoking*: Current smoking, % (n/N): 5.6 (14/250); past smoking, % (n/N): 8.0 (20/250) *Body Mass Index (BMI)*: in kg per m^2^, mean (SD): 23.6 (4.2) *Blood pressure status*: Hypertensive, % (n/N): 100 (250/250) *Antihypertensive medication used*: Any hypertensive medication use, % (n/N): 94..4 (236/250); diuretic, % (n/N): 0.4 (1/250); calcium channel blockers, % (n/N): 3.6 (9/250); ACE inhibitor or ARB, % (n/N): 32.0 (80/250); beta‐blockers, % (n/N): 20.0 (50/250); alpha‐blocker, % (n/N): 39.0 (97/250) *Cardiovascular disease or stroke*: History of CVD, % (n/N): 1.6 (4/250) *Diabetes mellitus*: History of DM, % (n/N): 20.5 (51/250) *Renal impairment*: Acute or chronic kidney disease, % (n/N): 0 (0/250) *Dietary potassium intake*: NR *Dietary sodium intake*: NR *Urinary potassium excretion*: in grams (24 hours), mean (SD): 0.86 (0.53) *Urinary sodium excretion*: in grams (24 hours), mean (SD): 3.64 (1.73) Overall *Age*: NR *Gender*: NR *Ethnicity/race*: Asian *Smoking*: NR *Body Mass Index (BMI)*: NR *Blood pressure status*: NR *Antihypertensive medication used*: NR *Cardiovascular disease or stroke*: NR *Diabetes mellitus*: NR *Renal impairment*: Acute or chronic kidney disease, % (n/N): 0 (0/502) *Renal impairment*: NR *Dietary potassium intake*: NR *Dietary sodium intake*: NR *Urinary potassium excretion*: NR *Urinary sodium excretion*: NR **Inclusion criteria:** Adults aged 18 years or over with a history of hypertension diagnosed by a health professional (hypertension may be self‐reported and antihypertensive drugs may or may not be used for management) who eat most of their meals at home. **Exclusion criteria:** Participants or any of their household members with self‐reported acute or chronic kidney disease (according to standard diagnostic clinical criteria; verified by study physician at baseline), who used potassium‐sparing diuretics, potassium supplements, who were not expected to live longer than 6 months from the baseline assessment, or where another household member was already enrolled in the study **Pretreatment:** Baseline clinical and demographic characteristics of participants were similar between the intervention and the control group. **Method of recruitment of participants:** Villages were purposively selected based on their willingness to be involved and their proximity to the infrastructure necessary for the study, including delivery of the intervention salts and being accessible to study personnel. After we approached the households, each household decided for themselves which of the adult members of the household (who also met eligibility criteria) would be the study participant. **Informed consent obtained:** Yes (written consent) **Clusters:** n/a **Subgroups planned/measured:** NR **Subgroups reported:** Age < 65 years vs. >= 65 years; male vs. female; diabetes vs. no diabetes *Participant flow* **Assessed for eligibility:** n = 1923 **Excluded (number with reasons):** n = 1421 (n = 286 eat most meals outside the home; n = 21 withdrew consent; n = 350 had concerns about use of LSSS; n = 206 used a potassium‐sparing diuretic; n = 193 used potassium supplements; n = 117 had kidney disease; n = 132 another household member already enrolled; n = 101 not interested) **Randomised:** n = 502 **Allocated to LSSS intervention(s):** n = 252 **Allocated to control:** n = 252 **Received allocated LSSS intervention(s):** NR **Did not receive allocated LSSS intervention(s):** NR **Lost to follow‐up (LSSS intervention group):** n = 3 at 1 month (n = 2 lost contact; n = 1 death); n = 6 at 3 months (n = 4 lost contact; n = 2 hospitalised) **Discontinued intervention (LSSS intervention group):** n = 1 at 3 months **Analysed (LSSS intervention group):** SBP; DBP: n = 242; urinary sodium and potassium (24‐hour urine): n = 238; questionnaire: n = 242 **Excluded from analysis (LSSS intervention group):** Urinary sodium and potassium: n = 26 excluded due to incomplete urine samples **Received allocated control:** NR **Did not receive allocated control:** NR **Lost to follow‐up (control group):** n = 3 at 1 month (n = 3 lost contact); n = 7 at 3 months (n = 7 lost contact) **Discontinued intervention (control group):** n = 3 at 1 month; n = 3 at 3 months **Analysed (control group):** SBP, DBP: n = 234; urinary sodium and potassium (24‐hour urine): 224; questionnaire: 234 **Excluded from analysis (control group):** Urinary sodium and potassium: n = 29 excluded due to incomplete urine samples	
Interventions	**Intervention Characteristics** LSSS intervention *Theoretical basis*: LSSS may lower SBP and DBP through reduced dietary sodium and increased potassium consumption. *Description*: Reduced‐sodium, added‐potassium salt substitute (70% sodium chloride; 30% potassium chloride) *LSSS category*: ≥ 30% KCl *Contains fortificant*: iodine *Delivery*: discretionary use *Duration of run‐in period*: none *Duration of active intervention*: 3 months *Duration of follow‐up (as reported)*: none *Total duration of study (as reported)*: 3 months *Timing*: LSSS provided to replace all household salt, and be used in cooking, seasoning and food preservation throughout the study *Implementation*: Provision of a 3‐month supply by study personnel during second face‐to‐face study visit to household (calculated as an average of 20 g/person/day to a maximum of 5 kg per 3 months for a household) *Providers*: Siddharth Starch Pvt. Ltd. company based in Maharashtra, India blended and supplied the LSSS. *Co‐interventions*: NR *Resource requirements*: NR *Integrity of delivery*: Acceptability and adherence assessed with questionnaire. Reported LSSS use at 1 month: No. of days in week prior, mean (SD): 6.5 (1.6). No. of meals per typical day, mean (SD): 2.7 (0.8). Reported LSSS use at 3 months: No. of days in week prior, mean (SD): 6.3 (1.8). No. of meals per typical day, mean (SD): 2.6 (0.6) Control *Theoretical basis*: To enable comparison of LSSS intervention with standard practice *Description*: Regular salt (100% sodium chloride) *Contains fortificant*: iodine *Delivery*: discretionary use *Duration of run‐in period*: none *Duration of active intervention*: 3 months *Duration of follow‐up (as reported)*: none *Total duration of study (as reported)*: 3 months *Timing*: Regular salt provided to replace all household salt, and be used in cooking, seasoning and food preservation throughout the study *Implementation*: Provision of a 3‐month supply by study personnel during second face‐to‐face study visit to household (calculated as an average of 20 g/person/day to a maximum of 5 kg per 3 months for a household) *Providers*: Siddharth Starch Pvt. Ltd. company based in Maharashtra, India supplied regular salt. *Co‐interventions*: NR *Resource requirements*: NR *Integrity of delivery*: Acceptability and adherence assessed with questionnaire. Reported LSSS use at 1 month: No. of days in week prior, mean (SD): 6.4 (1.6). No. of meals per typical day, mean (SD): 2.6 (0.5). Reported LSSS use at 3 months: No. of days in week prior, mean (SD): 6.3 (1.8). No. of meals per typical day, mean (SD): 2.5 (0.7)	
Outcomes	**Primary outcomes: ** **Diastolic Blood Pressure (DBP):** Outcome measurement: measured three times for each participant, in a sitting position in a quiet room, using an automated BP monitor (A&D Medical) according to standardised methods and criteria, and the mean of the last two measurements was recorded. Time points: 1, 3 months **Systolic Blood Pressure (SBP):** Outcome measurement: measured three times for each participant, in a sitting position in a quiet room, using an automated BP monitor (A&D Medical) according to standardised methods and criteria, and the mean of the last two measurements was recorded. Time points: 1, 3 months **Hypertension (as reported, or SBP > 140 mmHg or DBP > 85 mmHg):** NR **Blood pressure control (achieving blood pressure threshold or 'control' as prespecified):** NR **Cardiovascular events‐stroke:** NR **Cardiovascular events‐myocardial infarction:** NR **Cardiovascular events‐other:** NR **Cardiovascular mortality:** NR **Blood potassium:** NR **Hyperkalaemia:** Outcome measurement: Investigation of suspected clinical cases of hyperkalaemia (methods NR); time points: 1, 3 months **Hypokalaemia:** NR **Secondary outcomes: ** **All‐cause mortality:** NR **Adverse events:** Outcome measurement: Recording of all adverse events among study participants or household members, whether likely to be related to the study intervention or not; time points: 1, 3 months **Antihypertensive medication use:** NR **Body Mass index (BMI):** NR **Serum creatinine:** NR **Albuminuria:** NR **Urinary albumin‐to‐creatinine ratio (uACR):** NR **Fasting blood glucose:** NR **Blood triglycerides:** NR **Total blood cholesterol:** NR **24‐h urinary sodium excretion:** 24‐hour urine sample. Time points: 3 months **24‐h urinary potassium excretion:** 24‐hour urine sample. Time points: 3 months	
Notes	**Funding source:** The George Institute for Global Health Seed Grant (grant number 0141030). Regular salt used in the study was provided free of charge by Siddharth Starch Pvt. Ltd. company. **Authors name:** Jie Yu **Institution:** The George Institute for Global Health, University of New South Wales, Sydney, New South Wales, Australia and Department of Cardiology, Peking University Third Hospital, Beijing, China **Email:** jyu1@georgeinstitute.org.au **Possible conflicts of interest (for study authors):** The authors reported no conflicts of interest.	
***Risk of bias***		
**Bias**	**Authors' judgement**	**Support for judgement**
Random sequence generation (selection bias)	Low risk	The authors stated that random assignment occurred through a central computerised process, done by an independent biostatistician.
Allocation concealment (selection bias)	Low risk	Participants were allocated to either group according to a prepared random allocation sequence list, drawn up by an independent biostatistician.
Blinding of participants and personnel (performance bias) All outcomes	Low risk	Participants and study personnel were blinded. LSSS and regular salt were provided as masked, identical packages, with only the unique identification number on each pack.
Blinding of outcome assessment (detection bias) All outcomes	Low risk	Study personnel who collected outcome data were blinded.
Incomplete outcome data (attrition bias) All outcomes	Low risk	BP data were available for 96 % (242/252) and 92.6% (234/250) of intervention and control participants, respectively. In addition, the study authors demonstrated that there were no differences in demographic and clinical characteristics of participants who completed the study, compared to those who did not (supplementary data).
Selective reporting (reporting bias)	Unclear risk	Primary and secondary outcomes reported according to the published study protocol. Pulse rate and medication use were indicated as outcomes at one and three months, but were not reported. Dietary recall (24 h) was also indicated as an outcome at three months, but was not reported. The authors reported a subgroup analysis which was not prespecified.
Other bias	Low risk	None identified
Overall risk of bias	Low risk	Low risk of bias for allocation concealment and incomplete outcome data

**Table:** Zhang 2015

***Study characteristics***		
Methods	**Study design:** Cluster‐randomised controlled trial **Country:** China **Setting:** Intervention conducted in nursing houses in northern China; outcomes measured in these nursing houses **Aim of study:** To observe the long‐term effect of consuming enriched potassium salt (KCl:NaCl = 1:1 by weight) on blood pressure, all‐cause mortality, target organ damage and safety in persons living in Chinese nursing houses. **Unit of allocation:** Nursing houses **Start date:** 2011 to 2012 **End date:** NR **Relevant study limitations as reported by study authors:** NR **Sample size calculation:** NR	
Participants	**Baseline Characteristics** LSSS intervention *Age*: NR *Gender*: NR *Ethnicity/race*: Chinese *Smoking*: NR *Body Mass Index (BMI)*: NR *Blood pressure status*: NR *Antihypertensive medication used*: NR *Cardiovascular disease or stroke*: NR *Diabetes mellitus*: NR *Renal impairment*: Serum creatinine in µmol/L, mean (SD): 69 (18.7) *Dietary potassium intake*: NR *Dietary sodium intake*: NR *Urinary potassium excretion*: in mmol/24 h, mean (SD): 25.4 (10.0) *Urinary sodium excretion*: NR Control *Age*: NR *Gender*: NR *Ethnicity/race*: Chinese *Smoking*: NR *Body Mass Index (BMI)*: NR *Blood pressure status*: NR *Antihypertensive medication used*: NR *Cardiovascular disease or stroke*: NR *Diabetes mellitus*: NR *Renal impairment*: Serum creatinine in µmol/L, mean (SD): 69 (20.4) *Dietary potassium intake*: NR *Dietary sodium intake*: NR *Urinary potassium excretion*: NR *Urinary sodium excretion*: NR Overall *Age*: in years, mean: 65 *Gender*: NR *Ethnicity/race*: Chinese *Smoking*: NR *Body Mass Index (BMI)*: NR *Blood pressure status*: NR *Antihypertensive medication used*: NR *Cardiovascular disease or stroke*: NR *Diabetes mellitus*: NR *Renal impairment*: NR *Dietary potassium intake*: NR *Dietary sodium intake*: NR *Urinary potassium excretion*: NR *Urinary sodium excretion*:NR **Inclusion criteria:** Clusters: nursing houses in the northern regions of China. Participants: inhabitants of nursing houses **Exclusion criteria:** None reported **Pretreatment:** No apparent imbalance **Method of recruitment of participants:** NR **Informed consent obtained:** NR **Clusters:** n = 30 clusters (number of participants NR); no details on whether analyses were adjusted for clustering **Subgroups planned/measured:** NR **Subgroups reported:** NR *Participant flow* **Assessed for eligibility:** NR **Excluded (number with reasons):** NR **Randomised:** n = 30 clusters; number of participants NR **Allocated to LSSS intervention(s):** NR **Allocated to control:** NR **Received allocated LSSS intervention(s):** NR **Did not receive allocated LSSS intervention(s):** NR **Lost to follow‐up (LSSS intervention group):** NR **Discontinued intervention (LSSS intervention group):** NR **Analysed (LSSS intervention group):** n = 30 clusters at 3 to 5 months and 1 to 1.5 years, n = 22 clusters at 3 years, and n = 28 clusters at 4 years in total group (trial arm distribution NR); n = 1105 participants at 3 to 5 months and 1 to 1.5 years, n = 1032 at 3 years, and number of participants NR at 4 years **Excluded from analysis (LSSS intervention group):** NR **Received allocated control:** NR **Did not receive allocated control:** NR **Lost to follow‐up (control group):** NR **Discontinued intervention (control group):** NR **Analysed (control group):** n = 30 clusters at 3 to 5 months and 1 to 1.5 years, n = 22 clusters at 3 years, and n = 28 clusters at 4 years in total group (trial arm distribution NR); n = 947 participants at 3 to 5 months and 1 to 1.5 years, n = 807 at 3 years, and number of participants NR at 4 years **Excluded from analysis (control group):** NR	
Interventions	**Intervention Characteristics** LSSS intervention *Theoretical basis*: Replacing regular salt with enriched potassium salt may have implications for blood pressure, safety, mortality and organ damage in older persons. *Description*: Enriched potassium salt (NaCl:KCl = 1:1) *LSSS category*: ≥ 30% KCl *Contains fortificant*: NR *Delivery*: discretionary use *Duration of run‐in period*: none *Duration of active intervention*: 4 years *Duration of follow‐up (as reported)*: none *Total duration of study (as reported)*: 4 years *Timing*: NR *Implementation*: 10 g/person/day dispensed to each nursing home every 3 months *Providers*: NR *Co‐interventions*: NR *Resource requirements*: NR *Integrity of delivery*: NR Control *Theoretical basis*: To enable comparison of LSSS intervention with standard practice *Description*: 'Normal' salt used *Contains fortificant*: NR *Delivery*: discretionary use *Duration of run‐in period*: none *Duration of active intervention*: 4 years *Duration of follow‐up (as reported)*: none *Total duration of study (as reported)*: 4 years *Timing*: NR *Implementation*: 10 g/person/day dispensed to each nursing home every 3 months *Providers*: NR *Co‐interventions*: NR *Resource requirements*: NR *Integrity of delivery*: NR	
Outcomes	**Primary outcomes: ** **Diastolic Blood Pressure (DBP):** Outcome measurement: NR. Time points: 3 to 5 months, 1 to 1.5 years, 3, 4 years **Systolic Blood Pressure (SBP):** Outcome measurement: NR. Time points: 3 to 5 months, 1 to 1.5 years, 3, 4 years **Hypertension (as reported, or SBP > 140 mmHg or DBP > 85 mmHg):** NR **Blood pressure control (achieving blood pressure threshold or 'control' as prespecified):** NR **Cardiovascular events‐stroke:** NR **Cardiovascular events‐myocardial infarction:** NR **Cardiovascular events‐other:** NR **Cardiovascular mortality:** NR **Blood potassium:** Outcome measurement: NR. Time points: 3 to 5 months, 1 to 1.5 years, 4 years **Hyperkalaemia:** Outcome measurement: Defined as > 5.5 mmol/L. Time points: 1 to 1.5 years **Hypokalaemia:** NR **Secondary outcomes: ** **All‐cause mortality:** Outcome measurement: calculated by observed person‐years; time points: 3, 4 years **Adverse events:** NR **Antihypertensive medication use:** NR **Body Mass index (BMI):** NR **Serum creatinine:** Outcome measurement: NR. Time points: 3 to 5 months, 1 to 1.5 years, 4 years **Albuminuria:** Outcome measurement: Defined as ratio of microalbumin to creatinine ≥ 30 mg/g. Time points: 3 years **Urinary albumin‐to‐creatinine ratio (uACR):** NR **Fasting blood glucose:** NR **Blood triglycerides:** NR **Total blood cholesterol:** NR **24‐h Urinary sodium excretion:** NR **24‐h Urinary potassium excretion:** NR	
Notes	**Funding source:** NR **Authors name:** Hongye Zhang **Institution:** Beijing Hypertension League Institute, Beijing, China **Email:** NR **Possible conflicts of interest (for study authors):** NR **Sources used for data extraction:** Conference abstract about the trial **Trial registration details:** NR	
***Risk of bias***		
**Bias**	**Authors' judgement**	**Support for judgement**
Random sequence generation (selection bias)	Unclear risk	No information on how the randomisation sequence was generated
Allocation concealment (selection bias)	Unclear risk	No information on how the randomisation sequence was protected
Blinding of participants and personnel (performance bias) All outcomes	Unclear risk	There was no information available on whether participants and people delivering the intervention were blinded. Outcomes are objective and not likely to be affected by a lack of blinding, but modifications in behaviour due to awareness of assignment may have resulted in some performance bias.
Blinding of outcome assessment (detection bias) All outcomes	Unclear risk	Insufficient information available
Incomplete outcome data (attrition bias) All outcomes	Unclear risk	Insufficient information available
Selective reporting (reporting bias)	Unclear risk	No study protocol or prospective trial registry entry available
Other bias	Low risk	None identified
Recruitment bias (cluster‐RCTs)	Unclear risk	Insufficient information reported on whether clusters were identified before randomisation
Comparability with individually randomised trials (cluster‐RCTs)	Low risk	A previous RCT investigated the use of mineral salt with 10% potassium in middle‐aged and elderly Japanese and also found it to be a safe alternative to regular salt (Kawasaki 1998). This study also found a significant decrease in blood pressure in those consuming the mineral salt. Another RCT in older Dutch people found that replacing regular salt with low‐sodium high‐potassium salt led to blood pressure reduction in people with mild to moderate hypertension (Geleijnse 1994). A parallel‐design study (Zhou 2009) also reported similar reduction of blood pressure.
Loss of clusters (cluster‐RCTs)	High risk	A total of 27% (8/30) of clusters were not included in the analysis at year 3, and 7% (2/30) of clusters were not included in the analysis at year 4.
Baseline imbalance (cluster‐RCTs)	Unclear risk	Based on five baseline characteristics reported, there appeared to be no serious baseline imbalance initially; however, due to in‐ and out‐migration to and from the nursing homes, it was not clear whether baseline equivalence was maintained over time.
Incorrect analysis (cluster‐RCTs)	Unclear risk	Insufficient information on statistical analysis and adjustment for clustering in conference abstracts (full text not available)
Overall risk of bias	High risk	Unclear risk of bias for incomplete outcome data, recruitment bias and baseline imbalance; high risk for loss of clusters

**Table:** Zhao 2014

***Study characteristics***		
Methods	**Study design:** Randomised controlled trial **Study grouping:** Parallel group **Country:** Tibet **Setting:** Intervention conducted in households in two townships (Yangbajing and Gongtangxiang); setting where outcomes were measured was not reported. **Aim of study:** To evaluate the effect of low‐sodium, high‐potassium salt substitute in blood pressure reduction in Tibetans living at high altitudes **Unit of allocation:** adults (aged 18 years or older) **Start date:** February 2009 **End date:** May 2009 **Relevant study limitations as reported by study authors:** A single follow‐up was done and urinary potassium and sodium were not measured, due to financial constraints and difficult sampling conditions. In addition, baseline information on education, occupation, smoking and alcohol use were only collected for a subsample of participants. Salt consumption data collected did not include sodium intake from sodium naturally occurring in food and was also only done for a small subsample. The study definition of hypertension excluded isolated diastolic hypertension, limiting findings. The mean blood pressure in the control group dropped significantly, which the authors attributed to either seasonal effects or regression to the mean (which should have taken into account the 'white coat hypertension' effect). **Sample size calculation:** The study was powered to detect a difference of 5.0 mmHg in systolic blood pressure at 90% power using a one‐tailed test and alpha of 0.05.	
Participants	**Baseline Characteristics** LSSS intervention *Age*: in years, mean (SD): 62.8 (11.1) *Gender*: Female, % (n/N): 60.3 (85/141) *Ethnicity/race*: Tibetan *Smoking*: Smokers (20 packs smoked in their lifetime or at least one cigarette per day for at least one year), % (n/N): 8.2 (6/141) *Body Mass Index (BMI)*: in kg/^2^, mean (SD): 23.7 (3.1) *Blood pressure status*: Systolic hypertension (≥ 140 mmHg), % (n/N): 100 (141/141); stage 1 hypertension, % (n/N): 20.6 (29/141); stage 2 hypertension, % (n/N): 79.4 (112/141) *Antihypertensive medication used*: Antihypertensive use in the past month, % (n/N): 47.0 (61/141); average number of antihypertensive medicines taken, mean (SD): 0.4 (0.5) *Cardiovascular disease or stroke*: NR *Diabetes mellitus*: NR *Renal impairment*: History of kidney disease, % (n/N): 0 (0/141) *Dietary potassium intake*: NR *Dietary sodium intake*: NR *Urinary potassium excretion*: NR *Urinary sodium excretion*: NR Control *Age*: in years, mean (SD): 63.5 (11.3) *Gender*: Female, % (n/N): 57.4 (81/141) *Ethnicity/race*: Tibetan *Smoking*: Smokers (20 packs smoked in their lifetime or at least one cigarette per day for at least one year), % (n/N): 9.7 (7/141) *Body Mass Index (BMI)*: in kg/m^2^, mean (SD): 23.6 (3.4) *Blood pressure status*: Systolic hypertension (≥ 140 mmHg), % (n/N): 100 (141/141); stage 1 hypertension, % (n/N): 19.1 (27/141); stage 2 hypertension, % (n/N): 80.9 (114/141) *Antihypertensive medication used*: Antihypertensive use in the past month, % (n/N): 50.7 (71/141); average number of antihypertensive medicines taken, mean (SD): 0.5 (0.5) *Cardiovascular disease or stroke*: NR *Diabetes mellitus*: NR *Renal impairment*: History of kidney disease, % (n/N): 0 (0/141) *Dietary sodium intake*: NR *Urinary potassium excretion*: NR *Urinary sodium excretion*: NR **Inclusion criteria:** Participants 40 years or older in the Gongtangxian, Yangbajing and Gongtangxiang townships; systolic blood pressure was confirmed greater or equal to 140 mmHg regardless use of antihypertensive medications **Exclusion criteria:** Participants were excluded for current use of potassium supplements, previously diagnosed kidney disease or gout (or if they lived in a household with someone who does), if their physician judged that they could not take a salt substitute, or if they intended to use salt outside the assigned treatment salt. **Pretreatment:** None **Method of recruitment of participants:** Potentially eligible participants were identified in a community‐based survey; they were first invited to a hypertension screening, and those who had a systolic blood pressure ≥ 140 mmHg were invited to the trial three months later. If the person's systolic blood pressure was again confirmed as ≥ 140 mmHg they were invited to participate in the trial, regardless of their antihypertensive medication use. **Informed consent obtained:** Yes, unclear whether written or oral. Provided by participants and family patriarchs **Clusters:** n/a **Subgroups planned/measured:** NR **Subgroups reported:** NR *Participant flow* **Assessed for eligibility:** n = 287 **Excluded (number with reasons):** n = 5 (n = 1 too old and weak to travel, n = 1 withdrew after informed consent, n = 3 excluded due to living too far thereby making follow‐up too difficult) **Randomised:** n = 282 **Allocated to LSSS intervention(s):** n = 141 **Allocated to control:** n = 141 **Received allocated LSSS intervention(s):** NR **Did not receive allocated LSSS intervention(s):** NR **Lost to follow‐up (LSSS intervention group):** n = 42 (n = 14 lost to follow‐up, n = 1 fatal cerebral haemorrhage, n = 27 discontinued intervention) **Discontinued intervention (LSSS intervention group):** n = 27 (n = 25 use study salt and local salt alternately, n = 1 abdominal distension, n = 1 stomach ache) **Analysed (LSSS intervention group):** n = 99 per protocol; n = 141 intention‐to‐treat **Excluded from analysis (LSSS intervention group):** n = 42 (n = 14 lost to follow‐up, n = 1 fatal cerebral haemorrhage, n = 27 discontinued intervention) **Received allocated control:** NR **Did not receive allocated control:** NR **Lost to follow‐up (control group):** n = 27 (n = 17 lost to follow‐up, n = 1 fatal cerebral haemorrhage, n = 1 fatal liver cancer, n = 8 discontinued intervention) **Discontinued intervention (control group):** n = 8 (n = 7 use study salt and local salt alternately, n = 1 complained of poor taste) **Analysed (control group):** n = 114 per protocol; n = 141 intention‐to‐treat **Excluded from analysis (control group):** n = 27 (n = 17 lost to follow‐up, n = 1 fatal cerebral haemorrhage, n = 1 fatal liver cancer, n = 8 discontinued intervention)	
Interventions	**Intervention Characteristics** LSSS intervention *Theoretical basis*: Hypertension and a very high burden of stroke is prevalent among Tibetans, due to high dietary salt intake. Previously studies have shown marked reductions in systolic blood pressure with the use of LSSS in Han Chinese. LSSS may result in even more dramatic reductions in hypertension among Tibetans. *Description*: salt substitute (65% NaCl; 25% KCl; 10% MgSO_4_) *LSSS category*: < 30% KCl *Contains fortificant*: NR *Delivery*: discretionary use *Duration of run‐in period*: none *Duration of active intervention*: 3 months *Duration of follow‐up (as reported)*: none *Total duration of study (as reported)*: 3 months *Timing*: LSSS to be used exclusively by the entire household for the duration of the study for cooking meals, making yak tea and bacon, and as table salt. *Implementation*: LSSS was provided free of charge to participants, in sufficient amounts to cover all household uses during the study period. LSSS was provided in containers indistinguishable from those for the control salt, and the two study salts are also indistinguishable in their appearance. In previous triangle food tests, 70 to 80% of taste testers could not tell the difference between LSSS and regular salt. *Providers*: NR *Co‐interventions*: Patients with pre‐existing antihypertensive medications were not directed to alter their prior regimen. *Resource requirements*: NR *Integrity of delivery*: Participants who intended on using salt outside the LSSS were excluded at randomisation, but n = 25 did use study salt and local salt alternately during the study. Investigators visited a random sample of households to determine acceptance of LSSS at one month. At the end of the study, investigators weighed salt to determine the amount consumed by the entire household. Control *Theoretical basis*: To enable comparison of LSSS intervention with standard practice *Description*: Regular salt (100% NaCl) *Contains fortificant*: NR *Delivery*: discretionary use *Duration of run‐in period*: none *Duration of active intervention*: 3 months *Duration of follow‐up (as reported)*: none *Total duration of study (as reported)*: 3 months *Timing*: Regular salt to be used exclusively by the entire household for the duration of the study for cooking meals, making yak tea and bacon, and as table salt. *Implementation*: Regular salt was provided free of charge to participants, in sufficient amounts to cover all household uses during the study period. Regular salt was provided in containers indistinguishable from those for the LSSS, and the two study salts were also indistinguishable in their appearance. In previous triangle food tests, 70 to 80% of taste testers could not tell the difference between LSSS and regular salt. *Providers*: NR *Co‐interventions*: Patients with pre‐existing antihypertensive medications were not directed to alter their prior regimen. *Resource requirements*: NR *Integrity of delivery*: Participants who intended on using salt outside the provided salt were excluded at randomisation, but n = 7 did use study salt and local salt alternately during the study. Investigators visited a random sample of households to determine acceptance of regular salt at one month. At the end of the study, investigators weighed salt to determine the amount consumed by the entire household.	
Outcomes	**Outcomes** **Primary outcomes: ** **Diastolic Blood Pressure (DBP):** Outcome measurement: Measurements taken with an automatic sphygmomanometer (after 5 mins rest) in sitting position. Three measurements with 1 minute in between taken in a quiet room. Time points: 3 months **Systolic Blood Pressure (SBP):** Outcome measurement: Measurements taken with an automatic sphygmomanometer (after 5 mins rest) in sitting position. Three measurements with 1 minute in between taken in a quiet room. Time points: 3 months **Hypertension (as reported, or SBP > 140 mmHg or DBP > 85 mmHg):** NR **Blood pressure control (achieving blood pressure threshold or 'control' as prespecified):** Outcome measurement: Defined as systolic blood pressure < 140 and diastolic blood pressure < 90 mmHg. Time points: 3 months **Cardiovascular events‐stroke:** NR **Cardiovascular events‐myocardial infarction:** NR **Cardiovascular events‐other:** NR **Cardiovascular mortality:** NR **Blood potassium:** NR **Hyperkalaemia:** NR **Hypokalaemia:** NR **Secondary outcomes:** **All‐cause mortality:** NR **Adverse events:** Abdominal distension, stomach ache; Outcome measurement: NR. Time points: duration of study **Antihypertensive medication use:** Outcome measurement: NR. Time points: duration of study **Body Mass index (BMI):** NR **Serum creatinine:** NR **Albuminuria:** NR **Urinary albumin‐to‐creatinine ratio (uACR):** NR **Fasting blood glucose:** NR **Blood triglycerides:** NR **Total blood cholesterol:** NR **24‐h urinary sodium excretion:** NR **24‐h urinary potassium excretion:** NR	
Notes	**Funding source:** The study was funded by the Bureau of Science and Technology of Tibet Autonomous Region Grant; the United States National Heart, Lung, and Blood Institute (National Institutes of Health); the United Health Group Chronic Disease Initiative; and the People's Republic of China Ministry of Science and Technology. **China Salt Substitute Study in Tibet (CSSS‐Tibet)** **Authors name:** Xingshan Zhao and Yangfeng Wu **Institution:** Department of Cardiology, Beijing Jishuitan Hospital, Peking University (X. Zhao); The George Institute for Global Health at Peking University Health Science Center and Department of Epidemiology and Biostatistics, Peking University (Y. Wu) **Email:** ywu@georgeinstitute.org.cn **Possible conflicts of interest (for study authors):** "There [are] no competing interests there and the funding does not alter the authors' adherence to PLOS One policies on sharing data and materials." **Sources used for data extraction:** Journal article with results of the trial, trial protocol published with journal article, non‐commercial trial registry record **Trial registration details:** NCT01429246	
***Risk of bias***		
**Bias**	**Authors' judgement**	**Support for judgement**
Random sequence generation (selection bias)	Low risk	A random number generator and computer‐generated randomisation list was used to assign participants following stratification by township, gender and dichotomised baseline SBP.
Allocation concealment (selection bias)	Low risk	A random number generator provided a treatment allocation identification number to each patient, and assignment was secured in a password‐protected encrypted digital registry. Salt was provided in indistinguishable containers with only the study allocation number.
Blinding of participants and personnel (performance bias) All outcomes	Low risk	Only participants were blinded to treatment allocation; the registry also reported that this was a single‐blinded trial. Intervention and control salts and containers were indistinguishable in appearance, and 70 to 80% of taste testers could not tell the difference between the two. The authors reported successful participant blinding as one control participant left the study due to 'poor taste' of the salt. Outcomes were objective in nature and it was unlikely that a lack of personnel blinding would have resulted in performance bias.
Blinding of outcome assessment (detection bias) All outcomes	Low risk	Outcome assessors were not blinded, but blood pressure and mortality outcomes are highly objective and not prone to detection bias. In the case of medication use and adverse events, participants were the outcome assessors; and they were blinded.
Incomplete outcome data (attrition bias) All outcomes	Low risk	Overall attrition was high, with 42/141 (30%) in the intervention and 27/141 (19%) in the control group. Of these, 27/141 (19%) intervention and 8/141 (6%) control participants discontinued use of the salts. Intention‐to‐treat analyses using multiple imputation were performed for the primary outcomes; with no change in nominal significance for these variables.
Selective reporting (reporting bias)	Unclear risk	Study protocol available (published concurrently with journal article) confirmed three‐month follow‐up, and indicated that six‐month follow‐up (with minor alterations to intervention) was intended. Intended outcomes were not explicitly reported. Trial was registered retrospectively.
Other bias	Low risk	None identified
Overall risk of bias	Low risk	Low risk of bias for allocation concealment and incomplete outcome data

**Table:** Zhou 2009

***Study characteristics***		
Methods	**Study design:** Randomised controlled trial **Study grouping:** Parallel group **Country:** China **Setting:** Intervention conducted in rural households in the Hedong district, Tianjin; outcomes measured at participants' homes **Aim of study:** To evaluate the safety and efficacy of Compound Ion Salt on blood pressure control in an aged population with high dietary salt intake **Unit of allocation:** adults (aged 18 years or older) **Start date:** September 2003 **End date:** May 2004 **Relevant study limitations as reported by study authors:** The single‐blinded design of the study may have resulted in systematic bias in blood pressure measurement; only aged people were included in the study due to their regularised work‐rest schedule and them taking most meals at home, thereby limiting generalisability; quantitative determination of salty flavour perception was not done, only flavour tests in a small population which found no difference in perceived taste between the intervention and control. **Sample size calculation:** The sample size was calculated to detect a ≥ 8 mmHg difference in systolic blood pressure with 80% power and significance of 5% using a two‐tailed test.	
Participants	**Baseline Characteristics** LSSS intervention (participants with normal blood pressure) *Age*: in years, mean (SD): 68.1 (8.3) *Gender*: Female, % (n/N): 50.9 (29/57) *Ethnicity/race*: Han Chinese *Smoking*: Current smoker, % (n/N): 21.1 (12/57); past smoker, % (n/N): 12.3 (7/57) *Body Mass Index (BMI)*: in kg/m^2^, mean (SD): 23.9 (3.2) *Blood pressure status*: Participants with normal blood pressure, % (n/N): 100 (57/57) *Antihypertensive medication used*: n/a *Cardiovascular disease or stroke*: Heart attack or stroke in past 6 months, current angina pectoris or congestive heart failure, % (n/N): 0 (0/57) *Diabetes mellitus*: % (n/N): 0 (0/57) *Renal impairment*: Serum creatinine, in µmol/L, mean (SD): 75.6 (21.2) *Dietary potassium intake*: NR *Dietary sodium intake*: NR *Urinary potassium excretion*: in mmol/24 h, mean (SD): 22.8 (7.15) *Urinary sodium excretion*: in mmol/24 h, mean (SD): 237 (50.0) Control (participants with normal blood pressure) *Age*: in years, mean (SD): 65.4 (4.5) *Gender*: Female, % (n/N): 55.4 (36/65) *Ethnicity/race*: Han Chinese *Smoking*: Current smoker, % (n/N): 24.6 (16/65); past smoker, % (n/N): 12.3 (8/65) *Body Mass Index (BMI)*: in kg/m^2^, mean (SD): 23.7 (3.3) *Blood pressure status*: Participants with normal blood pressure, % (n/N): 100 (65/65) *Antihypertensive medication used*: n/a *Cardiovascular disease or stroke*: Heart attack or stroke in past 6 months, current angina pectoris or congestive heart failure, % (n/N): 0 (0/65) *Diabetes mellitus*: % (n/N): 0 (0/65) *Renal impairment*: Serum creatinine, in µmol/L, mean (SD): 77.5 (18.9) *Dietary potassium intake*: NR *Dietary sodium intake*: NR *Urinary potassium excretion*: in mmol/24 h, mean (SD): 23.7 (7.81) *Urinary sodium excretion*: in mmol/24 h, mean (SD): 239 (36.6) LSSS intervention (participants with hypertension) *Age*: in years, mean (SD): 67.5 (5.2) *Gender*: Female, % (n/N): 56.5 (35/62) *Ethnicity/race*: Han Chinese *Smoking*: Current smoker, % (n/N): 21.0 (13/62); past smoker, % (n/N): 9.7 (6/62) *Body Mass Index (BMI)*: in kg/m^2^, mean (SD): 25.2 (3.5) *Blood pressure status*: Mild or moderate hypertension, % (n/N): 100 (62/62) *Antihypertensive medication used*: % (n/N): 53.2 (33/62) *Cardiovascular disease or stroke*: Heart attack or stroke in past 6 months, current angina pectoris or congestive heart failure, % (n/N): 0 (0/62) *Diabetes mellitus*: %, (n/N): 0 (0/62) *Renal impairment*: Serum creatinine, in µmol/L, mean (SD): 78.5 (18.5) *Dietary sodium intake*: NR *Urinary potassium excretion*: in mmol/24 h, mean (SD): 23.5 (7.16) *Urinary sodium excretion*: in mmol/24 h, mean (SD): 238 (38.5) Control (participants with hypertension) *Age*: in years, mean (SD): 65.7 (6.3) *Gender*: Female, % (n/N): 57.8 (37/64) *Ethnicity/race*: Han Chinese *Smoking*: Current smoker, % (n/N): 23.4 (15/64); past smoker, % (n/N): 12.5 (8/64) *Body Mass Index (BMI)*: in kg/m^2^, mean (SD): 24.9 (3.7) *Blood pressure status*: Mild or moderate hypertension, % (n/N): 100 (64/64) *Antihypertensive medication used*: % (n/N): 54.7 (35/64) *Cardiovascular disease or stroke*: Heart attack or stroke in past 6 months, current angina pectoris or congestive heart failure, % (n/N): 0 (0/64) *Diabetes mellitus*: % (n/N): 0 (0/64) *Renal impairment*: Serum creatinine, in µmol/L, mean (SD): 76.8 (19.0) *Dietary potassium intake*: NR *Dietary sodium intake*: NR *Urinary potassium excretion*: in mmol/24 h, mean (SD): 24.6 (8.12) *Urinary sodium excretion*: in mmol/24 h, mean (SD): 241 (46.2) **Inclusion criteria:** Men and women, aged between 50 and 80, willing to undertake long‐term use of the intervention salt; with serum potassium < 5.5 mmol/L, and net increase of < 1.0 mmol/L for serum potassium at the end of the run‐in period **Exclusion criteria:** Participants who ate more than one meal per week outside their homes; currently used potassium‐sparing medication/diuretics; already had a family member enrolled in the study; had a history of heart attack or stroke in the past six months, current angina pectoris, or congestive heart failure; had diabetes mellitus, serious mental or physical illnesses, malignancy, or secondary hypertension; or had impaired renal function **Pretreatment:** None **Method of recruitment of participants:** Han people from ten communities in the Hedong District, Tianjin were recruited. **Informed consent obtained:** Yes, unclear whether written or oral **Clusters:** n/a **Subgroups planned/measured:** Participants with normal blood pressure and participants with hypertension **Subgroups reported:** Participants with normal blood pressure and participants with hypertension *Participant flow* **Assessed for eligibility:** n = 264 **Excluded (number with reasons):** n = 16 (met exclusion criteria, < 5.5 mmol/L serum potassium or net change of <1.0 mmol/L serum potassium following run‐in) **Randomised:** n = 248 **Allocated to LSSS intervention(s):** n = 119 (n = 62 participants with hypertension; n = 57 participants with normal blood pressure) **Allocated to control:** n = 129 (n = 64 participants with hypertension; n = 65 participants with normal blood pressure) **Received allocated LSSS intervention(s):** NR **Did not receive allocated LSSS intervention(s):** NR **Lost to follow‐up (LSSS intervention group):** n = 13 (n = 10 (n = 8 participants with hypertension; n = 2 participants with normal blood pressure) withdrew; n = 3 participants with normal blood pressure lost to contact) **Discontinued intervention (LSSS intervention group):** n = 10 (n = 8 participants with hypertension; n = 2 participants with normal blood pressure) **Analysed (LSSS intervention group):** n = 119 (intention‐to‐treat with baseline values carried forward); n = 62 (participants with hypertension) n = 57 (participants with normal blood pressure) **Excluded from analysis (LSSS intervention group):** n = 0 **Received allocated control:** NR **Did not receive allocated control:** NR **Lost to follow‐up (control group):** n = 9 (n = 8 (n = 6 participants with hypertension; n = 2 participants with normal blood pressure) withdrew; n = 1 participants with normal blood pressure lost to contact) **Discontinued intervention (control group):** n = 8 (n = 6 participants with hypertension; n = 2 participants with normal blood pressure) **Analysed (control group):** n = 129 (intention‐to‐treat with baseline values carried forward) **Excluded from analysis (control group):** n = 0	
Interventions	**Intervention Characteristics** LSSS intervention *Theoretical basis*: Salt restriction is an important approach in the prevention of hypertension. *Description*: 'Compound ion' salt (CISalt) (65% NaCl, 30% KCl, 5% calcium salts) *LSSS category*: ≥ 30% KCl *Contains fortificant*: Folic acid (12 mg/kg) *Delivery*: discretionary use *Duration of run‐in period*: 4 weeks *Duration of active intervention*: 6 months *Duration of follow‐up (as reported)*: none *Total duration of study (as reported)*: 6 months *Timing*: LSSS to be used by the entire household for cooking and other purposes. *Implementation*: LSSS was provided in 1 kg bags, at 3 kg per household per month. *Providers*: LSSS was manufactured and packaged by Tianjin Kaifu S&T Development, supervised by the China National Center for Salt Product Quality Control. Details about persons delivering the intervention not reported *Co‐interventions*: NR *Resource requirements*: NR *Integrity of delivery*: Older persons were recruited, due to their regularised work‐rest schedules and predominant in‐home eating habits. The authors reported that compliance was attained over the course of the study. Control *Theoretical basis*: To enable comparison of LSSS intervention with standard practice *Description*: 'Normal' salt *Contains fortificant*: NR *Delivery*: discretionary use *Duration of run‐in period*: 4 weeks *Duration of active intervention*: 6 months *Duration of follow‐up (as reported)*: none *Total duration of study (as reported)*: 6 months *Timing*: Normal salt to be used by the entire household for cooking and other purposes. *Implementation*: Normal salt was provided in 1 kg bags, at 3 kg per household per month. *Providers*: Normal salt was manufactured and packaged by Tianjin Kaifu S&T Development, supervised by the China National Center for Salt Product Quality Control. Details about persons delivering the intervention not reported *Co‐interventions*: NR *Resource requirements*: NR *Integrity of delivery*: Older persons were recruited, due to their regularised work‐rest schedules and predominant in‐home eating habits. The authors reported that compliance was attained over the course of the study.	
Outcomes	**Primary outcomes: ** **Diastolic Blood Pressure (DBP):** Outcome measurement: Measurements performed in each participant's home using the auscultatory method by 2 experienced physicians. Time points: 2, 4, 6 months **Systolic Blood Pressure (SBP):** Outcome measurement: Measurements performed in each participant's home using the auscultatory method by 2 experienced physicians. Time points: 2, 4, 6 months **Hypertension (as reported, or SBP > 140 mmHg or DBP > 85 mmHg):** NR **Blood pressure control (achieving blood pressure threshold or 'control' as prespecified):** NR **Cardiovascular events‐stroke:** NR **Cardiovascular events‐myocardial infarction:** NR **Cardiovascular events‐other:** Adverse cardiovascular events; Outcome measurement: NR. Time points: duration of study **Cardiovascular mortality:** NR **Blood potassium: **NR **Hyperkalaemia:** NR **Hypokalaemia:** NR **Secondary outcomes:** **All‐cause mortality:** NR **Adverse events:** NR **Antihypertensive medication use:** NR **Body Mass index (BMI):** NR **Serum creatinine:** NR **Albuminuria:** NR **Urinary albumin‐to‐creatinine ratio (uACR):** NR **Fasting blood glucose:** Outcome measurement: Determined from venous blood samples drawn after overnight fasting using biochemical assay. Time points: 6 months **Blood triglycerides:** Outcome measurement: Determined from venous blood samples drawn after overnight fasting using biochemical assay. Time points: 6 months **Total blood cholesterol:** Outcome measurement: Determined from venous blood samples drawn after overnight fasting using biochemical assay. Time points: 6 months **24‐h urinary sodium excretion:** 24‐hour urine sample. Time points: 6 months **24‐h urinary potassium excretion:** 24‐hour urine sample. Time points: 6 months	
Notes	**Funding source:** NR **Authors name:** Xin Zhou and Yu‐Ming Li **Institution:** Institute of Cardiovascular Disease and Division of Cardiology, Pingjin Hospital, Medical College of the Chinese People's Armed Police Forces, Tianjin **Email:** cardiolab@gmail.com **Possible conflicts of interest (for study authors):** None **Sources used for data extraction:** Journal article of meeting report, with results of the trial **Trial registration details:** NR	
***Risk of bias***		
**Bias**	**Authors' judgement**	**Support for judgement**
Random sequence generation (selection bias)	Low risk	Randomisation was done using a computer‐generated random sequence.
Allocation concealment (selection bias)	Low risk	Randomisation sequence numbers were placed in sealed envelopes. Intervention and control bags were manufactured by a supplier and only identified with the randomisation sequence.
Blinding of participants and personnel (performance bias) All outcomes	Low risk	Participants were blinded to assignment; no information on whether people delivering the intervention were blinded. Given that participants were blinded and minimal interventionist involvement was reported, the study is not likely to be prone to performance bias.
Blinding of outcome assessment (detection bias) All outcomes	Low risk	Outcome assessors were not blinded. Outcomes which may be prone to detection bias, e.g. auscultatory determination of blood pressure, were done by two experienced physicians. Other outcomes were not prone to detection bias as they were laboratory‐determined, or self‐reported by blinded participants.
Incomplete outcome data (attrition bias) All outcomes	Low risk	Overall attrition was 22/248 (9%), with 13/248 (5%) in the intervention and 9/248 (4%) in the control group; and similar reasons for dropouts reported per group.
Selective reporting (reporting bias)	Unclear risk	No study protocol or prospectively trial registry entry available
Other bias	Low risk	None identified
Overall risk of bias	Low risk	Low risk of bias for allocation concealment and incomplete outcome data

**Table:** Zhou 2013

***Study characteristics***		
Methods	**Study design:** Cluster‐randomised controlled trial **Country:** China **Setting:** Intervention conducted in families from five villages in Liaoning, rural North China; setting where outcomes were measured not reported, but presumably at participants' homes **Aim of study:** To investigate the effect of the long‐term use of low‐sodium salt substitutes, to reduce dietary sodium intake, on the blood pressure of people with normal blood pressure and people who are moderately hypertensive **Unit of allocation:** Families **Start date:** NR **End date:** NR **Relevant study limitations as reported by study authors:** Sample sizes were too small to adequately explore the effect of LSSS on blood pressure in various age subgroups; variations in blood pressure were observed throughout the year, which the authors attributed to seasonal changes; baseline blood pressure was higher in the intervention group than in the control group; first morning urine instead of 24‐hour urine samples were collected at 24‐month follow‐up as a previous study reported good safety, and no significant difference in urinary electrolytes at this time point should be interpreted with caution. **Sample size calculation:** NR	
Participants	**Baseline Characteristics** LSSS intervention *Age*: in years, mean (SD): 45.63 (13.72) *Gender*: Female, % (n/N): 50.45 (113/224) *Ethnicity/race*: Chinese *Smoking*: % (n/N): 45.09 (101/224) *Body Mass Index (BMI)*: in kg/m^2^, mean (SD): 25.94 (3.82) *Blood pressure status*: Family history of hypertension, % (n/N): 64 (143/224); history of hypertension, % (n/N): 75 (169/224) *Antihypertensive medication used*: Medication use, mainly for hypertension and CVD (captopril, nifedipine or compound reserpine), % (n/N): 41.07 (92/224) *Cardiovascular disease or stroke*: Cardiovascular and cerebrovascular diseases, % (n/N): 17.86 (40/224) *Diabetes mellitus*: Treated diabetes, % (n/N): 1.34 (3/224) *Renal impairment*: Significant renal impairment, % (n/N): 0 (0/224) *Dietary potassium intake*: NR *Dietary sodium intake*: NR *Urinary potassium excretion*: subsample (N = 80); in mmol/L, median (IQR): 26 (17 to 42) *Urinary sodium excretion*: subsample (N = 80); in mmol/L, median (175 (139 to 201) Control *Age*: in years, mean (SD): 47.05 (13.46) *Gender*: Female, % (n/N): 50.84 (121/238) *Ethnicity/race*: Chinese *Smoking*: %, (n/N): 39.5 (94/238) *Body Mass Index (BMI)*: in kg/m^2^, mean (SD): 26.66 (4.27) *Blood pressure status*: Family history of hypertension, % (n/N): 50 (118/238); history of hypertension, % (n/N): 74 (176/238) *Antihypertensive medication used*: Medication use, mainly for hypertension and CVD (captopril, nifedipine or compound reserpine), % (n/N): 40 (94/238) *Cardiovascular disease or stroke*: Cardiovascular and cerebrovascular diseases, % (n/N): 13.02 (31/238) *Diabetes mellitus*: Treated diabetes, % (n/N): 2.94 (7/238) *Renal impairment*: Significant renal impairment, % (n/N): 0 (0/238) *Dietary potassium intake*: NR *Dietary sodium intake*: NR *Urinary potassium excretion*: subsample (N = 80); in mmol/L, median (IQR): 25 (16 to 42) mmol/L *Urinary sodium excretion*: subsample (N = 80); in mmol/L, median (IQR)170 (134 to 233) **Inclusion criteria:** Families: At least one family member is a patient with hypertension, with systolic blood pressure > 140 mmHg or diastolic blood pressure > 90 mmHg and with an estimated daily sodium intake of ≥ 260 mmol/day Participants: Aged at least 18 years **Exclusion criteria:** Families: Member of the family with hypertension moving away during the study period. Participants: Family members with significant renal impairment or other indication for using potassium‐sparing medication, normotensive family members who move away during the study period **Pretreatment:** Participants in the LSSS intervention group had higher alcohol consumption (P < 0.01) and were more likely to have a family history of hypertension (P < 0.01) compared to those in the control group. **Method of recruitment of participants:** NR **Informed consent obtained:** yes (written) **Clusters:** n = 200 clusters with n = 462 participants; average cluster size (number of family members) not reported; no adjustment for clustering **Subgroups planned/measured:** Age and gender **Subgroups reported:** Age (<= 40 years vs. 40 to 50 years vs. 50 to 60 years vs. 60 to 70 years vs. > 70 years) and gender *Participant flow* **Assessed for eligibility:** n = 223 clusters with n = 543 participants **Excluded (number with reasons):** n = 23 clusters with n = 81 participants (n = 3 clusters with n = 12 people refused to participate; n = 20 clusters with n = 69 people did not meet inclusion criteria; with n = 1 cluster having member(s) using potassium‐sparing medication, n = 7 clusters having members with renal disease and n = 12 clusters not resident) **Randomised:** n = 200 clusters with n = 462 participants **Allocated to LSSS intervention(s):** n = 100 clusters with n = 224 participants **Allocated to control:** n = 100 clusters with n = 238 participants **Received allocated LSSS intervention(s):** NR **Did not receive allocated LSSS intervention(s):** NR **Lost to follow‐up (LSSS intervention group):** n = 11 clusters with n = 41 participants (n = 7 died, n = 15 lost contact, n = 10 reluctant to follow up, and n = 9 withdrew; distribution by cluster not reported) at 2 years. At 3 years: n = 0 (n = 183 individuals completed follow‐up) **Discontinued intervention (LSSS intervention group):** At 2 years: n = 9 withdrew **Analysed (LSSS intervention group):** At 2 years: n = 224 participants (ITT analysis; ITT method not described) **Excluded from analysis (LSSS intervention group):** n = 0 participants **Received allocated control:** NR **Did not receive allocated control:** NR **Lost to follow‐up (control group):** n = 7 clusters with n = 49 participants (n = 8 died, n = 23 lost contact, and n = 18 withdrew; distribution by cluster not reported). At 3 years: n = 1 (n = 188 individuals completed follow‐up) **Discontinued intervention (control group):** At 2 years: n = 18 withdrew **Analysed (control group):** At 2 years: n = 238 participants (ITT analysis; ITT method not described) **Excluded from analysis (control group):** n = 0 participants	
Interventions	**Intervention Characteristics** LSSS intervention *Theoretical basis*: LSSS may reduce BP of people with normal blood pressure and individuals with hypertension over the long term by lowering sodium intake and by the provision of potassium. *Description*: Salt substitute (65% NaCl, 25% KCl and 10% MgSO_4_) for food preparation *LSSS category*: < 30% KCl *Contains fortificant*: NR *Delivery*: discretionary use *Duration of run‐in period*: none *Duration of active intervention*: 3 years *Duration of follow‐up (as reported)*: none *Total duration of study (as reported)*: 3 years *Timing*: Households instructed to prepare all foods with study salt for the duration of the study *Implementation*: Each family provided with identical 1 kg bags every 3 months, according to estimated baseline salt intake of the household. The same amount was given at all subsequent visits. *Providers*: Study investigators provided the LSSS to participants at each study visit. The LSSS was manufactured, packaged and labelled by the Shenyang Hongmei Salt Industrial Company in accordance with Chinese Manufacturing Standards. *Co‐interventions*: NR *Resource requirements*: NR *Integrity of delivery*: Families were questioned about their salt consumption at each study visit. Some participants reported that LSSS tasted 'light' in comparison to usual salt; some families added comparable amounts of LSSS to food while others added more to compensate for this change in taste. Control *Theoretical basis*: To enable comparison of LSSS intervention with standard practice *Description*: 'Normal' salt (100% NaCl) for food preparation *Contains fortificant*: NR *Delivery*: discretionary use *Duration of run‐in period*: none *Duration of active intervention*: 3 years *Duration of follow‐up (as reported)*: none *Total duration of study (as reported)*: 3 years *Timing*: Households instructed to prepare all foods with study salt for the duration of the study. *Implementation*: Each family provided with identical 1 kg bags every 3 months, according to estimated baseline salt intake of the household. The same amount was given at all subsequent visits. *Providers*: Study investigators provided the normal salt to participants at each study visit. Normal salt for the study was manufactured, packaged and labelled by the Shenyang Hongmei Salt Industrial Company in accordance with Chinese Manufacturing Standards. *Co‐interventions*: NR *Resource requirements*: NR *Integrity of delivery*: Families were questioned re. their salt consumption at each follow‐up visit. Data NR.	
Outcomes	**Primary outcomes: ** **Diastolic Blood Pressure (DBP):** Outcome measurement: Measured at each visit by trained doctors using an automatic sphygmomanometer in the seated position. The mean of two readings used for the analysis, the interval between the readings was at least 2 min. Time points: 3, 6, 9, 12, 15, 18, 21, 24, 30, 33 and 36 months **Systolic Blood Pressure (SBP):** Outcome measurement: Measured at each visit by trained doctors using an automatic sphygmomanometer in the seated position. The mean of two readings used for the analysis, the interval between the readings was at least 2 min. Time points: 3, 6, 9, 12, 15, 18, 21, 24, 30, 33 and 36 months **Hypertension (as reported, or SBP > 140 mmHg or DBP > 85 mmHg):** NR **Blood pressure control (achieving blood pressure threshold or 'control' as prespecified):** NR **Cardiovascular events‐stroke:** NR **Cardiovascular events‐myocardial infarction:** NR **Cardiovascular events‐other:** NR **Cardiovascular mortality:** NR **Blood potassium:** NR **Hyperkalaemia:** NR **Hypokalaemia:** NR **Secondary outcomes: ** **All‐cause mortality:** Outcome measurement: NR. Time points: duration of study **Adverse events:** NR **Antihypertensive medication use:** Outcome measurement: questionnaire‐based report of hypotensive medication use. Time points: 3, 6, 9, 12, 15, 18, 21, 24, 30, 33 and 36 months **Body Mass index (BMI):** NR **Serum creatinine:** NR **Albuminuria:** NR **Urinary albumin‐to‐creatinine ratio (uACR):** NR **Fasting blood glucose:** NR **Blood triglycerides:** NR **Total blood cholesterol:** NR **24‐h urinary sodium excretion:** NR **24‐h urinary potassium excretion:** NR	
Notes	**Funding source:** Programme of the National Natural Science Foundation of China **Authors name:** Bo Zhou and Jing‐Pu Shi **Institution:** Department Cardiovascular of Clinical Epidemiology, The First Hospital of China Medical University and Institute of China Medical University, China Medical University, Shenyang, China **Email:** sjp56@sina.com; sjp562013@163.com **Possible conflicts of interest (for study authors):** "The authors declare no conflict of interest." **Sources used for data extraction:** Journal articles with results of the trial **Trial registration details:** NR	
***Risk of bias***		
**Bias**	**Authors' judgement**	**Support for judgement**
Random sequence generation (selection bias)	Low risk	Clusters were randomised based on a computer‐generated randomisation scheme.
Allocation concealment (selection bias)	Unclear risk	Intervention and control salts were packaged in identical bags and were only identified by a three‐digit code; they were packaged and labelled by an external salt manufacturing company, but insufficient information on how the randomisation sequence was protected
Blinding of participants and personnel (performance bias) All outcomes	Low risk	Participants and investigators were blinded to treatment allocation ‐ LSSS and control salt packaged in identical bags with three‐digit codes (unlocked at the end of the study).
Blinding of outcome assessment (detection bias) All outcomes	Unclear risk	It was not clear whether outcome assessors were blinded to allocation, but the clinical and mortality outcomes of the study were objective and unlikely to be influenced by a lack of blinding. Blood pressure was measured with an automatic sphygmomanometer. Any self‐reported outcomes were assessed by participants ‐ not clear whether they were still blinded at 36 months.
Incomplete outcome data (attrition bias) All outcomes	Low risk	Attrition was high in both groups at 24‐month follow‐up: LSSS intervention group 18.3% (41/22) vs. control group 20.6% (49/238). However, the study authors reported minimal attrition at the 36‐month follow‐up (LSSS intervention group 18.3% (41/224) vs. control group 20.6% (50/238)). Blood pressure outcomes were assessed using an intention‐to‐treat approach with mixed linear models; it was not clear what the ITT approach was for other outcomes.
Selective reporting (reporting bias)	Unclear risk	Study protocol or prospective trial registry entry not available
Other bias	Low risk	None identified
Recruitment bias (cluster‐RCTs)	Unclear risk	It was not clear whether all the individual participants were identified before the randomisation of the families.
Comparability with individually randomised trials (cluster‐RCTs)	Low risk	The China Salt Substitute Study also found no change in DBP during a one‐year follow‐up; another RCT by Little 2004 found no decrease in SBP and DBP in persons randomised to LSSS and lifestyle interventions at six months follow‐up; the authors report interpreted these findings as being reflected by DBP only changing after 18 months in the present study.
Loss of clusters (cluster‐RCTs)	Unclear risk	Loss of clusters (families) was similar for both groups (LSSS intervention group 11/100; control 7/100). Although the study authors stated that the main reason for the loss of clusters was that participants withdrew from the study, they did not comment on any differences in baseline characteristics of families who were lost to follow‐up, compared to those who remained in the study.
Baseline imbalance (cluster‐RCTs)	Low risk	Baseline imbalances were found for family history of hypertension and alcohol consumption, both of which were significantly higher in the intervention group (P = 0.007 and P = 0.012, respectively). Analyses were adjusted for these covariates and were reported as showing that these imbalances were due to chance.
Incorrect analysis (cluster‐RCTs)	High risk	Adjustment to account for the effect of clustering was not reported in the statistical analysis or results section.
Overall risk of bias	Unclear risk	Low risk of bias for incomplete outcome data and baseline imbalance; unclear risk of bias for loss of clusters and recruitment bias

ACE: angiotensin‐converting enzyme AE: adverse event Alx: aortic pressure augmentation index AMI: acute myocardial infarction ARB: angiotensin receptor blocker AUG: aortic pressure augmentation BMI: body mass index BP: blood pressure Ca: calcium CDP: central diastolic pressure CPP: central pulse pressure CSP: central systolic pressure CSSS: China Salt Substitute Study CVD: cardiovascular disease DBP: diastolic blood pressure DM: diabetes mellitus (e)GFR: (estimated) glomerular filtration rate FFQ: food frequency questionnaire GEE: generalised estimating equation ICC: intra‐cluster correlation coefficient ICD‐9/10: International Classification of Diseases (ninth revision/tenth revision) IQR: interquartile range ITT: intention‐to‐treat Kcal: kilocalorie K(Cl): potassium (chloride) LSSS: low‐sodium salt substitute MAPA: ambulatory monitoring of arterial pressure/ambulatory blood pressure monitoring MgSO_4_: magnesium sulfate mmHg: millimeters of mercury MSG: monosodium glutamate n/a: not applicable Na(CI): sodium (chloride) NR: not reported NSS: non‐salt‐sensitive PEN: Peruvian Sol pm: post meridiem RCT: randomised controlled trial RT: reflection time SBP: systolic blood pressure SD: standard deviation SS: salt‐sensitive SSaSS: Salt Substitute and Stroke Study SSiIS: Salt Substitute in India Study uACR: urinary albumin‐to‐creatinine ratio WHO: World Health Organization

### Characteristics of excluded studies [ordered by study ID]

**Table:**

Study	Reason for exclusion
Allaert 2015	Wrong study design (single‐arm trial)
Baek 2015	Wrong comparator (sodium intake identical between LSSS and control group)
Barros 2013	Wrong study design (quasi‐randomised controlled trial)
Barros 2015	Wrong study design (quasi‐randomised controlled trial)
ChiCTR1800016804	Wrong type of intervention (salt reduction intervention strategy)
ChiCTR1800018119	Multifactorial intervention ‐ could not isolate LSSS effect
ChiCTR1800019727	Case series
ChiCTR2000033349	Multifactorial intervention ‐ could not isolate LSSS effect
Dent 2011	Wrong study design
Doorenbos 2003	Wrong study design (case study)
Farrand 2019	Wrong study design (commentary)
He 2020	Wrong study design
Hueston 1989	Wrong study design (case series)
Itoh 1997	Wrong study design
Janda 2017	Wrong study design (non‐randomised trial)
Jeffrey 1984	Wrong comparator (low sodium high potassium diet)
JPRN‐UMIN000012560	Wrong outcomes (sensory/organoleptic outcomes)
Karppanen 1984	Wrong study design (cross‐over study but first‐phase data unavailable)
Katz 1999	Wrong study design (single‐arm trial)
Lambert 2019	Wrong study design (letter in response to excluded review by Farrand 2019)
Little 2004	Multifactorial intervention ‐ could not isolate LSSS effect
Maleki 2016	Wrong outcomes (sensory/organoleptic outcomes)
Maruya 2020	Wrong outcomes (sensory/organoleptic outcomes)
Matlou 1986	Wrong type of intervention (potassium chloride salt administered as a supplement; not instead of sodium chloride)
Mu 2020	Wrong study design (single‐arm trial)
Nakano 2016	Wrong type of intervention (salt‐restriction education)
NCT02105727	Wrong study design (single‐arm trial) (NCT02105727)
Pietinen 1981	Wrong study design (non‐randomised trial)
Robare 2010	Wrong type of intervention (dietary sodium reduction intervention)
Salvetti 1988	Wrong comparator (metoprolol treatment)
Sciarrone 1992	Wrong type of intervention (low‐sodium low‐fat high fibre diet)
Zoccali 1985	Wrong comparator (lactose placebo)

LSSS: low‐sodium salt substitute

### Characteristics of studies awaiting classification [ordered by study ID]

**Table:** Jones 2019

Methods	Design: Proposed RCT Country: USA Setting: NR
Participants	Inclusion criteria: age > 50 years with at least one of the following: presence of clinical or subclinical cardiovascular disease (pooled Cohort Equation Risk Score for 10‐year cardiovascular disease risk > 15%) or chronic kidney disease (defined as eGFR 20–59 mL/min/1.73 m^2^) or age > 70 years Exclusion criteria: NR
Interventions	Combination intervention of diet education, primarily focussed on increased dietary potassium, and the use of a potassium‐based salt substitute for food preparation Comparator: Traditional diet
Outcomes	Acute coronary syndrome (ACS), stroke, congestive heart failure (CHF), cardiovascular (CV) death, and a 15% change in eGFR
Notes

**Table:** Neutel 1996

Methods	Design: Double‐blind, placebo‐controlled clinical study Country: NR Setting: NR
Participants	Inclusion criteria: Patients with mild to moderate hypertension Exclusion criteria: NR
Interventions	Intervention: Reduced sodium salt containing potassium and magnesium Comparator: Regular salt
Outcomes	Lowering blood pressure
Notes

**Table:** Voloshyna 2017

Methods	Design: RCT Country: Ukraine Setting: NR
Participants	Inclusion criteria: Symptomatic patients with chronic heart failure aged 66 to 80 years on stable treatment with three or more drugs and baseline serum potassium levels 3.9 to 4.7 mmol/L Exclusion criteria: NR
Interventions	Intervention: 5 to 6 g/day of potassium‐enriched salt (30% potassium; 5% magnesium) Comparator: Moderately salt‐restricted diet (5 to 6 g salt/day)
Outcomes	Physical function assessment, quality of life, health status, daily activity
Notes	

ACS: acute coronary syndrome CHF: congestive heart failure CV: cardiovascular eGFR: estimated glomerular filtration rate NR: not reported RCT: randomised controlled trial

### Characteristics of ongoing studies [ordered by study ID]

**Table:** ACTRN12619000352101

Study name	Salt ALTernatives Study (SALTS): a smartphone app and dietary alternative salt to lower blood pressure for adults with high blood pressure
Methods	Design: RCT Country: New Zealand Setting: Household and supermarkets, Auckland
Participants	Inclusion criteria: >= 18 years or older who have a SBP >= 140 mmHg and/or DBP >= 85 mmHg Exclusion criteria: Severe kidney disease; use of a potassium‐sparing diuretic, furosemide, NSAIDs, prednisone; history of a stroke or cardiovascular event (hospitalisation for heart attack, coronary artery revascularisation (CABG or stenting), stroke or heart failure) in the previous 6 months; diagnosed heart failure
Interventions	Intervention: Consists of two components: (1) a smartphone application (app) called SaltSwitch (helps users to select lower salt packaged foods by enabling them to scan the barcode of a packaged food and receive an immediate, interpretive traffic light nutrition label on‐screen plus suggestions for lower salt alternatives), and (2) a dietary alternative salt (74.5% potassium chloride and 24.5% sodium chloride and ~1% silicon dioxide) Comparator: Generic information about heart‐healthy eating from the Heart Foundation of New Zealand
Outcomes	Primary: SBP (week 12) Secondary: SBP (week 6); urinary sodium; sodium content of packaged food purchases; BP control (135/85 mmHg); use of SaltSwitch app; use of dietary alternative salt
Starting date	May 2019
Contact information	Contact person(s): Helen Eyles Email: h.eyles@auckland.ac.nz
Notes	Sponsors and Collaborators: Health Research Council of New Zealand

**Table:** ChiCTR08000296

Study name	China Salt trial
Methods	Design: Cluster‐randomised trial Country: China Setting: Families/households, Shandong province
Participants	Inclusion criteria: families with adults > 18 years with treated or untreated hypertension who has an estimated salt intake > 200 mmol/d with 50% of the salt added in the cooking or at the table (estimated through an interview with the participant) Exclusion criteria: Chronic renal disease with renal impairment, e.g. nephritis, nephrotic syndrome, nephrolithiasis, renal cysts; plasma creatinine > 1.7 mg/dL or eGFR < 40 mL/min; plasma K > 4.5 mmol/L at start or > 5.0 mmol/L at end of 1 month run‐in; use of potassium retaining drugs or NSAIDs; malignancy during the past 5 years; any other serious life‐threatening illness that required regular medical treatment; psychiatric disease; pregnancy or intent to become pregnant during the study
Interventions	Intervention: Supply of table salt containing equal amounts by weight of NaCl and KCl to be used in cooking and at the table or with education materials and with appropriate items to reduce their NaCl intake to 6 g NaCl/day Comparator: Supply of normal salt from market to be used in cooking and at the table
Outcomes	Blood pressure, urine Na and K concentration, microalbuminuria, plasma renin activity, plasma aldosterone
Starting date	May 2009
Contact information	Contact person(s): Jichun Chen; Lisheng Liu, Dongfeng Gu Email: jcch70@sina.com; lshypt@yahoo.com.cn
Notes	Sponsors and Collaborators: Fuwai Hospital

**Table:** ChiCTR09000538

Study name	The study on pathogeny of hypertension and the salt intervention on diet in the population of Zhangwu County
Methods	Design: RCT Country: China Setting: Families or households
Participants	Inclusion criteria: Families with at least one adult (aged 18 to 70 years) with hypertension (SBP > 140 mmHg or DBP > 90 mmHg). Diagnosis of stroke, TIA and coronary heart disease made by the hospital of the county or higher level. Exclusion criteria: Liver or kidney disease
Interventions	Interventions: Salt with lower sodium (Mg 10%, K 25%, Na 65%) Comparator: Common iodine salt
Outcomes	Primary: Blood pressure Secondary: Salty feeling tendency, urinary sodium and potassium
Starting date	01 June 2005
Contact information	Contact person(s): Gang Lv, Jingpu Shi Email: sjp56@yahoo.com
Notes	Sponsors and Collaborators:; Science and technology projects of Liaoning Province; the First Hospital of China Medical University

**Table:** ChiCTR2000029017

Study name	Study on the application of low‐sodium formula salt in hypertensive patients with diabetes
Methods	Design: RCT Country: China Setting: Chongqing Nan'an District People's Hospital
Participants	Inclusion criteria: 35 to 75 years with diabetes and hypertension, currently taking medications to lower blood sugar and blood pressure (the type and dose remain unchanged for > 3 months) with baseline untreated systolic blood pressure between 130‐159 mmHg, and diastolic blood pressure between 80‐100 mmHg Exclusion criteria: Malignancy, acute myocardial infarction and acute stroke within three months or expected survival time is < 1 year; secondary hypertension (hypercortisolism or hyperaldosteronism); acute diseases; deafness, dementia and other disabilities, as well as severe depression or other mental disorders; abnormal liver function, renal dysfunction (stage 4 and 5 chronic kidney disease); serum potassium < 3.5 mmol/L or > 5.5 mmol/L; use of potassium diuretics; pregnant women, or those with other serious chronic diseases
Interventions	Intervention: DASH diet and 65% low‐sodium salt; DASH diet and 18% low‐sodium salt Comparator: DASH diet and common salt
Outcomes	Primary: Blood pressure, fasting blood glucose, 2‐hour postprandial glucose
Starting date	February 2020 (not yet recruiting)
Contact information	Contact person(s): Huakun Rao; Lihong Mu Email: 931575032@qq.com; 1097123703@qq.com
Notes	Sponsors and Collaborators: Chongqing Science and Technology Bureau

**Table:** IRCT2016103130572N1

Study name	The effects of a salt substitute on an Iranian population
Methods	Design: RCT Country: Iran Setting: Preventive & Health Promoting Clinic, Baharlou Hospital, Tehran
Participants	Inclusion criteria: 25‐65 years; SBP > 140 mmHg or DBP > 90 mmHg in three measurements in two settings or consuming an antihypertensive drug Exclusion criteria: Abnormal serum creatinine or urea; consuming medications: digoxin, lithium, K‐sparing diuretics; acute or severe cardiovascular disease or psychotic disorders
Interventions	Intervention: Low‐sodium salt (80 percent sodium chloride and 20 percent potassium chloride) Comparator: Regular salt (100 percent sodium chloride)
Outcomes	Primary: SBP; DBP; 24‐hour urinary sodium and potassium
Starting date	December 2016
Contact information	Contact person(s): Mohammad Mohammadi Email: mohammadi.mo@razi.tums.ac.ir
Notes	Sponsors and Collaborators: Research Deputy of Tehran University of Medical Sciences; Shournamak Yazd Company

**Table:** NCT02016404

Study name	The effect of a low sodium‐high potassium salt on blood pressure in Vietnamese adults
Methods	Design: RCT Country: Vietnam Setting: Hai Ba Trung district, Hanoi
Participants	Inclusion criteria: Adults aged between 45 and 64 years with untreated prehypertension or mild hypertension (systolic BP between 130 and 160 mmHg and/or diastolic BP between 85 and 100 mmHg) based on the mean of two measurements with a 1‐week interval Exclusion criteria: Diagnosed or self‐reported cardiovascular or kidney disease
Interventions	Intervention: Iodised low‐sodium, high‐potassium botcanh (a traditional mixture of salt, MSG, sugar and herbs) plus and iodised low‐sodium, high‐potassium salt for home food preparation Comparator: Regular iodised high‐sodium botcanh (a traditional mixture of salt, MSG, sugar and herbs) and high‐sodium salt
Outcomes	Primary: SBP, DBP Secondary: 24‐hour urinary sodium, potassium, calcium, creatinine, protein, iodine; body weight; heart rate
Starting date	June 2013
Contact information	Contact person(s): Ha TP Do Email: NR
Notes	Sponsors and Collaborators: Wageningen University; National Institute of Nutrition, Vietnam

**Table:** NCT02021435

Study name	Tibet salt reduction study
Methods	Design: Cluster‐randomised trial Country: Tibet Setting: Villages from Damxung and Maizhokunggar county
Participants	Inclusion criteria: All family households (including children and adults) in selected villages Exclusion criteria (families): any family member reporting the use of a potassium‐sparing diuretic or potassium supplement; has serious renal impairment or life expectancy < 6 months
Interventions	Intervention: Salt substitute provided to households free of charge. To receive health education on salt reduction Comparator: Buy own normal salt. To receive health education on salt reduction
Outcomes	Primary: Blood pressure Secondary: Total mortality, cardiovascular mortality, life expectancy, serum cholesterol and random blood glucose levels (> 60 years)
Starting date	April 2014
Contact information	Contact person(s): Xingshan Zhao Email: xingshanzh@gmail.com
Notes	Sponsors and Collaborators: Beijing Jishuitan Hospital; the George Institute for Global Health at PUHSC; the George Institute for Global Health, Australia; Science and Technology Department of Ningxia; People's Hospital of Tibet; People's Hospital of Maizhokunggar; Tibet University; Peking University; People's Hospital of Damxung County, Tibet

**Table:** NCT03290716

Study name	Diet, exercise and cardiovascular health ‐ effect of salt substitute and stepwise salt supply control in reducing blood pressure in the elderly in nursing homes in China
Methods	Design: Cluster‐randomised trial Country: China Setting: Nursing Homes, Jincheng, Shanxi
Participants	Inclusion criteria (nursing homes): nursing homes with at least 20 residents; Inclusion criteria (individuals): Permanent residents with life expectancy over six months Exclusion criteria: None
Interventions	Intervention: Salt substitute only: Replace regular salt with market available potassium‐enriched salt substitute in kitchens of nursing homes Stepwise salt supply control only: A stepwise approach to reduce salt used in the kitchen of nursing homes by controlling the supply of salt; or Salt substitute plus stepwise salt supply control: combination of both interventions Comparator: No intervention
Outcomes	Primary: SBP Secondary: Hyperkalaemia; hyponatremia; 24‐hour urinary sodium and potassium; DBP; cardiovascular events; total mortality; incremental cost‐effectiveness ratio
Starting date	25 September 2017
Contact information	Contact person(s): Yangfeng Wu Email: NR
Notes	Sponsors and Collaborators: Peking University; Changzhi Medical College; Xian Jiaotong University; Hohhot Center for Disease Control and Prevention; Yangcheng Ophthalmology Hospital

BP: blood pressure CABG: coronary artery bypass graft DASH: Dietary Approaches to Stop Hypertension DBP: diastolic blood pressure eGFR: estimated glomerular filtration rate K(Cl): potassium (chloride) Mg: magnesium MSG: monosodium glutamate Na(Cl): sodium (chloride) NSAID: non‐steroidal anti‐inflammatory drug RCT: randomised controlled trial SBP: systolic blood pressure TIA: transient ischaemic attack

## Differences between protocol and review

*Populations at risk of hyperkalaemia:* The PICO was initially conceptualised into six comparisons by the WHO NUGAG Subgroup on Diet and Health, such that effects of LSSS in populations at possible risk of hyperkalaemia would be explored by stratifying the data into three comparisons in each of the subpopulations, namely adults, children, pregnant women possibly at risk of hyperkalaemia. During the guideline development process, the WHO NUGAG requested that effects of LSSS in populations at possible risk of hyperkalaemia rather be explored by subgroup analysis instead of by this stratification, and only for the safety outcomes (change in blood potassium, hyperkalaemia, hypokalaemia, adverse events, renal function and hyponatraemia). 

The WHO NUGAG decided to exclude the effectiveness outcomes (change in DBP, change in SBP, hypertension, blood pressure control, cardiovascular events, cardiovascular mortality, all‐cause mortality, antihypertensive medication use, change in fasting blood glucose, change in blood triglycerides, change in total blood cholesterol, change in 24‐h urinary sodium and potassium excretion as well as diagnosis of diabetes mellitus and change in BMI (for adults) and growth changes, bone densitometry and bone health (for children)) from this subgrouping, since there are no clinical justifications to expect differences in effectiveness outcomes in the ‘at risk’ populations, and their ‘at risk status’ is related specifically to the safety outcomes, which are primarily linked to potassium metabolism. The sodium in most of the low‐sodium salt substitutes is partially replaced by potassium resulting in an increase in potassium intake with their use. 

*Change to original title:* We removed 'renal health' from the original title to ensure the title more accurately reflected the focus of the primary cardiovascular outcomes of the review, since renal outcomes were only included as secondary outcomes.

*Screening:* The protocol stated that 'an initial electronic title screen using keywords to remove records that are obviously irrelevant' would be conducted. We replaced this with a full duplicate and independent screening process of all records yielded by the searches.

*Measures of treatment effect:* The search update in August 2022 resulted in the inclusion of a trial reporting event rates. In addition, a stepped‐wedge trial reporting hazard ratios for incident hypertension was included in the review. The analytical approaches to meta‐analysis of these data were added to the Methods section.

*Additional outcomes*: Two additional outcomes (change in 24‐hour urinary sodium and potassium excretion) were added by WHO NUGAG following the guideline development process, and were consequently incorporated into the review. A third outcome in children (all‐cause mortality) was erroneously omitted from the protocol and was included in the review.

*Subgroups:* During the guideline development process, it was decided that the subgroups total potassium intake and total sodium intake were not important to include, and were therefore excluded. The subgroup listed as duration of intervention in the protocol is included in the review as duration of study.

*Sensitivity analyses:* The protocol stated that 'We will also consider other potential sources of heterogeneity, such as methodological sources (using sensitivity analysis)', but did not specify the exact sensitivity analyses that were planned. Sensitivity analyses investigating the effect of excluding trials at high risk of bias and excluding trials with clusters as the unit of allocation on primary outcomes were reported in the methods conducted in the review.
